# Development and validation of a machine-learning-based model for identification of genes associated with sepsis-associated acute kidney injury

**DOI:** 10.3389/fgene.2025.1561331

**Published:** 2025-07-22

**Authors:** Chen Lin, Meng Zheng, Wensi Wu, Zhishan Wang, Guofeng Lu, Shaodan Feng, Xinlan Zhang

**Affiliations:** ^1^Department of Emergency, The Third Affiliated People’s Hospital, Fujian University of Traditional Chinese Medicine, Fuzhou, China; ^2^Hemodialysis Center, The Third Affiliated People’s Hospital, Fujian University of Traditional Chinese Medicine, Fuzhou, China; ^3^Department of Emergency, The First Affiliated Hospital, Fujian Medical University, Fuzhou, China

**Keywords:** sepsis, acute kidney injury, machine learning, diagnostic modeling, immune infiltration

## Abstract

**Background:**

Sepsis frequently induces acute kidney injury (AKI), and the complex interplay between these two conditions worsens prognosis, prolongs hospitalization, and increases mortality. Despite therapeutic options such as antibiotics and supportive care, early diagnosis and treatment remain a challenge. Understanding the underlying molecular mechanisms linking sepsis and AKI is critical for the development of effective diagnostic tools and therapeutic strategies.

**Methods:**

We used two sepsis (GSE57065 and GSE28750) and three AKI (GSE30718, GSE139061, and GSE67401) datasets from the NCBI Gene Expression Omnibus (GEO) for model development and validation, and performed batch effect mitigation, differential gene, and functional enrichment analysis using R software packages. We assessed 113 combinations of 12 different algorithms to develop an internally and externally validated machine-learning model for diagnosing AKI. Finally, we used functional enrichment analysis to identify potential therapeutic agents for AKI.

**Results:**

We identified 556 and 725 DEGs associated with sepsis and AKI, respectively, with 28 overlapping genes suggesting shared pathways. Functional enrichment analysis revealed important associations of AKI with immune responses and cell adhesion processes. The immune infiltration analysis showed significant differences in immune cell presence between sepsis and AKI patients compared with the control group. The machine-learning models identified eight key genes (*NR3C2*, *PLEKHO1*, *CEACAM1*, *CDC25B*, *HEPACAM2*, *VNN1*, *SLC2A3*, *RPL36*) with potential for diagnosing AKI. The diagnostic performance of the model constructed in this way was excellent (area under the curve = 0.978), especially in the under 60 years and male patient subgroups. The diagnostic performance outperformed previous models in both the training and validation sets. In addition, cyclosporin A and nine other drugs were identified as potential agents for treating sepsis-associated AKI.

**Conclusion:**

This study highlights the potential of integrating bioinformatics and machine-learning approaches to generate a new diagnostic model for sepsis-associated AKI using molecular crossovers with sepsis. The genes identified have potential to serve as biomarkers and therapeutic targets, providing avenues for future research aimed at enhancing sepsis-associated AKI diagnosis and treatment.

## 1 Introduction

Sepsis and acute kidney injury (AKI) are serious healthcare challenges that place a heavy burden on patient management and healthcare systems worldwide (Zarjou and Agarwal, 2011; Skube et al., 2018). Every year, millions of people develop sepsis, leading to increased mortality rates and healthcare costs as a result of longer hospital stays and increased need for intensive care (Souza et al., 2021). Current treatment strategies for sepsis-associated AKI (SA-AKI) include antibiotic therapy, fluid resuscitation, and supportive care (Kellum et al., 2019; Montomoli et al., 2019; Reddy et al., 2023). However, these approaches are often limited by the difficulty of early detection and accurate prediction of patient prognosis. The complex biological interactions between sepsis and AKI highlight the urgency of further research to unravel the underlying mechanisms and identify novel biomarkers that can aid in early diagnosis and timely intervention. The purpose of this study was to explore the relationship between sepsis and AKI in order to obtain new insights to improve the early diagnosis and treatment of patients with SA-AKI.

Previous studies indicate a biological link between sepsis and AKI, suggesting sepsis may trigger AKI development (Martensson et al., 2010; Liu et al., 2024). Conversely, AKI can exacerbate the clinical severity of sepsis (Vodovar et al., 2021). Determining the time-point of onset of AKI in sepsis is difficult (Murugan et al., 2010; Wen et al., 2020). To enhance early diagnosis and treatment of SA-AKI, a deeper understanding of the genes and mechanisms linking these conditions is vital, and biomarkers play an important role in this process (Derive and Gibot, 2011; Odum et al., 2022). Traditional kidney function markers like serum creatinine and urine output are essential for diagnosing AKI. Their limitations are becoming apparent, especially in patients with sepsis, where changes in kidney function tend to lag behind the AKI. This phenomenon has prompted clinicians to search for new biomarkers in order to recognize AKI early and improve the prognosis. Binding of tissue inhibitory factor-2 (TIMP-2) and insulin-like growth factor binding protein 7 (IGFBP7) in urine has been identified as a useful biomarkers for predicting SA-AKI (Su et al., 2017; Jiang and Zheng, 2022). In addition, the use of novel biomarkers such as kidney injury molecule-1 (KIM-1) and neutrophil gelatinase-associated lipid carrier (NGAL) provides more options for early diagnosis (Ersavas et al., 2016; Ucakturk et al., 2016; Laurentino et al., 2019). However, the diagnostic potential and immunologic significance of predicting AKI using sepsis genes remains largely unexplored.

Therefore, we chose to use bioinformatics methods and multiple machine learning strategies to identify key biomarkers associated with SA-AKI, to elucidate the intrinsic mechanisms and linkages between these key biomarkers, to establish an early diagnostic model, and to provide new perspectives for the early identification of SA-AKI.

## 2 Methods

### 2.1 Data collection and consolidation

In this study, we obtained relevant datasets from the National Center for Biotechnology Information (NCBI) Gene Expression Omnibus (GEO) database (https://www.ncbi.nlm.nih.gov/geo/) using the keywords “sepsis” and “acute kidney injury.” We used two sepsis datasets (GSE57065, GSE28750) and three AKI datasets (GSE30718, GSE139061, GSE67401). GSE57065 contained data on 25 control and 82 sepsis samples; GSE28750 contained data on 20 control and 21 sepsis samples; GSE30718 contained data on 8 control and 28 AKI samples; and GSE139061 contained data on 9 control and 39 AKI samples. GSE67401 contained data on 58 control and 53 AKI samples (Table 1). Two sepsis datasets (GSE57065 and GSE28750) and two AKI datasets (GSE30718 and GSE139061) were merged as training sets. The “ComBat” function within the “sva package” of the R programming language was used to eradicate the batch effect from the data (Leek et al., 2012). The Institutional Review Board of the Third People’s Hospital, which is associated with Fujian University of Traditional Chinese Medicine, exempted the necessity for ethical approval. This exemption was granted due to the fact that the data utilized is readily accessible to the public via the GEO database. Consequently, participants, along with their legal guardians or next of kin, were not mandated to furnish written informed consent for their involvement in this study, in accordance with relevant national regulations and institutional protocols.

**TABLE 1:** Basic information on the GEO dataset.

Dataset	Disease	Sample	Platform	Attribute
GSE57065	Sepsis	82 patients with sepsis and 25 controls	GPL570	Training set
GSE28750	Sepsis	21 patients with sepsis and 20 control	GPL570	Training set
GSE30718	AKI	28 patients with AKI and 8 controls	GPL570	Training set
GSE139061	AKI	39 patients with AKI and 9 controls	GPL20301	Training set
GSE67401	AKI	53 patients with AKI and 58 controls	GPL9115	Validation set

AKI: acute kidney injury.

### 2.2 Identification of differentially expressed genes (DEGs)

We used the R package “limma” to identify differentially expressed genes (DEGs) in the sepsis and AKI datasets (Ritchie et al., 2015). The established thresholds were |log2 fold change (FC)| > 1 and *P* < 0.05. To illustrate the findings, volcano plots and heat maps were created using the “ggplot2” and “pheatmap” packages in R. We constructed a Venn diagram to show the overlap between the DEGs associated with sepsis and those associated with AKI.

### 2.3 Enrichment analysis of intersecting genes

We constructed a protein-protein interaction (PPI) network using the GeneMANIA (http://genemania.org/) website to show the interrelationships between crossover genes. For genomic functional enrichment analysis, we used the Gene Ontology (GO) and Kyoto Encyclopedia of the Genome (KEGG) with the R “org.Hs.e.g.,.db” and “clusterProfiler” packages (*P* < 0.05).

### 2.4 Machine-learning algorithms

We evaluated the diagnostic accuracy of 113 combinations of 12 distinct machine-learning algorithms (Supplementary Table S1). We used the AKI de-batch effect datasets (GSE30718 and GSE139061) as the training set and an external dataset (GSE67401) as the validation set. During the model training phase, we evaluated the performance of a variety of machine-learning algorithms, including Enet regression (λ = 0.1), Lasso regression (λ = 0.05), Ridge regression (λ = 1.0), SVM (C = 1.0, γ = 0.01), LDA, Gradient Boosting Machine (GBM, learning rate = 0.1, number of trees = 100), RF (number of trees = 200), XGBoost (XGB, learning rate = 0.01, number of trees = 150). The models were developed using the training set and the hyperparameters were refined by cross-validation (Supplementary Table S2). During the model evaluation phase, the area under the curve (AUC) of each model was computed using the validation set to evaluate its classification accuracy. The model with the highest AUC was chosen as the optimal model. Calibration curves were used to evaluate the reliability, whereas decision curve analysis (DCA) was used to assess the clinical applicability of each model. The performance of the models was assessed in different subgroups and compared with published models of key genes for sepsis AKI pathogenesis were compared (DeLong test, *P* < 0.05).

### 2.5 Analysis of immune infiltration

We performed immune infiltration analysis of sepsis and AKI data using the “CIBERSORT” package in R with LM22 as the reference data set (*P* < 0.05). The results were illustrated using box-plots. Furthermore, heatmaps were used to show the correlation between immune cells and DEGs.

### 2.6 Identification of potential drug candidates

We used the Enrich platform (https://maayanlab.cloud/Enrichr/) to identify drug candidates capable of targeting the underlying pathological mechanisms associated with sepsis and AKI.

### 2.7 Statistical analysis

Data were processed and analyzed using R software (version 4.2.2; The R Foundation for Statistical Computing, Vienna, Austria). Normality of continuous variables was assessed using the Shapiro-Wilk test, supplemented by visual inspection of histograms. Variables conforming to a normal distribution (Shapiro-Wilk *P* ≥ 0.05) were compared between groups using the independent samples t-test, whereas non-normally distributed variables (Shapiro-Wilk *P* < 0.05) were compared between groups using the Mann-Whitney U test. Statistical significance was defined as a two-tailed *P* value <0.05.

## 3 Results

### 3.1 Data collection and de-batching

**FIGURE 1:**
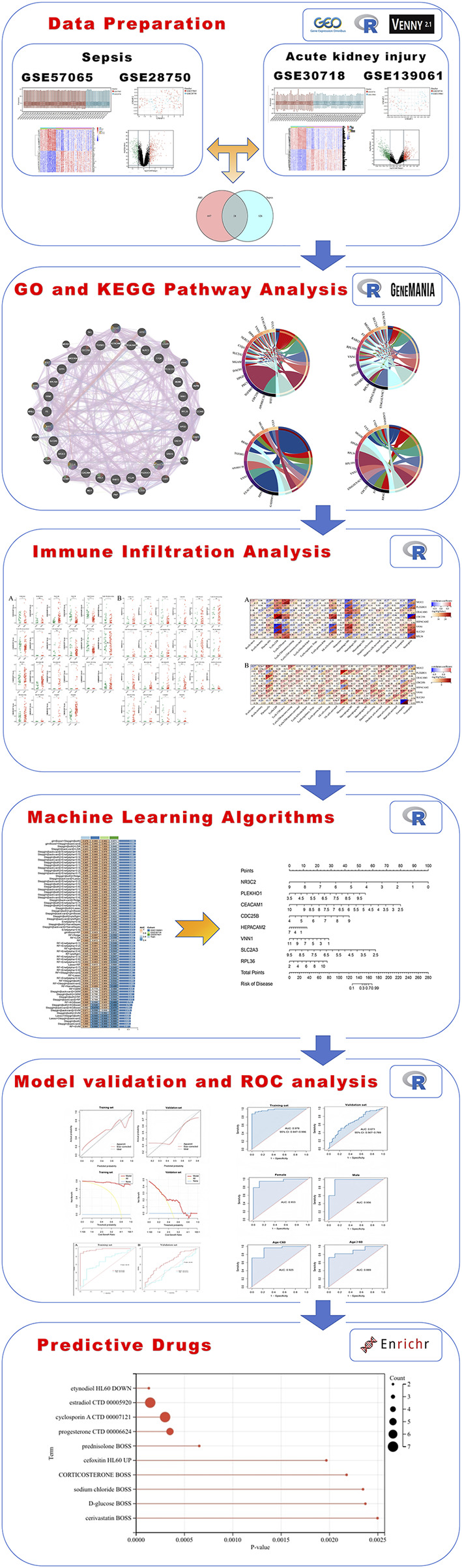
A full flowchart for the diagnosis of acute kidney injury based on sepsis-related genes. The six sections include data preparation, GO and KEGG pathway analysis, immune infiltration analysis, machine-learning algorithms, model validation and ROC analysis, and predictive drugs. GO, Gene Ontology; KEGG, Kyoto Encyclopedia of Genes and Genomes; ROC, receiver operating characteristic.

**FIGURE 2:**
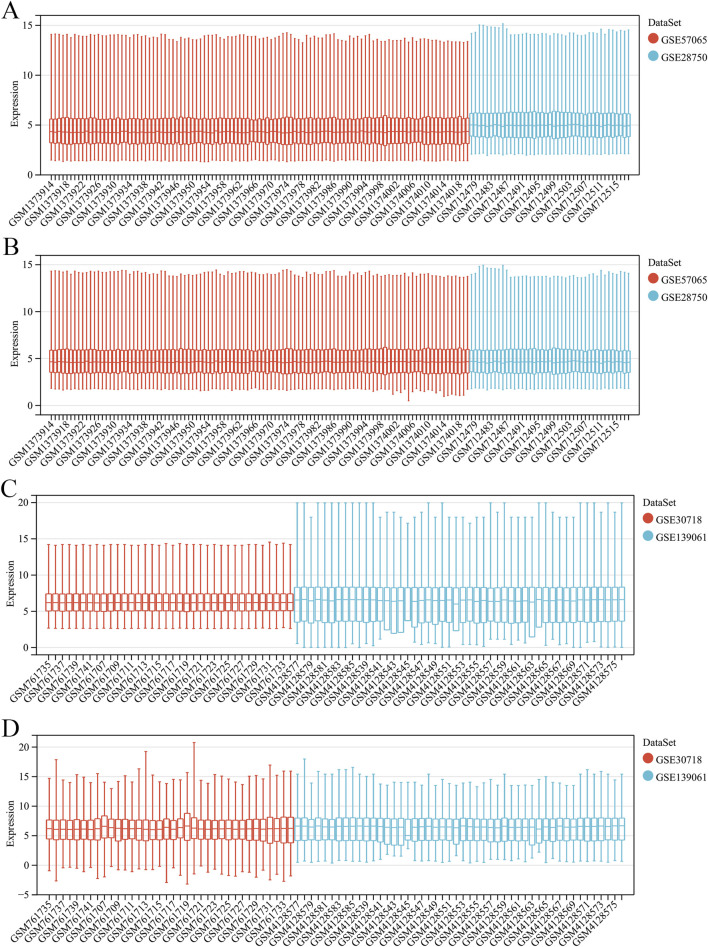
Merging and de-batching of sepsis dataset and acute kidney injury (AKI). **(A)** Boxplots of the sepsis dataset before removing batch effects. **(B)** Boxplots of the sepsis dataset after removing the batch effect. **(C)** Boxplots of the AKI dataset before removing batch effects. **(D)** Boxplots of the AKI dataset after removing the batch effect.

**FIGURE 3:**
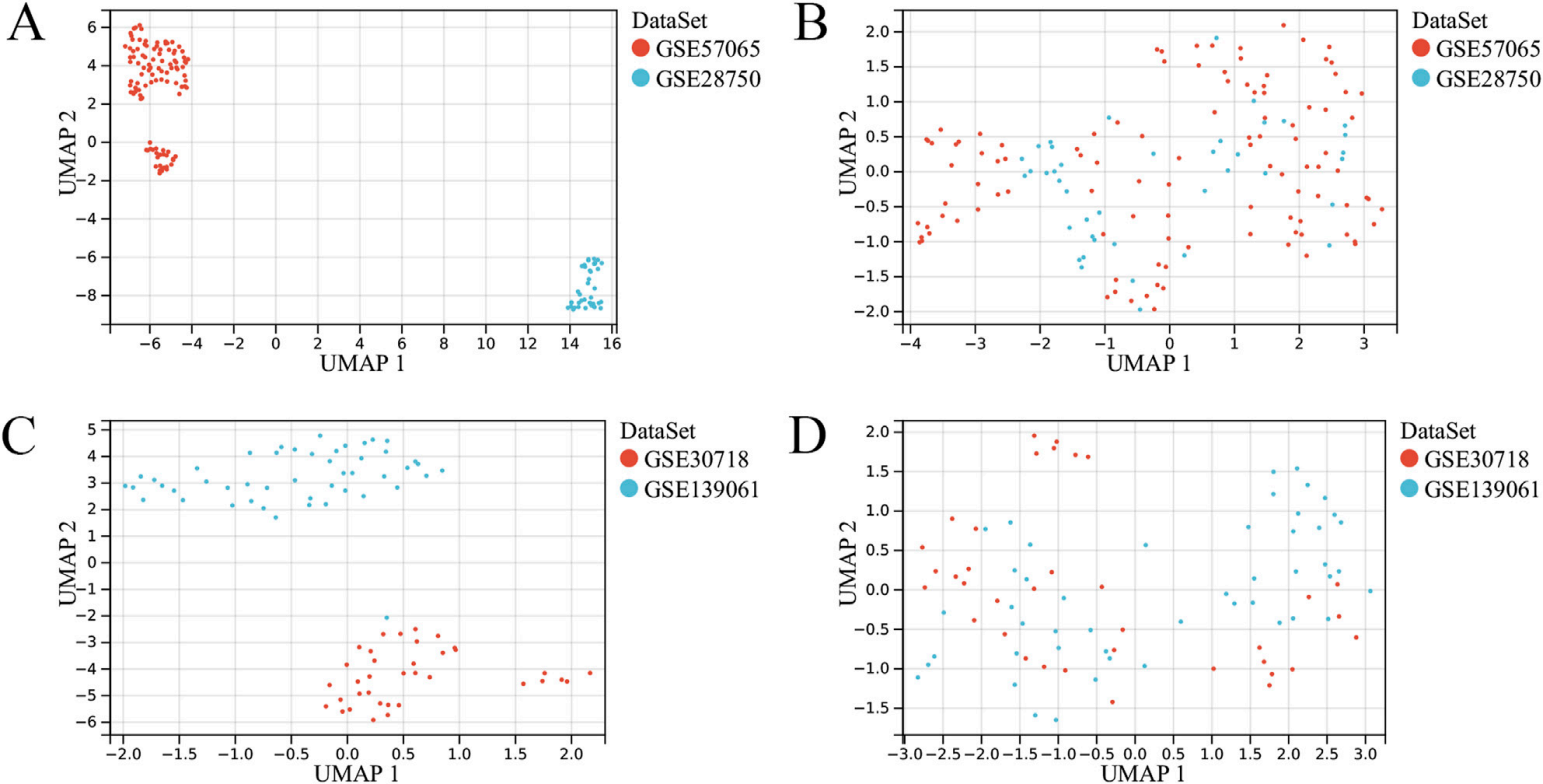
UMAP plots of the merged and de-batched sepsis dataset and acute kidney injury (AKI). **(A)** UMAP plot of the sepsis dataset before removing batch effects. **(B)** UMAP plot of the sepsis dataset after removing the batch effect. **(C)** UMAP plot of the AKI dataset before removing batch effects. **(D)** UMAP plot of the AKI dataset after removing the batch effect.

A summary of the study design is shown in Figure 1. After eliminating the batch effects from the sepsis datasets (GSE57065 and GSE28750), 103 cases of sepsis and 45 healthy controls were identified (Figures 2A,B). Similarly, after eliminating the batch effects from the AKI datasets (GSE30718 and GSE139061), 67 cases of AKI and 17 healthy controls were identified (Figures 2C,D). Boxplots effectively highlighted the variances observed prior to and subsequent to the removal of the batch effect. Furthermore, Uniform Manifold Approximation and Projection (UMAP) plots indicated that, prior to the batch-effect correction, the samples from each dataset exhibited a central clustering pattern. However, after correction, the samples displayed a more dispersed distribution, indicating a successful mitigation of the batch effect (Figures 3A–D).

### 3.2 Determination of sepsis and AKI DEGs separately

**FIGURE 4:**
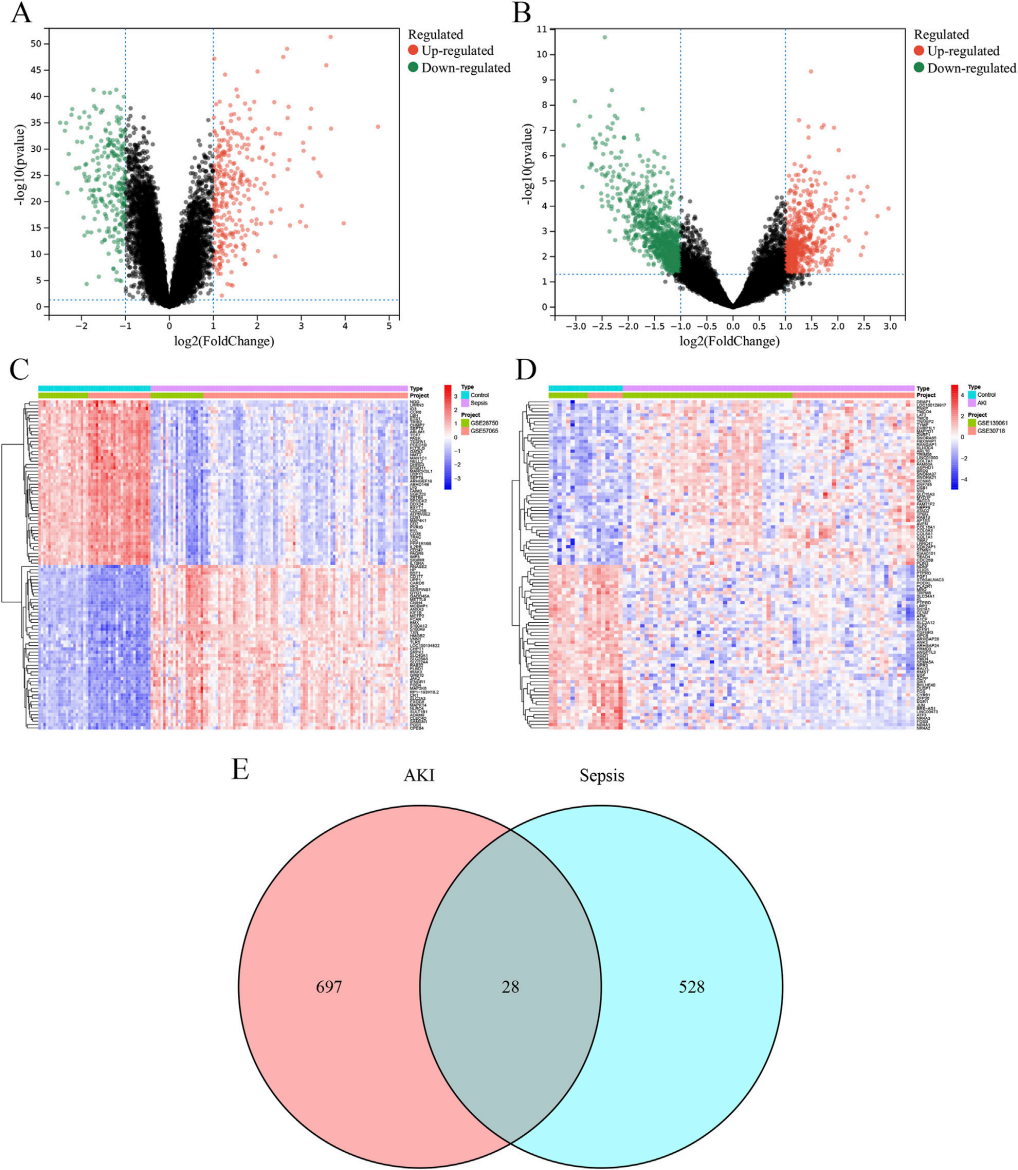
Identification of DEGs. **(A)** The volcano plot depicts the differential genes between the control and sepsis groups. **(B)** The volcano plot depicts the differential genes between the control and AKI groups. **(C)** Heatmap depicting the top 50 differential genes between the control and sepsis groups. **(D)** Heatmap depicting the top 50 differential genes between the control and AKI groups. **(E)** Venn diagram of the intersection of sepsis and AKI DEGs. AKI, acute kidney injury; DEG, differentially expressed gene.

A total of 309 upregulated DEGs and 247 downregulated DEGs were identified in the sepsis dataset (Figure 4A), and a total of 231 upregulated DEGs and 494 downregulated DEGs were identified in the AKI dataset (Figure 4B). Hierarchical clustering heatmaps of these DEGs revealed different expression patterns between the two groups (Figures 4C,D). Comparison of the two datasets revealed 28 overlapping co-morbid genes in sepsis and AKI (Figure 4E; Supplementary Table S3).

### 3.3 Functional enrichment analysis of sepsis and AKI intersection genes

**FIGURE 5:**
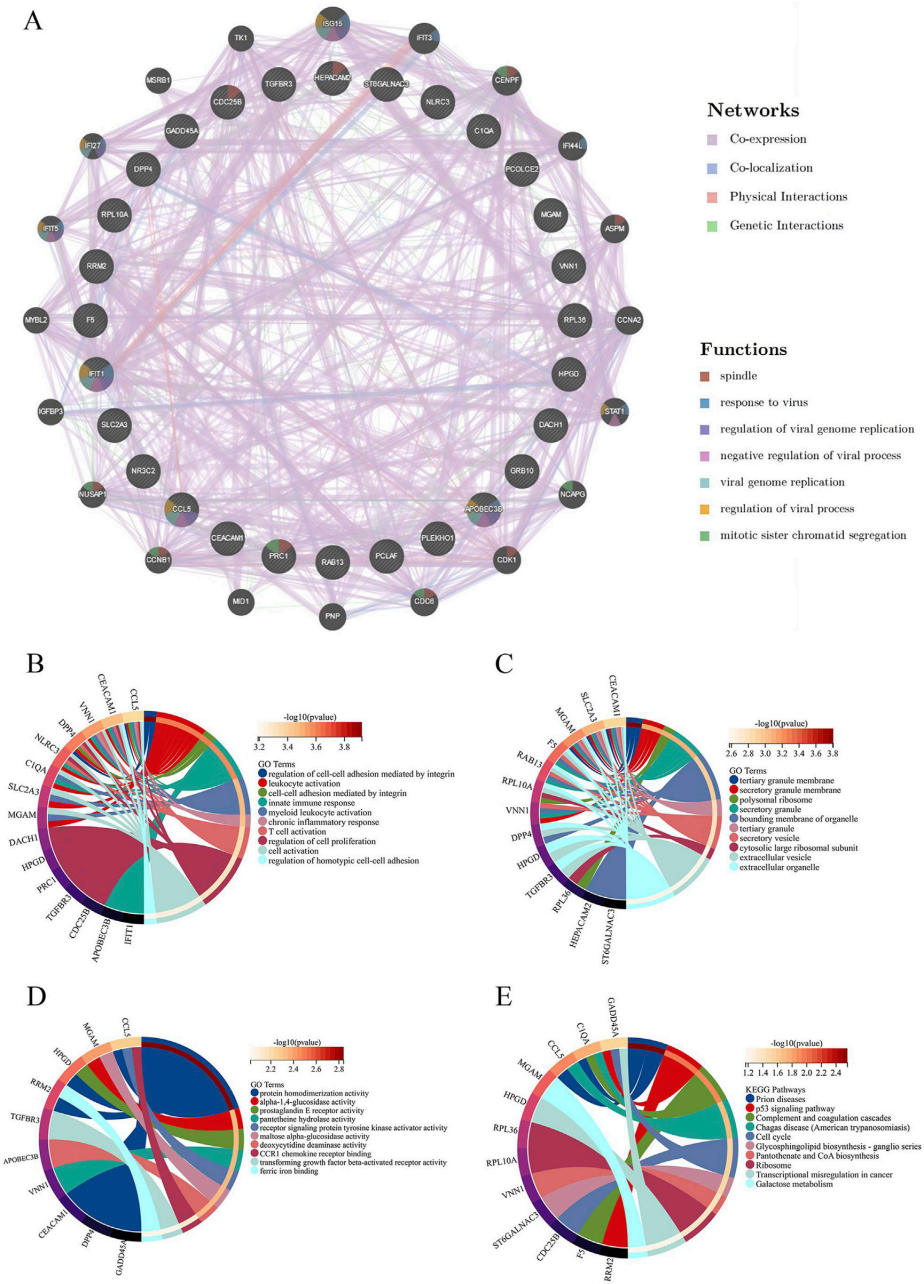
Protein-protein interaction (PPI) network analysis and enrichment analysis. **(A)** PPI analysis of intersecting differential genes. **(B)** Circle plot of GO enrichment analysis results for biological process. **(C)** Circle plot of GO enrichment analysis results for the cellular component. **(D)** Circle plot of GO enrichment analysis results for molecular function. **(E)** Circle plot of KEGG pathway enrichment analysis.GO, Gene Ontology; KEGG, Kyoto Encyclopedia of Genes and Genomes.

A PPI network diagram depicting the genes implicated in SA-AKI was constructed using the GeneMANIA database (Figure 5A). The biological processes (BP) identified through GO enrichment analysis (Supplementary Table S4) encompassed the regulation of cell-cell adhesion facilitated by integrins, leukocyte activation, cell-cell adhesion mediated by integrins, innate immune responses, and myeloid leukocyte activation (Figure 5B). The cellular components (CC) comprised the tertiary granule membrane, secretory granule membrane, polysomal ribosome, secretory granule, and the bounding membranes of organelles (Figure 5C). The molecular functions (MF) included protein homodimerization activity, alpha-1,4-glucosidase activity, prostaglandin E receptor activity, pantetheine hydrolase activity, and receptor signaling protein tyrosine kinase activator activity (Figure 5D). Additionally, the KEGG enrichment analysis revealed pathways associated with prion diseases, the p53 signaling pathway, complement and coagulation cascades, Chaga’s disease (American trypanosomiasis), and the cell cycle (Figure 5E; Supplementary Table S5).

### 3.4 Development of a diagnostic model for SA-AKI through machine learning

**FIGURE 6:**
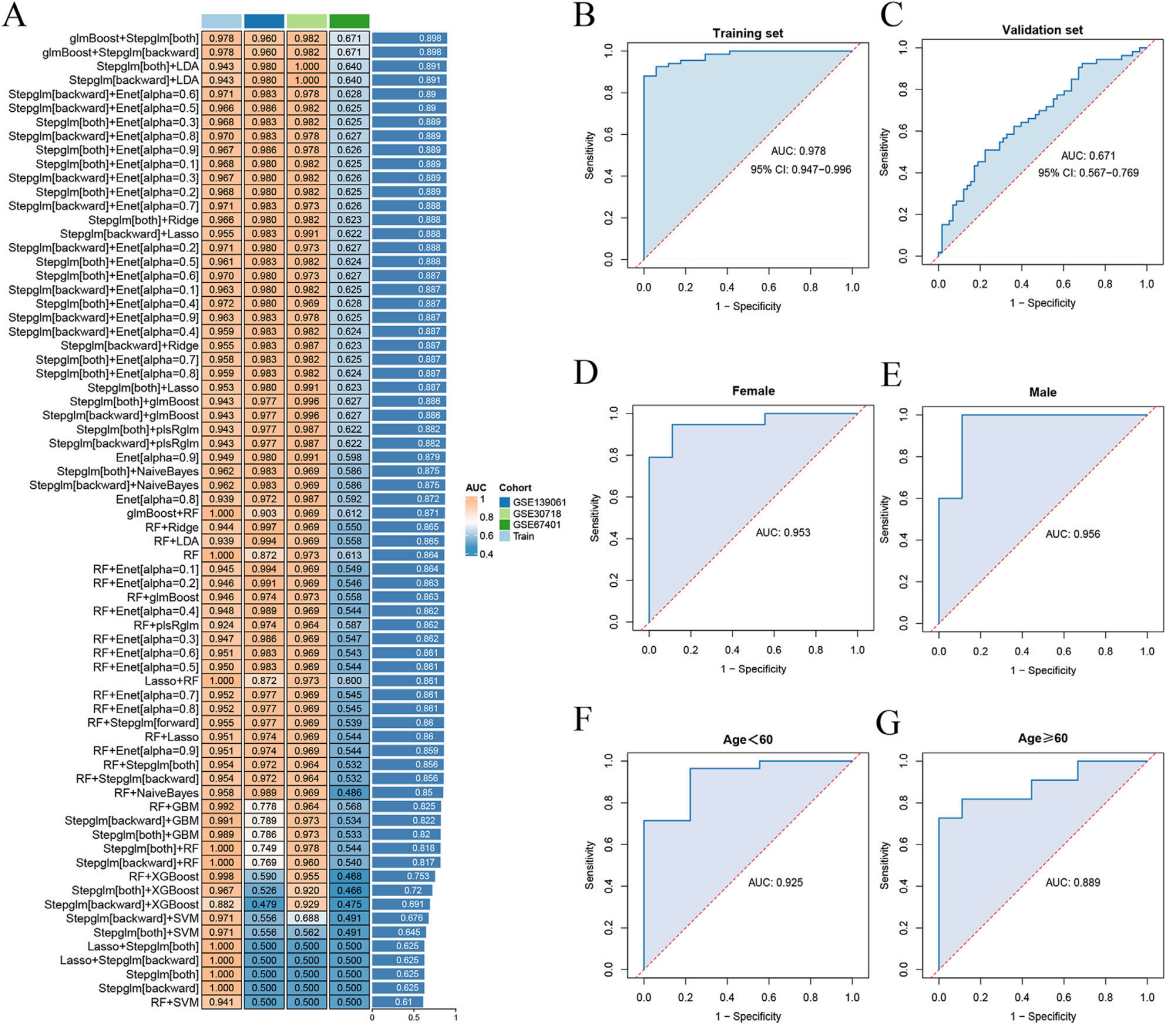
Diagnostic performance of machine-learning models. **(A)** 113 combinations of machine-learning algorithms with less than 12 genes were included. **(B)** ROC curves for training set. **(C)** ROC curves for validation set. **(D)** ROC curves for the female subgroup model. **(E)** ROC curves for the male subgroup model. **(F)** ROC curves for the age under 60 years subgroup model. **(G)** ROC curves for the age 60 years or older subgroup model. ROC, receiver operating characteristic.

**FIGURE 7:**
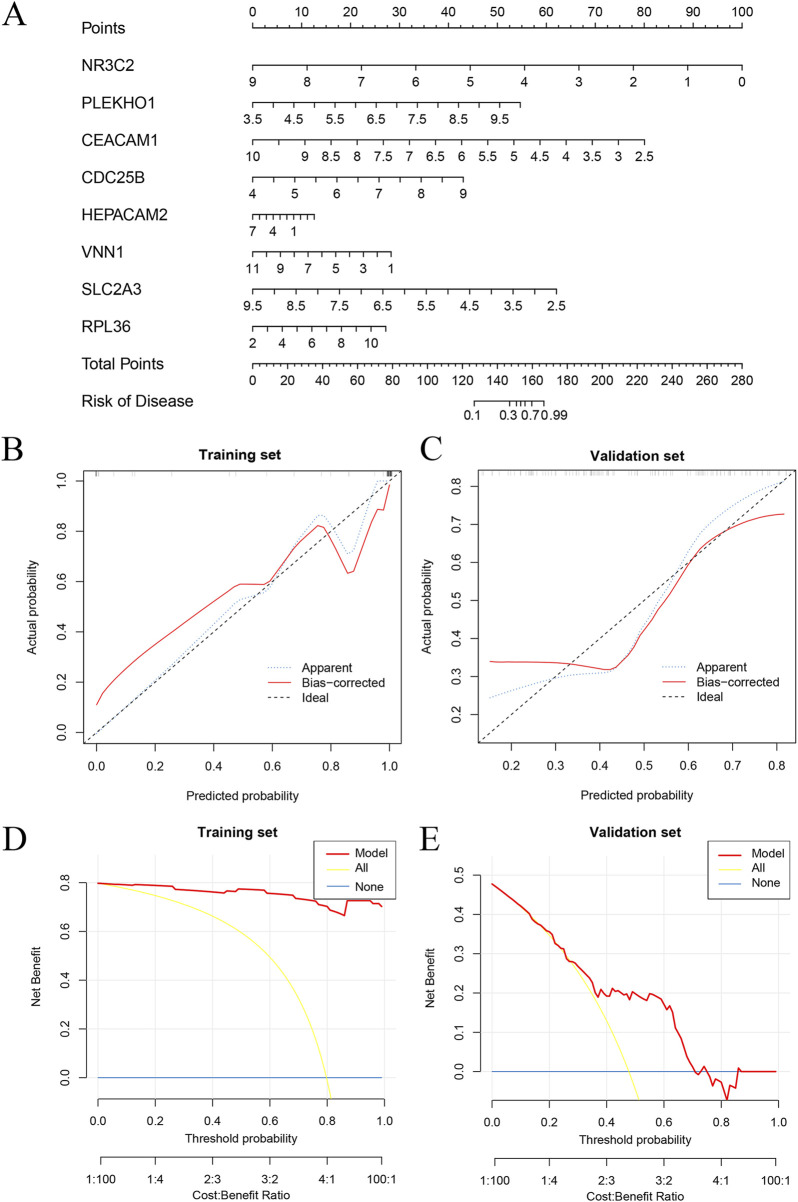
Nomogram and calibration of AKI diagnostic gene models. **(A)** Nomogram based on eight key genes. **(B)** Calibration curves for training set. **(C)** Calibration curves for validation set. **(D)** DCA curve for the training set. **(E)** DCA curve for the validation set. AKI, acute kidney injury; DCA, decision curve analysis.

Model training was conducted by amalgamating data from the AKI datasets to establish a training set, using 113 combinations of 12 distinct machine-learning algorithms for performance evaluation. The final synthesis of the glmBoost and Stepglm [both] algorithms yielded a model that demonstrated the best performance, with an AUC of 0.978. Validation against an external AKI dataset (GSE67401) indicated moderate performance with an AUC of 0.671 (Figures 6A–C; Supplementary Table S6). The model performed equally well for the subgroup under 60 years of age (AUC = 0.925) and the male subgroup (AUC = 0.956; Figures 6D–G). The algorithm identified eight key genes (*NR3C2, PLEKHO1, CEACAM1, CDC25B, HEPACAM2, VNN1, SLC2A3, RPL36*), which were subsequently used to develop a nomogram. The calibration and DCA curves demonstrated good agreement between the predicted and actual probabilities of occurrence, and good clinical utility of the model (Figures 7A–E).

### 3.5 Analysis of immune cell infiltration in sepsis and AKI

**FIGURE 8:**
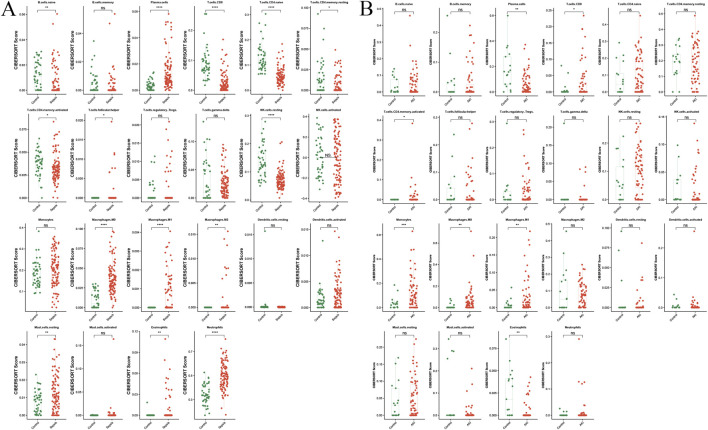
Immune infiltration analysis of sepsis and AKI. **(A)** Split-plane visualization of the CIBERSORT score of immune cells in the control and sepsis groups, with each facet representing a different immune cell subtype. **(B)** Visualization of the CIBERSORT score of immune cells in the control and AKI groups, with each facet representing a different immune cell subtype. *P < 0.05, **P < 0.01, ***P < 0.001, AKI, acute kidney injury.

Immune infiltration analysis showed distinct infiltration levels in the sepsis group compared with the control group, which included increased plasma cells, activated natural killer (NK) cells, M0 macrophages, eosinophils, CD8 T-cells, M1 macrophages, M2 macrophages, naive B cells, resting memory CD4 T-cells, naive CD4 T-cells, activated memory CD4 T-cells, gamma delta T cells, activated dendritic cells, neutrophils, and memory B cells in the sepsis group (Figure 8A). Similarly, differences in immune cell infiltration were observed between the AKI and control groups, including differences in plasma cells, CD8 T-cells, activated memory CD4 T-cells, monocytes, M0 macrophages, M1 macrophages, and eosinophils (Figure 8B).

**FIGURE 9:**
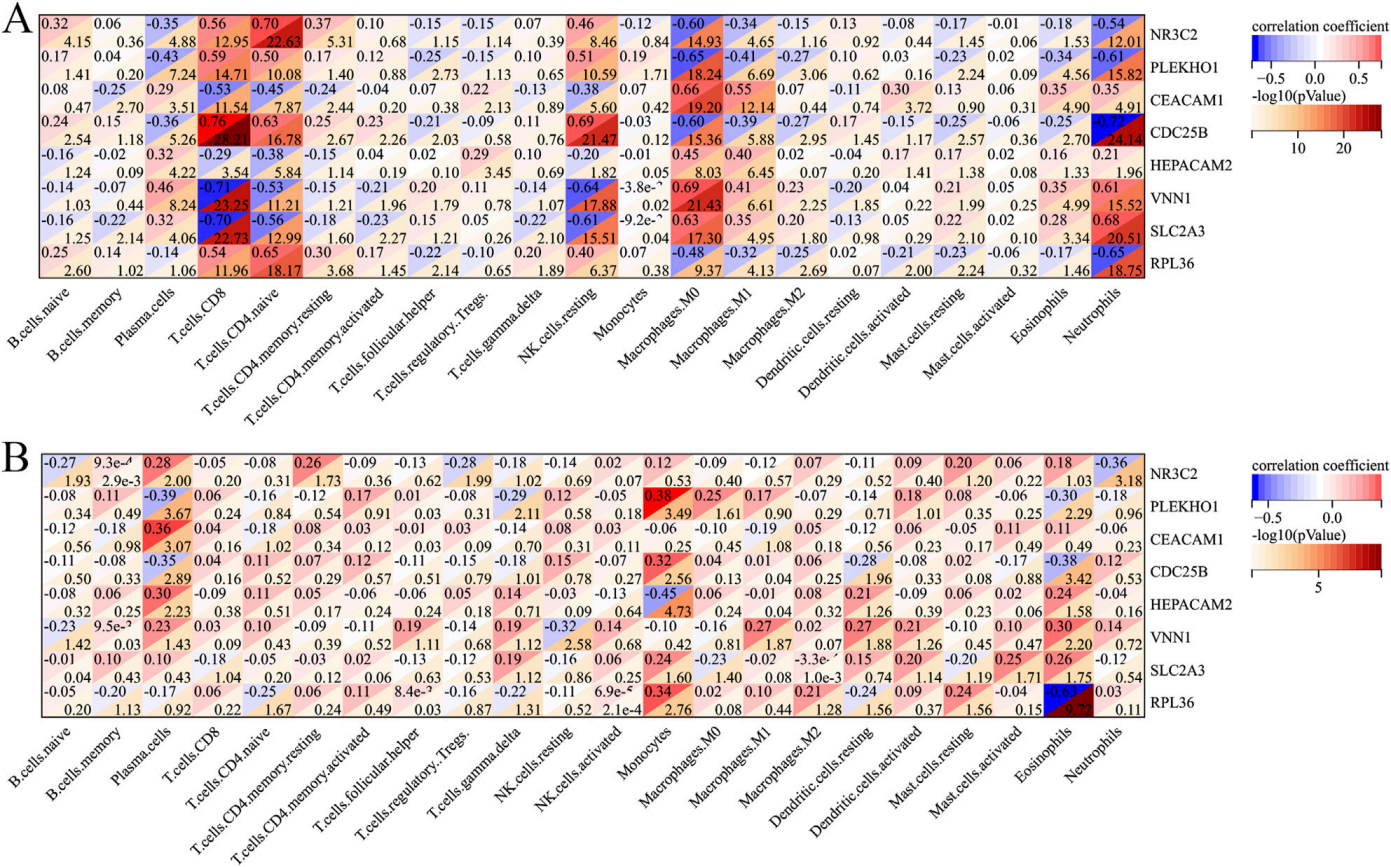
Heatmap of the correlation of each immune cell with the model genes. **(A)** Heatmap of the correlation of each immune cell type with sepsis-related genes. **(B)** Heatmap of the correlation of each immune cell type with AKI-related genes. AKI, acute kidney injury.

Figure 9 shows the correlation between the immune cell counts and the sepsis and AKI model genes. Table 2 shows immune cell indicators were highly correlated (absolute magnitude of the correlation coefficient >0.60) with model genes in each data set.

**TABLE 2:** Correlation of immune cells with model genes.

Data set	Gene name	Immune cell	Correlation coefficient
Sepsis	NR3C2	T.cells.CD4.naive	+0.70
Sepsis	NR3C2	Macrophages.M0	−0.60
Sepsis	PLEKHO1	Neutrophils	−0.61
Sepsis	PLEKHO1	Macrophages.M0	−0.65
Sepsis	CDC25B	Neutrophils	−0.72
Sepsis	CDC25B	Macrophages.M0	−0.60
Sepsis	CDC25B	NK cells.resting	+0.69
Sepsis	CDC25B	T.cells.CD4.naive	+0.63
Sepsis	VNN1	Neutrophils	+0.61
Sepsis	VNN1	Macrophages.M0	+0.69
Sepsis	VNN1	NK.cells.resting	−0.64
Sepsis	VNN1	T.cells.CD8	−0.71
Sepsis	SLC2A3	Neutrophils	+0.68
Sepsis	SLC2A3	Macrophages.M0	+0.63
Sepsis	SLC2A3	NK.cells.resting	−0.61
Sepsis	SLC2A3	T.cells.CD8	−0.70
Sepsis	RPL36	Neutrophils	−0.65
Sepsis	RPL36	T.cells.CD4.naive	+0.65
AKI	RPL36	Eosinophils	−0.63

AKI: acute kidney injury.

### 3.6 Comparison of AKI diagnostic models

**FIGURE 10:**
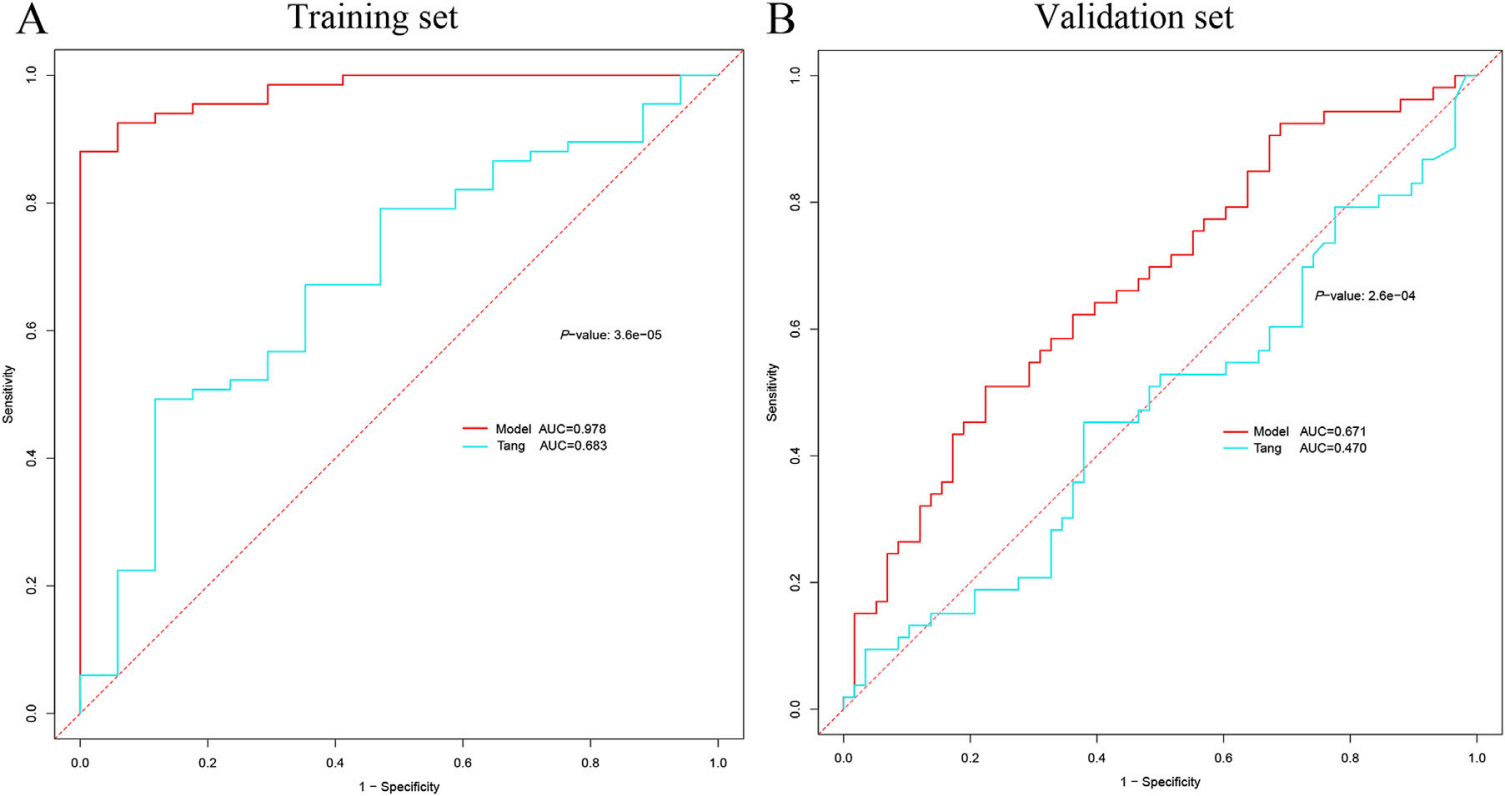
Comparison of AKI diagnostic genes. **(A)** Comparison of ROC curves between the current model in the training cohort and the model of Tang et al. **(B)** Comparison of ROC curves between the current model in the validation set and the model of Tang et al. AKI, acute kidney injury; ROC, receiver operating characteristic.

In order to comprehensively compare the performance of our model with other models, we compared the involvement of *VMP1, SLPI, PTX3, TIMP1, OLFM4, LCN2*, and *S100A9* genes in the development of AKI in patients with sepsis as described in the paper by Tang et al. (2021). The *VMP1* gene was not found in the validation cohort and was excluded. In the training cohort, our model achieved an AUC of 0.978 (95% CI: 0.952–0.995) vs 0.683 (95% CI: 0.538–0.828) for the Tang model (DeLong test, *P* = 3.6e−05). The AUC of our model was also higher than that of the Tang model using the validation cohort with AUCs of 0.671 (95% CI: 0.575–0.767) and 0.470 (95% CI: 0.362–0.583) for our model and the Tang model, respectively (DeLong test, *P* = 2.6e−04) (Figures 10A,B).

### 3.7 Drug predictions

**FIGURE 11:**
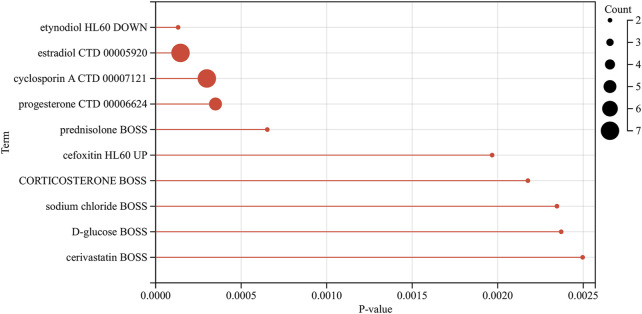
Bar graph of drug prediction for the intersection of sepsis and AKI genes.

We analyzed the model genes using the Drug Signatures Database (DSigDB) and the Enrich platform to identify potential pharmacological agents that could be used to treat AKI in patients with sepsis. The leading ten drug candidates identified were etynodiol (HL60 DOWN), estradiol (CTD 00005920), cyclosporin A (CTD 00007121), progesterone (CTD 00006624), prednisolone (BOSS), cefoxitin (HL60 UP), corticosteroid (BOSS), sodium chloride (BOSS), D-glucose (BOSS), and cerivastatin (BOSS) (Figure 11).

## 4 Discussion

The integration of bioinformatics with machine learning is becoming progressively significant in the realm of disease diagnosis. With the rapid growth of biomedical data, traditional analytical methods are no longer adequate for a deeper understanding of complex biological systems (Qian et al., 2024). In recent years, many studies (Pillai et al., 2022; Alsaeedi et al., 2024) have shown that the combination of machine-learning methods with bioinformatics approaches can enhance the training and validation process, identify the best interpretable features, and facilitate deeper investigation of features and models. Therefore, this study developed a model for diagnosing AKI based on a database of patients with sepsis, using a combination of machine learning and bioinformatics. The integration of these methods holds promise for improving the early detection and clinical treatment of SA-AKI.

This study highlights the intricate relationship between sepsis and AKI by identifying DEGs. 28 genes were identified as shared differential genes. These 28 overlapping genes may lead to common molecular pathways in the pathogenesis of both sepsis and AKI. This is consistent with previous findings that emphasize the importance of changes in gene expression for understanding disease mechanisms (Goggi et al., 2020).

Through KEGG enrichment analysis, we identified several key pathways that reveal the intrinsic mechanisms of interdependence between sepsis and AKI. The p53 signaling pathway is crucial for the cellular response to stress, the induction of apoptosis, and the regulation of the cell cycle. Its dysregulation is associated with a variety of pathologic conditions, including sepsis (Hotchkiss et al., 2000). Activation of p53 may lead to kidney cell apoptosis and exacerbate AKI during sepsis (Lee et al., 2024; Lv et al., 2024). The complement and coagulation cascades represent an additional pathway of enrichment, aligning with findings from prior research (Lin et al., 2024; Wei et al., 2024). The complement system is activated during sepsis, promoting an inflammatory response, and potentially leading to kidney microvascular injury. Activation of this pathway can lead to the formation of membrane attack complexes, which can damage kidney endothelial cells and further exacerbate AKI. The analysis revealed that the regulation of cell-cell adhesion mediated by integrins is an important biological process. Integrins are essential for maintaining the integrity of the endothelial barrier (Ayloo et al., 2022), and their dysregulation leads to increased vascular permeability and tissue edema (Infanger et al., 2008), a common feature of sepsis and AKI. Disruption of cell adhesion mechanisms may promote leukocyte infiltration and exacerbate inflammation, leading to AKI (Grande et al., 2010).

Immune cell analysis showed significant differences between the sepsis and control groups, highlighting the roles of specific immune cells in sepsis and AKI. The main cell types involved included activated NK cells, M0 macrophages, and CD8 T-cells. Activated NK cells are crucial for the innate immune response, recognizing and eliminating infected or malignant cells (Wu and Lanier, 2003; Brandstadter and Yang, 2011). In sepsis, the presence of NK cells is associated with an improved immune response to infection. NK cells produce pro-inflammatory cytokines, contributing to the systemic inflammatory response in sepsis (Jensen et al., 2021). The infiltration of activated NK cells in sepsis suggests that this may be a compensatory mechanism to restore homeostasis, but overactivation of NK cells can lead to tissue damage, including AKI. M0 macrophages maintain tissue homeostasis and initiate immune responses (Chen et al., 2017). The infiltration of M0 macrophages in sepsis suggests potential transformation into a pro-inflammatory phenotype, which is crucial in early sepsis. M0 macrophages can differentiate into inflammatory M1 or tissue-repairing M2 macrophages (Enderlin Vaz Da Silva et al., 2014; Isali et al., 2022). The balance between these phenotypes is critical in determining the outcome of sepsis and AKI. CD8 T-cells, which are cytotoxic, are key to the adaptive immune response (Jung et al., 2021). CD8 T-cells exacerbate inflammation and lead to organ dysfunction (Martin et al., 2018). In this study, the activation of CD8 T-cells was correlated with poorer prognosis of sepsis. The pattern of immune cell infiltration highlights the complex relationship between the immune system and sepsis and AKI. The study of activated NK cells, M0 macrophages, and CD8 T-cells could shed light on the pathogenesis of AKI in patients with sepsis and provide further insights into the relationship between these two conditions.

Early diagnosis of SA-AKI is challenging for clinicians (Zhang et al., 2024). Delayed diagnosis not only leads to deterioration of the disease, but also sometimes leads to the transformation of SA-AKI into chronic kidney disease in some patients (Heung and Chawla, 2014), resulting in increased medical and social costs. Although the application of machine-learning techniques in this field shows good prospects, it still faces various challenges such as data quality, model interpretability, and clinical applicability. In a study based on machine-learning algorithms (Li et al., 2023; Li et al., 2024), it was shown that a model built using electronic medical record data enabled accurate prediction of SA-AKI in patients admitted to the intensive care unit. Our study differs from previous studies in that we focus on the overlap of genes between sepsis and AKI, which may be the key genes in the development of sepsis into AKI, and in this way build a more suitable model. Among 113 combinations of 12 machine-learning algorithms, the models were screened for number of genes <12. Of the models considered, the glmBoost and Stepglm [both] algorithms combined produced the highest AUC value, which suggests their potential utility in a clinical setting. This algorithm also reduces the bias caused by incomplete algorithm selection and subjective human selection (Liu et al., 2022; Qin et al., 2023). In terms of model effect validation, our model showed excellent diagnostic accuracy based on the high AUC in both the internal and external validation sets, especially in the age under 60 years and male patient subgroups, which showed stronger diagnostic performance. At the same time, our model achieved diagnostic performance superior to that of the previous diagnostic model identified by Tang et al. (2021). that influences the composition of genes important for the development of SA-AKI, both in the training and validation cohorts.

In our model, four genes (*NR3C2, CEACAM1, VNN1, and SLC2A3*) were linked to the development of AKI in patients with sepsis. *NR3C2* participates in corticosteroid signaling, a process that is essential for modulating the inflammatory response observed in sepsis (Wong et al., 2016). The upregulation of *NR3C2* in the sepsis cohort suggests a compensatory mechanism designed to counteract the inflammatory milieu, which may have implications for therapeutic interventions targeting this receptor. *CEACAM1* is a member of the carcinoembryonic antigen family and plays a crucial role in immune regulation and cell-cell interactions. It modulates T cell responses and promotes inflammatory regression (Skubitz, 2024). In our AKI cohort, downregulation of *CEACAM1* may reflect impaired immune responses in the context of sepsis, leading to the development of SA-AKI. Downregulation of *SLC2A3* expression in sepsis decreases glucose uptake by kidney cells, leading to insufficient intracellular energy metabolism. The proper physiological functioning of renal cells is contingent upon a sufficient energy supply, and insufficient energy impairs tubular reabsorption, glomerular filtration and other functions, and triggers oxidative stress, which damages the biomolecules of kidney cells, further aggravating the kidney injury, and promoting the development of AKI (Luo et al., 2023). *VNN1* is involved in processes such as lipid metabolism (Ucakturk et al., 2016). *VNN1* triggers senescence in renal tubular cells by stimulating *RB1* expression (Chen et al., 2022). In animal models, elevated *VNN1* expression correlates with the degree of AKI and inhibition of *VNN1* activity attenuates pathological changes in the kidney (Hosohata et al., 2011). *PLEKHO1* is involved in a variety of cellular functions, including cytoskeletal arrangement and cell signaling (Nie et al., 2013). In this study, the different expression levels of *PLEKHO1* highlight its potential role as a biomarker and therapeutic target for SA-AKI. Few studies have assessed the direct involvement of *CDC25B*, *HEPACAM2*, and *RPL36* in the progression of SA-AKI. Although these genes have important functions in intracellular signaling, cell cycle, intercellular interactions, and protein synthesis, their specific mechanisms of action in SA-AKI have not been fully clarified. Consequently, additional investigations into their particular roles and mechanisms of action are essential to convert these discoveries into clinical applications and to avert the onset of SA-AKI.

Finally, through drug analysis on the Enrich platform, we identified 10 drug candidates, and the identification of potential therapeutic agents, including cyclosporine A and estradiol, opens new avenues for targeted therapeutic strategies. Cyclosporin A is a potent immunosuppressant that acts primarily on T lymphocytes (Flores et al., 2019). The primary mechanism may involve reducing the harm to the kidney tissue caused by the inflammatory response by suppressing the excessive activation of immune cells. Estradiol has antioxidant properties. It may have a therapeutic effect on AKI by blocking the mitochondrial apoptotic pathway through its direct effect on organelles (Borras et al., 2010), helping to maintain the normal structure and function of kidney cells. These drug candidates warrant further investigation through clinical trials to evaluate their efficacy in SA-AKI.

This study has some limitations. First, the lack of wet-laboratory validation limits the ability to confirm the biological relevance of identified DEGs and their interactions. Although bioinformatics methods provide valuable insights, they cannot fully replace empirical data. Second, the longitudinal design of GSE57065 (with multiple time points per septic shock patient) may introduce intra-individual correlation bias, potentially inflating statistical significance for certain DEGs. Furthermore, while ComBat was applied to harmonize GSE57065 (septic shock) and GSE28750 (sepsis without shock), this batch correction method may inadvertently attenuate biologically meaningful time-dependent expression patterns. Third, despite the incorporation of multiple GEO datasets, the limited sample size could potentially restrict the broader applicability of the results obtained. Future studies should prioritize multi-omics validation in prospectively collected cohorts with standardized sampling time points while adopting advanced batch correction methods that better preserve temporal dynamics.

## 5 Conclusion

In conclusion, this study identified the key genes linking sepsis and AKI through comprehensive bioinformatics analysis. A model was developed for early AKI diagnosis in the context of sepsis using machine learning. These findings have the potential to advance the development of clinical diagnostic and co-treatment strategies, paving the way for improved prognosis of SA-AKI.

## Data Availability

The datasets presented in this study can be found in online repositories. The names of the repository/repositories and accession number(s) can be found below: https://www.ncbi.nlm.nih.gov/geo/, GSE57065 https://www.ncbi.nlm.nih.gov/geo/, GSE28750 https://www.ncbi.nlm.nih.gov/geo/, GSE30718 https://www.ncbi.nlm.nih.gov/geo/, GSE139061 https://www.ncbi.nlm.nih.gov/geo/, GSE67401.
