# Supplementary material for: Development and validation of a machine-learning-based model for identification of genes associated with sepsis-associated acute kidney injury
Source: Front Genet. 2025 Jul 22;16:1561331. doi: 10.3389/fgene.2025.1561331 (PMC12321556; doi:10.3389/fgene.2025.1561331)
Supplement: Supplementary file 1 [file Table1.docx]

Supplementary Material

# ****Supplementary Table 1. 113 algorithmic models****

| **No** | **Model** | **Full model name** | **No** | **Model** | **Full model name** |
| --- | --- | --- | --- | --- | --- |
| 1 | Lasso + Stepglm [both] | Lasso + Stepwise GLM [both] | 24 | Enet [alpha=0.9] | Elastic Net [alpha=0.9] |
| 2 | SVM | Support Vector Machine (SVM) | 25 | Lasso | Lasso Regression |
| 3 | glmBoost + SVM | glmBoost + Support Vector Machine | 26 | Enet [alpha=0.7] | Elastic Net [alpha=0.7] |
| 4 | Ridge | Ridge Regression | 27 | glmBoost + Enet [alpha=0.9] | glmBoost + Elastic Net [alpha=0.9] |
| 5 | Lasso + SVM | Lasso + Support Vector Machine | 28 | glmBoost + Lasso | glmBoost + Lasso |
| 6 | glmBoost + Ridge | glmBoost + Ridge Regression | 29 | Lasso + plsRglm | Lasso + Partial Least Squares Regression Generalized Linear Model (plsRglm) |
| 7 | Enet [alpha=0.1] | Elastic Net [alpha=0.1] | 30 | glmBoost + plsRglm | glmBoost + Partial Least Squares Regression Generalized Linear Model (plsRglm) |
| 8 | glmBoost + Enet [alpha=0.1] | glmBoost + Elastic Net [alpha=0.1] | 31 | glmBoost + Stepglm [forward] | glmBoost + Stepwise GLM [forward] |
| 9 | Enet [alpha=0.2] | Elastic Net [alpha=0.2] | 32 | Lasso + Stepglm [forward] | Lasso + Stepwise GLM [forward] |
| 10 | Enet [alpha=0.3] | Elastic Net [alpha=0.3] | 33 | RF + SVM | Random Forest + Support Vector Machine (RF + SVM) |
| 11 | glmBoost + Enet [alpha=0.3] | glmBoost + Elastic Net [alpha=0.3] | 34 | Stepglm [forward] | Stepwise GLM [forward] |
| 12 | glmBoost + Enet [alpha=0.2] | glmBoost + Elastic Net [alpha=0.2] | 35 | plsRglm | Partial Least Squares Regression Generalized Linear Model (plsRglm) |
| 13 | Enet [alpha=0.4] | Elastic Net [alpha=0.4] | 36 | RF + Ridge | Random Forest + Ridge Regression |
| 14 | glmBoost + Enet [alpha=0.4] | glmBoost + Elastic Net [alpha=0.4] | 37 | RF + Enet [alpha=0.1] | Random Forest + Elastic Net [alpha=0.1] |
| 15 | Lasso + glmBoost | Lasso + glmBoost | 38 | RF + plsRglm | Random Forest + Partial Least Squares Regression Generalized Linear Model (plsRglm) |
| 16 | Enet [alpha=0.5] | Elastic Net [alpha=0.5] | 39 | RF + Stepglm [forward] | Random Forest + Stepwise GLM [forward] |
| 17 | glmBoost | glmBoost | 40 | RF + Enet [alpha=0.2] | Random Forest + Elastic Net [alpha=0.2] |
| 18 | glmBoost + Enet [alpha=0.5] | glmBoost + Elastic Net [alpha=0.5] | 41 | RF + Enet [alpha=0.3] | Random Forest + Elastic Net [alpha=0.3] |
| 19 | Enet [alpha=0.6] | Elastic Net [alpha=0.6] | 42 | RF + Enet [alpha=0.6] | Random Forest + Elastic Net [alpha=0.6] |
| 20 | glmBoost + Enet [alpha=0.6] | glmBoost + Elastic Net [alpha=0.6] | 43 | RF + Lasso | Random Forest + Lasso |
| 21 | glmBoost + Enet [alpha=0.7] | glmBoost + Elastic Net [alpha=0.7] | 44 | RF + Enet [alpha=0.7] | Random Forest + Elastic Net [alpha=0.7] |
| 22 | glmBoost + Enet [alpha=0.8] | glmBoost + Elastic Net [alpha=0.8] | 45 | RF + Enet [alpha=0.5] | Random Forest + Elastic Net [alpha=0.5] |
| 23 | Enet [alpha=0.8] | Elastic Net [alpha=0.8] | 46 | RF + glmBoost | Random Forest + glmBoost |
| 47 | RF + Enet [alpha=0.9] | Random Forest + Elastic Net [alpha=0.9] | 72 | Stepglm [backward] + glmBoost | Stepwise GLM [backward] + glmBoost |
| 48 | RF + Enet [alpha=0.4] | Random Forest + Elastic Net [alpha=0.4] | 73 | Stepglm [both] + Enet [alpha=0.5] | Stepwise GLM [both] + Elastic Net [alpha=0.5] |
| 49 | RF + Enet [alpha=0.8] | Random Forest + Elastic Net [alpha=0.8] | 74 | Stepglm [backward] + Enet [alpha=0.5] | Stepwise GLM [backward] + Elastic Net [alpha=0.5] |
| 50 | RF + Stepglm [both] | Random Forest + Stepwise GLM [both] | 75 | glmBoost + RF | glmBoost + Random Forest |
| 51 | RF + Stepglm [backward] | Random Forest + Stepwise GLM [backward] | 76 | RF | Random Forest |
| 52 | Stepglm [both] + Ridge | Stepwise GLM [both] + Ridge Regression | 77 | Lasso + GBM | Lasso + Gradient Boosting Machine (GBM) |
| 53 | Stepglm [backward] + Ridge | Stepwise GLM [backward] + Ridge Regression | 78 | RF + GBM | Random Forest + Gradient Boosting Machine (GBM) |
| 54 | Stepglm [both] + plsRglm | Stepwise GLM [both] + Partial Least Squares Regression Generalized Linear Model (plsRglm) | 79 | GBM | Gradient Boosting Machine (GBM) |
| 55 | Stepglm [backward] + plsRglm | Stepwise GLM [backward] + Partial Least Squares Regression Generalized Linear Model (plsRglm) | 80 | Stepglm [both] + SVM | Stepwise GLM [both] + Support Vector Machine (SVM) |
| 56 | Stepglm [both] + Enet [alpha=0.9] | Stepwise GLM [both] + Elastic Net [alpha=0.9] | 81 | Stepglm [backward] + SVM | Stepwise GLM [backward] + Support Vector Machine (SVM) |
| 57 | Stepglm [backward] + Enet [alpha=0.9] | Stepwise GLM [backward] + Elastic Net [alpha=0.9] | 82 | Lasso + RF | Lasso + Random Forest |
| 58 | Stepglm [both] + Enet [alpha=0.1] | Stepwise GLM [both] + Elastic Net [alpha=0.1] | 83 | Stepglm [both] + GBM | Stepwise GLM [both] + Gradient Boosting Machine (GBM) |
| 59 | Stepglm [backward] + Enet [alpha=0.1] | Stepwise GLM [backward] + Elastic Net [alpha=0.1] | 84 | Stepglm [backward] + GBM | Stepwise GLM [backward] + Gradient Boosting Machine (GBM) |
| 60 | Stepglm [both] + Enet [alpha=0.8] | Stepwise GLM [both] + Elastic Net [alpha=0.8] | 85 | Stepglm [both] + RF | Stepwise GLM [both] + Random Forest |
| 61 | Stepglm [backward] + Enet [alpha=0.8] | Stepwise GLM [backward] + Elastic Net [alpha=0.8] | 86 | LDA | Linear Discriminant Analysis (LDA) |
| 62 | Stepglm [both] + Enet [alpha=0.2] | Stepwise GLM [both] + Elastic Net [alpha=0.2] | 87 | glmBoost + LDA | glmBoost + Linear Discriminant Analysis (LDA) |
| 63 | Stepglm [backward] + Enet [alpha=0.2] | Stepwise GLM [backward] + Elastic Net [alpha=0.2] | 88 | RF+LDA | Random Forest + Linear Discriminant Analysis (LDA) |
| 64 | Stepglm [both] + Lasso | Stepwise GLM [both] + Lasso | 89 | Stepglm [both]+LDA | Stepwise GLM [both] + Linear Discriminant Analysis (LDA) |
| 65 | Stepglm [backward] + Lasso | Stepwise GLM [backward] + Lasso | 90 | Stepglm [backward]+LDA | Stepwise GLM [backward] + Linear Discriminant Analysis (LDA) |
| 66 | Stepglm [both] + Enet [alpha=0.6] | Stepwise GLM [both] + Elastic Net [alpha=0.6] | 91 | Lasso + LDA | Lasso + Linear Discriminant Analysis (LDA) |
| 67 | Stepglm [backward] + Enet [alpha=0.6] | Stepwise GLM [backward] + Elastic Net [alpha=0.6] | 92 | Stepglm [backward] + RF | Stepwise GLM [backward] + Random Forest |
| 68 | glmBoost + GBM | glmBoost + Gradient Boosting Machine (GBM) | 93 | XGBoost | eXtreme Gradient Boosting (XGBoost) |
| 69 | Stepglm [both] + Enet [alpha=0.7] | Stepwise GLM [both] + Elastic Net [alpha=0.7] | 94 | Lasso+XGBoost | Lasso + eXtreme Gradient Boosting (XGBoost) |
| 70 | Stepglm [backward] + Enet [alpha=0.7] | Stepwise GLM [backward] + Elastic Net [alpha=0.7] | 95 | glmBoost+XGBoost | glmBoost + eXtreme Gradient Boosting (XGBoost) |
| 71 | Lasso + Stepglm [backward] | Lasso + Stepwise GLM [backward] | 96 | RF+XGBoost | Random Forest + eXtreme Gradient Boosting (XGBoost) |
| 97 | Stepglm [both] | Stepwise GLM [both] | 106 | Stepglm[both]+XGBoost | Stepwise GLM [both] + eXtreme Gradient Boosting (XGBoost) |
| 98 | Stepglm [backward] | Stepwise GLM [backward] | 107 | Stepglm[backward]+XGBoost | Stepwise GLM [backward] + eXtreme Gradient Boosting (XGBoost) |
| 99 | glmBoost + Stepglm [both] | glmBoost + Stepwise GLM [both] | 108 | NaiveBayes | Naive Bayes |
| 100 | glmBoost + Stepglm [backward] | glmBoost + Stepwise GLM [backward] | 109 | Lasso+NaiveBayes | Lasso + Naive Bayes |
| 101 | Stepglm [both] + Enet [alpha=0.4] | Stepwise GLM [both] + Elastic Net [alpha=0.4] | 110 | glmBoost+NaiveBayes | glmBoost + Naive Bayes |
| 102 | Stepglm [backward] + Enet [alpha=0.4] | Stepwise GLM [backward] + Elastic Net [alpha=0.4] | 111 | RF+NaiveBayes | Random Forest + Naive Bayes |
| 103 | Stepglm [both] + Enet [alpha=0.3] | Stepwise GLM [both] + Elastic Net [alpha=0.3] | 112 | Stepglm[both]+NaiveBayes | Stepwise GLM [both] + Naive Bayes |
| 104 | Stepglm [backward] + Enet [alpha=0.3] | Stepwise GLM [backward] + Elastic Net [alpha=0.3] | 113 | Stepglm[backward]+NaiveBayes | Stepwise GLM [backward] + Naive Bayes |
| 105 | Stepglm [both] + glmBoost | Stepwise GLM [both] + glmBoost |  |  |  |

# ****Supplementary Table 2. Machine Learning Model Hyperparameter Optimization Strategies****

| **Model** | **Hyperparameter** | **Range/Candidate Values** | **Optimization Strategy** | **Reference** |
| --- | --- | --- | --- | --- |
| **Elastic Net** | α (L1/L2 mixing ratio) | [0, 0.2, 0.4, 0.6, 0.8, 1] | Grid search + 10-fold cross-validation | Zou et al.,2005 |
|  | λ (regularization strength) | Auto-computed (100 values) | Minimize binomial deviance via | Friedman et al., 2010 |
| **Lasso/Ridge** | λ (regularization strength) | Auto-computed (100 values) | Minimize binomial deviance via | Tibshirani, 1996 |
| **SVM** | C (cost) | [0.1, 1, 10] | Grid search + 5-fold cross-validation | Cortes et al., 1995 |
|  | γ (kernel width) | Adaptive calculation | Formula: γ = 1/(n_features · var(X)) | Chapelle et al., 2002 |
| **GBM** | N trees (number of trees) | Dynamic (max=10,000) | Early stopping (10-fold CV, patience=50) | Friedman, 2001 |
|  | Interaction depth | 3 (fixed) | Literature-based recommendation | Ridgeway, 2007 |
|  | shrinkage (learning rate) | 0.001 (fixed) | Ensure stable convergence | - |
| **XGBoost** | nround (iterations) | Dynamic (max=10) | 5-fold cross-validation | Chen et al., 2016 |
|  | Max depth (tree depth) | 2 (fixed) | Pre-experimental validation | - |
|  | eta (learning rate) | 1 (fixed) | Benchmark testing results | - |
| **Random Forest** | N tree (number of trees) | 1,000 (fixed) | Empirical balance of efficiency/stability | Breiman, 2001 |
|  | Node size (min node size) | 5 (fixed) | Limit tree complexity | - |
| **glmBoost** | M stop (iterations) | Dynamic (max=40) | Cross-validation (cvrisk) | Bühlmann et al., 2007 |
| **plsRglm** | nt (number of components) | 10 (fixed) | Pre-experimental cv.plsRglm validation | Bastien et al., 2005 |
| **Naive Bayes** | None | - | Probability density estimation | Rish, 2001 |

**Reference**

Zou, H., and Hastie, T. (2005). Regularization and variable selection via the elastic net. J. R. Stat. Soc. B 67, 301-320.

Friedman, J., Hastie, T., and Tibshirani, R. (2010). Regularization paths for generalized linear models via coordinate descent. J. Stat. Softw. 33, 1-22.

Tibshirani, R. (1996). Regression shrinkage and selection via the lasso. J. R. Stat. Soc. B 58, 267-288.

Hoerl, A.E., and Kennard, R.W. (1970). Ridge regression: Biased estimation for nonorthogonal problems. Technometrics 12, 55-67.

Cortes, C., and Vapnik, V. (1995). Support-vector networks. Mach. Learn. 20, 273-297.

Chapelle, O., Vapnik, V., Bousquet, O., and Mukherjee, S. (2002). Choosing multiple parameters for support vector machines. Mach. Learn. 46, 131-159.

Friedman, J.H. (2001). Greedy function approximation: A gradient boosting machine. Ann. Stat. 29, 1189-1232.

Ridgeway, G. (2007). Generalized boosted models: A guide to the gbm package. R Package Vignette 1-12.

Chen, T., and Guestrin, C. (2016). XGBoost: A scalable tree boosting system. Proc. 22nd ACM SIGKDD Int. Conf. Knowl. Discov. Data Min., 785-794.

Breiman, L. (2001). Random forests. Mach. Learn. 45, 5-32.

Bühlmann, P., and Hothorn, T. (2007). Boosting algorithms: Regularization, prediction and model fitting. Stat. Sci. 22, 477-505.

Bastien, P., Vinzi, V.E., and Tenenhaus, M. (2005). PLS generalised linear regression. Comput. Stat. Data Anal. 48, 17-46.

Rish, I. (2001). An empirical study of the naive Bayes classifier. IJCAI 2001 Work. Empir. Methods Artif. Intell. 3, 41-46.

# ****Supplementary Table 3. 28 differentially intersecting genes in sepsis and acute kidney injury****

| **No** | **Gene ID** | **No** | **Gene ID** | **No** | **Gene ID** | **No** | **Gene ID** |
| --- | --- | --- | --- | --- | --- | --- | --- |
| 1 | GADD45A | 8 | DACH1 | 15 | TGFBR3 | 22 | PCOLCE2 |
| 2 | CDC25B | 9 | F5 | 16 | CCL5 | 23 | RRM2 |
| 3 | VNN1 | 10 | NLRC3 | 17 | RPL10A | 24 | C1QA |
| 4 | GRB10 | 11 | NR3C2 | 18 | RPL36 | 25 | PRC1 |
| 5 | SLC2A3 | 12 | ST6GALNAC3 | 19 | CEACAM1 | 26 | HEPACAM2 |
| 6 | RAB13 | 13 | PLEKHO1 | 20 | HPGD | 27 | IFIT1 |
| 7 | DPP4 | 14 | MGAM | 21 | KIAA0101 | 28 | APOBEC3B |

# ****Supplementary Table 4. GO function enrichment analysis****

| **ID** | **ONTOLOGY** | **Description** | **GeneRatio** | **BgRatio** | **pvalue** | **p.adjust** | **qvalue** | **geneID** | **Count** |
| --- | --- | --- | --- | --- | --- | --- | --- | --- | --- |
| GO:0033632 | BP | regulation of cell-cell adhesion mediated by integrin | 2/27 | 11/17910 | 0.000119 | 0.08145 | 0.060979 | DPP4/CCL5 | 2 |
| GO:0045321 | BP | leukocyte activation | 8/27 | 1186/17910 | 0.000258 | 0.08145 | 0.060979 | DPP4/MGAM/SLC2A3/VNN1/NLRC3/CCL5/C1QA/CEACAM1 | 8 |
| GO:0033631 | BP | cell-cell adhesion mediated by integrin | 2/27 | 16/17910 | 0.000259 | 0.08145 | 0.060979 | DPP4/CCL5 | 2 |
| GO:0045087 | BP | innate immune response | 7/27 | 893/17910 | 0.000276 | 0.08145 | 0.060979 | IFIT1/APOBEC3B/VNN1/NLRC3/CCL5/C1QA/CEACAM1 | 7 |
| GO:0002274 | BP | myeloid leukocyte activation | 6/27 | 652/17910 | 0.00035 | 0.08145 | 0.060979 | MGAM/SLC2A3/VNN1/CCL5/C1QA/CEACAM1 | 6 |
| GO:0002544 | BP | chronic inflammatory response | 2/27 | 19/17910 | 0.000368 | 0.08145 | 0.060979 | VNN1/CCL5 | 2 |
| GO:0042110 | BP | T cell activation | 5/27 | 443/17910 | 0.000466 | 0.08145 | 0.060979 | DPP4/VNN1/NLRC3/CCL5/CEACAM1 | 5 |
| GO:0042127 | BP | regulation of cell proliferation | 9/27 | 1664/17910 | 0.000503 | 0.08145 | 0.060979 | CDC25B/TGFBR3/PRC1/DPP4/HPGD/DACH1/NLRC3/CCL5/CEACAM1 | 9 |
| GO:0001775 | BP | cell activation | 8/27 | 1342/17910 | 0.000595 | 0.08145 | 0.060979 | DPP4/MGAM/SLC2A3/VNN1/NLRC3/CCL5/C1QA/CEACAM1 | 8 |
| GO:0034110 | BP | regulation of homotypic cell-cell adhesion | 2/27 | 25/17910 | 0.000643 | 0.08145 | 0.060979 | CCL5/CEACAM1 | 2 |
| GO:0014066 | BP | regulation of phosphatidylinositol 3-kinase signaling | 3/27 | 117/17910 | 0.000709 | 0.08145 | 0.060979 | NLRC3/CCL5/CEACAM1 | 3 |
| GO:0006955 | BP | immune response | 10/27 | 2148/17910 | 0.000735 | 0.08145 | 0.060979 | TGFBR3/IFIT1/MGAM/SLC2A3/APOBEC3B/VNN1/NLRC3/CCL5/C1QA/CEACAM1 | 10 |
| GO:0002694 | BP | regulation of leukocyte activation | 5/27 | 492/17910 | 0.00075 | 0.08145 | 0.060979 | DPP4/VNN1/CCL5/C1QA/CEACAM1 | 5 |
| GO:0042119 | BP | neutrophil activation | 5/27 | 499/17910 | 0.0008 | 0.08145 | 0.060979 | MGAM/SLC2A3/VNN1/CCL5/CEACAM1 | 5 |
| GO:0036230 | BP | granulocyte activation | 5/27 | 506/17910 | 0.000851 | 0.08145 | 0.060979 | MGAM/SLC2A3/VNN1/CCL5/CEACAM1 | 5 |
| GO:1903037 | BP | regulation of leukocyte cell-cell adhesion | 4/27 | 295/17910 | 0.000939 | 0.08145 | 0.060979 | DPP4/VNN1/CCL5/CEACAM1 | 4 |
| GO:0002696 | BP | positive regulation of leukocyte activation | 4/27 | 305/17910 | 0.001062 | 0.08145 | 0.060979 | DPP4/VNN1/CCL5/C1QA | 4 |
| GO:0050863 | BP | regulation of T cell activation | 4/27 | 307/17910 | 0.001088 | 0.08145 | 0.060979 | DPP4/VNN1/CCL5/CEACAM1 | 4 |
| GO:0050865 | BP | regulation of cell activation | 5/27 | 535/17910 | 0.001093 | 0.08145 | 0.060979 | DPP4/VNN1/CCL5/C1QA/CEACAM1 | 5 |
| GO:0048147 | BP | negative regulation of fibroblast proliferation | 2/27 | 33/17910 | 0.001123 | 0.08145 | 0.060979 | DACH1/NLRC3 | 2 |
| GO:0014065 | BP | phosphatidylinositol 3-kinase signaling | 3/27 | 138/17910 | 0.001143 | 0.08145 | 0.060979 | NLRC3/CCL5/CEACAM1 | 3 |
| GO:0050867 | BP | positive regulation of cell activation | 4/27 | 319/17910 | 0.001254 | 0.082141 | 0.061496 | DPP4/VNN1/CCL5/C1QA | 4 |
| GO:0045070 | BP | positive regulation of viral genome replication | 2/27 | 35/17910 | 0.001263 | 0.082141 | 0.061496 | IFIT1/CCL5 | 2 |
| GO:0007159 | BP | leukocyte cell-cell adhesion | 4/27 | 328/17910 | 0.001389 | 0.083861 | 0.062784 | DPP4/VNN1/CCL5/CEACAM1 | 4 |
| GO:0009605 | BP | response to external stimulus | 10/27 | 2331/17910 | 0.001401 | 0.083861 | 0.062784 | RAB13/GADD45A/IFIT1/HPGD/APOBEC3B/NLRC3/CCL5/C1QA/GRB10/CEACAM1 | 10 |
| GO:0006887 | BP | exocytosis | 6/27 | 862/17910 | 0.001515 | 0.08717 | 0.065261 | F5/MGAM/SLC2A3/VNN1/CCL5/CEACAM1 | 6 |
| GO:0002252 | BP | immune effector process | 7/27 | 1197/17910 | 0.001588 | 0.087999 | 0.065882 | IFIT1/MGAM/SLC2A3/APOBEC3B/VNN1/C1QA/CEACAM1 | 7 |
| GO:0008283 | BP | cell proliferation | 9/27 | 1969/17910 | 0.001704 | 0.088973 | 0.066611 | CDC25B/TGFBR3/PRC1/DPP4/HPGD/DACH1/NLRC3/CCL5/CEACAM1 | 9 |
| GO:0048015 | BP | phosphatidylinositol-mediated signaling | 3/27 | 160/17910 | 0.001748 | 0.088973 | 0.066611 | NLRC3/CCL5/CEACAM1 | 3 |
| GO:0048017 | BP | inositol lipid-mediated signaling | 3/27 | 163/17910 | 0.001844 | 0.088973 | 0.066611 | NLRC3/CCL5/CEACAM1 | 3 |
| GO:0050680 | BP | negative regulation of epithelial cell proliferation | 3/27 | 163/17910 | 0.001844 | 0.088973 | 0.066611 | TGFBR3/NLRC3/CEACAM1 | 3 |
| GO:0033628 | BP | regulation of cell adhesion mediated by integrin | 2/27 | 45/17910 | 0.002082 | 0.093478 | 0.069984 | DPP4/CCL5 | 2 |
| GO:0009725 | BP | response to hormone | 6/27 | 919/17910 | 0.0021 | 0.093478 | 0.069984 | TGFBR3/RAB13/NR3C2/HPGD/GRB10/CEACAM1 | 6 |
| GO:0050678 | BP | regulation of epithelial cell proliferation | 4/27 | 369/17910 | 0.002138 | 0.093478 | 0.069984 | TGFBR3/NLRC3/CCL5/CEACAM1 | 4 |
| GO:0008284 | BP | positive regulation of cell proliferation | 6/27 | 931/17910 | 0.002243 | 0.093478 | 0.069984 | CDC25B/TGFBR3/PRC1/DPP4/HPGD/CCL5 | 6 |
| GO:0046649 | BP | lymphocyte activation | 5/27 | 630/17910 | 0.002249 | 0.093478 | 0.069984 | DPP4/VNN1/NLRC3/CCL5/CEACAM1 | 5 |
| GO:0019083 | BP | viral transcription | 3/27 | 177/17910 | 0.002331 | 0.09426 | 0.070569 | RPL10A/RPL36/CCL5 | 3 |
| GO:0006413 | BP | translational initiation | 3/27 | 183/17910 | 0.002562 | 0.095189 | 0.071264 | RPL10A/RPL36/CCL5 | 3 |
| GO:0006952 | BP | defense response | 8/27 | 1681/17910 | 0.002582 | 0.095189 | 0.071264 | DPP4/IFIT1/APOBEC3B/VNN1/NLRC3/CCL5/C1QA/CEACAM1 | 8 |
| GO:0022407 | BP | regulation of cell-cell adhesion | 4/27 | 389/17910 | 0.002589 | 0.095189 | 0.071264 | DPP4/VNN1/CCL5/CEACAM1 | 4 |
| GO:0071495 | BP | cellular response to endogenous stimulus | 7/27 | 1305/17910 | 0.002609 | 0.095189 | 0.071264 | TGFBR3/RAB13/NR3C2/HPGD/CCL5/GRB10/CEACAM1 | 7 |
| GO:0051249 | BP | regulation of lymphocyte activation | 4/27 | 401/17910 | 0.002889 | 0.100623 | 0.075332 | DPP4/VNN1/CCL5/CEACAM1 | 4 |
| GO:0019080 | BP | viral gene expression | 3/27 | 191/17910 | 0.002892 | 0.100623 | 0.075332 | RPL10A/RPL36/CCL5 | 3 |
| GO:0043434 | BP | response to peptide hormone | 4/27 | 404/17910 | 0.002968 | 0.100906 | 0.075545 | TGFBR3/RAB13/GRB10/CEACAM1 | 4 |
| GO:0032869 | BP | cellular response to insulin stimulus | 3/27 | 197/17910 | 0.003156 | 0.101897 | 0.076287 | RAB13/GRB10/CEACAM1 | 3 |
| GO:0002376 | BP | immune system process | 11/27 | 3063/17910 | 0.003198 | 0.101897 | 0.076287 | TGFBR3/DPP4/IFIT1/MGAM/SLC2A3/APOBEC3B/VNN1/NLRC3/CCL5/C1QA/CEACAM1 | 11 |
| GO:0050870 | BP | positive regulation of T cell activation | 3/27 | 198/17910 | 0.003201 | 0.101897 | 0.076287 | DPP4/VNN1/CCL5 | 3 |
| GO:0045824 | BP | negative regulation of innate immune response | 2/27 | 57/17910 | 0.003319 | 0.103442 | 0.077443 | NLRC3/CEACAM1 | 2 |
| GO:0045071 | BP | negative regulation of viral genome replication | 2/27 | 59/17910 | 0.003552 | 0.104418 | 0.078174 | IFIT1/CCL5 | 2 |
| GO:1903902 | BP | positive regulation of viral life cycle | 2/27 | 59/17910 | 0.003552 | 0.104418 | 0.078174 | IFIT1/CCL5 | 2 |
| GO:0050673 | BP | epithelial cell proliferation | 4/27 | 425/17910 | 0.00356 | 0.104418 | 0.078174 | TGFBR3/NLRC3/CCL5/CEACAM1 | 4 |
| GO:0033627 | BP | cell adhesion mediated by integrin | 2/27 | 61/17910 | 0.003792 | 0.105333 | 0.078859 | DPP4/CCL5 | 2 |
| GO:1903039 | BP | positive regulation of leukocyte cell-cell adhesion | 3/27 | 214/17910 | 0.003982 | 0.105333 | 0.078859 | DPP4/VNN1/CCL5 | 3 |
| GO:0040011 | BP | locomotion | 8/27 | 1835/17910 | 0.004465 | 0.105333 | 0.078859 | TGFBR3/RAB13/DPP4/PLEKHO1/DACH1/CCL5/GRB10/CEACAM1 | 8 |
| GO:0000023 | BP | maltose metabolic process | 1/27 | 3/17910 | 0.004516 | 0.105333 | 0.078859 | MGAM | 1 |
| GO:0007181 | BP | transforming growth factor beta receptor complex assembly | 1/27 | 3/17910 | 0.004516 | 0.105333 | 0.078859 | TGFBR3 | 1 |
| GO:0043316 | BP | cytotoxic T cell degranulation | 1/27 | 3/17910 | 0.004516 | 0.105333 | 0.078859 | CEACAM1 | 1 |
| GO:0150062 | BP | complement-mediated synapse pruning | 1/27 | 3/17910 | 0.004516 | 0.105333 | 0.078859 | C1QA | 1 |
| GO:0150064 | BP | vertebrate eye-specific patterning | 1/27 | 3/17910 | 0.004516 | 0.105333 | 0.078859 | C1QA | 1 |
| GO:1903385 | BP | regulation of homophilic cell adhesion | 1/27 | 3/17910 | 0.004516 | 0.105333 | 0.078859 | CEACAM1 | 1 |
| GO:0032940 | BP | secretion by cell | 7/27 | 1449/17910 | 0.004693 | 0.105333 | 0.078859 | F5/DPP4/MGAM/SLC2A3/VNN1/CCL5/CEACAM1 | 7 |
| GO:0002181 | BP | cytoplasmic translation | 2/27 | 69/17910 | 0.004825 | 0.105333 | 0.078859 | RPL10A/RPL36 | 2 |
| GO:0097305 | BP | response to alcohol | 3/27 | 232/17910 | 0.004989 | 0.105333 | 0.078859 | TGFBR3/RPL10A/HPGD | 3 |
| GO:0010564 | BP | regulation of cell cycle process | 5/27 | 764/17910 | 0.00515 | 0.105333 | 0.078859 | CDC25B/GADD45A/PRC1/RRM2/DACH1 | 5 |
| GO:0016477 | BP | cell migration | 7/27 | 1474/17910 | 0.005158 | 0.105333 | 0.078859 | TGFBR3/RAB13/DPP4/PLEKHO1/DACH1/CCL5/CEACAM1 | 7 |
| GO:0045055 | BP | regulated exocytosis | 5/27 | 768/17910 | 0.005265 | 0.105333 | 0.078859 | F5/MGAM/SLC2A3/VNN1/CEACAM1 | 5 |
| GO:0033002 | BP | muscle cell proliferation | 3/27 | 243/17910 | 0.005673 | 0.105333 | 0.078859 | TGFBR3/HPGD/CCL5 | 3 |
| GO:0043312 | BP | neutrophil degranulation | 4/27 | 485/17910 | 0.005686 | 0.105333 | 0.078859 | MGAM/SLC2A3/VNN1/CEACAM1 | 4 |
| GO:0002283 | BP | neutrophil activation involved in immune response | 4/27 | 488/17910 | 0.00581 | 0.105333 | 0.078859 | MGAM/SLC2A3/VNN1/CEACAM1 | 4 |
| GO:1901652 | BP | response to peptide | 4/27 | 491/17910 | 0.005937 | 0.105333 | 0.078859 | TGFBR3/RAB13/GRB10/CEACAM1 | 4 |
| GO:0034109 | BP | homotypic cell-cell adhesion | 2/27 | 77/17910 | 0.005973 | 0.105333 | 0.078859 | CCL5/CEACAM1 | 2 |
| GO:0034699 | BP | response to luteinizing hormone | 1/27 | 4/17910 | 0.006017 | 0.105333 | 0.078859 | TGFBR3 | 1 |
| GO:0036343 | BP | psychomotor behavior | 1/27 | 4/17910 | 0.006017 | 0.105333 | 0.078859 | DPP4 | 1 |
| GO:0060244 | BP | negative regulation of cell proliferation involved in contact inhibition | 1/27 | 4/17910 | 0.006017 | 0.105333 | 0.078859 | DACH1 | 1 |
| GO:0060935 | BP | cardiac fibroblast cell differentiation | 1/27 | 4/17910 | 0.006017 | 0.105333 | 0.078859 | TGFBR3 | 1 |
| GO:0060936 | BP | cardiac fibroblast cell development | 1/27 | 4/17910 | 0.006017 | 0.105333 | 0.078859 | TGFBR3 | 1 |
| GO:0060938 | BP | epicardium-derived cardiac fibroblast cell differentiation | 1/27 | 4/17910 | 0.006017 | 0.105333 | 0.078859 | TGFBR3 | 1 |
| GO:0060939 | BP | epicardium-derived cardiac fibroblast cell development | 1/27 | 4/17910 | 0.006017 | 0.105333 | 0.078859 | TGFBR3 | 1 |
| GO:2000346 | BP | negative regulation of hepatocyte proliferation | 1/27 | 4/17910 | 0.006017 | 0.105333 | 0.078859 | CEACAM1 | 1 |
| GO:0001570 | BP | vasculogenesis | 2/27 | 78/17910 | 0.006125 | 0.105333 | 0.078859 | TGFBR3/CEACAM1 | 2 |
| GO:0022409 | BP | positive regulation of cell-cell adhesion | 3/27 | 251/17910 | 0.006205 | 0.105333 | 0.078859 | DPP4/VNN1/CCL5 | 3 |
| GO:0032868 | BP | response to insulin | 3/27 | 252/17910 | 0.006273 | 0.105333 | 0.078859 | RAB13/GRB10/CEACAM1 | 3 |
| GO:0002446 | BP | neutrophil mediated immunity | 4/27 | 499/17910 | 0.006282 | 0.105333 | 0.078859 | MGAM/SLC2A3/VNN1/CEACAM1 | 4 |
| GO:0051726 | BP | regulation of cell cycle | 6/27 | 1149/17910 | 0.006348 | 0.105333 | 0.078859 | CDC25B/GADD45A/PRC1/RRM2/HPGD/DACH1 | 6 |
| GO:0051251 | BP | positive regulation of lymphocyte activation | 3/27 | 258/17910 | 0.006694 | 0.105333 | 0.078859 | DPP4/VNN1/CCL5 | 3 |
| GO:0002443 | BP | leukocyte mediated immunity | 5/27 | 814/17910 | 0.006719 | 0.105333 | 0.078859 | MGAM/SLC2A3/VNN1/C1QA/CEACAM1 | 5 |
| GO:1901700 | BP | response to oxygen-containing compound | 7/27 | 1551/17910 | 0.006813 | 0.105333 | 0.078859 | TGFBR3/RAB13/RPL10A/HPGD/CCL5/GRB10/CEACAM1 | 7 |
| GO:0032465 | BP | regulation of cytokinesis | 2/27 | 83/17910 | 0.006908 | 0.105333 | 0.078859 | CDC25B/PRC1 | 2 |
| GO:0016032 | BP | viral process | 5/27 | 822/17910 | 0.006999 | 0.105333 | 0.078859 | RPL10A/RPL36/DPP4/IFIT1/CCL5 | 5 |
| GO:0009719 | BP | response to endogenous stimulus | 7/27 | 1560/17910 | 0.00703 | 0.105333 | 0.078859 | TGFBR3/RAB13/NR3C2/HPGD/CCL5/GRB10/CEACAM1 | 7 |
| GO:0006767 | BP | water-soluble vitamin metabolic process | 2/27 | 84/17910 | 0.00707 | 0.105333 | 0.078859 | SLC2A3/VNN1 | 2 |
| GO:1903901 | BP | negative regulation of viral life cycle | 2/27 | 85/17910 | 0.007234 | 0.105333 | 0.078859 | IFIT1/CCL5 | 2 |
| GO:0002682 | BP | regulation of immune system process | 7/27 | 1570/17910 | 0.007277 | 0.105333 | 0.078859 | DPP4/IFIT1/VNN1/NLRC3/CCL5/C1QA/CEACAM1 | 7 |
| GO:0048145 | BP | regulation of fibroblast proliferation | 2/27 | 86/17910 | 0.007399 | 0.105333 | 0.078859 | DACH1/NLRC3 | 2 |
| GO:0003150 | BP | muscular septum morphogenesis | 1/27 | 5/17910 | 0.007516 | 0.105333 | 0.078859 | TGFBR3 | 1 |
| GO:0010716 | BP | negative regulation of extracellular matrix disassembly | 1/27 | 5/17910 | 0.007516 | 0.105333 | 0.078859 | DPP4 | 1 |
| GO:0031584 | BP | activation of phospholipase D activity | 1/27 | 5/17910 | 0.007516 | 0.105333 | 0.078859 | CCL5 | 1 |
| GO:0051097 | BP | negative regulation of helicase activity | 1/27 | 5/17910 | 0.007516 | 0.105333 | 0.078859 | IFIT1 | 1 |
| GO:0060318 | BP | definitive erythrocyte differentiation | 1/27 | 5/17910 | 0.007516 | 0.105333 | 0.078859 | TGFBR3 | 1 |
| GO:0097070 | BP | ductus arteriosus closure | 1/27 | 5/17910 | 0.007516 | 0.105333 | 0.078859 | HPGD | 1 |
| GO:0097368 | BP | establishment of Sertoli cell barrier | 1/27 | 5/17910 | 0.007516 | 0.105333 | 0.078859 | RAB13 | 1 |
| GO:1900226 | BP | negative regulation of NLRP3 inflammasome complex assembly | 1/27 | 5/17910 | 0.007516 | 0.105333 | 0.078859 | NLRC3 | 1 |
| GO:1901142 | BP | insulin metabolic process | 1/27 | 5/17910 | 0.007516 | 0.105333 | 0.078859 | CEACAM1 | 1 |
| GO:0048144 | BP | fibroblast proliferation | 2/27 | 87/17910 | 0.007567 | 0.105333 | 0.078859 | DACH1/NLRC3 | 2 |
| GO:0046903 | BP | secretion | 7/27 | 1584/17910 | 0.007634 | 0.105333 | 0.078859 | F5/DPP4/MGAM/SLC2A3/VNN1/CCL5/CEACAM1 | 7 |
| GO:0006928 | BP | movement of cell or subcellular component | 8/27 | 2012/17910 | 0.007808 | 0.105333 | 0.078859 | TGFBR3/RAB13/DPP4/PLEKHO1/DACH1/CCL5/GRB10/CEACAM1 | 8 |
| GO:0080134 | BP | regulation of response to stress | 7/27 | 1592/17910 | 0.007844 | 0.105333 | 0.078859 | GADD45A/IFIT1/VNN1/NLRC3/CCL5/C1QA/CEACAM1 | 7 |
| GO:0043299 | BP | leukocyte degranulation | 4/27 | 532/17910 | 0.007852 | 0.105333 | 0.078859 | MGAM/SLC2A3/VNN1/CEACAM1 | 4 |
| GO:0043542 | BP | endothelial cell migration | 3/27 | 275/17910 | 0.007975 | 0.105333 | 0.078859 | RAB13/DPP4/CEACAM1 | 3 |
| GO:0048870 | BP | cell motility | 7/27 | 1611/17910 | 0.008359 | 0.105333 | 0.078859 | TGFBR3/RAB13/DPP4/PLEKHO1/DACH1/CCL5/CEACAM1 | 7 |
| GO:0051674 | BP | localization of cell | 7/27 | 1611/17910 | 0.008359 | 0.105333 | 0.078859 | TGFBR3/RAB13/DPP4/PLEKHO1/DACH1/CCL5/CEACAM1 | 7 |
| GO:0002275 | BP | myeloid cell activation involved in immune response | 4/27 | 542/17910 | 0.008374 | 0.105333 | 0.078859 | MGAM/SLC2A3/VNN1/CEACAM1 | 4 |
| GO:0051704 | BP | multi-organism process | 9/27 | 2496/17910 | 0.008581 | 0.105333 | 0.078859 | CDC25B/RPL10A/RPL36/DPP4/IFIT1/HPGD/APOBEC3B/DACH1/CCL5 | 9 |
| GO:0002444 | BP | myeloid leukocyte mediated immunity | 4/27 | 549/17910 | 0.008753 | 0.105333 | 0.078859 | MGAM/SLC2A3/VNN1/CEACAM1 | 4 |
| GO:0045069 | BP | regulation of viral genome replication | 2/27 | 95/17910 | 0.008964 | 0.105333 | 0.078859 | IFIT1/CCL5 | 2 |
| GO:0032101 | BP | regulation of response to external stimulus | 5/27 | 873/17910 | 0.008976 | 0.105333 | 0.078859 | IFIT1/NLRC3/CCL5/C1QA/CEACAM1 | 5 |
| GO:0010536 | BP | positive regulation of activation of Janus kinase activity | 1/27 | 6/17910 | 0.009012 | 0.105333 | 0.078859 | CCL5 | 1 |
| GO:0015939 | BP | pantothenate metabolic process | 1/27 | 6/17910 | 0.009012 | 0.105333 | 0.078859 | VNN1 | 1 |
| GO:0032687 | BP | negative regulation of interferon-alpha production | 1/27 | 6/17910 | 0.009012 | 0.105333 | 0.078859 | NLRC3 | 1 |
| GO:0033634 | BP | positive regulation of cell-cell adhesion mediated by integrin | 1/27 | 6/17910 | 0.009012 | 0.105333 | 0.078859 | CCL5 | 1 |
| GO:0034112 | BP | positive regulation of homotypic cell-cell adhesion | 1/27 | 6/17910 | 0.009012 | 0.105333 | 0.078859 | CCL5 | 1 |
| GO:0044245 | BP | polysaccharide digestion | 1/27 | 6/17910 | 0.009012 | 0.105333 | 0.078859 | MGAM | 1 |
| GO:0060979 | BP | vasculogenesis involved in coronary vascular morphogenesis | 1/27 | 6/17910 | 0.009012 | 0.105333 | 0.078859 | TGFBR3 | 1 |
| GO:0061890 | BP | positive regulation of astrocyte activation | 1/27 | 6/17910 | 0.009012 | 0.105333 | 0.078859 | C1QA | 1 |
| GO:2000110 | BP | negative regulation of macrophage apoptotic process | 1/27 | 6/17910 | 0.009012 | 0.105333 | 0.078859 | CCL5 | 1 |
| GO:2000503 | BP | positive regulation of natural killer cell chemotaxis | 1/27 | 6/17910 | 0.009012 | 0.105333 | 0.078859 | CCL5 | 1 |
| GO:2001300 | BP | lipoxin metabolic process | 1/27 | 6/17910 | 0.009012 | 0.105333 | 0.078859 | HPGD | 1 |
| GO:2001301 | BP | lipoxin biosynthetic process | 1/27 | 6/17910 | 0.009012 | 0.105333 | 0.078859 | HPGD | 1 |
| GO:0044403 | BP | symbiont process | 5/27 | 884/17910 | 0.009449 | 0.10766 | 0.080601 | RPL10A/RPL36/DPP4/IFIT1/CCL5 | 5 |
| GO:0031347 | BP | regulation of defense response | 5/27 | 885/17910 | 0.009492 | 0.10766 | 0.080601 | IFIT1/NLRC3/CCL5/C1QA/CEACAM1 | 5 |
| GO:0048525 | BP | negative regulation of viral process | 2/27 | 99/17910 | 0.009703 | 0.10766 | 0.080601 | IFIT1/CCL5 | 2 |
| GO:0071375 | BP | cellular response to peptide hormone stimulus | 3/27 | 297/17910 | 0.009837 | 0.10766 | 0.080601 | RAB13/GRB10/CEACAM1 | 3 |
| GO:0009968 | BP | negative regulation of signal transduction | 6/27 | 1266/17910 | 0.010076 | 0.10766 | 0.080601 | TGFBR3/VNN1/NLRC3/CCL5/GRB10/CEACAM1 | 6 |
| GO:0048661 | BP | positive regulation of smooth muscle cell proliferation | 2/27 | 101/17910 | 0.010083 | 0.10766 | 0.080601 | HPGD/CCL5 | 2 |
| GO:0071310 | BP | cellular response to organic substance | 9/27 | 2564/17910 | 0.010213 | 0.10766 | 0.080601 | TGFBR3/PCOLCE2/RAB13/NR3C2/IFIT1/HPGD/CCL5/GRB10/CEACAM1 | 9 |
| GO:0006614 | BP | SRP-dependent cotranslational protein targeting to membrane | 2/27 | 103/17910 | 0.010469 | 0.10766 | 0.080601 | RPL10A/RPL36 | 2 |
| GO:0014812 | BP | muscle cell migration | 2/27 | 103/17910 | 0.010469 | 0.10766 | 0.080601 | PLEKHO1/CCL5 | 2 |
| GO:0048524 | BP | positive regulation of viral process | 2/27 | 103/17910 | 0.010469 | 0.10766 | 0.080601 | IFIT1/CCL5 | 2 |
| GO:0001915 | BP | negative regulation of T cell mediated cytotoxicity | 1/27 | 7/17910 | 0.010507 | 0.10766 | 0.080601 | CEACAM1 | 1 |
| GO:0007144 | BP | female meiosis I | 1/27 | 7/17910 | 0.010507 | 0.10766 | 0.080601 | CDC25B | 1 |
| GO:0035726 | BP | common myeloid progenitor cell proliferation | 1/27 | 7/17910 | 0.010507 | 0.10766 | 0.080601 | CEACAM1 | 1 |
| GO:0046719 | BP | regulation by virus of viral protein levels in host cell | 1/27 | 7/17910 | 0.010507 | 0.10766 | 0.080601 | IFIT1 | 1 |
| GO:0061744 | BP | motor behavior | 1/27 | 7/17910 | 0.010507 | 0.10766 | 0.080601 | DPP4 | 1 |
| GO:0070100 | BP | negative regulation of chemokine-mediated signaling pathway | 1/27 | 7/17910 | 0.010507 | 0.10766 | 0.080601 | CCL5 | 1 |
| GO:1902581 | BP | multi-organism cellular localization | 1/27 | 7/17910 | 0.010507 | 0.10766 | 0.080601 | IFIT1 | 1 |
| GO:1902583 | BP | multi-organism intracellular transport | 1/27 | 7/17910 | 0.010507 | 0.10766 | 0.080601 | IFIT1 | 1 |
| GO:0022402 | BP | cell cycle process | 6/27 | 1280/17910 | 0.010609 | 0.107969 | 0.080833 | CDC25B/GADD45A/PRC1/RRM2/DACH1/HEPACAM2 | 6 |
| GO:0006613 | BP | cotranslational protein targeting to membrane | 2/27 | 106/17910 | 0.01106 | 0.111793 | 0.083695 | RPL10A/RPL36 | 2 |
| GO:1904659 | BP | glucose transmembrane transport | 2/27 | 107/17910 | 0.01126 | 0.113053 | 0.084639 | SLC2A3/GRB10 | 2 |
| GO:0045047 | BP | protein targeting to ER | 2/27 | 108/17910 | 0.011462 | 0.113138 | 0.084702 | RPL10A/RPL36 | 2 |
| GO:0044419 | BP | interspecies interaction between organisms | 5/27 | 928/17910 | 0.011515 | 0.113138 | 0.084702 | RPL10A/RPL36/DPP4/IFIT1/CCL5 | 5 |
| GO:0070887 | BP | cellular response to chemical stimulus | 10/27 | 3100/17910 | 0.011538 | 0.113138 | 0.084702 | TGFBR3/PCOLCE2/RAB13/NR3C2/IFIT1/HPGD/VNN1/CCL5/GRB10/CEACAM1 | 10 |
| GO:0007049 | BP | cell cycle | 7/27 | 1716/17910 | 0.011683 | 0.113138 | 0.084702 | CDC25B/GADD45A/PRC1/RRM2/HPGD/DACH1/HEPACAM2 | 7 |
| GO:0002420 | BP | natural killer cell mediated cytotoxicity directed against tumor cell target | 1/27 | 8/17910 | 0.011999 | 0.113138 | 0.084702 | CEACAM1 | 1 |
| GO:0002858 | BP | regulation of natural killer cell mediated cytotoxicity directed against tumor cell target | 1/27 | 8/17910 | 0.011999 | 0.113138 | 0.084702 | CEACAM1 | 1 |
| GO:0003223 | BP | ventricular compact myocardium morphogenesis | 1/27 | 8/17910 | 0.011999 | 0.113138 | 0.084702 | TGFBR3 | 1 |
| GO:0010533 | BP | regulation of activation of Janus kinase activity | 1/27 | 8/17910 | 0.011999 | 0.113138 | 0.084702 | CCL5 | 1 |
| GO:0030853 | BP | negative regulation of granulocyte differentiation | 1/27 | 8/17910 | 0.011999 | 0.113138 | 0.084702 | CEACAM1 | 1 |
| GO:0009607 | BP | response to biotic stimulus | 5/27 | 938/17910 | 0.012025 | 0.113138 | 0.084702 | IFIT1/HPGD/APOBEC3B/CCL5/CEACAM1 | 5 |
| GO:0007098 | BP | centrosome cycle | 2/27 | 112/17910 | 0.012285 | 0.113244 | 0.084782 | GADD45A/HEPACAM2 | 2 |
| GO:0008645 | BP | hexose transmembrane transport | 2/27 | 112/17910 | 0.012285 | 0.113244 | 0.084782 | SLC2A3/GRB10 | 2 |
| GO:0072599 | BP | establishment of protein localization to endoplasmic reticulum | 2/27 | 112/17910 | 0.012285 | 0.113244 | 0.084782 | RPL10A/RPL36 | 2 |
| GO:0009615 | BP | response to virus | 3/27 | 323/17910 | 0.012339 | 0.113244 | 0.084782 | IFIT1/APOBEC3B/CCL5 | 3 |
| GO:0019058 | BP | viral life cycle | 3/27 | 324/17910 | 0.012442 | 0.113492 | 0.084967 | DPP4/IFIT1/CCL5 | 3 |
| GO:0010033 | BP | response to organic substance | 10/27 | 3138/17910 | 0.012555 | 0.113826 | 0.085217 | TGFBR3/PCOLCE2/RAB13/RPL10A/NR3C2/IFIT1/HPGD/CCL5/GRB10/CEACAM1 | 10 |
| GO:0015749 | BP | monosaccharide transmembrane transport | 2/27 | 114/17910 | 0.012706 | 0.113826 | 0.085217 | SLC2A3/GRB10 | 2 |
| GO:1901222 | BP | regulation of NIK/NF-kappaB signaling | 2/27 | 114/17910 | 0.012706 | 0.113826 | 0.085217 | NR3C2/NLRC3 | 2 |
| GO:0034219 | BP | carbohydrate transmembrane transport | 2/27 | 116/17910 | 0.013134 | 0.114011 | 0.085356 | SLC2A3/GRB10 | 2 |
| GO:1901699 | BP | cellular response to nitrogen compound | 4/27 | 620/17910 | 0.013253 | 0.114011 | 0.085356 | RAB13/IFIT1/GRB10/CEACAM1 | 4 |
| GO:0000022 | BP | mitotic spindle elongation | 1/27 | 9/17910 | 0.013489 | 0.114011 | 0.085356 | PRC1 | 1 |
| GO:0002423 | BP | natural killer cell mediated immune response to tumor cell | 1/27 | 9/17910 | 0.013489 | 0.114011 | 0.085356 | CEACAM1 | 1 |
| GO:0002676 | BP | regulation of chronic inflammatory response | 1/27 | 9/17910 | 0.013489 | 0.114011 | 0.085356 | CCL5 | 1 |
| GO:0002855 | BP | regulation of natural killer cell mediated immune response to tumor cell | 1/27 | 9/17910 | 0.013489 | 0.114011 | 0.085356 | CEACAM1 | 1 |
| GO:0005984 | BP | disaccharide metabolic process | 1/27 | 9/17910 | 0.013489 | 0.114011 | 0.085356 | MGAM | 1 |
| GO:0019852 | BP | L-ascorbic acid metabolic process | 1/27 | 9/17910 | 0.013489 | 0.114011 | 0.085356 | SLC2A3 | 1 |
| GO:0060242 | BP | contact inhibition | 1/27 | 9/17910 | 0.013489 | 0.114011 | 0.085356 | DACH1 | 1 |
| GO:2000501 | BP | regulation of natural killer cell chemotaxis | 1/27 | 9/17910 | 0.013489 | 0.114011 | 0.085356 | CCL5 | 1 |
| GO:0000184 | BP | nuclear-transcribed mRNA catabolic process, nonsense-mediated decay | 2/27 | 118/17910 | 0.013568 | 0.11403 | 0.08537 | RPL10A/RPL36 | 2 |
| GO:0019079 | BP | viral genome replication | 2/27 | 120/17910 | 0.014008 | 0.117069 | 0.087645 | IFIT1/CCL5 | 2 |
| GO:0010648 | BP | negative regulation of cell communication | 6/27 | 1364/17910 | 0.014249 | 0.117926 | 0.088287 | TGFBR3/VNN1/NLRC3/CCL5/GRB10/CEACAM1 | 6 |
| GO:0023057 | BP | negative regulation of signaling | 6/27 | 1368/17910 | 0.014442 | 0.117926 | 0.088287 | TGFBR3/VNN1/NLRC3/CCL5/GRB10/CEACAM1 | 6 |
| GO:0031023 | BP | microtubule organizing center organization | 2/27 | 122/17910 | 0.014454 | 0.117926 | 0.088287 | GADD45A/HEPACAM2 | 2 |
| GO:0009263 | BP | deoxyribonucleotide biosynthetic process | 1/27 | 10/17910 | 0.014977 | 0.117926 | 0.088287 | RRM2 | 1 |
| GO:0010944 | BP | negative regulation of transcription by competitive promoter binding | 1/27 | 10/17910 | 0.014977 | 0.117926 | 0.088287 | DACH1 | 1 |
| GO:0048245 | BP | eosinophil chemotaxis | 1/27 | 10/17910 | 0.014977 | 0.117926 | 0.088287 | CCL5 | 1 |
| GO:0051231 | BP | spindle elongation | 1/27 | 10/17910 | 0.014977 | 0.117926 | 0.088287 | PRC1 | 1 |
| GO:0070099 | BP | regulation of chemokine-mediated signaling pathway | 1/27 | 10/17910 | 0.014977 | 0.117926 | 0.088287 | CCL5 | 1 |
| GO:0098883 | BP | synapse pruning | 1/27 | 10/17910 | 0.014977 | 0.117926 | 0.088287 | C1QA | 1 |
| GO:2000109 | BP | regulation of macrophage apoptotic process | 1/27 | 10/17910 | 0.014977 | 0.117926 | 0.088287 | CCL5 | 1 |
| GO:2001214 | BP | positive regulation of vasculogenesis | 1/27 | 10/17910 | 0.014977 | 0.117926 | 0.088287 | CEACAM1 | 1 |
| GO:0032870 | BP | cellular response to hormone stimulus | 4/27 | 646/17910 | 0.015214 | 0.119167 | 0.089216 | RAB13/NR3C2/GRB10/CEACAM1 | 4 |
| GO:0045471 | BP | response to ethanol | 2/27 | 127/17910 | 0.015596 | 0.121517 | 0.090975 | RPL10A/HPGD | 2 |
| GO:0010631 | BP | epithelial cell migration | 3/27 | 354/17910 | 0.015762 | 0.121924 | 0.09128 | RAB13/DPP4/CEACAM1 | 3 |
| GO:0070972 | BP | protein localization to endoplasmic reticulum | 2/27 | 128/17910 | 0.015829 | 0.121924 | 0.09128 | RPL10A/RPL36 | 2 |
| GO:1901698 | BP | response to nitrogen compound | 5/27 | 1006/17910 | 0.015911 | 0.121924 | 0.09128 | TGFBR3/RAB13/IFIT1/GRB10/CEACAM1 | 5 |
| GO:0090132 | BP | epithelium migration | 3/27 | 357/17910 | 0.016119 | 0.121924 | 0.09128 | RAB13/DPP4/CEACAM1 | 3 |
| GO:0030155 | BP | regulation of cell adhesion | 4/27 | 659/17910 | 0.016261 | 0.121924 | 0.09128 | DPP4/VNN1/CCL5/CEACAM1 | 4 |
| GO:0001889 | BP | liver development | 2/27 | 130/17910 | 0.016299 | 0.121924 | 0.09128 | TGFBR3/CEACAM1 | 2 |
| GO:0006766 | BP | vitamin metabolic process | 2/27 | 130/17910 | 0.016299 | 0.121924 | 0.09128 | SLC2A3/VNN1 | 2 |
| GO:0000185 | BP | activation of MAPKKK activity | 1/27 | 11/17910 | 0.016463 | 0.121924 | 0.09128 | GADD45A | 1 |
| GO:0033089 | BP | positive regulation of T cell differentiation in thymus | 1/27 | 11/17910 | 0.016463 | 0.121924 | 0.09128 | VNN1 | 1 |
| GO:0035747 | BP | natural killer cell chemotaxis | 1/27 | 11/17910 | 0.016463 | 0.121924 | 0.09128 | CCL5 | 1 |
| GO:0007292 | BP | female gamete generation | 2/27 | 132/17910 | 0.016775 | 0.122037 | 0.091365 | CDC25B/HPGD | 2 |
| GO:0090130 | BP | tissue migration | 3/27 | 363/17910 | 0.016847 | 0.122037 | 0.091365 | RAB13/DPP4/CEACAM1 | 3 |
| GO:1901653 | BP | cellular response to peptide | 3/27 | 364/17910 | 0.01697 | 0.122037 | 0.091365 | RAB13/GRB10/CEACAM1 | 3 |
| GO:0061008 | BP | hepaticobiliary system development | 2/27 | 133/17910 | 0.017016 | 0.122037 | 0.091365 | TGFBR3/CEACAM1 | 2 |
| GO:0071363 | BP | cellular response to growth factor stimulus | 4/27 | 674/17910 | 0.017525 | 0.122037 | 0.091365 | TGFBR3/HPGD/CCL5/GRB10 | 4 |
| GO:0006950 | BP | response to stress | 11/27 | 3822/17910 | 0.017656 | 0.122037 | 0.091365 | TGFBR3/F5/GADD45A/DPP4/IFIT1/APOBEC3B/VNN1/NLRC3/CCL5/C1QA/CEACAM1 | 11 |
| GO:0006216 | BP | cytidine catabolic process | 1/27 | 12/17910 | 0.017947 | 0.122037 | 0.091365 | APOBEC3B | 1 |
| GO:0009972 | BP | cytidine deamination | 1/27 | 12/17910 | 0.017947 | 0.122037 | 0.091365 | APOBEC3B | 1 |
| GO:0016322 | BP | neuron remodeling | 1/27 | 12/17910 | 0.017947 | 0.122037 | 0.091365 | C1QA | 1 |
| GO:0035641 | BP | locomotory exploration behavior | 1/27 | 12/17910 | 0.017947 | 0.122037 | 0.091365 | DPP4 | 1 |
| GO:0043301 | BP | negative regulation of leukocyte degranulation | 1/27 | 12/17910 | 0.017947 | 0.122037 | 0.091365 | CEACAM1 | 1 |
| GO:0043922 | BP | negative regulation by host of viral transcription | 1/27 | 12/17910 | 0.017947 | 0.122037 | 0.091365 | CCL5 | 1 |
| GO:0046087 | BP | cytidine metabolic process | 1/27 | 12/17910 | 0.017947 | 0.122037 | 0.091365 | APOBEC3B | 1 |
| GO:0051095 | BP | regulation of helicase activity | 1/27 | 12/17910 | 0.017947 | 0.122037 | 0.091365 | IFIT1 | 1 |
| GO:0051451 | BP | myoblast migration | 1/27 | 12/17910 | 0.017947 | 0.122037 | 0.091365 | PLEKHO1 | 1 |
| GO:0070493 | BP | thrombin-activated receptor signaling pathway | 1/27 | 12/17910 | 0.017947 | 0.122037 | 0.091365 | HPGD | 1 |
| GO:0071888 | BP | macrophage apoptotic process | 1/27 | 12/17910 | 0.017947 | 0.122037 | 0.091365 | CCL5 | 1 |
| GO:0090331 | BP | negative regulation of platelet aggregation | 1/27 | 12/17910 | 0.017947 | 0.122037 | 0.091365 | CEACAM1 | 1 |
| GO:0002366 | BP | leukocyte activation involved in immune response | 4/27 | 684/17910 | 0.018401 | 0.123627 | 0.092555 | MGAM/SLC2A3/VNN1/CEACAM1 | 4 |
| GO:0002831 | BP | regulation of response to biotic stimulus | 2/27 | 139/17910 | 0.018489 | 0.123627 | 0.092555 | IFIT1/CEACAM1 | 2 |
| GO:0002263 | BP | cell activation involved in immune response | 4/27 | 688/17910 | 0.018759 | 0.123627 | 0.092555 | MGAM/SLC2A3/VNN1/CEACAM1 | 4 |
| GO:0043603 | BP | cellular amide metabolic process | 5/27 | 1051/17910 | 0.018907 | 0.123627 | 0.092555 | ST6GALNAC3/RPL10A/RPL36/VNN1/CCL5 | 5 |
| GO:0001967 | BP | suckling behavior | 1/27 | 13/17910 | 0.019428 | 0.123627 | 0.092555 | DACH1 | 1 |
| GO:0043116 | BP | negative regulation of vascular permeability | 1/27 | 13/17910 | 0.019428 | 0.123627 | 0.092555 | CEACAM1 | 1 |
| GO:0060312 | BP | regulation of blood vessel remodeling | 1/27 | 13/17910 | 0.019428 | 0.123627 | 0.092555 | CEACAM1 | 1 |
| GO:0060347 | BP | heart trabecula formation | 1/27 | 13/17910 | 0.019428 | 0.123627 | 0.092555 | TGFBR3 | 1 |
| GO:0061888 | BP | regulation of astrocyte activation | 1/27 | 13/17910 | 0.019428 | 0.123627 | 0.092555 | C1QA | 1 |
| GO:0071850 | BP | mitotic cell cycle arrest | 1/27 | 13/17910 | 0.019428 | 0.123627 | 0.092555 | GADD45A | 1 |
| GO:1900225 | BP | regulation of NLRP3 inflammasome complex assembly | 1/27 | 13/17910 | 0.019428 | 0.123627 | 0.092555 | NLRC3 | 1 |
| GO:0050777 | BP | negative regulation of immune response | 2/27 | 143/17910 | 0.019501 | 0.123627 | 0.092555 | NLRC3/CEACAM1 | 2 |
| GO:0016192 | BP | vesicle-mediated transport | 7/27 | 1899/17910 | 0.019681 | 0.123627 | 0.092555 | RAB13/F5/MGAM/SLC2A3/VNN1/CCL5/CEACAM1 | 7 |
| GO:0006633 | BP | fatty acid biosynthetic process | 2/27 | 144/17910 | 0.019757 | 0.123627 | 0.092555 | HPGD/CEACAM1 | 2 |
| GO:0045785 | BP | positive regulation of cell adhesion | 3/27 | 388/17910 | 0.020078 | 0.123627 | 0.092555 | DPP4/VNN1/CCL5 | 3 |
| GO:0070848 | BP | response to growth factor | 4/27 | 704/17910 | 0.020234 | 0.123627 | 0.092555 | TGFBR3/HPGD/CCL5/GRB10 | 4 |
| GO:0000910 | BP | cytokinesis | 2/27 | 147/17910 | 0.020535 | 0.123627 | 0.092555 | CDC25B/PRC1 | 2 |
| GO:1903900 | BP | regulation of viral life cycle | 2/27 | 147/17910 | 0.020535 | 0.123627 | 0.092555 | IFIT1/CCL5 | 2 |
| GO:0008643 | BP | carbohydrate transport | 2/27 | 148/17910 | 0.020797 | 0.123627 | 0.092555 | SLC2A3/GRB10 | 2 |
| GO:0010942 | BP | positive regulation of cell death | 4/27 | 710/17910 | 0.020806 | 0.123627 | 0.092555 | GADD45A/HPGD/CCL5/C1QA | 4 |
| GO:0002834 | BP | regulation of response to tumor cell | 1/27 | 14/17910 | 0.020907 | 0.123627 | 0.092555 | CEACAM1 | 1 |
| GO:0002837 | BP | regulation of immune response to tumor cell | 1/27 | 14/17910 | 0.020907 | 0.123627 | 0.092555 | CEACAM1 | 1 |
| GO:0010820 | BP | positive regulation of T cell chemotaxis | 1/27 | 14/17910 | 0.020907 | 0.123627 | 0.092555 | CCL5 | 1 |
| GO:0032688 | BP | negative regulation of interferon-beta production | 1/27 | 14/17910 | 0.020907 | 0.123627 | 0.092555 | NLRC3 | 1 |
| GO:0042976 | BP | activation of Janus kinase activity | 1/27 | 14/17910 | 0.020907 | 0.123627 | 0.092555 | CCL5 | 1 |
| GO:0044546 | BP | NLRP3 inflammasome complex assembly | 1/27 | 14/17910 | 0.020907 | 0.123627 | 0.092555 | NLRC3 | 1 |
| GO:0045953 | BP | negative regulation of natural killer cell mediated cytotoxicity | 1/27 | 14/17910 | 0.020907 | 0.123627 | 0.092555 | CEACAM1 | 1 |
| GO:0046133 | BP | pyrimidine ribonucleoside catabolic process | 1/27 | 14/17910 | 0.020907 | 0.123627 | 0.092555 | APOBEC3B | 1 |
| GO:0070234 | BP | positive regulation of T cell apoptotic process | 1/27 | 14/17910 | 0.020907 | 0.123627 | 0.092555 | CCL5 | 1 |
| GO:0072677 | BP | eosinophil migration | 1/27 | 14/17910 | 0.020907 | 0.123627 | 0.092555 | CCL5 | 1 |
| GO:1903054 | BP | negative regulation of extracellular matrix organization | 1/27 | 14/17910 | 0.020907 | 0.123627 | 0.092555 | DPP4 | 1 |
| GO:2000345 | BP | regulation of hepatocyte proliferation | 1/27 | 14/17910 | 0.020907 | 0.123627 | 0.092555 | CEACAM1 | 1 |
| GO:2001212 | BP | regulation of vasculogenesis | 1/27 | 14/17910 | 0.020907 | 0.123627 | 0.092555 | CEACAM1 | 1 |
| GO:0002684 | BP | positive regulation of immune system process | 5/27 | 1087/17910 | 0.021561 | 0.126987 | 0.095071 | DPP4/VNN1/CCL5/C1QA/CEACAM1 | 5 |
| GO:0002716 | BP | negative regulation of natural killer cell mediated immunity | 1/27 | 15/17910 | 0.022385 | 0.128361 | 0.096099 | CEACAM1 | 1 |
| GO:0010759 | BP | positive regulation of macrophage chemotaxis | 1/27 | 15/17910 | 0.022385 | 0.128361 | 0.096099 | CCL5 | 1 |
| GO:0010819 | BP | regulation of T cell chemotaxis | 1/27 | 15/17910 | 0.022385 | 0.128361 | 0.096099 | CCL5 | 1 |
| GO:0030852 | BP | regulation of granulocyte differentiation | 1/27 | 15/17910 | 0.022385 | 0.128361 | 0.096099 | CEACAM1 | 1 |
| GO:0034111 | BP | negative regulation of homotypic cell-cell adhesion | 1/27 | 15/17910 | 0.022385 | 0.128361 | 0.096099 | CEACAM1 | 1 |
| GO:0060009 | BP | Sertoli cell development | 1/27 | 15/17910 | 0.022385 | 0.128361 | 0.096099 | RAB13 | 1 |
| GO:0071702 | BP | organic substance transport | 8/27 | 2416/17910 | 0.022394 | 0.128361 | 0.096099 | RPL10A/RPL36/DPP4/IFIT1/SLC2A3/CCL5/GRB10/CEACAM1 | 8 |
| GO:0071695 | BP | anatomical structure maturation | 2/27 | 155/17910 | 0.022672 | 0.129453 | 0.096917 | CDC25B/C1QA | 2 |
| GO:0045859 | BP | regulation of protein kinase activity | 4/27 | 732/17910 | 0.022988 | 0.130761 | 0.097896 | CDC25B/GADD45A/CCL5/CEACAM1 | 4 |
| GO:0042221 | BP | response to chemical | 12/27 | 4520/17910 | 0.023158 | 0.131228 | 0.098245 | TGFBR3/PCOLCE2/RAB13/RPL10A/NR3C2/IFIT1/HPGD/VNN1/CCL5/C1QA/GRB10/CEACAM1 | 12 |
| GO:0007567 | BP | parturition | 1/27 | 16/17910 | 0.02386 | 0.131712 | 0.098608 | HPGD | 1 |
| GO:0014067 | BP | negative regulation of phosphatidylinositol 3-kinase signaling | 1/27 | 16/17910 | 0.02386 | 0.131712 | 0.098608 | NLRC3 | 1 |
| GO:0019372 | BP | lipoxygenase pathway | 1/27 | 16/17910 | 0.02386 | 0.131712 | 0.098608 | HPGD | 1 |
| GO:0032354 | BP | response to follicle-stimulating hormone | 1/27 | 16/17910 | 0.02386 | 0.131712 | 0.098608 | TGFBR3 | 1 |
| GO:0033033 | BP | negative regulation of myeloid cell apoptotic process | 1/27 | 16/17910 | 0.02386 | 0.131712 | 0.098608 | CCL5 | 1 |
| GO:0033262 | BP | regulation of nuclear cell cycle DNA replication | 1/27 | 16/17910 | 0.02386 | 0.131712 | 0.098608 | DACH1 | 1 |
| GO:0046325 | BP | negative regulation of glucose import | 1/27 | 16/17910 | 0.02386 | 0.131712 | 0.098608 | GRB10 | 1 |
| GO:0051302 | BP | regulation of cell division | 2/27 | 162/17910 | 0.024614 | 0.135348 | 0.10133 | CDC25B/PRC1 | 2 |
| GO:0008285 | BP | negative regulation of cell proliferation | 4/27 | 751/17910 | 0.024983 | 0.135348 | 0.10133 | TGFBR3/DACH1/NLRC3/CEACAM1 | 4 |
| GO:0002418 | BP | immune response to tumor cell | 1/27 | 17/17910 | 0.025333 | 0.135348 | 0.10133 | CEACAM1 | 1 |
| GO:0010715 | BP | regulation of extracellular matrix disassembly | 1/27 | 17/17910 | 0.025333 | 0.135348 | 0.10133 | DPP4 | 1 |
| GO:0048711 | BP | positive regulation of astrocyte differentiation | 1/27 | 17/17910 | 0.025333 | 0.135348 | 0.10133 | C1QA | 1 |
| GO:0070233 | BP | negative regulation of T cell apoptotic process | 1/27 | 17/17910 | 0.025333 | 0.135348 | 0.10133 | CCL5 | 1 |
| GO:0071360 | BP | cellular response to exogenous dsRNA | 1/27 | 17/17910 | 0.025333 | 0.135348 | 0.10133 | IFIT1 | 1 |
| GO:0072673 | BP | lamellipodium morphogenesis | 1/27 | 17/17910 | 0.025333 | 0.135348 | 0.10133 | PLEKHO1 | 1 |
| GO:1903980 | BP | positive regulation of microglial cell activation | 1/27 | 17/17910 | 0.025333 | 0.135348 | 0.10133 | C1QA | 1 |
| GO:0048523 | BP | negative regulation of cellular process | 12/27 | 4580/17910 | 0.025603 | 0.136305 | 0.102047 | TGFBR3/GADD45A/RRM2/DPP4/HPGD/APOBEC3B/DACH1/VNN1/NLRC3/CCL5/GRB10/CEACAM1 | 12 |
| GO:0051262 | BP | protein tetramerization | 2/27 | 166/17910 | 0.025753 | 0.136619 | 0.102282 | RRM2/CCL5 | 2 |
| GO:0006612 | BP | protein targeting to membrane | 2/27 | 167/17910 | 0.026041 | 0.136852 | 0.102456 | RPL10A/RPL36 | 2 |
| GO:0048469 | BP | cell maturation | 2/27 | 167/17910 | 0.026041 | 0.136852 | 0.102456 | CDC25B/C1QA | 2 |
| GO:0050796 | BP | regulation of insulin secretion | 2/27 | 169/17910 | 0.026622 | 0.136852 | 0.102456 | DPP4/CCL5 | 2 |
| GO:0001911 | BP | negative regulation of leukocyte mediated cytotoxicity | 1/27 | 18/17910 | 0.026803 | 0.136852 | 0.102456 | CEACAM1 | 1 |
| GO:0060977 | BP | coronary vasculature morphogenesis | 1/27 | 18/17910 | 0.026803 | 0.136852 | 0.102456 | TGFBR3 | 1 |
| GO:0070230 | BP | positive regulation of lymphocyte apoptotic process | 1/27 | 18/17910 | 0.026803 | 0.136852 | 0.102456 | CCL5 | 1 |
| GO:0072574 | BP | hepatocyte proliferation | 1/27 | 18/17910 | 0.026803 | 0.136852 | 0.102456 | CEACAM1 | 1 |
| GO:0072575 | BP | epithelial cell proliferation involved in liver morphogenesis | 1/27 | 18/17910 | 0.026803 | 0.136852 | 0.102456 | CEACAM1 | 1 |
| GO:0090026 | BP | positive regulation of monocyte chemotaxis | 1/27 | 18/17910 | 0.026803 | 0.136852 | 0.102456 | CCL5 | 1 |
| GO:0090330 | BP | regulation of platelet aggregation | 1/27 | 18/17910 | 0.026803 | 0.136852 | 0.102456 | CEACAM1 | 1 |
| GO:1902176 | BP | negative regulation of oxidative stress-induced intrinsic apoptotic signaling pathway | 1/27 | 18/17910 | 0.026803 | 0.136852 | 0.102456 | VNN1 | 1 |
| GO:0098609 | BP | cell-cell adhesion | 4/27 | 771/17910 | 0.027194 | 0.138376 | 0.103597 | DPP4/VNN1/CCL5/CEACAM1 | 4 |
| GO:0048660 | BP | regulation of smooth muscle cell proliferation | 2/27 | 172/17910 | 0.027502 | 0.138671 | 0.103818 | HPGD/CCL5 | 2 |
| GO:0045088 | BP | regulation of innate immune response | 3/27 | 440/17910 | 0.027831 | 0.138671 | 0.103818 | NLRC3/CCL5/CEACAM1 | 3 |
| GO:0002697 | BP | regulation of immune effector process | 3/27 | 441/17910 | 0.027994 | 0.138671 | 0.103818 | IFIT1/C1QA/CEACAM1 | 3 |
| GO:0048659 | BP | smooth muscle cell proliferation | 2/27 | 174/17910 | 0.028096 | 0.138671 | 0.103818 | HPGD/CCL5 | 2 |
| GO:0002710 | BP | negative regulation of T cell mediated immunity | 1/27 | 19/17910 | 0.028272 | 0.138671 | 0.103818 | CEACAM1 | 1 |
| GO:0006677 | BP | glycosylceramide metabolic process | 1/27 | 19/17910 | 0.028272 | 0.138671 | 0.103818 | ST6GALNAC3 | 1 |
| GO:0010544 | BP | negative regulation of platelet activation | 1/27 | 19/17910 | 0.028272 | 0.138671 | 0.103818 | CEACAM1 | 1 |
| GO:0030949 | BP | positive regulation of vascular endothelial growth factor receptor signaling pathway | 1/27 | 19/17910 | 0.028272 | 0.138671 | 0.103818 | GRB10 | 1 |
| GO:0050860 | BP | negative regulation of T cell receptor signaling pathway | 1/27 | 19/17910 | 0.028272 | 0.138671 | 0.103818 | CEACAM1 | 1 |
| GO:0072576 | BP | liver morphogenesis | 1/27 | 19/17910 | 0.028272 | 0.138671 | 0.103818 | CEACAM1 | 1 |
| GO:0140131 | BP | positive regulation of lymphocyte chemotaxis | 1/27 | 19/17910 | 0.028272 | 0.138671 | 0.103818 | CCL5 | 1 |
| GO:0006954 | BP | inflammatory response | 4/27 | 784/17910 | 0.028694 | 0.140281 | 0.105023 | VNN1/NLRC3/CCL5/C1QA | 4 |
| GO:0001667 | BP | ameboidal-type cell migration | 3/27 | 446/17910 | 0.028816 | 0.140418 | 0.105125 | RAB13/DPP4/CEACAM1 | 3 |
| GO:0038061 | BP | NIK/NF-kappaB signaling | 2/27 | 179/17910 | 0.029602 | 0.142592 | 0.106753 | NR3C2/NLRC3 | 2 |
| GO:0033630 | BP | positive regulation of cell adhesion mediated by integrin | 1/27 | 20/17910 | 0.029738 | 0.142592 | 0.106753 | CCL5 | 1 |
| GO:0060216 | BP | definitive hemopoiesis | 1/27 | 20/17910 | 0.029738 | 0.142592 | 0.106753 | TGFBR3 | 1 |
| GO:0097503 | BP | sialylation | 1/27 | 20/17910 | 0.029738 | 0.142592 | 0.106753 | ST6GALNAC3 | 1 |
| GO:1905523 | BP | positive regulation of macrophage migration | 1/27 | 20/17910 | 0.029738 | 0.142592 | 0.106753 | CCL5 | 1 |
| GO:0043902 | BP | positive regulation of multi-organism process | 2/27 | 180/17910 | 0.029907 | 0.142943 | 0.107017 | IFIT1/CCL5 | 2 |
| GO:0043901 | BP | negative regulation of multi-organism process | 2/27 | 182/17910 | 0.030521 | 0.144518 | 0.108195 | IFIT1/CCL5 | 2 |
| GO:0048585 | BP | negative regulation of response to stimulus | 6/27 | 1620/17910 | 0.030695 | 0.144518 | 0.108195 | TGFBR3/VNN1/NLRC3/CCL5/GRB10/CEACAM1 | 6 |
| GO:0007179 | BP | transforming growth factor beta receptor signaling pathway | 2/27 | 184/17910 | 0.031141 | 0.144518 | 0.108195 | TGFBR3/HPGD | 2 |
| GO:0000226 | BP | microtubule cytoskeleton organization | 3/27 | 460/17910 | 0.031185 | 0.144518 | 0.108195 | GADD45A/PRC1/HEPACAM2 | 3 |
| GO:0006925 | BP | inflammatory cell apoptotic process | 1/27 | 21/17910 | 0.031203 | 0.144518 | 0.108195 | CCL5 | 1 |
| GO:0010829 | BP | negative regulation of glucose transmembrane transport | 1/27 | 21/17910 | 0.031203 | 0.144518 | 0.108195 | GRB10 | 1 |
| GO:0015721 | BP | bile acid and bile salt transport | 1/27 | 21/17910 | 0.031203 | 0.144518 | 0.108195 | CEACAM1 | 1 |
| GO:0030728 | BP | ovulation | 1/27 | 21/17910 | 0.031203 | 0.144518 | 0.108195 | HPGD | 1 |
| GO:0042454 | BP | ribonucleoside catabolic process | 1/27 | 21/17910 | 0.031203 | 0.144518 | 0.108195 | APOBEC3B | 1 |
| GO:0060008 | BP | Sertoli cell differentiation | 1/27 | 21/17910 | 0.031203 | 0.144518 | 0.108195 | RAB13 | 1 |
| GO:0043549 | BP | regulation of kinase activity | 4/27 | 807/17910 | 0.031468 | 0.145297 | 0.108778 | CDC25B/GADD45A/CCL5/CEACAM1 | 4 |
| GO:1903047 | BP | mitotic cell cycle process | 4/27 | 816/17910 | 0.032596 | 0.146747 | 0.109864 | CDC25B/GADD45A/PRC1/RRM2 | 4 |
| GO:0009251 | BP | glucan catabolic process | 1/27 | 22/17910 | 0.032665 | 0.146747 | 0.109864 | MGAM | 1 |
| GO:0010528 | BP | regulation of transposition | 1/27 | 22/17910 | 0.032665 | 0.146747 | 0.109864 | APOBEC3B | 1 |
| GO:0010529 | BP | negative regulation of transposition | 1/27 | 22/17910 | 0.032665 | 0.146747 | 0.109864 | APOBEC3B | 1 |
| GO:0031342 | BP | negative regulation of cell killing | 1/27 | 22/17910 | 0.032665 | 0.146747 | 0.109864 | CEACAM1 | 1 |
| GO:0034695 | BP | response to prostaglandin E | 1/27 | 22/17910 | 0.032665 | 0.146747 | 0.109864 | TGFBR3 | 1 |
| GO:0071359 | BP | cellular response to dsRNA | 1/27 | 22/17910 | 0.032665 | 0.146747 | 0.109864 | IFIT1 | 1 |
| GO:1900016 | BP | negative regulation of cytokine production involved in inflammatory response | 1/27 | 22/17910 | 0.032665 | 0.146747 | 0.109864 | NLRC3 | 1 |
| GO:1903306 | BP | negative regulation of regulated secretory pathway | 1/27 | 22/17910 | 0.032665 | 0.146747 | 0.109864 | CEACAM1 | 1 |
| GO:0002347 | BP | response to tumor cell | 1/27 | 23/17910 | 0.034125 | 0.151039 | 0.113077 | CEACAM1 | 1 |
| GO:0010758 | BP | regulation of macrophage chemotaxis | 1/27 | 23/17910 | 0.034125 | 0.151039 | 0.113077 | CCL5 | 1 |
| GO:0044247 | BP | cellular polysaccharide catabolic process | 1/27 | 23/17910 | 0.034125 | 0.151039 | 0.113077 | MGAM | 1 |
| GO:0045717 | BP | negative regulation of fatty acid biosynthetic process | 1/27 | 23/17910 | 0.034125 | 0.151039 | 0.113077 | CEACAM1 | 1 |
| GO:0071677 | BP | positive regulation of mononuclear cell migration | 1/27 | 23/17910 | 0.034125 | 0.151039 | 0.113077 | CCL5 | 1 |
| GO:0050727 | BP | regulation of inflammatory response | 3/27 | 479/17910 | 0.034563 | 0.151659 | 0.113541 | NLRC3/CCL5/C1QA | 3 |
| GO:0035556 | BP | intracellular signal transduction | 8/27 | 2624/17910 | 0.035025 | 0.151659 | 0.113541 | TGFBR3/RAB13/GADD45A/NR3C2/VNN1/NLRC3/CCL5/CEACAM1 | 8 |
| GO:0000956 | BP | nuclear-transcribed mRNA catabolic process | 2/27 | 197/17910 | 0.035286 | 0.151659 | 0.113541 | RPL10A/RPL36 | 2 |
| GO:0006810 | BP | transport | 12/27 | 4785/17910 | 0.035484 | 0.151659 | 0.113541 | RAB13/F5/RPL10A/RPL36/DPP4/IFIT1/MGAM/SLC2A3/VNN1/CCL5/GRB10/CEACAM1 | 12 |
| GO:0001914 | BP | regulation of T cell mediated cytotoxicity | 1/27 | 24/17910 | 0.035583 | 0.151659 | 0.113541 | CEACAM1 | 1 |
| GO:0002407 | BP | dendritic cell chemotaxis | 1/27 | 24/17910 | 0.035583 | 0.151659 | 0.113541 | CCL5 | 1 |
| GO:0032897 | BP | negative regulation of viral transcription | 1/27 | 24/17910 | 0.035583 | 0.151659 | 0.113541 | CCL5 | 1 |
| GO:0033081 | BP | regulation of T cell differentiation in thymus | 1/27 | 24/17910 | 0.035583 | 0.151659 | 0.113541 | VNN1 | 1 |
| GO:0046135 | BP | pyrimidine nucleoside catabolic process | 1/27 | 24/17910 | 0.035583 | 0.151659 | 0.113541 | APOBEC3B | 1 |
| GO:0048143 | BP | astrocyte activation | 1/27 | 24/17910 | 0.035583 | 0.151659 | 0.113541 | C1QA | 1 |
| GO:0060343 | BP | trabecula formation | 1/27 | 24/17910 | 0.035583 | 0.151659 | 0.113541 | TGFBR3 | 1 |
| GO:0062009 | BP | secondary palate development | 1/27 | 24/17910 | 0.035583 | 0.151659 | 0.113541 | TGFBR3 | 1 |
| GO:0150078 | BP | positive regulation of neuroinflammatory response | 1/27 | 24/17910 | 0.035583 | 0.151659 | 0.113541 | C1QA | 1 |
| GO:0090276 | BP | regulation of peptide hormone secretion | 2/27 | 199/17910 | 0.035942 | 0.152753 | 0.11436 | DPP4/CCL5 | 2 |
| GO:0030073 | BP | insulin secretion | 2/27 | 200/17910 | 0.036272 | 0.153491 | 0.114913 | DPP4/CCL5 | 2 |
| GO:0000272 | BP | polysaccharide catabolic process | 1/27 | 25/17910 | 0.037039 | 0.153491 | 0.114913 | MGAM | 1 |
| GO:0001556 | BP | oocyte maturation | 1/27 | 25/17910 | 0.037039 | 0.153491 | 0.114913 | CDC25B | 1 |
| GO:0010818 | BP | T cell chemotaxis | 1/27 | 25/17910 | 0.037039 | 0.153491 | 0.114913 | CCL5 | 1 |
| GO:0035640 | BP | exploration behavior | 1/27 | 25/17910 | 0.037039 | 0.153491 | 0.114913 | DPP4 | 1 |
| GO:0042759 | BP | long-chain fatty acid biosynthetic process | 1/27 | 25/17910 | 0.037039 | 0.153491 | 0.114913 | HPGD | 1 |
| GO:0045948 | BP | positive regulation of translational initiation | 1/27 | 25/17910 | 0.037039 | 0.153491 | 0.114913 | CCL5 | 1 |
| GO:0090025 | BP | regulation of monocyte chemotaxis | 1/27 | 25/17910 | 0.037039 | 0.153491 | 0.114913 | CCL5 | 1 |
| GO:1901623 | BP | regulation of lymphocyte chemotaxis | 1/27 | 25/17910 | 0.037039 | 0.153491 | 0.114913 | CCL5 | 1 |
| GO:0050792 | BP | regulation of viral process | 2/27 | 204/17910 | 0.037603 | 0.155396 | 0.116339 | IFIT1/CCL5 | 2 |
| GO:0009636 | BP | response to toxic substance | 3/27 | 499/17910 | 0.038318 | 0.157336 | 0.117791 | RPL10A/HPGD/CCL5 | 3 |
| GO:0032196 | BP | transposition | 1/27 | 26/17910 | 0.038493 | 0.157336 | 0.117791 | APOBEC3B | 1 |
| GO:0032647 | BP | regulation of interferon-alpha production | 1/27 | 26/17910 | 0.038493 | 0.157336 | 0.117791 | NLRC3 | 1 |
| GO:1902175 | BP | regulation of oxidative stress-induced intrinsic apoptotic signaling pathway | 1/27 | 26/17910 | 0.038493 | 0.157336 | 0.117791 | VNN1 | 1 |
| GO:0045860 | BP | positive regulation of protein kinase activity | 3/27 | 501/17910 | 0.038704 | 0.157771 | 0.118117 | CDC25B/GADD45A/CCL5 | 3 |
| GO:0015949 | BP | nucleobase-containing small molecule interconversion | 1/27 | 27/17910 | 0.039944 | 0.160336 | 0.120038 | RRM2 | 1 |
| GO:0036336 | BP | dendritic cell migration | 1/27 | 27/17910 | 0.039944 | 0.160336 | 0.120038 | CCL5 | 1 |
| GO:0043921 | BP | modulation by host of viral transcription | 1/27 | 27/17910 | 0.039944 | 0.160336 | 0.120038 | CCL5 | 1 |
| GO:0050858 | BP | negative regulation of antigen receptor-mediated signaling pathway | 1/27 | 27/17910 | 0.039944 | 0.160336 | 0.120038 | CEACAM1 | 1 |
| GO:0052472 | BP | modulation by host of symbiont transcription | 1/27 | 27/17910 | 0.039944 | 0.160336 | 0.120038 | CCL5 | 1 |
| GO:0051701 | BP | interaction with host | 2/27 | 211/17910 | 0.039977 | 0.160336 | 0.120038 | DPP4/IFIT1 | 2 |
| GO:0014070 | BP | response to organic cyclic compound | 4/27 | 878/17910 | 0.041021 | 0.161684 | 0.121047 | NR3C2/IFIT1/HPGD/CCL5 | 4 |
| GO:0030511 | BP | positive regulation of transforming growth factor beta receptor signaling pathway | 1/27 | 28/17910 | 0.041394 | 0.161684 | 0.121047 | TGFBR3 | 1 |
| GO:0032607 | BP | interferon-alpha production | 1/27 | 28/17910 | 0.041394 | 0.161684 | 0.121047 | NLRC3 | 1 |
| GO:0033032 | BP | regulation of myeloid cell apoptotic process | 1/27 | 28/17910 | 0.041394 | 0.161684 | 0.121047 | CCL5 | 1 |
| GO:0034698 | BP | response to gonadotropin | 1/27 | 28/17910 | 0.041394 | 0.161684 | 0.121047 | TGFBR3 | 1 |
| GO:0052312 | BP | modulation of transcription in other organism involved in symbiotic interaction | 1/27 | 28/17910 | 0.041394 | 0.161684 | 0.121047 | CCL5 | 1 |
| GO:0070229 | BP | negative regulation of lymphocyte apoptotic process | 1/27 | 28/17910 | 0.041394 | 0.161684 | 0.121047 | CCL5 | 1 |
| GO:1903846 | BP | positive regulation of cellular response to transforming growth factor beta stimulus | 1/27 | 28/17910 | 0.041394 | 0.161684 | 0.121047 | TGFBR3 | 1 |
| GO:2000108 | BP | positive regulation of leukocyte apoptotic process | 1/27 | 28/17910 | 0.041394 | 0.161684 | 0.121047 | CCL5 | 1 |
| GO:2000406 | BP | positive regulation of T cell migration | 1/27 | 28/17910 | 0.041394 | 0.161684 | 0.121047 | CCL5 | 1 |
| GO:0033993 | BP | response to lipid | 4/27 | 883/17910 | 0.041751 | 0.162654 | 0.121773 | TGFBR3/NR3C2/HPGD/CCL5 | 4 |
| GO:0043393 | BP | regulation of protein binding | 2/27 | 217/17910 | 0.042057 | 0.16342 | 0.122346 | TGFBR3/IFIT1 | 2 |
| GO:0051234 | BP | establishment of localization | 12/27 | 4908/17910 | 0.042658 | 0.164334 | 0.123031 | RAB13/F5/RPL10A/RPL36/DPP4/IFIT1/MGAM/SLC2A3/VNN1/CCL5/GRB10/CEACAM1 | 12 |
| GO:0000083 | BP | regulation of transcription involved in G1/S transition of mitotic cell cycle | 1/27 | 29/17910 | 0.042841 | 0.164334 | 0.123031 | RRM2 | 1 |
| GO:0009262 | BP | deoxyribonucleotide metabolic process | 1/27 | 29/17910 | 0.042841 | 0.164334 | 0.123031 | RRM2 | 1 |
| GO:0010737 | BP | protein kinase A signaling | 1/27 | 29/17910 | 0.042841 | 0.164334 | 0.123031 | RAB13 | 1 |
| GO:0046627 | BP | negative regulation of insulin receptor signaling pathway | 1/27 | 29/17910 | 0.042841 | 0.164334 | 0.123031 | GRB10 | 1 |
| GO:0002526 | BP | acute inflammatory response | 2/27 | 220/17910 | 0.043112 | 0.164949 | 0.123491 | VNN1/C1QA | 2 |
| GO:0006469 | BP | negative regulation of protein kinase activity | 2/27 | 221/17910 | 0.043466 | 0.165879 | 0.124187 | GADD45A/CEACAM1 | 2 |
| GO:0045137 | BP | development of primary sexual characteristics | 2/27 | 223/17910 | 0.044177 | 0.167304 | 0.125254 | RAB13/DACH1 | 2 |
| GO:0007143 | BP | female meiotic nuclear division | 1/27 | 30/17910 | 0.044286 | 0.167304 | 0.125254 | CDC25B | 1 |
| GO:0010543 | BP | regulation of platelet activation | 1/27 | 30/17910 | 0.044286 | 0.167304 | 0.125254 | CEACAM1 | 1 |
| GO:0034694 | BP | response to prostaglandin | 1/27 | 30/17910 | 0.044286 | 0.167304 | 0.125254 | TGFBR3 | 1 |
| GO:0051338 | BP | regulation of transferase activity | 4/27 | 902/17910 | 0.044592 | 0.167675 | 0.125532 | CDC25B/GADD45A/CCL5/CEACAM1 | 4 |
| GO:0051707 | BP | response to other organism | 4/27 | 903/17910 | 0.044745 | 0.167675 | 0.125532 | IFIT1/HPGD/APOBEC3B/CCL5 | 4 |
| GO:0071407 | BP | cellular response to organic cyclic compound | 3/27 | 532/17910 | 0.044957 | 0.167675 | 0.125532 | NR3C2/IFIT1/CCL5 | 3 |
| GO:0043207 | BP | response to external biotic stimulus | 4/27 | 905/17910 | 0.045051 | 0.167675 | 0.125532 | IFIT1/HPGD/APOBEC3B/CCL5 | 4 |
| GO:0030866 | BP | cortical actin cytoskeleton organization | 1/27 | 31/17910 | 0.04573 | 0.167675 | 0.125532 | RAB13 | 1 |
| GO:0035767 | BP | endothelial cell chemotaxis | 1/27 | 31/17910 | 0.04573 | 0.167675 | 0.125532 | RAB13 | 1 |
| GO:0043330 | BP | response to exogenous dsRNA | 1/27 | 31/17910 | 0.04573 | 0.167675 | 0.125532 | IFIT1 | 1 |
| GO:0044319 | BP | wound healing, spreading of cells | 1/27 | 31/17910 | 0.04573 | 0.167675 | 0.125532 | CEACAM1 | 1 |
| GO:0045920 | BP | negative regulation of exocytosis | 1/27 | 31/17910 | 0.04573 | 0.167675 | 0.125532 | CEACAM1 | 1 |
| GO:0090505 | BP | epiboly involved in wound healing | 1/27 | 31/17910 | 0.04573 | 0.167675 | 0.125532 | CEACAM1 | 1 |
| GO:1900077 | BP | negative regulation of cellular response to insulin stimulus | 1/27 | 31/17910 | 0.04573 | 0.167675 | 0.125532 | GRB10 | 1 |
| GO:1903978 | BP | regulation of microglial cell activation | 1/27 | 31/17910 | 0.04573 | 0.167675 | 0.125532 | C1QA | 1 |
| GO:0033674 | BP | positive regulation of kinase activity | 3/27 | 541/17910 | 0.046863 | 0.168821 | 0.12639 | CDC25B/GADD45A/CCL5 | 3 |
| GO:0051607 | BP | defense response to virus | 2/27 | 231/17910 | 0.047065 | 0.168821 | 0.12639 | IFIT1/APOBEC3B | 2 |
| GO:0009164 | BP | nucleoside catabolic process | 1/27 | 32/17910 | 0.047171 | 0.168821 | 0.12639 | APOBEC3B | 1 |
| GO:0030851 | BP | granulocyte differentiation | 1/27 | 32/17910 | 0.047171 | 0.168821 | 0.12639 | CEACAM1 | 1 |
| GO:0033028 | BP | myeloid cell apoptotic process | 1/27 | 32/17910 | 0.047171 | 0.168821 | 0.12639 | CCL5 | 1 |
| GO:0060317 | BP | cardiac epithelial to mesenchymal transition | 1/27 | 32/17910 | 0.047171 | 0.168821 | 0.12639 | TGFBR3 | 1 |
| GO:0061384 | BP | heart trabecula morphogenesis | 1/27 | 32/17910 | 0.047171 | 0.168821 | 0.12639 | TGFBR3 | 1 |
| GO:0070232 | BP | regulation of T cell apoptotic process | 1/27 | 32/17910 | 0.047171 | 0.168821 | 0.12639 | CCL5 | 1 |
| GO:0090504 | BP | epiboly | 1/27 | 32/17910 | 0.047171 | 0.168821 | 0.12639 | CEACAM1 | 1 |
| GO:1900745 | BP | positive regulation of p38MAPK cascade | 1/27 | 32/17910 | 0.047171 | 0.168821 | 0.12639 | GADD45A | 1 |
| GO:0043903 | BP | regulation of symbiosis, encompassing mutualism through parasitism | 2/27 | 232/17910 | 0.047431 | 0.169348 | 0.126784 | IFIT1/CCL5 | 2 |
| GO:0072657 | BP | protein localization to membrane | 3/27 | 545/17910 | 0.047723 | 0.169847 | 0.127158 | RAB13/RPL10A/RPL36 | 3 |
| GO:0071560 | BP | cellular response to transforming growth factor beta stimulus | 2/27 | 233/17910 | 0.047798 | 0.169847 | 0.127158 | TGFBR3/HPGD | 2 |
| GO:0001913 | BP | T cell mediated cytotoxicity | 1/27 | 33/17910 | 0.04861 | 0.170305 | 0.127501 | CEACAM1 | 1 |
| GO:0010039 | BP | response to iron ion | 1/27 | 33/17910 | 0.04861 | 0.170305 | 0.127501 | C1QA | 1 |
| GO:0030947 | BP | regulation of vascular endothelial growth factor receptor signaling pathway | 1/27 | 33/17910 | 0.04861 | 0.170305 | 0.127501 | GRB10 | 1 |
| GO:0043032 | BP | positive regulation of macrophage activation | 1/27 | 33/17910 | 0.04861 | 0.170305 | 0.127501 | C1QA | 1 |
| GO:0045922 | BP | negative regulation of fatty acid metabolic process | 1/27 | 33/17910 | 0.04861 | 0.170305 | 0.127501 | CEACAM1 | 1 |
| GO:1901223 | BP | negative regulation of NIK/NF-kappaB signaling | 1/27 | 33/17910 | 0.04861 | 0.170305 | 0.127501 | NLRC3 | 1 |
| GO:0006935 | BP | chemotaxis | 3/27 | 551/17910 | 0.049027 | 0.171367 | 0.128296 | RAB13/CCL5/GRB10 | 3 |
| GO:0031348 | BP | negative regulation of defense response | 2/27 | 237/17910 | 0.049276 | 0.171518 | 0.128409 | NLRC3/CEACAM1 | 2 |
| GO:0010243 | BP | response to organonitrogen compound | 4/27 | 932/17910 | 0.0493 | 0.171518 | 0.128409 | TGFBR3/RAB13/GRB10/CEACAM1 | 4 |
| GO:0042330 | BP | taxis | 3/27 | 553/17910 | 0.049466 | 0.171697 | 0.128543 | RAB13/CCL5/GRB10 | 3 |
| GO:0000278 | BP | mitotic cell cycle | 4/27 | 934/17910 | 0.049623 | 0.171844 | 0.128653 | CDC25B/GADD45A/PRC1/RRM2 | 4 |
| GO:0071559 | BP | response to transforming growth factor beta | 2/27 | 239/17910 | 0.050022 | 0.172511 | 0.129152 | TGFBR3/HPGD | 2 |
| GO:2000403 | BP | positive regulation of lymphocyte migration | 1/27 | 34/17910 | 0.050047 | 0.172511 | 0.129152 | CCL5 | 1 |
| GO:0030072 | BP | peptide hormone secretion | 2/27 | 241/17910 | 0.050771 | 0.173999 | 0.130266 | DPP4/CCL5 | 2 |
| GO:0071417 | BP | cellular response to organonitrogen compound | 3/27 | 559/17910 | 0.050794 | 0.173999 | 0.130266 | RAB13/GRB10/CEACAM1 | 3 |
| GO:0030865 | BP | cortical cytoskeleton organization | 1/27 | 35/17910 | 0.051481 | 0.173999 | 0.130266 | RAB13 | 1 |
| GO:0046131 | BP | pyrimidine ribonucleoside metabolic process | 1/27 | 35/17910 | 0.051481 | 0.173999 | 0.130266 | APOBEC3B | 1 |
| GO:0048246 | BP | macrophage chemotaxis | 1/27 | 35/17910 | 0.051481 | 0.173999 | 0.130266 | CCL5 | 1 |
| GO:0048710 | BP | regulation of astrocyte differentiation | 1/27 | 35/17910 | 0.051481 | 0.173999 | 0.130266 | C1QA | 1 |
| GO:0072529 | BP | pyrimidine-containing compound catabolic process | 1/27 | 35/17910 | 0.051481 | 0.173999 | 0.130266 | APOBEC3B | 1 |
| GO:0110111 | BP | negative regulation of animal organ morphogenesis | 1/27 | 35/17910 | 0.051481 | 0.173999 | 0.130266 | CEACAM1 | 1 |
| GO:0033673 | BP | negative regulation of kinase activity | 2/27 | 243/17910 | 0.051525 | 0.173999 | 0.130266 | GADD45A/CEACAM1 | 2 |
| GO:0048584 | BP | positive regulation of response to stimulus | 7/27 | 2322/17910 | 0.051906 | 0.17489 | 0.130934 | TGFBR3/GADD45A/NR3C2/CCL5/C1QA/GRB10/CEACAM1 | 7 |
| GO:0044772 | BP | mitotic cell cycle phase transition | 3/27 | 565/17910 | 0.05214 | 0.175095 | 0.131087 | CDC25B/GADD45A/RRM2 | 3 |
| GO:1902531 | BP | regulation of intracellular signal transduction | 6/27 | 1838/17910 | 0.052201 | 0.175095 | 0.131087 | GADD45A/NR3C2/VNN1/NLRC3/CCL5/CEACAM1 | 6 |
| GO:0043114 | BP | regulation of vascular permeability | 1/27 | 36/17910 | 0.052914 | 0.176695 | 0.132285 | CEACAM1 | 1 |
| GO:1905521 | BP | regulation of macrophage migration | 1/27 | 36/17910 | 0.052914 | 0.176695 | 0.132285 | CCL5 | 1 |
| GO:0001662 | BP | behavioral fear response | 1/27 | 37/17910 | 0.054345 | 0.180524 | 0.135152 | DPP4 | 1 |
| GO:0032467 | BP | positive regulation of cytokinesis | 1/27 | 37/17910 | 0.054345 | 0.180524 | 0.135152 | CDC25B | 1 |
| GO:0051301 | BP | cell division | 3/27 | 575/17910 | 0.054423 | 0.180524 | 0.135152 | CDC25B/PRC1/HEPACAM2 | 3 |
| GO:0007167 | BP | enzyme linked receptor protein signaling pathway | 4/27 | 968/17910 | 0.055307 | 0.180991 | 0.135501 | TGFBR3/HPGD/GRB10/CEACAM1 | 4 |
| GO:0060326 | BP | cell chemotaxis | 2/27 | 253/17910 | 0.055354 | 0.180991 | 0.135501 | RAB13/CCL5 | 2 |
| GO:0046883 | BP | regulation of hormone secretion | 2/27 | 254/17910 | 0.055743 | 0.180991 | 0.135501 | DPP4/CCL5 | 2 |
| GO:0002209 | BP | behavioral defense response | 1/27 | 38/17910 | 0.055773 | 0.180991 | 0.135501 | DPP4 | 1 |
| GO:0002707 | BP | negative regulation of lymphocyte mediated immunity | 1/27 | 38/17910 | 0.055773 | 0.180991 | 0.135501 | CEACAM1 | 1 |
| GO:0007520 | BP | myoblast fusion | 1/27 | 38/17910 | 0.055773 | 0.180991 | 0.135501 | PLEKHO1 | 1 |
| GO:0043331 | BP | response to dsRNA | 1/27 | 38/17910 | 0.055773 | 0.180991 | 0.135501 | IFIT1 | 1 |
| GO:0050856 | BP | regulation of T cell receptor signaling pathway | 1/27 | 38/17910 | 0.055773 | 0.180991 | 0.135501 | CEACAM1 | 1 |
| GO:1901658 | BP | glycosyl compound catabolic process | 1/27 | 38/17910 | 0.055773 | 0.180991 | 0.135501 | APOBEC3B | 1 |
| GO:2000279 | BP | negative regulation of DNA biosynthetic process | 1/27 | 38/17910 | 0.055773 | 0.180991 | 0.135501 | DACH1 | 1 |
| GO:0042269 | BP | regulation of natural killer cell mediated cytotoxicity | 1/27 | 39/17910 | 0.0572 | 0.184023 | 0.137772 | CEACAM1 | 1 |
| GO:0042596 | BP | fear response | 1/27 | 39/17910 | 0.0572 | 0.184023 | 0.137772 | DPP4 | 1 |
| GO:0044275 | BP | cellular carbohydrate catabolic process | 1/27 | 39/17910 | 0.0572 | 0.184023 | 0.137772 | MGAM | 1 |
| GO:0060045 | BP | positive regulation of cardiac muscle cell proliferation | 1/27 | 39/17910 | 0.0572 | 0.184023 | 0.137772 | TGFBR3 | 1 |
| GO:0006260 | BP | DNA replication | 2/27 | 259/17910 | 0.0577 | 0.185233 | 0.138677 | RRM2/DACH1 | 2 |
| GO:0002823 | BP | negative regulation of adaptive immune response based on somatic recombination of immune receptors built from immunoglobulin superfamily domains | 1/27 | 40/17910 | 0.058624 | 0.187397 | 0.140297 | CEACAM1 | 1 |
| GO:0032692 | BP | negative regulation of interleukin-1 production | 1/27 | 40/17910 | 0.058624 | 0.187397 | 0.140297 | CEACAM1 | 1 |
| GO:0021700 | BP | developmental maturation | 2/27 | 262/17910 | 0.058885 | 0.187452 | 0.140338 | CDC25B/C1QA | 2 |
| GO:0032787 | BP | monocarboxylic acid metabolic process | 3/27 | 594/17910 | 0.058892 | 0.187452 | 0.140338 | HPGD/VNN1/CEACAM1 | 3 |
| GO:0034655 | BP | nucleobase-containing compound catabolic process | 3/27 | 598/17910 | 0.059855 | 0.187536 | 0.140401 | RPL10A/RPL36/APOBEC3B | 3 |
| GO:0006692 | BP | prostanoid metabolic process | 1/27 | 41/17910 | 0.060047 | 0.187536 | 0.140401 | HPGD | 1 |
| GO:0006693 | BP | prostaglandin metabolic process | 1/27 | 41/17910 | 0.060047 | 0.187536 | 0.140401 | HPGD | 1 |
| GO:0008631 | BP | intrinsic apoptotic signaling pathway in response to oxidative stress | 1/27 | 41/17910 | 0.060047 | 0.187536 | 0.140401 | VNN1 | 1 |
| GO:0014002 | BP | astrocyte development | 1/27 | 41/17910 | 0.060047 | 0.187536 | 0.140401 | C1QA | 1 |
| GO:0045601 | BP | regulation of endothelial cell differentiation | 1/27 | 41/17910 | 0.060047 | 0.187536 | 0.140401 | CEACAM1 | 1 |
| GO:0070317 | BP | negative regulation of G0 to G1 transition | 1/27 | 41/17910 | 0.060047 | 0.187536 | 0.140401 | RRM2 | 1 |
| GO:1903053 | BP | regulation of extracellular matrix organization | 1/27 | 41/17910 | 0.060047 | 0.187536 | 0.140401 | DPP4 | 1 |
| GO:2000404 | BP | regulation of T cell migration | 1/27 | 41/17910 | 0.060047 | 0.187536 | 0.140401 | CCL5 | 1 |
| GO:1902105 | BP | regulation of leukocyte differentiation | 2/27 | 267/17910 | 0.060881 | 0.189745 | 0.142055 | VNN1/CEACAM1 | 2 |
| GO:0002715 | BP | regulation of natural killer cell mediated immunity | 1/27 | 42/17910 | 0.061467 | 0.189879 | 0.142156 | CEACAM1 | 1 |
| GO:0009311 | BP | oligosaccharide metabolic process | 1/27 | 42/17910 | 0.061467 | 0.189879 | 0.142156 | MGAM | 1 |
| GO:0032456 | BP | endocytic recycling | 1/27 | 42/17910 | 0.061467 | 0.189879 | 0.142156 | RAB13 | 1 |
| GO:0048247 | BP | lymphocyte chemotaxis | 1/27 | 42/17910 | 0.061467 | 0.189879 | 0.142156 | CCL5 | 1 |
| GO:0000082 | BP | G1/S transition of mitotic cell cycle | 2/27 | 269/17910 | 0.061685 | 0.189879 | 0.142156 | GADD45A/RRM2 | 2 |
| GO:0007548 | BP | sex differentiation | 2/27 | 269/17910 | 0.061685 | 0.189879 | 0.142156 | RAB13/DACH1 | 2 |
| GO:0019752 | BP | carboxylic acid metabolic process | 4/27 | 1006/17910 | 0.062071 | 0.190675 | 0.142752 | HPGD/SLC2A3/VNN1/CEACAM1 | 4 |
| GO:0051348 | BP | negative regulation of transferase activity | 2/27 | 271/17910 | 0.062494 | 0.190944 | 0.142953 | GADD45A/CEACAM1 | 2 |
| GO:0044770 | BP | cell cycle phase transition | 3/27 | 609/17910 | 0.062542 | 0.190944 | 0.142953 | CDC25B/GADD45A/RRM2 | 3 |
| GO:0051347 | BP | positive regulation of transferase activity | 3/27 | 609/17910 | 0.062542 | 0.190944 | 0.142953 | CDC25B/GADD45A/CCL5 | 3 |
| GO:0043300 | BP | regulation of leukocyte degranulation | 1/27 | 43/17910 | 0.062885 | 0.191601 | 0.143444 | CEACAM1 | 1 |
| GO:0045786 | BP | negative regulation of cell cycle | 3/27 | 613/17910 | 0.063533 | 0.193182 | 0.144628 | GADD45A/RRM2/HPGD | 3 |
| GO:0002548 | BP | monocyte chemotaxis | 1/27 | 44/17910 | 0.064301 | 0.194332 | 0.145489 | CCL5 | 1 |
| GO:0048599 | BP | oocyte development | 1/27 | 44/17910 | 0.064301 | 0.194332 | 0.145489 | CDC25B | 1 |
| GO:0071622 | BP | regulation of granulocyte chemotaxis | 1/27 | 44/17910 | 0.064301 | 0.194332 | 0.145489 | CCL5 | 1 |
| GO:0090287 | BP | regulation of cellular response to growth factor stimulus | 2/27 | 276/17910 | 0.064531 | 0.194633 | 0.145715 | TGFBR3/GRB10 | 2 |
| GO:0001974 | BP | blood vessel remodeling | 1/27 | 45/17910 | 0.065715 | 0.195837 | 0.146616 | CEACAM1 | 1 |
| GO:0002820 | BP | negative regulation of adaptive immune response | 1/27 | 45/17910 | 0.065715 | 0.195837 | 0.146616 | CEACAM1 | 1 |
| GO:0042551 | BP | neuron maturation | 1/27 | 45/17910 | 0.065715 | 0.195837 | 0.146616 | C1QA | 1 |
| GO:0060412 | BP | ventricular septum morphogenesis | 1/27 | 45/17910 | 0.065715 | 0.195837 | 0.146616 | TGFBR3 | 1 |
| GO:0070316 | BP | regulation of G0 to G1 transition | 1/27 | 45/17910 | 0.065715 | 0.195837 | 0.146616 | RRM2 | 1 |
| GO:0071675 | BP | regulation of mononuclear cell migration | 1/27 | 45/17910 | 0.065715 | 0.195837 | 0.146616 | CCL5 | 1 |
| GO:0007017 | BP | microtubule-based process | 3/27 | 627/17910 | 0.067061 | 0.19733 | 0.147733 | GADD45A/PRC1/HEPACAM2 | 3 |
| GO:0032480 | BP | negative regulation of type I interferon production | 1/27 | 46/17910 | 0.067127 | 0.19733 | 0.147733 | NLRC3 | 1 |
| GO:0045687 | BP | positive regulation of glial cell differentiation | 1/27 | 46/17910 | 0.067127 | 0.19733 | 0.147733 | C1QA | 1 |
| GO:0070231 | BP | T cell apoptotic process | 1/27 | 46/17910 | 0.067127 | 0.19733 | 0.147733 | CCL5 | 1 |
| GO:1900015 | BP | regulation of cytokine production involved in inflammatory response | 1/27 | 46/17910 | 0.067127 | 0.19733 | 0.147733 | NLRC3 | 1 |
| GO:2000107 | BP | negative regulation of leukocyte apoptotic process | 1/27 | 46/17910 | 0.067127 | 0.19733 | 0.147733 | CCL5 | 1 |
| GO:0050776 | BP | regulation of immune response | 4/27 | 1033/17910 | 0.06714 | 0.19733 | 0.147733 | NLRC3/CCL5/C1QA/CEACAM1 | 4 |
| GO:0090068 | BP | positive regulation of cell cycle process | 2/27 | 283/17910 | 0.067421 | 0.197768 | 0.148061 | CDC25B/GADD45A | 2 |
| GO:0002832 | BP | negative regulation of response to biotic stimulus | 1/27 | 47/17910 | 0.068537 | 0.19832 | 0.148475 | CEACAM1 | 1 |
| GO:0014911 | BP | positive regulation of smooth muscle cell migration | 1/27 | 47/17910 | 0.068537 | 0.19832 | 0.148475 | CCL5 | 1 |
| GO:0033260 | BP | nuclear DNA replication | 1/27 | 47/17910 | 0.068537 | 0.19832 | 0.148475 | DACH1 | 1 |
| GO:0043124 | BP | negative regulation of I-kappaB kinase/NF-kappaB signaling | 1/27 | 47/17910 | 0.068537 | 0.19832 | 0.148475 | NLRC3 | 1 |
| GO:0045023 | BP | G0 to G1 transition | 1/27 | 47/17910 | 0.068537 | 0.19832 | 0.148475 | RRM2 | 1 |
| GO:0061383 | BP | trabecula morphogenesis | 1/27 | 47/17910 | 0.068537 | 0.19832 | 0.148475 | TGFBR3 | 1 |
| GO:1900744 | BP | regulation of p38MAPK cascade | 1/27 | 47/17910 | 0.068537 | 0.19832 | 0.148475 | GADD45A | 1 |
| GO:0090150 | BP | establishment of protein localization to membrane | 2/27 | 287/17910 | 0.069092 | 0.198554 | 0.14865 | RPL10A/RPL36 | 2 |
| GO:0044843 | BP | cell cycle G1/S phase transition | 2/27 | 288/17910 | 0.069512 | 0.198554 | 0.14865 | GADD45A/RRM2 | 2 |
| GO:0051270 | BP | regulation of cellular component movement | 4/27 | 1046/17910 | 0.069657 | 0.198554 | 0.14865 | TGFBR3/DACH1/CCL5/CEACAM1 | 4 |
| GO:0002704 | BP | negative regulation of leukocyte mediated immunity | 1/27 | 48/17910 | 0.069945 | 0.198554 | 0.14865 | CEACAM1 | 1 |
| GO:0002762 | BP | negative regulation of myeloid leukocyte differentiation | 1/27 | 48/17910 | 0.069945 | 0.198554 | 0.14865 | CEACAM1 | 1 |
| GO:0032648 | BP | regulation of interferon-beta production | 1/27 | 48/17910 | 0.069945 | 0.198554 | 0.14865 | NLRC3 | 1 |
| GO:0034656 | BP | nucleobase-containing small molecule catabolic process | 1/27 | 48/17910 | 0.069945 | 0.198554 | 0.14865 | APOBEC3B | 1 |
| GO:0055010 | BP | ventricular cardiac muscle tissue morphogenesis | 1/27 | 48/17910 | 0.069945 | 0.198554 | 0.14865 | TGFBR3 | 1 |
| GO:0060389 | BP | pathway-restricted SMAD protein phosphorylation | 1/27 | 48/17910 | 0.069945 | 0.198554 | 0.14865 | TGFBR3 | 1 |
| GO:0090329 | BP | regulation of DNA-dependent DNA replication | 1/27 | 48/17910 | 0.069945 | 0.198554 | 0.14865 | DACH1 | 1 |
| GO:0001818 | BP | negative regulation of cytokine production | 2/27 | 290/17910 | 0.070354 | 0.199336 | 0.149236 | NLRC3/CEACAM1 | 2 |
| GO:0046700 | BP | heterocycle catabolic process | 3/27 | 641/17910 | 0.070679 | 0.199516 | 0.14937 | RPL10A/RPL36/APOBEC3B | 3 |
| GO:0044270 | BP | cellular nitrogen compound catabolic process | 3/27 | 642/17910 | 0.070941 | 0.199516 | 0.14937 | RPL10A/RPL36/APOBEC3B | 3 |
| GO:0002534 | BP | cytokine production involved in inflammatory response | 1/27 | 49/17910 | 0.071351 | 0.199516 | 0.14937 | NLRC3 | 1 |
| GO:0009994 | BP | oocyte differentiation | 1/27 | 49/17910 | 0.071351 | 0.199516 | 0.14937 | CDC25B | 1 |
| GO:0030195 | BP | negative regulation of blood coagulation | 1/27 | 49/17910 | 0.071351 | 0.199516 | 0.14937 | CEACAM1 | 1 |
| GO:0055023 | BP | positive regulation of cardiac muscle tissue growth | 1/27 | 49/17910 | 0.071351 | 0.199516 | 0.14937 | TGFBR3 | 1 |
| GO:1904707 | BP | positive regulation of vascular smooth muscle cell proliferation | 1/27 | 49/17910 | 0.071351 | 0.199516 | 0.14937 | HPGD | 1 |
| GO:0002449 | BP | lymphocyte mediated immunity | 2/27 | 293/17910 | 0.071624 | 0.199797 | 0.149581 | C1QA/CEACAM1 | 2 |
| GO:0002460 | BP | adaptive immune response based on somatic recombination of immune receptors built from immunoglobulin superfamily domains | 2/27 | 295/17910 | 0.072475 | 0.199797 | 0.149581 | C1QA/CEACAM1 | 2 |
| GO:0032496 | BP | response to lipopolysaccharide | 2/27 | 295/17910 | 0.072475 | 0.199797 | 0.149581 | HPGD/CCL5 | 2 |
| GO:0001774 | BP | microglial cell activation | 1/27 | 50/17910 | 0.072755 | 0.199797 | 0.149581 | C1QA | 1 |
| GO:0002269 | BP | leukocyte activation involved in inflammatory response | 1/27 | 50/17910 | 0.072755 | 0.199797 | 0.149581 | C1QA | 1 |
| GO:0032608 | BP | interferon-beta production | 1/27 | 50/17910 | 0.072755 | 0.199797 | 0.149581 | NLRC3 | 1 |
| GO:0060976 | BP | coronary vasculature development | 1/27 | 50/17910 | 0.072755 | 0.199797 | 0.149581 | TGFBR3 | 1 |
| GO:1900047 | BP | negative regulation of hemostasis | 1/27 | 50/17910 | 0.072755 | 0.199797 | 0.149581 | CEACAM1 | 1 |
| GO:1905517 | BP | macrophage migration | 1/27 | 50/17910 | 0.072755 | 0.199797 | 0.149581 | CCL5 | 1 |
| GO:0003006 | BP | developmental process involved in reproduction | 3/27 | 649/17910 | 0.072787 | 0.199797 | 0.149581 | CDC25B/RAB13/DACH1 | 3 |
| GO:0043065 | BP | positive regulation of apoptotic process | 3/27 | 652/17910 | 0.073585 | 0.200871 | 0.150385 | GADD45A/HPGD/CCL5 | 3 |
| GO:1901701 | BP | cellular response to oxygen-containing compound | 4/27 | 1068/17910 | 0.07403 | 0.200871 | 0.150385 | RAB13/CCL5/GRB10/CEACAM1 | 4 |
| GO:0006213 | BP | pyrimidine nucleoside metabolic process | 1/27 | 51/17910 | 0.074157 | 0.200871 | 0.150385 | APOBEC3B | 1 |
| GO:0045744 | BP | negative regulation of G protein-coupled receptor signaling pathway | 1/27 | 51/17910 | 0.074157 | 0.200871 | 0.150385 | CCL5 | 1 |
| GO:0070098 | BP | chemokine-mediated signaling pathway | 1/27 | 51/17910 | 0.074157 | 0.200871 | 0.150385 | CCL5 | 1 |
| GO:0070228 | BP | regulation of lymphocyte apoptotic process | 1/27 | 51/17910 | 0.074157 | 0.200871 | 0.150385 | CCL5 | 1 |
| GO:0150077 | BP | regulation of neuroinflammatory response | 1/27 | 51/17910 | 0.074157 | 0.200871 | 0.150385 | C1QA | 1 |
| GO:0010646 | BP | regulation of cell communication | 9/27 | 3589/17910 | 0.074264 | 0.200871 | 0.150385 | TGFBR3/GADD45A/DPP4/NR3C2/VNN1/NLRC3/CCL5/GRB10/CEACAM1 | 9 |
| GO:0019439 | BP | aromatic compound catabolic process | 3/27 | 655/17910 | 0.074387 | 0.200871 | 0.150385 | RPL10A/RPL36/APOBEC3B | 3 |
| GO:0044281 | BP | small molecule metabolic process | 6/27 | 2012/17910 | 0.074918 | 0.201927 | 0.151175 | RRM2/HPGD/SLC2A3/APOBEC3B/VNN1/CEACAM1 | 6 |
| GO:0046879 | BP | hormone secretion | 2/27 | 301/17910 | 0.075048 | 0.201927 | 0.151175 | DPP4/CCL5 | 2 |
| GO:0043068 | BP | positive regulation of programmed cell death | 3/27 | 660/17910 | 0.075732 | 0.203398 | 0.152276 | GADD45A/HPGD/CCL5 | 3 |
| GO:0032147 | BP | activation of protein kinase activity | 2/27 | 303/17910 | 0.075912 | 0.203398 | 0.152276 | GADD45A/CCL5 | 2 |
| GO:0065009 | BP | regulation of molecular function | 8/27 | 3065/17910 | 0.07684 | 0.203398 | 0.152276 | CDC25B/TGFBR3/PCOLCE2/GADD45A/IFIT1/NLRC3/CCL5/CEACAM1 | 8 |
| GO:0000768 | BP | syncytium formation by plasma membrane fusion | 1/27 | 53/17910 | 0.076954 | 0.203398 | 0.152276 | PLEKHO1 | 1 |
| GO:0038066 | BP | p38MAPK cascade | 1/27 | 53/17910 | 0.076954 | 0.203398 | 0.152276 | GADD45A | 1 |
| GO:0046456 | BP | icosanoid biosynthetic process | 1/27 | 53/17910 | 0.076954 | 0.203398 | 0.152276 | HPGD | 1 |
| GO:0050819 | BP | negative regulation of coagulation | 1/27 | 53/17910 | 0.076954 | 0.203398 | 0.152276 | CEACAM1 | 1 |
| GO:0051290 | BP | protein heterotetramerization | 1/27 | 53/17910 | 0.076954 | 0.203398 | 0.152276 | RRM2 | 1 |
| GO:0060421 | BP | positive regulation of heart growth | 1/27 | 53/17910 | 0.076954 | 0.203398 | 0.152276 | TGFBR3 | 1 |
| GO:0140253 | BP | cell-cell fusion | 1/27 | 53/17910 | 0.076954 | 0.203398 | 0.152276 | PLEKHO1 | 1 |
| GO:0071345 | BP | cellular response to cytokine stimulus | 4/27 | 1084/17910 | 0.0773 | 0.203944 | 0.152686 | PCOLCE2/IFIT1/CCL5/CEACAM1 | 4 |
| GO:0006412 | BP | translation | 3/27 | 668/17910 | 0.077907 | 0.203944 | 0.152686 | RPL10A/RPL36/CCL5 | 3 |
| GO:0046907 | BP | intracellular transport | 5/27 | 1546/17910 | 0.078078 | 0.203944 | 0.152686 | RAB13/F5/RPL10A/RPL36/IFIT1 | 5 |
| GO:0002011 | BP | morphogenesis of an epithelial sheet | 1/27 | 54/17910 | 0.07835 | 0.203944 | 0.152686 | CEACAM1 | 1 |
| GO:0006636 | BP | unsaturated fatty acid biosynthetic process | 1/27 | 54/17910 | 0.07835 | 0.203944 | 0.152686 | HPGD | 1 |
| GO:0032715 | BP | negative regulation of interleukin-6 production | 1/27 | 54/17910 | 0.07835 | 0.203944 | 0.152686 | NLRC3 | 1 |
| GO:0042304 | BP | regulation of fatty acid biosynthetic process | 1/27 | 54/17910 | 0.07835 | 0.203944 | 0.152686 | CEACAM1 | 1 |
| GO:0070830 | BP | bicellular tight junction assembly | 1/27 | 54/17910 | 0.07835 | 0.203944 | 0.152686 | RAB13 | 1 |
| GO:0048514 | BP | blood vessel morphogenesis | 3/27 | 670/17910 | 0.078456 | 0.203944 | 0.152686 | TGFBR3/HPGD/CEACAM1 | 3 |
| GO:0002237 | BP | response to molecule of bacterial origin | 2/27 | 309/17910 | 0.078524 | 0.203944 | 0.152686 | HPGD/CCL5 | 2 |
| GO:0043436 | BP | oxoacid metabolic process | 4/27 | 1091/17910 | 0.078754 | 0.204072 | 0.152781 | HPGD/SLC2A3/VNN1/CEACAM1 | 4 |
| GO:0009914 | BP | hormone transport | 2/27 | 311/17910 | 0.079401 | 0.204072 | 0.152781 | DPP4/CCL5 | 2 |
| GO:0023051 | BP | regulation of signaling | 9/27 | 3635/17910 | 0.079404 | 0.204072 | 0.152781 | TGFBR3/GADD45A/DPP4/NR3C2/VNN1/NLRC3/CCL5/GRB10/CEACAM1 | 9 |
| GO:0003229 | BP | ventricular cardiac muscle tissue development | 1/27 | 55/17910 | 0.079743 | 0.204072 | 0.152781 | TGFBR3 | 1 |
| GO:0006949 | BP | syncytium formation | 1/27 | 55/17910 | 0.079743 | 0.204072 | 0.152781 | PLEKHO1 | 1 |
| GO:0031295 | BP | T cell costimulation | 1/27 | 55/17910 | 0.079743 | 0.204072 | 0.152781 | DPP4 | 1 |
| GO:0031663 | BP | lipopolysaccharide-mediated signaling pathway | 1/27 | 55/17910 | 0.079743 | 0.204072 | 0.152781 | CCL5 | 1 |
| GO:0120192 | BP | tight junction assembly | 1/27 | 55/17910 | 0.079743 | 0.204072 | 0.152781 | RAB13 | 1 |
| GO:0044092 | BP | negative regulation of molecular function | 4/27 | 1096/17910 | 0.079801 | 0.204072 | 0.152781 | GADD45A/IFIT1/NLRC3/CEACAM1 | 4 |
| GO:1901214 | BP | regulation of neuron death | 2/27 | 313/17910 | 0.080281 | 0.204951 | 0.153439 | CCL5/C1QA | 2 |
| GO:0006977 | BP | DNA damage response, signal transduction by p53 class mediator resulting in cell cycle arrest | 1/27 | 56/17910 | 0.081135 | 0.205453 | 0.153815 | GADD45A | 1 |
| GO:0031294 | BP | lymphocyte costimulation | 1/27 | 56/17910 | 0.081135 | 0.205453 | 0.153815 | DPP4 | 1 |
| GO:0061098 | BP | positive regulation of protein tyrosine kinase activity | 1/27 | 56/17910 | 0.081135 | 0.205453 | 0.153815 | CCL5 | 1 |
| GO:1903202 | BP | negative regulation of oxidative stress-induced cell death | 1/27 | 56/17910 | 0.081135 | 0.205453 | 0.153815 | VNN1 | 1 |
| GO:0032103 | BP | positive regulation of response to external stimulus | 2/27 | 315/17910 | 0.081165 | 0.205453 | 0.153815 | CCL5/C1QA | 2 |
| GO:0072330 | BP | monocarboxylic acid biosynthetic process | 2/27 | 318/17910 | 0.082496 | 0.206008 | 0.154231 | HPGD/CEACAM1 | 2 |
| GO:0010518 | BP | positive regulation of phospholipase activity | 1/27 | 57/17910 | 0.082524 | 0.206008 | 0.154231 | CCL5 | 1 |
| GO:0044786 | BP | cell cycle DNA replication | 1/27 | 57/17910 | 0.082524 | 0.206008 | 0.154231 | DACH1 | 1 |
| GO:0051055 | BP | negative regulation of lipid biosynthetic process | 1/27 | 57/17910 | 0.082524 | 0.206008 | 0.154231 | CEACAM1 | 1 |
| GO:0072431 | BP | signal transduction involved in mitotic G1 DNA damage checkpoint | 1/27 | 57/17910 | 0.082524 | 0.206008 | 0.154231 | GADD45A | 1 |
| GO:1900408 | BP | negative regulation of cellular response to oxidative stress | 1/27 | 57/17910 | 0.082524 | 0.206008 | 0.154231 | VNN1 | 1 |
| GO:1902400 | BP | intracellular signal transduction involved in G1 DNA damage checkpoint | 1/27 | 57/17910 | 0.082524 | 0.206008 | 0.154231 | GADD45A | 1 |
| GO:0006082 | BP | organic acid metabolic process | 4/27 | 1109/17910 | 0.082557 | 0.206008 | 0.154231 | HPGD/SLC2A3/VNN1/CEACAM1 | 4 |
| GO:1901361 | BP | organic cyclic compound catabolic process | 3/27 | 685/17910 | 0.082623 | 0.206008 | 0.154231 | RPL10A/RPL36/APOBEC3B | 3 |
| GO:0046626 | BP | regulation of insulin receptor signaling pathway | 1/27 | 58/17910 | 0.083912 | 0.208165 | 0.155845 | GRB10 | 1 |
| GO:0060043 | BP | regulation of cardiac muscle cell proliferation | 1/27 | 58/17910 | 0.083912 | 0.208165 | 0.155845 | TGFBR3 | 1 |
| GO:0070527 | BP | platelet aggregation | 1/27 | 58/17910 | 0.083912 | 0.208165 | 0.155845 | CEACAM1 | 1 |
| GO:0007178 | BP | transmembrane receptor protein serine/threonine kinase signaling pathway | 2/27 | 323/17910 | 0.084729 | 0.208165 | 0.155845 | TGFBR3/HPGD | 2 |
| GO:0043043 | BP | peptide biosynthetic process | 3/27 | 693/17910 | 0.084886 | 0.208165 | 0.155845 | RPL10A/RPL36/CCL5 | 3 |
| GO:0042267 | BP | natural killer cell mediated cytotoxicity | 1/27 | 59/17910 | 0.085297 | 0.208165 | 0.155845 | CEACAM1 | 1 |
| GO:0061900 | BP | glial cell activation | 1/27 | 59/17910 | 0.085297 | 0.208165 | 0.155845 | C1QA | 1 |
| GO:0072413 | BP | signal transduction involved in mitotic cell cycle checkpoint | 1/27 | 59/17910 | 0.085297 | 0.208165 | 0.155845 | GADD45A | 1 |
| GO:0072678 | BP | T cell migration | 1/27 | 59/17910 | 0.085297 | 0.208165 | 0.155845 | CCL5 | 1 |
| GO:0120193 | BP | tight junction organization | 1/27 | 59/17910 | 0.085297 | 0.208165 | 0.155845 | RAB13 | 1 |
| GO:1902402 | BP | signal transduction involved in mitotic DNA damage checkpoint | 1/27 | 59/17910 | 0.085297 | 0.208165 | 0.155845 | GADD45A | 1 |
| GO:1902403 | BP | signal transduction involved in mitotic DNA integrity checkpoint | 1/27 | 59/17910 | 0.085297 | 0.208165 | 0.155845 | GADD45A | 1 |
| GO:1902883 | BP | negative regulation of response to oxidative stress | 1/27 | 59/17910 | 0.085297 | 0.208165 | 0.155845 | VNN1 | 1 |
| GO:0007010 | BP | cytoskeleton organization | 4/27 | 1124/17910 | 0.085797 | 0.209044 | 0.156503 | RAB13/GADD45A/PRC1/HEPACAM2 | 4 |
| GO:0009967 | BP | positive regulation of signal transduction | 5/27 | 1591/17910 | 0.086019 | 0.209243 | 0.156652 | TGFBR3/GADD45A/NR3C2/CCL5/GRB10 | 5 |
| GO:0046324 | BP | regulation of glucose import | 1/27 | 60/17910 | 0.086681 | 0.209491 | 0.156838 | GRB10 | 1 |
| GO:1990868 | BP | response to chemokine | 1/27 | 60/17910 | 0.086681 | 0.209491 | 0.156838 | CCL5 | 1 |
| GO:1990869 | BP | cellular response to chemokine | 1/27 | 60/17910 | 0.086681 | 0.209491 | 0.156838 | CCL5 | 1 |
| GO:2000401 | BP | regulation of lymphocyte migration | 1/27 | 60/17910 | 0.086681 | 0.209491 | 0.156838 | CCL5 | 1 |
| GO:0046677 | BP | response to antibiotic | 2/27 | 328/17910 | 0.086982 | 0.209879 | 0.157129 | RPL10A/HPGD | 2 |
| GO:1903530 | BP | regulation of secretion by cell | 3/27 | 703/17910 | 0.087752 | 0.211163 | 0.15809 | DPP4/CCL5/CEACAM1 | 3 |
| GO:0048583 | BP | regulation of response to stimulus | 10/27 | 4275/17910 | 0.087796 | 0.211163 | 0.15809 | TGFBR3/GADD45A/NR3C2/IFIT1/VNN1/NLRC3/CCL5/C1QA/GRB10/CEACAM1 | 10 |
| GO:0002709 | BP | regulation of T cell mediated immunity | 1/27 | 61/17910 | 0.088062 | 0.211463 | 0.158315 | CEACAM1 | 1 |
| GO:0007596 | BP | blood coagulation | 2/27 | 331/17910 | 0.088342 | 0.211795 | 0.158563 | F5/CEACAM1 | 2 |
| GO:0044267 | BP | cellular protein metabolic process | 11/27 | 4871/17910 | 0.089254 | 0.21364 | 0.159944 | CDC25B/TGFBR3/ST6GALNAC3/PCOLCE2/RAB13/F5/GADD45A/RPL10A/RPL36/CCL5/CEACAM1 | 11 |
| GO:0007585 | BP | respiratory gaseous exchange | 1/27 | 62/17910 | 0.089442 | 0.213746 | 0.160024 | DACH1 | 1 |
| GO:0007599 | BP | hemostasis | 2/27 | 336/17910 | 0.090624 | 0.214237 | 0.160391 | F5/CEACAM1 | 2 |
| GO:0002228 | BP | natural killer cell mediated immunity | 1/27 | 63/17910 | 0.090819 | 0.214237 | 0.160391 | CEACAM1 | 1 |
| GO:0031571 | BP | mitotic G1 DNA damage checkpoint | 1/27 | 63/17910 | 0.090819 | 0.214237 | 0.160391 | GADD45A | 1 |
| GO:0043297 | BP | apical junction assembly | 1/27 | 63/17910 | 0.090819 | 0.214237 | 0.160391 | RAB13 | 1 |
| GO:0044819 | BP | mitotic G1/S transition checkpoint | 1/27 | 63/17910 | 0.090819 | 0.214237 | 0.160391 | GADD45A | 1 |
| GO:0050854 | BP | regulation of antigen receptor-mediated signaling pathway | 1/27 | 63/17910 | 0.090819 | 0.214237 | 0.160391 | CEACAM1 | 1 |
| GO:0055008 | BP | cardiac muscle tissue morphogenesis | 1/27 | 63/17910 | 0.090819 | 0.214237 | 0.160391 | TGFBR3 | 1 |
| GO:0055025 | BP | positive regulation of cardiac muscle tissue development | 1/27 | 63/17910 | 0.090819 | 0.214237 | 0.160391 | TGFBR3 | 1 |
| GO:0050790 | BP | regulation of catalytic activity | 6/27 | 2116/17910 | 0.090936 | 0.214237 | 0.160391 | CDC25B/PCOLCE2/GADD45A/IFIT1/CCL5/CEACAM1 | 6 |
| GO:0050817 | BP | coagulation | 2/27 | 337/17910 | 0.091082 | 0.214244 | 0.160397 | F5/CEACAM1 | 2 |
| GO:0006402 | BP | mRNA catabolic process | 2/27 | 338/17910 | 0.091542 | 0.214834 | 0.160838 | RPL10A/RPL36 | 2 |
| GO:0006631 | BP | fatty acid metabolic process | 2/27 | 339/17910 | 0.092002 | 0.214834 | 0.160838 | HPGD/CEACAM1 | 2 |
| GO:0001910 | BP | regulation of leukocyte mediated cytotoxicity | 1/27 | 64/17910 | 0.092195 | 0.214834 | 0.160838 | CEACAM1 | 1 |
| GO:0044783 | BP | G1 DNA damage checkpoint | 1/27 | 64/17910 | 0.092195 | 0.214834 | 0.160838 | GADD45A | 1 |
| GO:0046622 | BP | positive regulation of organ growth | 1/27 | 64/17910 | 0.092195 | 0.214834 | 0.160838 | TGFBR3 | 1 |
| GO:0046782 | BP | regulation of viral transcription | 1/27 | 64/17910 | 0.092195 | 0.214834 | 0.160838 | CCL5 | 1 |
| GO:0006687 | BP | glycosphingolipid metabolic process | 1/27 | 65/17910 | 0.093568 | 0.217021 | 0.162475 | ST6GALNAC3 | 1 |
| GO:0048207 | BP | vesicle targeting, rough ER to cis-Golgi | 1/27 | 65/17910 | 0.093568 | 0.217021 | 0.162475 | F5 | 1 |
| GO:0048208 | BP | COPII vesicle coating | 1/27 | 65/17910 | 0.093568 | 0.217021 | 0.162475 | F5 | 1 |
| GO:0032102 | BP | negative regulation of response to external stimulus | 2/27 | 343/17910 | 0.093848 | 0.217333 | 0.162709 | NLRC3/CEACAM1 | 2 |
| GO:0010948 | BP | negative regulation of cell cycle process | 2/27 | 344/17910 | 0.094312 | 0.218068 | 0.16326 | GADD45A/RRM2 | 2 |
| GO:0010517 | BP | regulation of phospholipase activity | 1/27 | 66/17910 | 0.09494 | 0.218508 | 0.163589 | CCL5 | 1 |
| GO:0043030 | BP | regulation of macrophage activation | 1/27 | 66/17910 | 0.09494 | 0.218508 | 0.163589 | C1QA | 1 |
| GO:1900076 | BP | regulation of cellular response to insulin stimulus | 1/27 | 66/17910 | 0.09494 | 0.218508 | 0.163589 | GRB10 | 1 |
| GO:0034097 | BP | response to cytokine | 4/27 | 1167/17910 | 0.095438 | 0.219298 | 0.16418 | PCOLCE2/IFIT1/CCL5/CEACAM1 | 4 |
| GO:0070997 | BP | neuron death | 2/27 | 347/17910 | 0.095706 | 0.219298 | 0.16418 | CCL5/C1QA | 2 |
| GO:0006901 | BP | vesicle coating | 1/27 | 67/17910 | 0.096309 | 0.219298 | 0.16418 | F5 | 1 |
| GO:0042531 | BP | positive regulation of tyrosine phosphorylation of STAT protein | 1/27 | 67/17910 | 0.096309 | 0.219298 | 0.16418 | CCL5 | 1 |
| GO:0050918 | BP | positive chemotaxis | 1/27 | 67/17910 | 0.096309 | 0.219298 | 0.16418 | CCL5 | 1 |
| GO:0051705 | BP | multi-organism behavior | 1/27 | 67/17910 | 0.096309 | 0.219298 | 0.16418 | DACH1 | 1 |
| GO:0070227 | BP | lymphocyte apoptotic process | 1/27 | 67/17910 | 0.096309 | 0.219298 | 0.16418 | CCL5 | 1 |
| GO:0046323 | BP | glucose import | 1/27 | 68/17910 | 0.097677 | 0.221401 | 0.165754 | GRB10 | 1 |
| GO:0060038 | BP | cardiac muscle cell proliferation | 1/27 | 68/17910 | 0.097677 | 0.221401 | 0.165754 | TGFBR3 | 1 |
| GO:0071674 | BP | mononuclear cell migration | 1/27 | 68/17910 | 0.097677 | 0.221401 | 0.165754 | CCL5 | 1 |
| GO:0010941 | BP | regulation of cell death | 5/27 | 1659/17910 | 0.098829 | 0.222473 | 0.166557 | GADD45A/HPGD/VNN1/CCL5/C1QA | 5 |
| GO:0001666 | BP | response to hypoxia | 2/27 | 354/17910 | 0.098983 | 0.222473 | 0.166557 | TGFBR3/DPP4 | 2 |
| GO:0006446 | BP | regulation of translational initiation | 1/27 | 69/17910 | 0.099042 | 0.222473 | 0.166557 | CCL5 | 1 |
| GO:0032720 | BP | negative regulation of tumor necrosis factor production | 1/27 | 69/17910 | 0.099042 | 0.222473 | 0.166557 | NLRC3 | 1 |
| GO:0033077 | BP | T cell differentiation in thymus | 1/27 | 69/17910 | 0.099042 | 0.222473 | 0.166557 | VNN1 | 1 |
| GO:0071479 | BP | cellular response to ionizing radiation | 1/27 | 69/17910 | 0.099042 | 0.222473 | 0.166557 | GADD45A | 1 |
| GO:0000280 | BP | nuclear division | 2/27 | 357/17910 | 0.100398 | 0.224189 | 0.167842 | CDC25B/PRC1 | 2 |
| GO:0048199 | BP | vesicle targeting, to, from or within Golgi | 1/27 | 70/17910 | 0.100406 | 0.224189 | 0.167842 | F5 | 1 |
| GO:0060193 | BP | positive regulation of lipase activity | 1/27 | 70/17910 | 0.100406 | 0.224189 | 0.167842 | CCL5 | 1 |
| GO:0090114 | BP | COPII-coated vesicle budding | 1/27 | 70/17910 | 0.100406 | 0.224189 | 0.167842 | F5 | 1 |
| GO:0001960 | BP | negative regulation of cytokine-mediated signaling pathway | 1/27 | 71/17910 | 0.101767 | 0.225994 | 0.169193 | CCL5 | 1 |
| GO:0033555 | BP | multicellular organismal response to stress | 1/27 | 71/17910 | 0.101767 | 0.225994 | 0.169193 | DPP4 | 1 |
| GO:1903556 | BP | negative regulation of tumor necrosis factor superfamily cytokine production | 1/27 | 71/17910 | 0.101767 | 0.225994 | 0.169193 | NLRC3 | 1 |
| GO:0043687 | BP | post-translational protein modification | 2/27 | 360/17910 | 0.101818 | 0.225994 | 0.169193 | RAB13/F5 | 2 |
| GO:0001568 | BP | blood vessel development | 3/27 | 751/17910 | 0.102084 | 0.226206 | 0.169352 | TGFBR3/HPGD/CEACAM1 | 3 |
| GO:0002532 | BP | production of molecular mediator involved in inflammatory response | 1/27 | 72/17910 | 0.103127 | 0.226206 | 0.169352 | NLRC3 | 1 |
| GO:0006073 | BP | cellular glucan metabolic process | 1/27 | 72/17910 | 0.103127 | 0.226206 | 0.169352 | MGAM | 1 |
| GO:0044042 | BP | glucan metabolic process | 1/27 | 72/17910 | 0.103127 | 0.226206 | 0.169352 | MGAM | 1 |
| GO:0009966 | BP | regulation of signal transduction | 8/27 | 3265/17910 | 0.103365 | 0.226206 | 0.169352 | TGFBR3/GADD45A/NR3C2/VNN1/NLRC3/CCL5/GRB10/CEACAM1 | 8 |
| GO:0043086 | BP | negative regulation of catalytic activity | 3/27 | 756/17910 | 0.10363 | 0.226206 | 0.169352 | GADD45A/IFIT1/CEACAM1 | 3 |
| GO:0006605 | BP | protein targeting | 2/27 | 364/17910 | 0.103721 | 0.226206 | 0.169352 | RPL10A/RPL36 | 2 |
| GO:0051046 | BP | regulation of secretion | 3/27 | 757/17910 | 0.10394 | 0.226206 | 0.169352 | DPP4/CCL5/CEACAM1 | 3 |
| GO:0036293 | BP | response to decreased oxygen levels | 2/27 | 365/17910 | 0.104198 | 0.226206 | 0.169352 | TGFBR3/DPP4 | 2 |
| GO:0051271 | BP | negative regulation of cellular component movement | 2/27 | 365/17910 | 0.104198 | 0.226206 | 0.169352 | TGFBR3/DACH1 | 2 |
| GO:0003208 | BP | cardiac ventricle morphogenesis | 1/27 | 73/17910 | 0.104484 | 0.226206 | 0.169352 | TGFBR3 | 1 |
| GO:0044766 | BP | multi-organism transport | 1/27 | 73/17910 | 0.104484 | 0.226206 | 0.169352 | IFIT1 | 1 |
| GO:0050688 | BP | regulation of defense response to virus | 1/27 | 73/17910 | 0.104484 | 0.226206 | 0.169352 | IFIT1 | 1 |
| GO:0061045 | BP | negative regulation of wound healing | 1/27 | 73/17910 | 0.104484 | 0.226206 | 0.169352 | CEACAM1 | 1 |
| GO:0072401 | BP | signal transduction involved in DNA integrity checkpoint | 1/27 | 73/17910 | 0.104484 | 0.226206 | 0.169352 | GADD45A | 1 |
| GO:0072422 | BP | signal transduction involved in DNA damage checkpoint | 1/27 | 73/17910 | 0.104484 | 0.226206 | 0.169352 | GADD45A | 1 |
| GO:1902579 | BP | multi-organism localization | 1/27 | 73/17910 | 0.104484 | 0.226206 | 0.169352 | IFIT1 | 1 |
| GO:0006732 | BP | coenzyme metabolic process | 2/27 | 366/17910 | 0.104676 | 0.226206 | 0.169352 | SLC2A3/VNN1 | 2 |
| GO:0015031 | BP | protein transport | 5/27 | 1689/17910 | 0.104787 | 0.226206 | 0.169352 | RPL10A/RPL36/DPP4/IFIT1/CCL5 | 5 |
| GO:0006401 | BP | RNA catabolic process | 2/27 | 368/17910 | 0.105634 | 0.227125 | 0.17004 | RPL10A/RPL36 | 2 |
| GO:0051098 | BP | regulation of binding | 2/27 | 368/17910 | 0.105634 | 0.227125 | 0.17004 | TGFBR3/IFIT1 | 2 |
| GO:0030193 | BP | regulation of blood coagulation | 1/27 | 74/17910 | 0.10584 | 0.227125 | 0.17004 | CEACAM1 | 1 |
| GO:0072395 | BP | signal transduction involved in cell cycle checkpoint | 1/27 | 74/17910 | 0.10584 | 0.227125 | 0.17004 | GADD45A | 1 |
| GO:0033036 | BP | macromolecule localization | 7/27 | 2735/17910 | 0.106008 | 0.227125 | 0.17004 | RAB13/RPL10A/RPL36/DPP4/IFIT1/CCL5/CEACAM1 | 7 |
| GO:0019221 | BP | cytokine-mediated signaling pathway | 3/27 | 764/17910 | 0.106123 | 0.227125 | 0.17004 | IFIT1/CCL5/CEACAM1 | 3 |
| GO:0031100 | BP | animal organ regeneration | 1/27 | 75/17910 | 0.107193 | 0.227786 | 0.170535 | TGFBR3 | 1 |
| GO:0048194 | BP | Golgi vesicle budding | 1/27 | 75/17910 | 0.107193 | 0.227786 | 0.170535 | F5 | 1 |
| GO:1900046 | BP | regulation of hemostasis | 1/27 | 75/17910 | 0.107193 | 0.227786 | 0.170535 | CEACAM1 | 1 |
| GO:1901224 | BP | positive regulation of NIK/NF-kappaB signaling | 1/27 | 75/17910 | 0.107193 | 0.227786 | 0.170535 | NR3C2 | 1 |
| GO:1903201 | BP | regulation of oxidative stress-induced cell death | 1/27 | 75/17910 | 0.107193 | 0.227786 | 0.170535 | VNN1 | 1 |
| GO:0045787 | BP | positive regulation of cell cycle | 2/27 | 373/17910 | 0.108039 | 0.228709 | 0.171226 | CDC25B/GADD45A | 2 |
| GO:0003281 | BP | ventricular septum development | 1/27 | 76/17910 | 0.108545 | 0.228709 | 0.171226 | TGFBR3 | 1 |
| GO:0014015 | BP | positive regulation of gliogenesis | 1/27 | 76/17910 | 0.108545 | 0.228709 | 0.171226 | C1QA | 1 |
| GO:0048844 | BP | artery morphogenesis | 1/27 | 76/17910 | 0.108545 | 0.228709 | 0.171226 | HPGD | 1 |
| GO:0060415 | BP | muscle tissue morphogenesis | 1/27 | 76/17910 | 0.108545 | 0.228709 | 0.171226 | TGFBR3 | 1 |
| GO:0150076 | BP | neuroinflammatory response | 1/27 | 76/17910 | 0.108545 | 0.228709 | 0.171226 | C1QA | 1 |
| GO:0009057 | BP | macromolecule catabolic process | 4/27 | 1226/17910 | 0.109494 | 0.229292 | 0.171662 | RPL10A/RPL36/MGAM/CEACAM1 | 4 |
| GO:0045685 | BP | regulation of glial cell differentiation | 1/27 | 77/17910 | 0.109895 | 0.229292 | 0.171662 | C1QA | 1 |
| GO:0048708 | BP | astrocyte differentiation | 1/27 | 77/17910 | 0.109895 | 0.229292 | 0.171662 | C1QA | 1 |
| GO:0051851 | BP | modification by host of symbiont morphology or physiology | 1/27 | 77/17910 | 0.109895 | 0.229292 | 0.171662 | CCL5 | 1 |
| GO:0060411 | BP | cardiac septum morphogenesis | 1/27 | 77/17910 | 0.109895 | 0.229292 | 0.171662 | TGFBR3 | 1 |
| GO:0060761 | BP | negative regulation of response to cytokine stimulus | 1/27 | 77/17910 | 0.109895 | 0.229292 | 0.171662 | CCL5 | 1 |
| GO:0071260 | BP | cellular response to mechanical stimulus | 1/27 | 77/17910 | 0.109895 | 0.229292 | 0.171662 | GADD45A | 1 |
| GO:0001578 | BP | microtubule bundle formation | 1/27 | 78/17910 | 0.111242 | 0.230816 | 0.172803 | PRC1 | 1 |
| GO:0010827 | BP | regulation of glucose transmembrane transport | 1/27 | 78/17910 | 0.111242 | 0.230816 | 0.172803 | GRB10 | 1 |
| GO:0022617 | BP | extracellular matrix disassembly | 1/27 | 78/17910 | 0.111242 | 0.230816 | 0.172803 | DPP4 | 1 |
| GO:0030512 | BP | negative regulation of transforming growth factor beta receptor signaling pathway | 1/27 | 78/17910 | 0.111242 | 0.230816 | 0.172803 | TGFBR3 | 1 |
| GO:0015833 | BP | peptide transport | 5/27 | 1722/17910 | 0.111552 | 0.231139 | 0.173045 | RPL10A/RPL36/DPP4/IFIT1/CCL5 | 5 |
| GO:0001944 | BP | vasculature development | 3/27 | 784/17910 | 0.112463 | 0.23168 | 0.17345 | TGFBR3/HPGD/CEACAM1 | 3 |
| GO:0042509 | BP | regulation of tyrosine phosphorylation of STAT protein | 1/27 | 79/17910 | 0.112588 | 0.23168 | 0.17345 | CCL5 | 1 |
| GO:0050818 | BP | regulation of coagulation | 1/27 | 79/17910 | 0.112588 | 0.23168 | 0.17345 | CEACAM1 | 1 |
| GO:0055021 | BP | regulation of cardiac muscle tissue growth | 1/27 | 79/17910 | 0.112588 | 0.23168 | 0.17345 | TGFBR3 | 1 |
| GO:0097581 | BP | lamellipodium organization | 1/27 | 79/17910 | 0.112588 | 0.23168 | 0.17345 | PLEKHO1 | 1 |
| GO:0010647 | BP | positive regulation of cell communication | 5/27 | 1730/17910 | 0.113226 | 0.232673 | 0.174193 | TGFBR3/GADD45A/NR3C2/CCL5/GRB10 | 5 |
| GO:0003151 | BP | outflow tract morphogenesis | 1/27 | 80/17910 | 0.113932 | 0.233482 | 0.174799 | TGFBR3 | 1 |
| GO:1903845 | BP | negative regulation of cellular response to transforming growth factor beta stimulus | 1/27 | 80/17910 | 0.113932 | 0.233482 | 0.174799 | TGFBR3 | 1 |
| GO:0023056 | BP | positive regulation of signaling | 5/27 | 1736/17910 | 0.114489 | 0.234303 | 0.175414 | TGFBR3/GADD45A/NR3C2/CCL5/GRB10 | 5 |
| GO:0072358 | BP | cardiovascular system development | 3/27 | 792/17910 | 0.115041 | 0.234625 | 0.175655 | TGFBR3/HPGD/CEACAM1 | 3 |
| GO:0002690 | BP | positive regulation of leukocyte chemotaxis | 1/27 | 81/17910 | 0.115273 | 0.234625 | 0.175655 | CCL5 | 1 |
| GO:0014068 | BP | positive regulation of phosphatidylinositol 3-kinase signaling | 1/27 | 81/17910 | 0.115273 | 0.234625 | 0.175655 | CCL5 | 1 |
| GO:0051702 | BP | interaction with symbiont | 1/27 | 81/17910 | 0.115273 | 0.234625 | 0.175655 | CCL5 | 1 |
| GO:0065003 | BP | protein-containing complex assembly | 5/27 | 1742/17910 | 0.11576 | 0.234796 | 0.175783 | TGFBR3/F5/RRM2/NLRC3/CCL5 | 5 |
| GO:0070482 | BP | response to oxygen levels | 2/27 | 389/17910 | 0.115835 | 0.234796 | 0.175783 | TGFBR3/DPP4 | 2 |
| GO:0007260 | BP | tyrosine phosphorylation of STAT protein | 1/27 | 82/17910 | 0.116613 | 0.234796 | 0.175783 | CCL5 | 1 |
| GO:0014855 | BP | striated muscle cell proliferation | 1/27 | 82/17910 | 0.116613 | 0.234796 | 0.175783 | TGFBR3 | 1 |
| GO:0031341 | BP | regulation of cell killing | 1/27 | 82/17910 | 0.116613 | 0.234796 | 0.175783 | CEACAM1 | 1 |
| GO:0048644 | BP | muscle organ morphogenesis | 1/27 | 82/17910 | 0.116613 | 0.234796 | 0.175783 | TGFBR3 | 1 |
| GO:0071158 | BP | positive regulation of cell cycle arrest | 1/27 | 82/17910 | 0.116613 | 0.234796 | 0.175783 | GADD45A | 1 |
| GO:2000106 | BP | regulation of leukocyte apoptotic process | 1/27 | 82/17910 | 0.116613 | 0.234796 | 0.175783 | CCL5 | 1 |
| GO:0051240 | BP | positive regulation of multicellular organismal process | 5/27 | 1750/17910 | 0.117465 | 0.235902 | 0.176611 | TGFBR3/VNN1/C1QA/GRB10/CEACAM1 | 5 |
| GO:0042886 | BP | amide transport | 5/27 | 1752/17910 | 0.117893 | 0.235902 | 0.176611 | RPL10A/RPL36/DPP4/IFIT1/CCL5 | 5 |
| GO:0000079 | BP | regulation of cyclin-dependent protein serine/threonine kinase activity | 1/27 | 83/17910 | 0.117951 | 0.235902 | 0.176611 | GADD45A | 1 |
| GO:0006672 | BP | ceramide metabolic process | 1/27 | 83/17910 | 0.117951 | 0.235902 | 0.176611 | ST6GALNAC3 | 1 |
| GO:0045582 | BP | positive regulation of T cell differentiation | 1/27 | 83/17910 | 0.117951 | 0.235902 | 0.176611 | VNN1 | 1 |
| GO:0048285 | BP | organelle fission | 2/27 | 395/17910 | 0.118797 | 0.236049 | 0.176722 | CDC25B/PRC1 | 2 |
| GO:0042058 | BP | regulation of epidermal growth factor receptor signaling pathway | 1/27 | 84/17910 | 0.119287 | 0.236049 | 0.176722 | CEACAM1 | 1 |
| GO:0048477 | BP | oogenesis | 1/27 | 84/17910 | 0.119287 | 0.236049 | 0.176722 | CDC25B | 1 |
| GO:0051781 | BP | positive regulation of cell division | 1/27 | 84/17910 | 0.119287 | 0.236049 | 0.176722 | CDC25B | 1 |
| GO:0055006 | BP | cardiac cell development | 1/27 | 84/17910 | 0.119287 | 0.236049 | 0.176722 | TGFBR3 | 1 |
| GO:0060420 | BP | regulation of heart growth | 1/27 | 84/17910 | 0.119287 | 0.236049 | 0.176722 | TGFBR3 | 1 |
| GO:0071621 | BP | granulocyte chemotaxis | 1/27 | 84/17910 | 0.119287 | 0.236049 | 0.176722 | CCL5 | 1 |
| GO:0090100 | BP | positive regulation of transmembrane receptor protein serine/threonine kinase signaling pathway | 1/27 | 84/17910 | 0.119287 | 0.236049 | 0.176722 | TGFBR3 | 1 |
| GO:0050778 | BP | positive regulation of immune response | 3/27 | 809/17910 | 0.120595 | 0.23681 | 0.177291 | CCL5/C1QA/CEACAM1 | 3 |
| GO:0014910 | BP | regulation of smooth muscle cell migration | 1/27 | 85/17910 | 0.120621 | 0.23681 | 0.177291 | CCL5 | 1 |
| GO:0045844 | BP | positive regulation of striated muscle tissue development | 1/27 | 85/17910 | 0.120621 | 0.23681 | 0.177291 | TGFBR3 | 1 |
| GO:0046427 | BP | positive regulation of JAK-STAT cascade | 1/27 | 85/17910 | 0.120621 | 0.23681 | 0.177291 | CCL5 | 1 |
| GO:0048636 | BP | positive regulation of muscle organ development | 1/27 | 85/17910 | 0.120621 | 0.23681 | 0.177291 | TGFBR3 | 1 |
| GO:0070542 | BP | response to fatty acid | 1/27 | 85/17910 | 0.120621 | 0.23681 | 0.177291 | TGFBR3 | 1 |
| GO:0006518 | BP | peptide metabolic process | 3/27 | 811/17910 | 0.121256 | 0.237744 | 0.17799 | RPL10A/RPL36/CCL5 | 3 |
| GO:0034103 | BP | regulation of tissue remodeling | 1/27 | 86/17910 | 0.121953 | 0.237864 | 0.17808 | CEACAM1 | 1 |
| GO:0044773 | BP | mitotic DNA damage checkpoint | 1/27 | 86/17910 | 0.121953 | 0.237864 | 0.17808 | GADD45A | 1 |
| GO:0061097 | BP | regulation of protein tyrosine kinase activity | 1/27 | 86/17910 | 0.121953 | 0.237864 | 0.17808 | CCL5 | 1 |
| GO:1901863 | BP | positive regulation of muscle tissue development | 1/27 | 86/17910 | 0.121953 | 0.237864 | 0.17808 | TGFBR3 | 1 |
| GO:0060021 | BP | roof of mouth development | 1/27 | 87/17910 | 0.123283 | 0.239211 | 0.179088 | TGFBR3 | 1 |
| GO:0072676 | BP | lymphocyte migration | 1/27 | 87/17910 | 0.123283 | 0.239211 | 0.179088 | CCL5 | 1 |
| GO:1903035 | BP | negative regulation of response to wounding | 1/27 | 87/17910 | 0.123283 | 0.239211 | 0.179088 | CEACAM1 | 1 |
| GO:1904029 | BP | regulation of cyclin-dependent protein kinase activity | 1/27 | 87/17910 | 0.123283 | 0.239211 | 0.179088 | GADD45A | 1 |
| GO:0002286 | BP | T cell activation involved in immune response | 1/27 | 88/17910 | 0.124611 | 0.239305 | 0.179159 | CEACAM1 | 1 |
| GO:0002456 | BP | T cell mediated immunity | 1/27 | 88/17910 | 0.124611 | 0.239305 | 0.179159 | CEACAM1 | 1 |
| GO:0007127 | BP | meiosis I | 1/27 | 88/17910 | 0.124611 | 0.239305 | 0.179159 | CDC25B | 1 |
| GO:0044264 | BP | cellular polysaccharide metabolic process | 1/27 | 88/17910 | 0.124611 | 0.239305 | 0.179159 | MGAM | 1 |
| GO:1900407 | BP | regulation of cellular response to oxidative stress | 1/27 | 88/17910 | 0.124611 | 0.239305 | 0.179159 | VNN1 | 1 |
| GO:1904705 | BP | regulation of vascular smooth muscle cell proliferation | 1/27 | 88/17910 | 0.124611 | 0.239305 | 0.179159 | HPGD | 1 |
| GO:1904894 | BP | positive regulation of STAT cascade | 1/27 | 88/17910 | 0.124611 | 0.239305 | 0.179159 | CCL5 | 1 |
| GO:1990874 | BP | vascular smooth muscle cell proliferation | 1/27 | 88/17910 | 0.124611 | 0.239305 | 0.179159 | HPGD | 1 |
| GO:0043604 | BP | amide biosynthetic process | 3/27 | 822/17910 | 0.124912 | 0.239575 | 0.179361 | RPL10A/RPL36/CCL5 | 3 |
| GO:0043900 | BP | regulation of multi-organism process | 2/27 | 408/17910 | 0.12528 | 0.239974 | 0.17966 | IFIT1/CCL5 | 2 |
| GO:0006903 | BP | vesicle targeting | 1/27 | 89/17910 | 0.125937 | 0.240309 | 0.179911 | F5 | 1 |
| GO:0009119 | BP | ribonucleoside metabolic process | 1/27 | 89/17910 | 0.125937 | 0.240309 | 0.179911 | APOBEC3B | 1 |
| GO:0032088 | BP | negative regulation of NF-kappaB transcription factor activity | 1/27 | 89/17910 | 0.125937 | 0.240309 | 0.179911 | NLRC3 | 1 |
| GO:0043085 | BP | positive regulation of catalytic activity | 4/27 | 1293/17910 | 0.126564 | 0.241197 | 0.180575 | CDC25B/PCOLCE2/GADD45A/CCL5 | 4 |
| GO:0030099 | BP | myeloid cell differentiation | 2/27 | 411/17910 | 0.126789 | 0.241319 | 0.180667 | TGFBR3/CEACAM1 | 2 |
| GO:0042102 | BP | positive regulation of T cell proliferation | 1/27 | 90/17910 | 0.127262 | 0.241406 | 0.180732 | CCL5 | 1 |
| GO:0044774 | BP | mitotic DNA integrity checkpoint | 1/27 | 90/17910 | 0.127262 | 0.241406 | 0.180732 | GADD45A | 1 |
| GO:0006900 | BP | vesicle budding from membrane | 1/27 | 91/17910 | 0.128584 | 0.241406 | 0.180732 | F5 | 1 |
| GO:0006958 | BP | complement activation, classical pathway | 1/27 | 91/17910 | 0.128584 | 0.241406 | 0.180732 | C1QA | 1 |
| GO:0014909 | BP | smooth muscle cell migration | 1/27 | 91/17910 | 0.128584 | 0.241406 | 0.180732 | CCL5 | 1 |
| GO:0045833 | BP | negative regulation of lipid metabolic process | 1/27 | 91/17910 | 0.128584 | 0.241406 | 0.180732 | CEACAM1 | 1 |
| GO:0060337 | BP | type I interferon signaling pathway | 1/27 | 91/17910 | 0.128584 | 0.241406 | 0.180732 | IFIT1 | 1 |
| GO:0061982 | BP | meiosis I cell cycle process | 1/27 | 91/17910 | 0.128584 | 0.241406 | 0.180732 | CDC25B | 1 |
| GO:0071357 | BP | cellular response to type I interferon | 1/27 | 91/17910 | 0.128584 | 0.241406 | 0.180732 | IFIT1 | 1 |
| GO:1901184 | BP | regulation of ERBB signaling pathway | 1/27 | 91/17910 | 0.128584 | 0.241406 | 0.180732 | CEACAM1 | 1 |
| GO:0006886 | BP | intracellular protein transport | 3/27 | 833/17910 | 0.12861 | 0.241406 | 0.180732 | RPL10A/RPL36/IFIT1 | 3 |
| GO:0001933 | BP | negative regulation of protein phosphorylation | 2/27 | 415/17910 | 0.128808 | 0.241475 | 0.180783 | GADD45A/CEACAM1 | 2 |
| GO:0045184 | BP | establishment of protein localization | 5/27 | 1803/17910 | 0.129079 | 0.241681 | 0.180937 | RPL10A/RPL36/DPP4/IFIT1/CCL5 | 5 |
| GO:0036473 | BP | cell death in response to oxidative stress | 1/27 | 92/17910 | 0.129904 | 0.242618 | 0.181639 | VNN1 | 1 |
| GO:0045638 | BP | negative regulation of myeloid cell differentiation | 1/27 | 92/17910 | 0.129904 | 0.242618 | 0.181639 | CEACAM1 | 1 |
| GO:0045621 | BP | positive regulation of lymphocyte differentiation | 1/27 | 93/17910 | 0.131223 | 0.244166 | 0.182798 | VNN1 | 1 |
| GO:0048010 | BP | vascular endothelial growth factor receptor signaling pathway | 1/27 | 93/17910 | 0.131223 | 0.244166 | 0.182798 | GRB10 | 1 |
| GO:1901216 | BP | positive regulation of neuron death | 1/27 | 93/17910 | 0.131223 | 0.244166 | 0.182798 | C1QA | 1 |
| GO:0048732 | BP | gland development | 2/27 | 422/17910 | 0.13236 | 0.244487 | 0.183038 | TGFBR3/CEACAM1 | 2 |
| GO:0001676 | BP | long-chain fatty acid metabolic process | 1/27 | 94/17910 | 0.132539 | 0.244487 | 0.183038 | HPGD | 1 |
| GO:0007229 | BP | integrin-mediated signaling pathway | 1/27 | 94/17910 | 0.132539 | 0.244487 | 0.183038 | CEACAM1 | 1 |
| GO:0060191 | BP | regulation of lipase activity | 1/27 | 94/17910 | 0.132539 | 0.244487 | 0.183038 | CCL5 | 1 |
| GO:1990823 | BP | response to leukemia inhibitory factor | 1/27 | 94/17910 | 0.132539 | 0.244487 | 0.183038 | PCOLCE2 | 1 |
| GO:1990830 | BP | cellular response to leukemia inhibitory factor | 1/27 | 94/17910 | 0.132539 | 0.244487 | 0.183038 | PCOLCE2 | 1 |
| GO:2001243 | BP | negative regulation of intrinsic apoptotic signaling pathway | 1/27 | 94/17910 | 0.132539 | 0.244487 | 0.183038 | VNN1 | 1 |
| GO:0030097 | BP | hemopoiesis | 3/27 | 847/17910 | 0.133375 | 0.245399 | 0.183721 | TGFBR3/VNN1/CEACAM1 | 3 |
| GO:0007052 | BP | mitotic spindle organization | 1/27 | 95/17910 | 0.133854 | 0.245399 | 0.183721 | PRC1 | 1 |
| GO:0019217 | BP | regulation of fatty acid metabolic process | 1/27 | 95/17910 | 0.133854 | 0.245399 | 0.183721 | CEACAM1 | 1 |
| GO:0034340 | BP | response to type I interferon | 1/27 | 95/17910 | 0.133854 | 0.245399 | 0.183721 | IFIT1 | 1 |
| GO:0072527 | BP | pyrimidine-containing compound metabolic process | 1/27 | 95/17910 | 0.133854 | 0.245399 | 0.183721 | APOBEC3B | 1 |
| GO:0046394 | BP | carboxylic acid biosynthetic process | 2/27 | 426/17910 | 0.1344 | 0.245997 | 0.184169 | HPGD/CEACAM1 | 2 |
| GO:0001932 | BP | regulation of protein phosphorylation | 4/27 | 1324/17910 | 0.134841 | 0.245997 | 0.184169 | CDC25B/GADD45A/CCL5/CEACAM1 | 4 |
| GO:0016053 | BP | organic acid biosynthetic process | 2/27 | 427/17910 | 0.134911 | 0.245997 | 0.184169 | HPGD/CEACAM1 | 2 |
| GO:0032652 | BP | regulation of interleukin-1 production | 1/27 | 96/17910 | 0.135167 | 0.245997 | 0.184169 | CEACAM1 | 1 |
| GO:0042116 | BP | macrophage activation | 1/27 | 96/17910 | 0.135167 | 0.245997 | 0.184169 | C1QA | 1 |
| GO:1901570 | BP | fatty acid derivative biosynthetic process | 1/27 | 96/17910 | 0.135167 | 0.245997 | 0.184169 | HPGD | 1 |
| GO:0006468 | BP | protein phosphorylation | 5/27 | 1835/17910 | 0.136354 | 0.24718 | 0.185054 | CDC25B/TGFBR3/GADD45A/CCL5/CEACAM1 | 5 |
| GO:0001909 | BP | leukocyte mediated cytotoxicity | 1/27 | 97/17910 | 0.136478 | 0.24718 | 0.185054 | CEACAM1 | 1 |
| GO:0120162 | BP | positive regulation of cold-induced thermogenesis | 1/27 | 97/17910 | 0.136478 | 0.24718 | 0.185054 | GRB10 | 1 |
| GO:1902882 | BP | regulation of response to oxidative stress | 1/27 | 97/17910 | 0.136478 | 0.24718 | 0.185054 | VNN1 | 1 |
| GO:0007043 | BP | cell-cell junction assembly | 1/27 | 98/17910 | 0.137786 | 0.248647 | 0.186153 | RAB13 | 1 |
| GO:0007631 | BP | feeding behavior | 1/27 | 98/17910 | 0.137786 | 0.248647 | 0.186153 | DACH1 | 1 |
| GO:0033559 | BP | unsaturated fatty acid metabolic process | 1/27 | 98/17910 | 0.137786 | 0.248647 | 0.186153 | HPGD | 1 |
| GO:0023061 | BP | signal release | 2/27 | 433/17910 | 0.137988 | 0.248712 | 0.186201 | DPP4/CCL5 | 2 |
| GO:0007155 | BP | cell adhesion | 4/27 | 1338/17910 | 0.138655 | 0.249613 | 0.186876 | DPP4/VNN1/CCL5/CEACAM1 | 4 |
| GO:0005976 | BP | polysaccharide metabolic process | 1/27 | 99/17910 | 0.139093 | 0.2498 | 0.187016 | MGAM | 1 |
| GO:1902106 | BP | negative regulation of leukocyte differentiation | 1/27 | 99/17910 | 0.139093 | 0.2498 | 0.187016 | CEACAM1 | 1 |
| GO:0046545 | BP | development of primary female sexual characteristics | 1/27 | 100/17910 | 0.140398 | 0.251239 | 0.188094 | DACH1 | 1 |
| GO:0060840 | BP | artery development | 1/27 | 100/17910 | 0.140398 | 0.251239 | 0.188094 | HPGD | 1 |
| GO:0097530 | BP | granulocyte migration | 1/27 | 100/17910 | 0.140398 | 0.251239 | 0.188094 | CCL5 | 1 |
| GO:0022610 | BP | biological adhesion | 4/27 | 1345/17910 | 0.14058 | 0.251263 | 0.188111 | DPP4/VNN1/CCL5/CEACAM1 | 4 |
| GO:0035335 | BP | peptidyl-tyrosine dephosphorylation | 1/27 | 101/17910 | 0.141702 | 0.252364 | 0.188936 | CDC25B | 1 |
| GO:0055024 | BP | regulation of cardiac muscle tissue development | 1/27 | 101/17910 | 0.141702 | 0.252364 | 0.188936 | TGFBR3 | 1 |
| GO:2000379 | BP | positive regulation of reactive oxygen species metabolic process | 1/27 | 101/17910 | 0.141702 | 0.252364 | 0.188936 | GADD45A | 1 |
| GO:0022414 | BP | reproductive process | 4/27 | 1353/17910 | 0.142793 | 0.253294 | 0.189632 | CDC25B/RAB13/HPGD/DACH1 | 4 |
| GO:0006275 | BP | regulation of DNA replication | 1/27 | 102/17910 | 0.143003 | 0.253294 | 0.189632 | DACH1 | 1 |
| GO:0006690 | BP | icosanoid metabolic process | 1/27 | 102/17910 | 0.143003 | 0.253294 | 0.189632 | HPGD | 1 |
| GO:0031623 | BP | receptor internalization | 1/27 | 102/17910 | 0.143003 | 0.253294 | 0.189632 | CEACAM1 | 1 |
| GO:0000003 | BP | reproduction | 4/27 | 1354/17910 | 0.14307 | 0.253294 | 0.189632 | CDC25B/RAB13/HPGD/DACH1 | 4 |
| GO:0032091 | BP | negative regulation of protein binding | 1/27 | 103/17910 | 0.144302 | 0.254872 | 0.190813 | IFIT1 | 1 |
| GO:0071887 | BP | leukocyte apoptotic process | 1/27 | 103/17910 | 0.144302 | 0.254872 | 0.190813 | CCL5 | 1 |
| GO:0000165 | BP | MAPK cascade | 3/27 | 882/17910 | 0.145566 | 0.256255 | 0.191849 | GADD45A/CCL5/CEACAM1 | 3 |
| GO:0002455 | BP | humoral immune response mediated by circulating immunoglobulin | 1/27 | 104/17910 | 0.1456 | 0.256255 | 0.191849 | C1QA | 1 |
| GO:0050868 | BP | negative regulation of T cell activation | 1/27 | 104/17910 | 0.1456 | 0.256255 | 0.191849 | CEACAM1 | 1 |
| GO:0002688 | BP | regulation of leukocyte chemotaxis | 1/27 | 105/17910 | 0.146895 | 0.257781 | 0.192991 | CCL5 | 1 |
| GO:0045446 | BP | endothelial cell differentiation | 1/27 | 105/17910 | 0.146895 | 0.257781 | 0.192991 | CEACAM1 | 1 |
| GO:0023014 | BP | signal transduction by protein phosphorylation | 3/27 | 886/17910 | 0.146983 | 0.257781 | 0.192991 | GADD45A/CCL5/CEACAM1 | 3 |
| GO:0002683 | BP | negative regulation of immune system process | 2/27 | 451/17910 | 0.147316 | 0.257895 | 0.193076 | NLRC3/CEACAM1 | 2 |
| GO:0008104 | BP | protein localization | 6/27 | 2417/17910 | 0.147393 | 0.257895 | 0.193076 | RAB13/RPL10A/RPL36/DPP4/IFIT1/CCL5 | 6 |
| GO:0072594 | BP | establishment of protein localization to organelle | 2/27 | 452/17910 | 0.147838 | 0.25808 | 0.193215 | RPL10A/RPL36 | 2 |
| GO:0030330 | BP | DNA damage response, signal transduction by p53 class mediator | 1/27 | 106/17910 | 0.148189 | 0.25808 | 0.193215 | GADD45A | 1 |
| GO:0055017 | BP | cardiac muscle tissue growth | 1/27 | 106/17910 | 0.148189 | 0.25808 | 0.193215 | TGFBR3 | 1 |
| GO:2000278 | BP | regulation of DNA biosynthetic process | 1/27 | 106/17910 | 0.148189 | 0.25808 | 0.193215 | DACH1 | 1 |
| GO:0048534 | BP | hematopoietic or lymphoid organ development | 3/27 | 890/17910 | 0.148406 | 0.258157 | 0.193272 | TGFBR3/VNN1/CEACAM1 | 3 |
| GO:0042326 | BP | negative regulation of phosphorylation | 2/27 | 454/17910 | 0.148884 | 0.258687 | 0.19367 | GADD45A/CEACAM1 | 2 |
| GO:0014902 | BP | myotube differentiation | 1/27 | 107/17910 | 0.149481 | 0.259123 | 0.193996 | PLEKHO1 | 1 |
| GO:0062014 | BP | negative regulation of small molecule metabolic process | 1/27 | 107/17910 | 0.149481 | 0.259123 | 0.193996 | CEACAM1 | 1 |
| GO:0051094 | BP | positive regulation of developmental process | 4/27 | 1378/17910 | 0.149803 | 0.259302 | 0.19413 | TGFBR3/VNN1/C1QA/CEACAM1 | 4 |
| GO:0050708 | BP | regulation of protein secretion | 2/27 | 456/17910 | 0.149931 | 0.259302 | 0.19413 | DPP4/CCL5 | 2 |
| GO:0006664 | BP | glycolipid metabolic process | 1/27 | 108/17910 | 0.150771 | 0.259854 | 0.194543 | ST6GALNAC3 | 1 |
| GO:0032612 | BP | interleukin-1 production | 1/27 | 108/17910 | 0.150771 | 0.259854 | 0.194543 | CEACAM1 | 1 |
| GO:0071156 | BP | regulation of cell cycle arrest | 1/27 | 108/17910 | 0.150771 | 0.259854 | 0.194543 | GADD45A | 1 |
| GO:1903509 | BP | liposaccharide metabolic process | 1/27 | 109/17910 | 0.152059 | 0.261772 | 0.195979 | ST6GALNAC3 | 1 |
| GO:0035239 | BP | tube morphogenesis | 3/27 | 905/17910 | 0.153781 | 0.264433 | 0.197972 | TGFBR3/HPGD/CEACAM1 | 3 |
| GO:0030334 | BP | regulation of cell migration | 3/27 | 906/17910 | 0.154142 | 0.264674 | 0.198152 | DACH1/CCL5/CEACAM1 | 3 |
| GO:0001934 | BP | positive regulation of protein phosphorylation | 3/27 | 907/17910 | 0.154503 | 0.264674 | 0.198152 | CDC25B/GADD45A/CCL5 | 3 |
| GO:0021782 | BP | glial cell development | 1/27 | 111/17910 | 0.154629 | 0.264674 | 0.198152 | C1QA | 1 |
| GO:0046620 | BP | regulation of organ growth | 1/27 | 111/17910 | 0.154629 | 0.264674 | 0.198152 | TGFBR3 | 1 |
| GO:0050900 | BP | leukocyte migration | 2/27 | 467/17910 | 0.155719 | 0.265956 | 0.199111 | CCL5/CEACAM1 | 2 |
| GO:0030218 | BP | erythrocyte differentiation | 1/27 | 112/17910 | 0.155911 | 0.265956 | 0.199111 | TGFBR3 | 1 |
| GO:0060419 | BP | heart growth | 1/27 | 112/17910 | 0.155911 | 0.265956 | 0.199111 | TGFBR3 | 1 |
| GO:1903706 | BP | regulation of hemopoiesis | 2/27 | 468/17910 | 0.156247 | 0.266094 | 0.199215 | VNN1/CEACAM1 | 2 |
| GO:0051649 | BP | establishment of localization in cell | 5/27 | 1919/17910 | 0.156348 | 0.266094 | 0.199215 | RAB13/F5/RPL10A/RPL36/IFIT1 | 5 |
| GO:0003279 | BP | cardiac septum development | 1/27 | 113/17910 | 0.157192 | 0.266923 | 0.199835 | TGFBR3 | 1 |
| GO:0051817 | BP | modification of morphology or physiology of other organism involved in symbiotic interaction | 1/27 | 113/17910 | 0.157192 | 0.266923 | 0.199835 | CCL5 | 1 |
| GO:1901575 | BP | organic substance catabolic process | 5/27 | 1925/17910 | 0.157824 | 0.267693 | 0.200412 | RPL10A/RPL36/MGAM/APOBEC3B/CEACAM1 | 5 |
| GO:0009116 | BP | nucleoside metabolic process | 1/27 | 114/17910 | 0.158471 | 0.268181 | 0.200777 | APOBEC3B | 1 |
| GO:0046660 | BP | female sex differentiation | 1/27 | 114/17910 | 0.158471 | 0.268181 | 0.200777 | DACH1 | 1 |
| GO:0007173 | BP | epidermal growth factor receptor signaling pathway | 1/27 | 115/17910 | 0.159747 | 0.269731 | 0.201938 | CEACAM1 | 1 |
| GO:0017015 | BP | regulation of transforming growth factor beta receptor signaling pathway | 1/27 | 115/17910 | 0.159747 | 0.269731 | 0.201938 | TGFBR3 | 1 |
| GO:0002698 | BP | negative regulation of immune effector process | 1/27 | 116/17910 | 0.161022 | 0.270662 | 0.202635 | CEACAM1 | 1 |
| GO:0022612 | BP | gland morphogenesis | 1/27 | 116/17910 | 0.161022 | 0.270662 | 0.202635 | CEACAM1 | 1 |
| GO:0030449 | BP | regulation of complement activation | 1/27 | 116/17910 | 0.161022 | 0.270662 | 0.202635 | C1QA | 1 |
| GO:1902850 | BP | microtubule cytoskeleton organization involved in mitosis | 1/27 | 116/17910 | 0.161022 | 0.270662 | 0.202635 | PRC1 | 1 |
| GO:0002761 | BP | regulation of myeloid leukocyte differentiation | 1/27 | 117/17910 | 0.162295 | 0.271581 | 0.203323 | CEACAM1 | 1 |
| GO:0045727 | BP | positive regulation of translation | 1/27 | 117/17910 | 0.162295 | 0.271581 | 0.203323 | CCL5 | 1 |
| GO:1903844 | BP | regulation of cellular response to transforming growth factor beta stimulus | 1/27 | 117/17910 | 0.162295 | 0.271581 | 0.203323 | TGFBR3 | 1 |
| GO:2000257 | BP | regulation of protein activation cascade | 1/27 | 117/17910 | 0.162295 | 0.271581 | 0.203323 | C1QA | 1 |
| GO:0098542 | BP | defense response to other organism | 2/27 | 481/17910 | 0.163151 | 0.272708 | 0.204166 | IFIT1/APOBEC3B | 2 |
| GO:0002791 | BP | regulation of peptide secretion | 2/27 | 483/17910 | 0.164218 | 0.274185 | 0.205272 | DPP4/CCL5 | 2 |
| GO:0051928 | BP | positive regulation of calcium ion transport | 1/27 | 119/17910 | 0.164836 | 0.27491 | 0.205815 | CCL5 | 1 |
| GO:0002521 | BP | leukocyte differentiation | 2/27 | 485/17910 | 0.165287 | 0.275355 | 0.206148 | VNN1/CEACAM1 | 2 |
| GO:0002520 | BP | immune system development | 3/27 | 938/17910 | 0.165834 | 0.275794 | 0.206477 | TGFBR3/VNN1/CEACAM1 | 3 |
| GO:0034101 | BP | erythrocyte homeostasis | 1/27 | 120/17910 | 0.166103 | 0.275794 | 0.206477 | TGFBR3 | 1 |
| GO:0046425 | BP | regulation of JAK-STAT cascade | 1/27 | 120/17910 | 0.166103 | 0.275794 | 0.206477 | CCL5 | 1 |
| GO:0002687 | BP | positive regulation of leukocyte migration | 1/27 | 121/17910 | 0.167369 | 0.276667 | 0.20713 | CCL5 | 1 |
| GO:0046718 | BP | viral entry into host cell | 1/27 | 121/17910 | 0.167369 | 0.276667 | 0.20713 | DPP4 | 1 |
| GO:0090101 | BP | negative regulation of transmembrane receptor protein serine/threonine kinase signaling pathway | 1/27 | 121/17910 | 0.167369 | 0.276667 | 0.20713 | TGFBR3 | 1 |
| GO:1903038 | BP | negative regulation of leukocyte cell-cell adhesion | 1/27 | 121/17910 | 0.167369 | 0.276667 | 0.20713 | CEACAM1 | 1 |
| GO:0034763 | BP | negative regulation of transmembrane transport | 1/27 | 122/17910 | 0.168633 | 0.278448 | 0.208464 | GRB10 | 1 |
| GO:0022411 | BP | cellular component disassembly | 2/27 | 492/17910 | 0.169037 | 0.278809 | 0.208734 | DPP4/C1QA | 2 |
| GO:0048468 | BP | cell development | 5/27 | 1971/17910 | 0.169348 | 0.279013 | 0.208887 | CDC25B/TGFBR3/RAB13/C1QA/GRB10 | 5 |
| GO:0008360 | BP | regulation of cell shape | 1/27 | 123/17910 | 0.169894 | 0.279299 | 0.209101 | PLEKHO1 | 1 |
| GO:0032479 | BP | regulation of type I interferon production | 1/27 | 123/17910 | 0.169894 | 0.279299 | 0.209101 | NLRC3 | 1 |
| GO:0065008 | BP | regulation of biological quality | 8/27 | 3661/17910 | 0.170204 | 0.279501 | 0.209252 | TGFBR3/F5/DPP4/PLEKHO1/IFIT1/CCL5/GRB10/CEACAM1 | 8 |
| GO:0003158 | BP | endothelium development | 1/27 | 124/17910 | 0.171155 | 0.280139 | 0.20973 | CEACAM1 | 1 |
| GO:0046330 | BP | positive regulation of JNK cascade | 1/27 | 124/17910 | 0.171155 | 0.280139 | 0.20973 | GADD45A | 1 |
| GO:2000134 | BP | negative regulation of G1/S transition of mitotic cell cycle | 1/27 | 124/17910 | 0.171155 | 0.280139 | 0.20973 | GADD45A | 1 |
| GO:0042327 | BP | positive regulation of phosphorylation | 3/27 | 955/17910 | 0.172157 | 0.281275 | 0.21058 | CDC25B/GADD45A/CCL5 | 3 |
| GO:0032606 | BP | type I interferon production | 1/27 | 125/17910 | 0.172413 | 0.281275 | 0.21058 | NLRC3 | 1 |
| GO:0050671 | BP | positive regulation of lymphocyte proliferation | 1/27 | 125/17910 | 0.172413 | 0.281275 | 0.21058 | CCL5 | 1 |
| GO:0050878 | BP | regulation of body fluid levels | 2/27 | 499/17910 | 0.172804 | 0.281607 | 0.210828 | F5/CEACAM1 | 2 |
| GO:0032270 | BP | positive regulation of cellular protein metabolic process | 4/27 | 1458/17910 | 0.173149 | 0.281861 | 0.211019 | CDC25B/PCOLCE2/GADD45A/CCL5 | 4 |
| GO:0032946 | BP | positive regulation of mononuclear cell proliferation | 1/27 | 126/17910 | 0.173669 | 0.282362 | 0.211394 | CCL5 | 1 |
| GO:0051051 | BP | negative regulation of transport | 2/27 | 502/17910 | 0.174423 | 0.282362 | 0.211394 | GRB10/CEACAM1 | 2 |
| GO:0045597 | BP | positive regulation of cell differentiation | 3/27 | 962/17910 | 0.174782 | 0.282362 | 0.211394 | VNN1/C1QA/CEACAM1 | 3 |
| GO:0031333 | BP | negative regulation of protein complex assembly | 1/27 | 127/17910 | 0.174924 | 0.282362 | 0.211394 | NLRC3 | 1 |
| GO:0010817 | BP | regulation of hormone levels | 2/27 | 503/17910 | 0.174963 | 0.282362 | 0.211394 | DPP4/CCL5 | 2 |
| GO:0006259 | BP | DNA metabolic process | 3/27 | 963/17910 | 0.175158 | 0.282362 | 0.211394 | GADD45A/RRM2/DACH1 | 3 |
| GO:0042325 | BP | regulation of phosphorylation | 4/27 | 1467/17910 | 0.175857 | 0.282362 | 0.211394 | CDC25B/GADD45A/CCL5/CEACAM1 | 4 |
| GO:0002576 | BP | platelet degranulation | 1/27 | 128/17910 | 0.176176 | 0.282362 | 0.211394 | F5 | 1 |
| GO:0006956 | BP | complement activation | 1/27 | 128/17910 | 0.176176 | 0.282362 | 0.211394 | C1QA | 1 |
| GO:0008286 | BP | insulin receptor signaling pathway | 1/27 | 128/17910 | 0.176176 | 0.282362 | 0.211394 | GRB10 | 1 |
| GO:0014013 | BP | regulation of gliogenesis | 1/27 | 128/17910 | 0.176176 | 0.282362 | 0.211394 | C1QA | 1 |
| GO:0032355 | BP | response to estradiol | 1/27 | 128/17910 | 0.176176 | 0.282362 | 0.211394 | HPGD | 1 |
| GO:0045216 | BP | cell-cell junction organization | 1/27 | 128/17910 | 0.176176 | 0.282362 | 0.211394 | RAB13 | 1 |
| GO:0051291 | BP | protein heterooligomerization | 1/27 | 128/17910 | 0.176176 | 0.282362 | 0.211394 | RRM2 | 1 |
| GO:2000145 | BP | regulation of cell motility | 3/27 | 966/17910 | 0.176288 | 0.282362 | 0.211394 | DACH1/CCL5/CEACAM1 | 3 |
| GO:0003206 | BP | cardiac chamber morphogenesis | 1/27 | 129/17910 | 0.177427 | 0.28358 | 0.212306 | TGFBR3 | 1 |
| GO:1904892 | BP | regulation of STAT cascade | 1/27 | 129/17910 | 0.177427 | 0.28358 | 0.212306 | CCL5 | 1 |
| GO:0006261 | BP | DNA-dependent DNA replication | 1/27 | 130/17910 | 0.178676 | 0.284362 | 0.212891 | DACH1 | 1 |
| GO:0007156 | BP | homophilic cell adhesion via plasma membrane adhesion molecules | 1/27 | 130/17910 | 0.178676 | 0.284362 | 0.212891 | CEACAM1 | 1 |
| GO:0050921 | BP | positive regulation of chemotaxis | 1/27 | 130/17910 | 0.178676 | 0.284362 | 0.212891 | CCL5 | 1 |
| GO:1902807 | BP | negative regulation of cell cycle G1/S phase transition | 1/27 | 130/17910 | 0.178676 | 0.284362 | 0.212891 | GADD45A | 1 |
| GO:0031349 | BP | positive regulation of defense response | 2/27 | 512/17910 | 0.179839 | 0.285436 | 0.213695 | CCL5/C1QA | 2 |
| GO:0003231 | BP | cardiac ventricle development | 1/27 | 131/17910 | 0.179924 | 0.285436 | 0.213695 | TGFBR3 | 1 |
| GO:0042770 | BP | signal transduction in response to DNA damage | 1/27 | 131/17910 | 0.179924 | 0.285436 | 0.213695 | GADD45A | 1 |
| GO:0000070 | BP | mitotic sister chromatid segregation | 1/27 | 132/17910 | 0.181169 | 0.286197 | 0.214265 | PRC1 | 1 |
| GO:0007586 | BP | digestion | 1/27 | 132/17910 | 0.181169 | 0.286197 | 0.214265 | MGAM | 1 |
| GO:0051250 | BP | negative regulation of lymphocyte activation | 1/27 | 132/17910 | 0.181169 | 0.286197 | 0.214265 | CEACAM1 | 1 |
| GO:1901657 | BP | glycosyl compound metabolic process | 1/27 | 132/17910 | 0.181169 | 0.286197 | 0.214265 | APOBEC3B | 1 |
| GO:0000077 | BP | DNA damage checkpoint | 1/27 | 133/17910 | 0.182413 | 0.287858 | 0.215509 | GADD45A | 1 |
| GO:0030260 | BP | entry into host cell | 1/27 | 134/17910 | 0.183654 | 0.287995 | 0.215611 | DPP4 | 1 |
| GO:0030509 | BP | BMP signaling pathway | 1/27 | 134/17910 | 0.183654 | 0.287995 | 0.215611 | TGFBR3 | 1 |
| GO:0044409 | BP | entry into host | 1/27 | 134/17910 | 0.183654 | 0.287995 | 0.215611 | DPP4 | 1 |
| GO:0051806 | BP | entry into cell of other organism involved in symbiotic interaction | 1/27 | 134/17910 | 0.183654 | 0.287995 | 0.215611 | DPP4 | 1 |
| GO:0051828 | BP | entry into other organism involved in symbiotic interaction | 1/27 | 134/17910 | 0.183654 | 0.287995 | 0.215611 | DPP4 | 1 |
| GO:0070665 | BP | positive regulation of leukocyte proliferation | 1/27 | 134/17910 | 0.183654 | 0.287995 | 0.215611 | CCL5 | 1 |
| GO:0002822 | BP | regulation of adaptive immune response based on somatic recombination of immune receptors built from immunoglobulin superfamily domains | 1/27 | 135/17910 | 0.184894 | 0.289333 | 0.216613 | CEACAM1 | 1 |
| GO:0002920 | BP | regulation of humoral immune response | 1/27 | 135/17910 | 0.184894 | 0.289333 | 0.216613 | C1QA | 1 |
| GO:0043933 | BP | protein-containing complex subunit organization | 5/27 | 2032/17910 | 0.185165 | 0.289453 | 0.216703 | TGFBR3/F5/RRM2/NLRC3/CCL5 | 5 |
| GO:0044255 | BP | cellular lipid metabolic process | 3/27 | 991/17910 | 0.185786 | 0.289453 | 0.216703 | ST6GALNAC3/HPGD/CEACAM1 | 3 |
| GO:0008015 | BP | blood circulation | 2/27 | 523/17910 | 0.185828 | 0.289453 | 0.216703 | F5/CEACAM1 | 2 |
| GO:0071705 | BP | nitrogen compound transport | 5/27 | 2035/17910 | 0.185958 | 0.289453 | 0.216703 | RPL10A/RPL36/DPP4/IFIT1/CCL5 | 5 |
| GO:0002706 | BP | regulation of lymphocyte mediated immunity | 1/27 | 136/17910 | 0.186132 | 0.289453 | 0.216703 | CEACAM1 | 1 |
| GO:1903305 | BP | regulation of regulated secretory pathway | 1/27 | 136/17910 | 0.186132 | 0.289453 | 0.216703 | CEACAM1 | 1 |
| GO:0008584 | BP | male gonad development | 1/27 | 137/17910 | 0.187369 | 0.290771 | 0.21769 | RAB13 | 1 |
| GO:0015718 | BP | monocarboxylic acid transport | 1/27 | 137/17910 | 0.187369 | 0.290771 | 0.21769 | CEACAM1 | 1 |
| GO:0038127 | BP | ERBB signaling pathway | 1/27 | 138/17910 | 0.188603 | 0.291779 | 0.218444 | CEACAM1 | 1 |
| GO:0044344 | BP | cellular response to fibroblast growth factor stimulus | 1/27 | 138/17910 | 0.188603 | 0.291779 | 0.218444 | CCL5 | 1 |
| GO:0046546 | BP | development of primary male sexual characteristics | 1/27 | 138/17910 | 0.188603 | 0.291779 | 0.218444 | RAB13 | 1 |
| GO:0007267 | BP | cell-cell signaling | 4/27 | 1510/17910 | 0.189014 | 0.292112 | 0.218693 | DPP4/CCL5/C1QA/GRB10 | 4 |
| GO:0042981 | BP | regulation of apoptotic process | 4/27 | 1512/17910 | 0.189634 | 0.292476 | 0.218966 | GADD45A/HPGD/VNN1/CCL5 | 4 |
| GO:0032675 | BP | regulation of interleukin-6 production | 1/27 | 139/17910 | 0.189836 | 0.292476 | 0.218966 | NLRC3 | 1 |
| GO:0045580 | BP | regulation of T cell differentiation | 1/27 | 139/17910 | 0.189836 | 0.292476 | 0.218966 | VNN1 | 1 |
| GO:0048666 | BP | neuron development | 3/27 | 1003/17910 | 0.190396 | 0.292865 | 0.219257 | RAB13/C1QA/GRB10 | 3 |
| GO:0003013 | BP | circulatory system process | 2/27 | 532/17910 | 0.190752 | 0.292865 | 0.219257 | F5/CEACAM1 | 2 |
| GO:0001837 | BP | epithelial to mesenchymal transition | 1/27 | 140/17910 | 0.191067 | 0.292865 | 0.219257 | TGFBR3 | 1 |
| GO:0007259 | BP | JAK-STAT cascade | 1/27 | 140/17910 | 0.191067 | 0.292865 | 0.219257 | CCL5 | 1 |
| GO:0034250 | BP | positive regulation of cellular amide metabolic process | 1/27 | 140/17910 | 0.191067 | 0.292865 | 0.219257 | CCL5 | 1 |
| GO:0031570 | BP | DNA integrity checkpoint | 1/27 | 141/17910 | 0.192296 | 0.294146 | 0.220216 | GADD45A | 1 |
| GO:0051053 | BP | negative regulation of DNA metabolic process | 1/27 | 141/17910 | 0.192296 | 0.294146 | 0.220216 | DACH1 | 1 |
| GO:0140013 | BP | meiotic nuclear division | 1/27 | 142/17910 | 0.193523 | 0.295721 | 0.221395 | CDC25B | 1 |
| GO:0061041 | BP | regulation of wound healing | 1/27 | 143/17910 | 0.194749 | 0.296082 | 0.221665 | CEACAM1 | 1 |
| GO:0106106 | BP | cold-induced thermogenesis | 1/27 | 143/17910 | 0.194749 | 0.296082 | 0.221665 | GRB10 | 1 |
| GO:0120161 | BP | regulation of cold-induced thermogenesis | 1/27 | 143/17910 | 0.194749 | 0.296082 | 0.221665 | GRB10 | 1 |
| GO:1902107 | BP | positive regulation of leukocyte differentiation | 1/27 | 143/17910 | 0.194749 | 0.296082 | 0.221665 | VNN1 | 1 |
| GO:1903670 | BP | regulation of sprouting angiogenesis | 1/27 | 143/17910 | 0.194749 | 0.296082 | 0.221665 | CEACAM1 | 1 |
| GO:0006665 | BP | sphingolipid metabolic process | 1/27 | 144/17910 | 0.195973 | 0.297338 | 0.222606 | ST6GALNAC3 | 1 |
| GO:0010212 | BP | response to ionizing radiation | 1/27 | 144/17910 | 0.195973 | 0.297338 | 0.222606 | GADD45A | 1 |
| GO:1902533 | BP | positive regulation of intracellular signal transduction | 3/27 | 1019/17910 | 0.196594 | 0.297978 | 0.223085 | GADD45A/NR3C2/CCL5 | 3 |
| GO:0051186 | BP | cofactor metabolic process | 2/27 | 543/17910 | 0.196797 | 0.297983 | 0.223089 | SLC2A3/VNN1 | 2 |
| GO:0002262 | BP | myeloid cell homeostasis | 1/27 | 145/17910 | 0.197194 | 0.297983 | 0.223089 | TGFBR3 | 1 |
| GO:0071774 | BP | response to fibroblast growth factor | 1/27 | 145/17910 | 0.197194 | 0.297983 | 0.223089 | CCL5 | 1 |
| GO:0043067 | BP | regulation of programmed cell death | 4/27 | 1539/17910 | 0.198077 | 0.299015 | 0.223862 | GADD45A/HPGD/VNN1/CCL5 | 4 |
| GO:0010562 | BP | positive regulation of phosphorus metabolic process | 3/27 | 1025/17910 | 0.198932 | 0.299249 | 0.224037 | CDC25B/GADD45A/CCL5 | 3 |
| GO:0045937 | BP | positive regulation of phosphate metabolic process | 3/27 | 1025/17910 | 0.198932 | 0.299249 | 0.224037 | CDC25B/GADD45A/CCL5 | 3 |
| GO:0034613 | BP | cellular protein localization | 4/27 | 1543/17910 | 0.199339 | 0.299249 | 0.224037 | RAB13/RPL10A/RPL36/IFIT1 | 4 |
| GO:0007051 | BP | spindle organization | 1/27 | 147/17910 | 0.199633 | 0.299249 | 0.224037 | PRC1 | 1 |
| GO:0007093 | BP | mitotic cell cycle checkpoint | 1/27 | 147/17910 | 0.199633 | 0.299249 | 0.224037 | GADD45A | 1 |
| GO:0071772 | BP | response to BMP | 1/27 | 147/17910 | 0.199633 | 0.299249 | 0.224037 | TGFBR3 | 1 |
| GO:0071773 | BP | cellular response to BMP stimulus | 1/27 | 147/17910 | 0.199633 | 0.299249 | 0.224037 | TGFBR3 | 1 |
| GO:0009306 | BP | protein secretion | 2/27 | 549/17910 | 0.200105 | 0.299657 | 0.224342 | DPP4/CCL5 | 2 |
| GO:0042060 | BP | wound healing | 2/27 | 550/17910 | 0.200657 | 0.299678 | 0.224358 | F5/CEACAM1 | 2 |
| GO:1902532 | BP | negative regulation of intracellular signal transduction | 2/27 | 550/17910 | 0.200657 | 0.299678 | 0.224358 | VNN1/NLRC3 | 2 |
| GO:0008277 | BP | regulation of G protein-coupled receptor signaling pathway | 1/27 | 148/17910 | 0.20085 | 0.299678 | 0.224358 | CCL5 | 1 |
| GO:0051247 | BP | positive regulation of protein metabolic process | 4/27 | 1548/17910 | 0.20092 | 0.299678 | 0.224358 | CDC25B/PCOLCE2/GADD45A/CCL5 | 4 |
| GO:0070727 | BP | cellular macromolecule localization | 4/27 | 1550/17910 | 0.201554 | 0.300187 | 0.224739 | RAB13/RPL10A/RPL36/IFIT1 | 4 |
| GO:0016202 | BP | regulation of striated muscle tissue development | 1/27 | 149/17910 | 0.202064 | 0.300187 | 0.224739 | TGFBR3 | 1 |
| GO:0032635 | BP | interleukin-6 production | 1/27 | 149/17910 | 0.202064 | 0.300187 | 0.224739 | NLRC3 | 1 |
| GO:0042129 | BP | regulation of T cell proliferation | 1/27 | 149/17910 | 0.202064 | 0.300187 | 0.224739 | CCL5 | 1 |
| GO:0040012 | BP | regulation of locomotion | 3/27 | 1035/17910 | 0.202845 | 0.301048 | 0.225383 | DACH1/CCL5/CEACAM1 | 3 |
| GO:0002819 | BP | regulation of adaptive immune response | 1/27 | 150/17910 | 0.203277 | 0.301092 | 0.225416 | CEACAM1 | 1 |
| GO:0097696 | BP | STAT cascade | 1/27 | 150/17910 | 0.203277 | 0.301092 | 0.225416 | CCL5 | 1 |
| GO:0030856 | BP | regulation of epithelial cell differentiation | 1/27 | 151/17910 | 0.204489 | 0.301691 | 0.225865 | CEACAM1 | 1 |
| GO:0032680 | BP | regulation of tumor necrosis factor production | 1/27 | 151/17910 | 0.204489 | 0.301691 | 0.225865 | NLRC3 | 1 |
| GO:0072376 | BP | protein activation cascade | 1/27 | 151/17910 | 0.204489 | 0.301691 | 0.225865 | C1QA | 1 |
| GO:1903046 | BP | meiotic cell cycle process | 1/27 | 151/17910 | 0.204489 | 0.301691 | 0.225865 | CDC25B | 1 |
| GO:0030168 | BP | platelet activation | 1/27 | 152/17910 | 0.205698 | 0.302283 | 0.226308 | CEACAM1 | 1 |
| GO:1901861 | BP | regulation of muscle tissue development | 1/27 | 152/17910 | 0.205698 | 0.302283 | 0.226308 | TGFBR3 | 1 |
| GO:1903707 | BP | negative regulation of hemopoiesis | 1/27 | 152/17910 | 0.205698 | 0.302283 | 0.226308 | CEACAM1 | 1 |
| GO:1990845 | BP | adaptive thermogenesis | 1/27 | 152/17910 | 0.205698 | 0.302283 | 0.226308 | GRB10 | 1 |
| GO:0048634 | BP | regulation of muscle organ development | 1/27 | 153/17910 | 0.206906 | 0.303164 | 0.226968 | TGFBR3 | 1 |
| GO:0050729 | BP | positive regulation of inflammatory response | 1/27 | 153/17910 | 0.206906 | 0.303164 | 0.226968 | C1QA | 1 |
| GO:0071346 | BP | cellular response to interferon-gamma | 1/27 | 153/17910 | 0.206906 | 0.303164 | 0.226968 | CCL5 | 1 |
| GO:0002250 | BP | adaptive immune response | 2/27 | 563/17910 | 0.207854 | 0.303573 | 0.227274 | C1QA/CEACAM1 | 2 |
| GO:0035051 | BP | cardiocyte differentiation | 1/27 | 154/17910 | 0.208112 | 0.303573 | 0.227274 | TGFBR3 | 1 |
| GO:0045931 | BP | positive regulation of mitotic cell cycle | 1/27 | 154/17910 | 0.208112 | 0.303573 | 0.227274 | CDC25B | 1 |
| GO:1901568 | BP | fatty acid derivative metabolic process | 1/27 | 154/17910 | 0.208112 | 0.303573 | 0.227274 | HPGD | 1 |
| GO:0051259 | BP | protein complex oligomerization | 2/27 | 564/17910 | 0.208409 | 0.303573 | 0.227274 | RRM2/CCL5 | 2 |
| GO:0006464 | BP | cellular protein modification process | 8/27 | 3851/17910 | 0.208605 | 0.303573 | 0.227274 | CDC25B/TGFBR3/ST6GALNAC3/RAB13/F5/GADD45A/CCL5/CEACAM1 | 8 |
| GO:0036211 | BP | protein modification process | 8/27 | 3851/17910 | 0.208605 | 0.303573 | 0.227274 | CDC25B/TGFBR3/ST6GALNAC3/RAB13/F5/GADD45A/CCL5/CEACAM1 | 8 |
| GO:0032640 | BP | tumor necrosis factor production | 1/27 | 155/17910 | 0.209316 | 0.303721 | 0.227385 | NLRC3 | 1 |
| GO:0032874 | BP | positive regulation of stress-activated MAPK cascade | 1/27 | 155/17910 | 0.209316 | 0.303721 | 0.227385 | GADD45A | 1 |
| GO:1903555 | BP | regulation of tumor necrosis factor superfamily cytokine production | 1/27 | 155/17910 | 0.209316 | 0.303721 | 0.227385 | NLRC3 | 1 |
| GO:0032879 | BP | regulation of localization | 6/27 | 2688/17910 | 0.209893 | 0.303991 | 0.227587 | TGFBR3/DPP4/DACH1/CCL5/GRB10/CEACAM1 | 6 |
| GO:0001906 | BP | cell killing | 1/27 | 156/17910 | 0.210518 | 0.303991 | 0.227587 | CEACAM1 | 1 |
| GO:0070304 | BP | positive regulation of stress-activated protein kinase signaling cascade | 1/27 | 156/17910 | 0.210518 | 0.303991 | 0.227587 | GADD45A | 1 |
| GO:0071347 | BP | cellular response to interleukin-1 | 1/27 | 156/17910 | 0.210518 | 0.303991 | 0.227587 | CCL5 | 1 |
| GO:0090288 | BP | negative regulation of cellular response to growth factor stimulus | 1/27 | 156/17910 | 0.210518 | 0.303991 | 0.227587 | TGFBR3 | 1 |
| GO:0000819 | BP | sister chromatid segregation | 1/27 | 157/17910 | 0.211719 | 0.30543 | 0.228664 | PRC1 | 1 |
| GO:0045936 | BP | negative regulation of phosphate metabolic process | 2/27 | 572/17910 | 0.212855 | 0.306274 | 0.229296 | GADD45A/CEACAM1 | 2 |
| GO:0051246 | BP | regulation of protein metabolic process | 6/27 | 2700/17910 | 0.212882 | 0.306274 | 0.229296 | CDC25B/PCOLCE2/GADD45A/CCL5/C1QA/CEACAM1 | 6 |
| GO:0002285 | BP | lymphocyte activation involved in immune response | 1/27 | 158/17910 | 0.212918 | 0.306274 | 0.229296 | CEACAM1 | 1 |
| GO:0010563 | BP | negative regulation of phosphorus metabolic process | 2/27 | 573/17910 | 0.213412 | 0.306395 | 0.229387 | GADD45A/CEACAM1 | 2 |
| GO:0120039 | BP | plasma membrane bounded cell projection morphogenesis | 2/27 | 573/17910 | 0.213412 | 0.306395 | 0.229387 | PLEKHO1/GRB10 | 2 |
| GO:0071396 | BP | cellular response to lipid | 2/27 | 575/17910 | 0.214525 | 0.307699 | 0.230363 | NR3C2/CCL5 | 2 |
| GO:0002673 | BP | regulation of acute inflammatory response | 1/27 | 160/17910 | 0.21531 | 0.307939 | 0.230542 | C1QA | 1 |
| GO:0035821 | BP | modification of morphology or physiology of other organism | 1/27 | 160/17910 | 0.21531 | 0.307939 | 0.230542 | CCL5 | 1 |
| GO:0046661 | BP | male sex differentiation | 1/27 | 160/17910 | 0.21531 | 0.307939 | 0.230542 | RAB13 | 1 |
| GO:0048858 | BP | cell projection morphogenesis | 2/27 | 577/17910 | 0.215639 | 0.308115 | 0.230675 | PLEKHO1/GRB10 | 2 |
| GO:0007610 | BP | behavior | 2/27 | 578/17910 | 0.216197 | 0.308466 | 0.230937 | DPP4/DACH1 | 2 |
| GO:0003018 | BP | vascular process in circulatory system | 1/27 | 161/17910 | 0.216504 | 0.308466 | 0.230937 | CEACAM1 | 1 |
| GO:0071706 | BP | tumor necrosis factor superfamily cytokine production | 1/27 | 161/17910 | 0.216504 | 0.308466 | 0.230937 | NLRC3 | 1 |
| GO:0051100 | BP | negative regulation of binding | 1/27 | 162/17910 | 0.217696 | 0.309869 | 0.231988 | IFIT1 | 1 |
| GO:0002790 | BP | peptide secretion | 2/27 | 582/17910 | 0.218428 | 0.310616 | 0.232547 | DPP4/CCL5 | 2 |
| GO:0002695 | BP | negative regulation of leukocyte activation | 1/27 | 163/17910 | 0.218886 | 0.310677 | 0.232592 | CEACAM1 | 1 |
| GO:2001242 | BP | regulation of intrinsic apoptotic signaling pathway | 1/27 | 163/17910 | 0.218886 | 0.310677 | 0.232592 | VNN1 | 1 |
| GO:0045619 | BP | regulation of lymphocyte differentiation | 1/27 | 164/17910 | 0.220074 | 0.312067 | 0.233633 | VNN1 | 1 |
| GO:0009617 | BP | response to bacterium | 2/27 | 588/17910 | 0.221779 | 0.314187 | 0.23522 | HPGD/CCL5 | 2 |
| GO:0005975 | BP | carbohydrate metabolic process | 2/27 | 589/17910 | 0.222338 | 0.314681 | 0.23559 | MGAM/SLC2A3 | 2 |
| GO:0030154 | BP | cell differentiation | 8/27 | 3923/17910 | 0.224116 | 0.316898 | 0.23725 | CDC25B/TGFBR3/RAB13/PLEKHO1/VNN1/C1QA/GRB10/CEACAM1 | 8 |
| GO:0008219 | BP | cell death | 5/27 | 2178/17910 | 0.22527 | 0.318044 | 0.238108 | GADD45A/HPGD/VNN1/CCL5/C1QA | 5 |
| GO:0032990 | BP | cell part morphogenesis | 2/27 | 595/17910 | 0.225695 | 0.318044 | 0.238108 | PLEKHO1/GRB10 | 2 |
| GO:0035295 | BP | tube development | 3/27 | 1093/17910 | 0.225916 | 0.318044 | 0.238108 | TGFBR3/HPGD/CEACAM1 | 3 |
| GO:0022408 | BP | negative regulation of cell-cell adhesion | 1/27 | 169/17910 | 0.22599 | 0.318044 | 0.238108 | CEACAM1 | 1 |
| GO:0046328 | BP | regulation of JNK cascade | 1/27 | 169/17910 | 0.22599 | 0.318044 | 0.238108 | GADD45A | 1 |
| GO:0001959 | BP | regulation of cytokine-mediated signaling pathway | 1/27 | 170/17910 | 0.227168 | 0.318205 | 0.238229 | CCL5 | 1 |
| GO:0003205 | BP | cardiac chamber development | 1/27 | 170/17910 | 0.227168 | 0.318205 | 0.238229 | TGFBR3 | 1 |
| GO:0048771 | BP | tissue remodeling | 1/27 | 170/17910 | 0.227168 | 0.318205 | 0.238229 | CEACAM1 | 1 |
| GO:0050728 | BP | negative regulation of inflammatory response | 1/27 | 170/17910 | 0.227168 | 0.318205 | 0.238229 | NLRC3 | 1 |
| GO:0097529 | BP | myeloid leukocyte migration | 1/27 | 170/17910 | 0.227168 | 0.318205 | 0.238229 | CCL5 | 1 |
| GO:0016064 | BP | immunoglobulin mediated immune response | 1/27 | 171/17910 | 0.228344 | 0.319255 | 0.239014 | C1QA | 1 |
| GO:0043433 | BP | negative regulation of DNA-binding transcription factor activity | 1/27 | 171/17910 | 0.228344 | 0.319255 | 0.239014 | NLRC3 | 1 |
| GO:0031400 | BP | negative regulation of protein modification process | 2/27 | 601/17910 | 0.229057 | 0.319405 | 0.239126 | GADD45A/CEACAM1 | 2 |
| GO:0022607 | BP | cellular component assembly | 6/27 | 2765/17910 | 0.229363 | 0.319405 | 0.239126 | TGFBR3/RAB13/F5/RRM2/NLRC3/CCL5 | 6 |
| GO:0044093 | BP | positive regulation of molecular function | 4/27 | 1636/17910 | 0.229398 | 0.319405 | 0.239126 | CDC25B/PCOLCE2/GADD45A/CCL5 | 4 |
| GO:0001659 | BP | temperature homeostasis | 1/27 | 172/17910 | 0.229519 | 0.319405 | 0.239126 | GRB10 | 1 |
| GO:0006888 | BP | ER to Golgi vesicle-mediated transport | 1/27 | 172/17910 | 0.229519 | 0.319405 | 0.239126 | F5 | 1 |
| GO:0019724 | BP | B cell mediated immunity | 1/27 | 173/17910 | 0.230691 | 0.31955 | 0.239236 | C1QA | 1 |
| GO:0034341 | BP | response to interferon-gamma | 1/27 | 173/17910 | 0.230691 | 0.31955 | 0.239236 | CCL5 | 1 |
| GO:0070613 | BP | regulation of protein processing | 1/27 | 173/17910 | 0.230691 | 0.31955 | 0.239236 | C1QA | 1 |
| GO:0071478 | BP | cellular response to radiation | 1/27 | 173/17910 | 0.230691 | 0.31955 | 0.239236 | GADD45A | 1 |
| GO:1903034 | BP | regulation of response to wounding | 1/27 | 173/17910 | 0.230691 | 0.31955 | 0.239236 | CEACAM1 | 1 |
| GO:1903317 | BP | regulation of protein maturation | 1/27 | 175/17910 | 0.233032 | 0.322494 | 0.241439 | C1QA | 1 |
| GO:0071222 | BP | cellular response to lipopolysaccharide | 1/27 | 176/17910 | 0.2342 | 0.323511 | 0.242201 | CCL5 | 1 |
| GO:1901136 | BP | carbohydrate derivative catabolic process | 1/27 | 176/17910 | 0.2342 | 0.323511 | 0.242201 | APOBEC3B | 1 |
| GO:0042098 | BP | T cell proliferation | 1/27 | 177/17910 | 0.235365 | 0.324223 | 0.242734 | CCL5 | 1 |
| GO:0043112 | BP | receptor metabolic process | 1/27 | 177/17910 | 0.235365 | 0.324223 | 0.242734 | CEACAM1 | 1 |
| GO:2000045 | BP | regulation of G1/S transition of mitotic cell cycle | 1/27 | 177/17910 | 0.235365 | 0.324223 | 0.242734 | GADD45A | 1 |
| GO:0031401 | BP | positive regulation of protein modification process | 3/27 | 1117/17910 | 0.23563 | 0.324289 | 0.242783 | CDC25B/GADD45A/CCL5 | 3 |
| GO:0006367 | BP | transcription initiation from RNA polymerase II promoter | 1/27 | 178/17910 | 0.23653 | 0.32493 | 0.243263 | NR3C2 | 1 |
| GO:0010565 | BP | regulation of cellular ketone metabolic process | 1/27 | 178/17910 | 0.23653 | 0.32493 | 0.243263 | CEACAM1 | 1 |
| GO:0006575 | BP | cellular modified amino acid metabolic process | 1/27 | 179/17910 | 0.237692 | 0.325928 | 0.24401 | VNN1 | 1 |
| GO:0035265 | BP | organ growth | 1/27 | 179/17910 | 0.237692 | 0.325928 | 0.24401 | TGFBR3 | 1 |
| GO:0048646 | BP | anatomical structure formation involved in morphogenesis | 3/27 | 1124/17910 | 0.23848 | 0.326323 | 0.244306 | TGFBR3/PLEKHO1/CEACAM1 | 3 |
| GO:0010952 | BP | positive regulation of peptidase activity | 1/27 | 180/17910 | 0.238853 | 0.326323 | 0.244306 | PCOLCE2 | 1 |
| GO:0043401 | BP | steroid hormone mediated signaling pathway | 1/27 | 180/17910 | 0.238853 | 0.326323 | 0.244306 | NR3C2 | 1 |
| GO:0050852 | BP | T cell receptor signaling pathway | 1/27 | 180/17910 | 0.238853 | 0.326323 | 0.244306 | CEACAM1 | 1 |
| GO:0002040 | BP | sprouting angiogenesis | 1/27 | 181/17910 | 0.240012 | 0.327444 | 0.245145 | CEACAM1 | 1 |
| GO:0072359 | BP | circulatory system development | 3/27 | 1128/17910 | 0.240111 | 0.327444 | 0.245145 | TGFBR3/HPGD/CEACAM1 | 3 |
| GO:0007346 | BP | regulation of mitotic cell cycle | 2/27 | 622/17910 | 0.240857 | 0.327991 | 0.245554 | CDC25B/GADD45A | 2 |
| GO:0019220 | BP | regulation of phosphate metabolic process | 4/27 | 1671/17910 | 0.241036 | 0.327991 | 0.245554 | CDC25B/GADD45A/CCL5/CEACAM1 | 4 |
| GO:1903708 | BP | positive regulation of hemopoiesis | 1/27 | 182/17910 | 0.241169 | 0.327991 | 0.245554 | VNN1 | 1 |
| GO:0051174 | BP | regulation of phosphorus metabolic process | 4/27 | 1673/17910 | 0.241706 | 0.328071 | 0.245615 | CDC25B/GADD45A/CCL5/CEACAM1 | 4 |
| GO:0048639 | BP | positive regulation of developmental growth | 1/27 | 183/17910 | 0.242325 | 0.328071 | 0.245615 | TGFBR3 | 1 |
| GO:0060759 | BP | regulation of response to cytokine stimulus | 1/27 | 183/17910 | 0.242325 | 0.328071 | 0.245615 | CCL5 | 1 |
| GO:0070555 | BP | response to interleukin-1 | 1/27 | 183/17910 | 0.242325 | 0.328071 | 0.245615 | CCL5 | 1 |
| GO:0071219 | BP | cellular response to molecule of bacterial origin | 1/27 | 183/17910 | 0.242325 | 0.328071 | 0.245615 | CCL5 | 1 |
| GO:0050731 | BP | positive regulation of peptidyl-tyrosine phosphorylation | 1/27 | 184/17910 | 0.243479 | 0.329335 | 0.246561 | CCL5 | 1 |
| GO:0016310 | BP | phosphorylation | 5/27 | 2243/17910 | 0.244019 | 0.329767 | 0.246884 | CDC25B/TGFBR3/GADD45A/CCL5/CEACAM1 | 5 |
| GO:0002703 | BP | regulation of leukocyte mediated immunity | 1/27 | 185/17910 | 0.244632 | 0.329999 | 0.247058 | CEACAM1 | 1 |
| GO:0016052 | BP | carbohydrate catabolic process | 1/27 | 185/17910 | 0.244632 | 0.329999 | 0.247058 | MGAM | 1 |
| GO:0002685 | BP | regulation of leukocyte migration | 1/27 | 186/17910 | 0.245782 | 0.330954 | 0.247773 | CCL5 | 1 |
| GO:0050920 | BP | regulation of chemotaxis | 1/27 | 186/17910 | 0.245782 | 0.330954 | 0.247773 | CCL5 | 1 |
| GO:0007166 | BP | cell surface receptor signaling pathway | 6/27 | 2831/17910 | 0.246566 | 0.331308 | 0.248038 | TGFBR3/IFIT1/HPGD/CCL5/GRB10/CEACAM1 | 6 |
| GO:0034645 | BP | cellular macromolecule biosynthetic process | 9/27 | 4634/17910 | 0.246688 | 0.331308 | 0.248038 | TGFBR3/ST6GALNAC3/RPL10A/RRM2/RPL36/NR3C2/DACH1/NLRC3/CCL5 | 9 |
| GO:0017157 | BP | regulation of exocytosis | 1/27 | 187/17910 | 0.246931 | 0.331308 | 0.248038 | CEACAM1 | 1 |
| GO:0050866 | BP | negative regulation of cell activation | 1/27 | 187/17910 | 0.246931 | 0.331308 | 0.248038 | CEACAM1 | 1 |
| GO:0043412 | BP | macromolecule modification | 8/27 | 4027/17910 | 0.247367 | 0.331596 | 0.248254 | CDC25B/TGFBR3/ST6GALNAC3/RAB13/F5/GADD45A/CCL5/CEACAM1 | 8 |
| GO:0016197 | BP | endosomal transport | 1/27 | 188/17910 | 0.248078 | 0.331797 | 0.248405 | RAB13 | 1 |
| GO:0030595 | BP | leukocyte chemotaxis | 1/27 | 188/17910 | 0.248078 | 0.331797 | 0.248405 | CCL5 | 1 |
| GO:0048729 | BP | tissue morphogenesis | 2/27 | 635/17910 | 0.248183 | 0.331797 | 0.248405 | TGFBR3/CEACAM1 | 2 |
| GO:0006643 | BP | membrane lipid metabolic process | 1/27 | 191/17910 | 0.25151 | 0.335646 | 0.251286 | ST6GALNAC3 | 1 |
| GO:1901654 | BP | response to ketone | 1/27 | 191/17910 | 0.25151 | 0.335646 | 0.251286 | TGFBR3 | 1 |
| GO:0071897 | BP | DNA biosynthetic process | 1/27 | 192/17910 | 0.252651 | 0.336867 | 0.2522 | DACH1 | 1 |
| GO:0051336 | BP | regulation of hydrolase activity | 3/27 | 1160/17910 | 0.25324 | 0.337353 | 0.252564 | PCOLCE2/IFIT1/CCL5 | 3 |
| GO:0007565 | BP | female pregnancy | 1/27 | 193/17910 | 0.253789 | 0.337784 | 0.252886 | HPGD | 1 |
| GO:0007626 | BP | locomotory behavior | 1/27 | 194/17910 | 0.254927 | 0.337833 | 0.252923 | DPP4 | 1 |
| GO:0031099 | BP | regeneration | 1/27 | 194/17910 | 0.254927 | 0.337833 | 0.252923 | TGFBR3 | 1 |
| GO:2000377 | BP | regulation of reactive oxygen species metabolic process | 1/27 | 194/17910 | 0.254927 | 0.337833 | 0.252923 | GADD45A | 1 |
| GO:0002253 | BP | activation of immune response | 2/27 | 647/17910 | 0.254956 | 0.337833 | 0.252923 | C1QA/CEACAM1 | 2 |
| GO:0061061 | BP | muscle structure development | 2/27 | 647/17910 | 0.254956 | 0.337833 | 0.252923 | TGFBR3/PLEKHO1 | 2 |
| GO:0046890 | BP | regulation of lipid biosynthetic process | 1/27 | 195/17910 | 0.256062 | 0.338699 | 0.253572 | CEACAM1 | 1 |
| GO:1902806 | BP | regulation of cell cycle G1/S phase transition | 1/27 | 195/17910 | 0.256062 | 0.338699 | 0.253572 | GADD45A | 1 |
| GO:0031399 | BP | regulation of protein modification process | 4/27 | 1718/17910 | 0.256908 | 0.339282 | 0.254008 | CDC25B/GADD45A/CCL5/CEACAM1 | 4 |
| GO:1901135 | BP | carbohydrate derivative metabolic process | 3/27 | 1169/17910 | 0.256956 | 0.339282 | 0.254008 | ST6GALNAC3/RRM2/APOBEC3B | 3 |
| GO:0000075 | BP | cell cycle checkpoint | 1/27 | 197/17910 | 0.258328 | 0.340793 | 0.255139 | GADD45A | 1 |
| GO:0030178 | BP | negative regulation of Wnt signaling pathway | 1/27 | 198/17910 | 0.259459 | 0.341944 | 0.256001 | GRB10 | 1 |
| GO:0002064 | BP | epithelial cell development | 1/27 | 199/17910 | 0.260588 | 0.341944 | 0.256001 | RAB13 | 1 |
| GO:0048762 | BP | mesenchymal cell differentiation | 1/27 | 199/17910 | 0.260588 | 0.341944 | 0.256001 | TGFBR3 | 1 |
| GO:0050670 | BP | regulation of lymphocyte proliferation | 1/27 | 199/17910 | 0.260588 | 0.341944 | 0.256001 | CCL5 | 1 |
| GO:0050679 | BP | positive regulation of epithelial cell proliferation | 1/27 | 199/17910 | 0.260588 | 0.341944 | 0.256001 | CCL5 | 1 |
| GO:0042592 | BP | homeostatic process | 4/27 | 1730/17910 | 0.261001 | 0.341944 | 0.256001 | TGFBR3/IFIT1/CCL5/GRB10 | 4 |
| GO:0007276 | BP | gamete generation | 2/27 | 658/17910 | 0.261171 | 0.341944 | 0.256001 | CDC25B/HPGD | 2 |
| GO:0048869 | BP | cellular developmental process | 8/27 | 4088/17910 | 0.261436 | 0.341944 | 0.256001 | CDC25B/TGFBR3/RAB13/PLEKHO1/VNN1/C1QA/GRB10/CEACAM1 | 8 |
| GO:0007254 | BP | JNK cascade | 1/27 | 200/17910 | 0.261715 | 0.341944 | 0.256001 | GADD45A | 1 |
| GO:0009612 | BP | response to mechanical stimulus | 1/27 | 200/17910 | 0.261715 | 0.341944 | 0.256001 | GADD45A | 1 |
| GO:0032944 | BP | regulation of mononuclear cell proliferation | 1/27 | 200/17910 | 0.261715 | 0.341944 | 0.256001 | CCL5 | 1 |
| GO:0002573 | BP | myeloid leukocyte differentiation | 1/27 | 201/17910 | 0.26284 | 0.343115 | 0.256877 | CEACAM1 | 1 |
| GO:1903531 | BP | negative regulation of secretion by cell | 1/27 | 202/17910 | 0.263964 | 0.344281 | 0.257751 | CEACAM1 | 1 |
| GO:0051321 | BP | meiotic cell cycle | 1/27 | 203/17910 | 0.265087 | 0.345199 | 0.258438 | CDC25B | 1 |
| GO:0009611 | BP | response to wounding | 2/27 | 665/17910 | 0.265129 | 0.345199 | 0.258438 | F5/CEACAM1 | 2 |
| GO:0009628 | BP | response to abiotic stimulus | 3/27 | 1191/17910 | 0.266077 | 0.346131 | 0.259136 | TGFBR3/GADD45A/DPP4 | 3 |
| GO:0040008 | BP | regulation of growth | 2/27 | 668/17910 | 0.266826 | 0.346509 | 0.259419 | TGFBR3/CEACAM1 | 2 |
| GO:0009056 | BP | catabolic process | 5/27 | 2320/17910 | 0.266831 | 0.346509 | 0.259419 | RPL10A/RPL36/MGAM/APOBEC3B/CEACAM1 | 5 |
| GO:0007169 | BP | transmembrane receptor protein tyrosine kinase signaling pathway | 2/27 | 669/17910 | 0.267392 | 0.346937 | 0.259739 | GRB10/CEACAM1 | 2 |
| GO:0071216 | BP | cellular response to biotic stimulus | 1/27 | 206/17910 | 0.268444 | 0.348 | 0.260534 | CCL5 | 1 |
| GO:0051129 | BP | negative regulation of cellular component organization | 2/27 | 673/17910 | 0.269655 | 0.349267 | 0.261483 | DPP4/NLRC3 | 2 |
| GO:0008610 | BP | lipid biosynthetic process | 2/27 | 676/17910 | 0.271352 | 0.351162 | 0.262902 | HPGD/CEACAM1 | 2 |
| GO:0051223 | BP | regulation of protein transport | 2/27 | 677/17910 | 0.271918 | 0.35159 | 0.263222 | DPP4/CCL5 | 2 |
| GO:0007411 | BP | axon guidance | 1/27 | 210/17910 | 0.272896 | 0.35255 | 0.263941 | GRB10 | 1 |
| GO:0010001 | BP | glial cell differentiation | 1/27 | 212/17910 | 0.275113 | 0.354801 | 0.265626 | C1QA | 1 |
| GO:0097485 | BP | neuron projection guidance | 1/27 | 212/17910 | 0.275113 | 0.354801 | 0.265626 | GRB10 | 1 |
| GO:0051049 | BP | regulation of transport | 4/27 | 1773/17910 | 0.275789 | 0.355176 | 0.265907 | DPP4/CCL5/GRB10/CEACAM1 | 4 |
| GO:0051345 | BP | positive regulation of hydrolase activity | 2/27 | 684/17910 | 0.275879 | 0.355176 | 0.265907 | PCOLCE2/CCL5 | 2 |
| GO:0034329 | BP | cell junction assembly | 1/27 | 214/17910 | 0.277323 | 0.356422 | 0.26684 | RAB13 | 1 |
| GO:0070663 | BP | regulation of leukocyte proliferation | 1/27 | 214/17910 | 0.277323 | 0.356422 | 0.26684 | CCL5 | 1 |
| GO:0009755 | BP | hormone-mediated signaling pathway | 1/27 | 215/17910 | 0.278426 | 0.357226 | 0.267442 | NR3C2 | 1 |
| GO:0090092 | BP | regulation of transmembrane receptor protein serine/threonine kinase signaling pathway | 1/27 | 215/17910 | 0.278426 | 0.357226 | 0.267442 | TGFBR3 | 1 |
| GO:0043408 | BP | regulation of MAPK cascade | 2/27 | 689/17910 | 0.278709 | 0.357241 | 0.267453 | GADD45A/CEACAM1 | 2 |
| GO:0044085 | BP | cellular component biogenesis | 6/27 | 2951/17910 | 0.278915 | 0.357241 | 0.267453 | TGFBR3/RAB13/F5/RRM2/NLRC3/CCL5 | 6 |
| GO:0008406 | BP | gonad development | 1/27 | 217/17910 | 0.280626 | 0.359125 | 0.268863 | RAB13 | 1 |
| GO:0001817 | BP | regulation of cytokine production | 2/27 | 698/17910 | 0.283802 | 0.362403 | 0.271318 | NLRC3/CEACAM1 | 2 |
| GO:0032872 | BP | regulation of stress-activated MAPK cascade | 1/27 | 220/17910 | 0.283915 | 0.362403 | 0.271318 | GADD45A | 1 |
| GO:0098813 | BP | nuclear chromosome segregation | 1/27 | 220/17910 | 0.283915 | 0.362403 | 0.271318 | PRC1 | 1 |
| GO:0009059 | BP | macromolecule biosynthetic process | 9/27 | 4806/17910 | 0.284975 | 0.363178 | 0.271898 | TGFBR3/ST6GALNAC3/RPL10A/RRM2/RPL36/NR3C2/DACH1/NLRC3/CCL5 | 9 |
| GO:0048738 | BP | cardiac muscle tissue development | 1/27 | 221/17910 | 0.285008 | 0.363178 | 0.271898 | TGFBR3 | 1 |
| GO:0070302 | BP | regulation of stress-activated protein kinase signaling cascade | 1/27 | 222/17910 | 0.286099 | 0.364259 | 0.272707 | GADD45A | 1 |
| GO:2001234 | BP | negative regulation of apoptotic signaling pathway | 1/27 | 223/17910 | 0.287189 | 0.365232 | 0.273436 | VNN1 | 1 |
| GO:0010468 | BP | regulation of gene expression | 9/27 | 4819/17910 | 0.287954 | 0.365232 | 0.273436 | TGFBR3/RPL10A/RRM2/RPL36/NR3C2/DACH1/NLRC3/CCL5/C1QA | 9 |
| GO:0030182 | BP | neuron differentiation | 3/27 | 1244/17910 | 0.28824 | 0.365232 | 0.273436 | RAB13/C1QA/GRB10 | 3 |
| GO:0043122 | BP | regulation of I-kappaB kinase/NF-kappaB signaling | 1/27 | 224/17910 | 0.288277 | 0.365232 | 0.273436 | NLRC3 | 1 |
| GO:0050851 | BP | antigen receptor-mediated signaling pathway | 1/27 | 224/17910 | 0.288277 | 0.365232 | 0.273436 | CEACAM1 | 1 |
| GO:0090087 | BP | regulation of peptide transport | 2/27 | 706/17910 | 0.288328 | 0.365232 | 0.273436 | DPP4/CCL5 | 2 |
| GO:0044706 | BP | multi-multicellular organism process | 1/27 | 225/17910 | 0.289363 | 0.366233 | 0.274185 | HPGD | 1 |
| GO:0051048 | BP | negative regulation of secretion | 1/27 | 226/17910 | 0.290448 | 0.367296 | 0.274981 | CEACAM1 | 1 |
| GO:1901137 | BP | carbohydrate derivative biosynthetic process | 2/27 | 711/17910 | 0.291156 | 0.36788 | 0.275418 | ST6GALNAC3/RRM2 | 2 |
| GO:1901566 | BP | organonitrogen compound biosynthetic process | 4/27 | 1819/17910 | 0.291799 | 0.368166 | 0.275633 | ST6GALNAC3/RPL10A/RPL36/CCL5 | 4 |
| GO:0080135 | BP | regulation of cellular response to stress | 2/27 | 713/17910 | 0.292288 | 0.368166 | 0.275633 | GADD45A/VNN1 | 2 |
| GO:0006486 | BP | protein glycosylation | 1/27 | 228/17910 | 0.292614 | 0.368166 | 0.275633 | ST6GALNAC3 | 1 |
| GO:0015850 | BP | organic hydroxy compound transport | 1/27 | 228/17910 | 0.292614 | 0.368166 | 0.275633 | CEACAM1 | 1 |
| GO:0043413 | BP | macromolecule glycosylation | 1/27 | 228/17910 | 0.292614 | 0.368166 | 0.275633 | ST6GALNAC3 | 1 |
| GO:0006352 | BP | DNA-templated transcription, initiation | 1/27 | 231/17910 | 0.295849 | 0.371301 | 0.277979 | NR3C2 | 1 |
| GO:0007050 | BP | cell cycle arrest | 1/27 | 231/17910 | 0.295849 | 0.371301 | 0.277979 | GADD45A | 1 |
| GO:0010594 | BP | regulation of endothelial cell migration | 1/27 | 231/17910 | 0.295849 | 0.371301 | 0.277979 | CEACAM1 | 1 |
| GO:1904018 | BP | positive regulation of vasculature development | 1/27 | 232/17910 | 0.296925 | 0.372338 | 0.278756 | CEACAM1 | 1 |
| GO:0070201 | BP | regulation of establishment of protein localization | 2/27 | 723/17910 | 0.297942 | 0.373059 | 0.279296 | DPP4/CCL5 | 2 |
| GO:0098742 | BP | cell-cell adhesion via plasma-membrane adhesion molecules | 1/27 | 233/17910 | 0.297998 | 0.373059 | 0.279296 | CEACAM1 | 1 |
| GO:0030217 | BP | T cell differentiation | 1/27 | 234/17910 | 0.299071 | 0.374088 | 0.280066 | VNN1 | 1 |
| GO:1901991 | BP | negative regulation of mitotic cell cycle phase transition | 1/27 | 235/17910 | 0.300141 | 0.375114 | 0.280834 | GADD45A | 1 |
| GO:0072659 | BP | protein localization to plasma membrane | 1/27 | 237/17910 | 0.302278 | 0.377154 | 0.282361 | RAB13 | 1 |
| GO:0140014 | BP | mitotic nuclear division | 1/27 | 237/17910 | 0.302278 | 0.377154 | 0.282361 | PRC1 | 1 |
| GO:0044283 | BP | small molecule biosynthetic process | 2/27 | 732/17910 | 0.303027 | 0.37754 | 0.28265 | HPGD/CEACAM1 | 2 |
| GO:0000086 | BP | G2/M transition of mitotic cell cycle | 1/27 | 238/17910 | 0.303344 | 0.37754 | 0.28265 | CDC25B | 1 |
| GO:0007281 | BP | germ cell development | 1/27 | 238/17910 | 0.303344 | 0.37754 | 0.28265 | CDC25B | 1 |
| GO:0042180 | BP | cellular ketone metabolic process | 1/27 | 241/17910 | 0.306532 | 0.380558 | 0.28491 | CEACAM1 | 1 |
| GO:0048872 | BP | homeostasis of number of cells | 1/27 | 241/17910 | 0.306532 | 0.380558 | 0.28491 | TGFBR3 | 1 |
| GO:0070085 | BP | glycosylation | 1/27 | 241/17910 | 0.306532 | 0.380558 | 0.28491 | ST6GALNAC3 | 1 |
| GO:0032268 | BP | regulation of cellular protein metabolic process | 5/27 | 2452/17910 | 0.307178 | 0.380925 | 0.285185 | CDC25B/PCOLCE2/GADD45A/CCL5/CEACAM1 | 5 |
| GO:0055086 | BP | nucleobase-containing small molecule metabolic process | 2/27 | 740/17910 | 0.307543 | 0.380925 | 0.285185 | RRM2/APOBEC3B | 2 |
| GO:0071383 | BP | cellular response to steroid hormone stimulus | 1/27 | 242/17910 | 0.307592 | 0.380925 | 0.285185 | NR3C2 | 1 |
| GO:0046651 | BP | lymphocyte proliferation | 1/27 | 244/17910 | 0.309707 | 0.38291 | 0.286671 | CCL5 | 1 |
| GO:0051924 | BP | regulation of calcium ion transport | 1/27 | 244/17910 | 0.309707 | 0.38291 | 0.286671 | CCL5 | 1 |
| GO:0050730 | BP | regulation of peptidyl-tyrosine phosphorylation | 1/27 | 245/17910 | 0.310762 | 0.383786 | 0.287327 | CCL5 | 1 |
| GO:0030855 | BP | epithelial cell differentiation | 2/27 | 746/17910 | 0.310928 | 0.383786 | 0.287327 | RAB13/CEACAM1 | 2 |
| GO:0032943 | BP | mononuclear cell proliferation | 1/27 | 246/17910 | 0.311815 | 0.384564 | 0.287909 | CCL5 | 1 |
| GO:0003007 | BP | heart morphogenesis | 1/27 | 249/17910 | 0.314966 | 0.38813 | 0.290579 | TGFBR3 | 1 |
| GO:0043406 | BP | positive regulation of MAP kinase activity | 1/27 | 250/17910 | 0.316014 | 0.38846 | 0.290826 | GADD45A | 1 |
| GO:0045637 | BP | regulation of myeloid cell differentiation | 1/27 | 250/17910 | 0.316014 | 0.38846 | 0.290826 | CEACAM1 | 1 |
| GO:2000027 | BP | regulation of animal organ morphogenesis | 1/27 | 250/17910 | 0.316014 | 0.38846 | 0.290826 | CEACAM1 | 1 |
| GO:0007249 | BP | I-kappaB kinase/NF-kappaB signaling | 1/27 | 253/17910 | 0.319146 | 0.391668 | 0.293227 | NLRC3 | 1 |
| GO:0051650 | BP | establishment of vesicle localization | 1/27 | 253/17910 | 0.319146 | 0.391668 | 0.293227 | F5 | 1 |
| GO:0030162 | BP | regulation of proteolysis | 2/27 | 762/17910 | 0.319943 | 0.392302 | 0.293702 | PCOLCE2/C1QA | 2 |
| GO:1901988 | BP | negative regulation of cell cycle phase transition | 1/27 | 254/17910 | 0.320187 | 0.392302 | 0.293702 | GADD45A | 1 |
| GO:0001816 | BP | cytokine production | 2/27 | 765/17910 | 0.321631 | 0.393748 | 0.294785 | NLRC3/CEACAM1 | 2 |
| GO:0044262 | BP | cellular carbohydrate metabolic process | 1/27 | 256/17910 | 0.322265 | 0.394201 | 0.295124 | MGAM | 1 |
| GO:0006915 | BP | apoptotic process | 4/27 | 1907/17910 | 0.322852 | 0.394597 | 0.29542 | GADD45A/HPGD/VNN1/CCL5 | 4 |
| GO:0044839 | BP | cell cycle G2/M phase transition | 1/27 | 257/17910 | 0.323301 | 0.394824 | 0.29559 | CDC25B | 1 |
| GO:0060485 | BP | mesenchyme development | 1/27 | 258/17910 | 0.324336 | 0.395765 | 0.296294 | TGFBR3 | 1 |
| GO:0006796 | BP | phosphate-containing compound metabolic process | 6/27 | 3115/17910 | 0.324931 | 0.396167 | 0.296596 | CDC25B/TGFBR3/GADD45A/RRM2/CCL5/CEACAM1 | 6 |
| GO:0006629 | BP | lipid metabolic process | 3/27 | 1338/17910 | 0.327984 | 0.399564 | 0.299138 | ST6GALNAC3/HPGD/CEACAM1 | 3 |
| GO:0051146 | BP | striated muscle cell differentiation | 1/27 | 263/17910 | 0.329489 | 0.40107 | 0.300266 | PLEKHO1 | 1 |
| GO:0072331 | BP | signal transduction by p53 class mediator | 1/27 | 264/17910 | 0.330515 | 0.401992 | 0.300956 | GADD45A | 1 |
| GO:0006793 | BP | phosphorus metabolic process | 6/27 | 3139/17910 | 0.331802 | 0.403171 | 0.301839 | CDC25B/TGFBR3/GADD45A/RRM2/CCL5/CEACAM1 | 6 |
| GO:0043270 | BP | positive regulation of ion transport | 1/27 | 266/17910 | 0.332562 | 0.403171 | 0.301839 | CCL5 | 1 |
| GO:0045927 | BP | positive regulation of growth | 1/27 | 266/17910 | 0.332562 | 0.403171 | 0.301839 | TGFBR3 | 1 |
| GO:0071356 | BP | cellular response to tumor necrosis factor | 1/27 | 266/17910 | 0.332562 | 0.403171 | 0.301839 | CCL5 | 1 |
| GO:0009653 | BP | anatomical structure morphogenesis | 5/27 | 2535/17910 | 0.333158 | 0.403336 | 0.301963 | TGFBR3/PLEKHO1/HPGD/GRB10/CEACAM1 | 5 |
| GO:0051239 | BP | regulation of multicellular organismal process | 6/27 | 3144/17910 | 0.333238 | 0.403336 | 0.301963 | TGFBR3/VNN1/NLRC3/C1QA/GRB10/CEACAM1 | 6 |
| GO:0043491 | BP | protein kinase B signaling | 1/27 | 268/17910 | 0.334603 | 0.404661 | 0.302955 | CCL5 | 1 |
| GO:0072593 | BP | reactive oxygen species metabolic process | 1/27 | 269/17910 | 0.335621 | 0.405565 | 0.303632 | GADD45A | 1 |
| GO:0051648 | BP | vesicle localization | 1/27 | 270/17910 | 0.336638 | 0.406053 | 0.303997 | F5 | 1 |
| GO:0070661 | BP | leukocyte proliferation | 1/27 | 270/17910 | 0.336638 | 0.406053 | 0.303997 | CCL5 | 1 |
| GO:0051641 | BP | cellular localization | 5/27 | 2548/17910 | 0.337259 | 0.406053 | 0.303997 | RAB13/F5/RPL10A/RPL36/IFIT1 | 5 |
| GO:0007162 | BP | negative regulation of cell adhesion | 1/27 | 271/17910 | 0.337654 | 0.406053 | 0.303997 | CEACAM1 | 1 |
| GO:0051403 | BP | stress-activated MAPK cascade | 1/27 | 271/17910 | 0.337654 | 0.406053 | 0.303997 | GADD45A | 1 |
| GO:0070372 | BP | regulation of ERK1 and ERK2 cascade | 1/27 | 271/17910 | 0.337654 | 0.406053 | 0.303997 | CEACAM1 | 1 |
| GO:0033365 | BP | protein localization to organelle | 2/27 | 798/17910 | 0.340144 | 0.40858 | 0.305889 | RPL10A/RPL36 | 2 |
| GO:0120036 | BP | plasma membrane bounded cell projection organization | 3/27 | 1367/17910 | 0.340301 | 0.40858 | 0.305889 | RAB13/PLEKHO1/GRB10 | 3 |
| GO:0007059 | BP | chromosome segregation | 1/27 | 275/17910 | 0.3417 | 0.409747 | 0.306762 | PRC1 | 1 |
| GO:0048609 | BP | multicellular organismal reproductive process | 2/27 | 801/17910 | 0.341821 | 0.409747 | 0.306762 | CDC25B/HPGD | 2 |
| GO:0001822 | BP | kidney development | 1/27 | 276/17910 | 0.342708 | 0.409825 | 0.306821 | HPGD | 1 |
| GO:0006959 | BP | humoral immune response | 1/27 | 276/17910 | 0.342708 | 0.409825 | 0.306821 | C1QA | 1 |
| GO:1990778 | BP | protein localization to cell periphery | 1/27 | 276/17910 | 0.342708 | 0.409825 | 0.306821 | RAB13 | 1 |
| GO:0005996 | BP | monosaccharide metabolic process | 1/27 | 277/17910 | 0.343714 | 0.410045 | 0.306986 | SLC2A3 | 1 |
| GO:0034330 | BP | cell junction organization | 1/27 | 277/17910 | 0.343714 | 0.410045 | 0.306986 | RAB13 | 1 |
| GO:0097193 | BP | intrinsic apoptotic signaling pathway | 1/27 | 277/17910 | 0.343714 | 0.410045 | 0.306986 | VNN1 | 1 |
| GO:0031098 | BP | stress-activated protein kinase signaling cascade | 1/27 | 278/17910 | 0.344719 | 0.410916 | 0.307638 | GADD45A | 1 |
| GO:0019953 | BP | sexual reproduction | 2/27 | 808/17910 | 0.34573 | 0.411793 | 0.308294 | CDC25B/HPGD | 2 |
| GO:0016050 | BP | vesicle organization | 1/27 | 280/17910 | 0.346725 | 0.412649 | 0.308935 | F5 | 1 |
| GO:0032504 | BP | multicellular organism reproduction | 2/27 | 811/17910 | 0.347403 | 0.413128 | 0.309294 | CDC25B/HPGD | 2 |
| GO:0016071 | BP | mRNA metabolic process | 2/27 | 815/17910 | 0.349633 | 0.415449 | 0.311032 | RPL10A/RPL36 | 2 |
| GO:0034612 | BP | response to tumor necrosis factor | 1/27 | 284/17910 | 0.350718 | 0.416408 | 0.311749 | CCL5 | 1 |
| GO:0042063 | BP | gliogenesis | 1/27 | 285/17910 | 0.351712 | 0.417257 | 0.312385 | C1QA | 1 |
| GO:0050793 | BP | regulation of developmental process | 5/27 | 2598/17910 | 0.353098 | 0.41857 | 0.313368 | TGFBR3/PLEKHO1/VNN1/C1QA/CEACAM1 | 5 |
| GO:0048699 | BP | generation of neurons | 3/27 | 1398/17910 | 0.353472 | 0.418681 | 0.313451 | RAB13/C1QA/GRB10 | 3 |
| GO:0030030 | BP | cell projection organization | 3/27 | 1400/17910 | 0.354322 | 0.419356 | 0.313956 | RAB13/PLEKHO1/GRB10 | 3 |
| GO:0070371 | BP | ERK1 and ERK2 cascade | 1/27 | 289/17910 | 0.355676 | 0.420625 | 0.314907 | CEACAM1 | 1 |
| GO:0072001 | BP | renal system development | 1/27 | 291/17910 | 0.357649 | 0.422625 | 0.316404 | HPGD | 1 |
| GO:0015849 | BP | organic acid transport | 1/27 | 293/17910 | 0.359616 | 0.424279 | 0.317642 | CEACAM1 | 1 |
| GO:0046942 | BP | carboxylic acid transport | 1/27 | 293/17910 | 0.359616 | 0.424279 | 0.317642 | CEACAM1 | 1 |
| GO:0010632 | BP | regulation of epithelial cell migration | 1/27 | 294/17910 | 0.360598 | 0.425102 | 0.318258 | CEACAM1 | 1 |
| GO:0016485 | BP | protein processing | 1/27 | 295/17910 | 0.361578 | 0.425922 | 0.318872 | C1QA | 1 |
| GO:0034599 | BP | cellular response to oxidative stress | 1/27 | 296/17910 | 0.362556 | 0.426738 | 0.319483 | VNN1 | 1 |
| GO:0006898 | BP | receptor-mediated endocytosis | 1/27 | 301/17910 | 0.367428 | 0.432132 | 0.323521 | CEACAM1 | 1 |
| GO:0009101 | BP | glycoprotein biosynthetic process | 1/27 | 302/17910 | 0.368397 | 0.432932 | 0.32412 | ST6GALNAC3 | 1 |
| GO:0048193 | BP | Golgi vesicle transport | 1/27 | 304/17910 | 0.370333 | 0.434865 | 0.325568 | F5 | 1 |
| GO:0012501 | BP | programmed cell death | 4/27 | 2049/17910 | 0.373661 | 0.438429 | 0.328236 | GADD45A/HPGD/VNN1/CCL5 | 4 |
| GO:0045930 | BP | negative regulation of mitotic cell cycle | 1/27 | 308/17910 | 0.374187 | 0.438702 | 0.32844 | GADD45A | 1 |
| GO:0044248 | BP | cellular catabolic process | 4/27 | 2054/17910 | 0.375457 | 0.439847 | 0.329297 | RPL10A/RPL36/MGAM/APOBEC3B | 4 |
| GO:0071214 | BP | cellular response to abiotic stimulus | 1/27 | 310/17910 | 0.376106 | 0.439917 | 0.32935 | GADD45A | 1 |
| GO:0104004 | BP | cellular response to environmental stimulus | 1/27 | 310/17910 | 0.376106 | 0.439917 | 0.32935 | GADD45A | 1 |
| GO:0006470 | BP | protein dephosphorylation | 1/27 | 313/17910 | 0.378973 | 0.442924 | 0.331601 | CDC25B | 1 |
| GO:0000902 | BP | cell morphogenesis | 2/27 | 873/17910 | 0.381698 | 0.445761 | 0.333725 | PLEKHO1/GRB10 | 2 |
| GO:2000026 | BP | regulation of multicellular organismal development | 4/27 | 2077/17910 | 0.383719 | 0.447079 | 0.334712 | TGFBR3/VNN1/C1QA/CEACAM1 | 4 |
| GO:0003002 | BP | regionalization | 1/27 | 318/17910 | 0.383723 | 0.447079 | 0.334712 | C1QA | 1 |
| GO:0007568 | BP | aging | 1/27 | 318/17910 | 0.383723 | 0.447079 | 0.334712 | C1QA | 1 |
| GO:0031175 | BP | neuron projection development | 2/27 | 879/17910 | 0.384984 | 0.448199 | 0.33555 | RAB13/GRB10 | 2 |
| GO:0071496 | BP | cellular response to external stimulus | 1/27 | 320/17910 | 0.385613 | 0.448583 | 0.335837 | GADD45A | 1 |
| GO:0043405 | BP | regulation of MAP kinase activity | 1/27 | 322/17910 | 0.387498 | 0.450075 | 0.336955 | GADD45A | 1 |
| GO:0071902 | BP | positive regulation of protein serine/threonine kinase activity | 1/27 | 322/17910 | 0.387498 | 0.450075 | 0.336955 | GADD45A | 1 |
| GO:0001655 | BP | urogenital system development | 1/27 | 324/17910 | 0.389377 | 0.451907 | 0.338326 | HPGD | 1 |
| GO:0030098 | BP | lymphocyte differentiation | 1/27 | 325/17910 | 0.390315 | 0.452293 | 0.338615 | VNN1 | 1 |
| GO:0048638 | BP | regulation of developmental growth | 1/27 | 325/17910 | 0.390315 | 0.452293 | 0.338615 | TGFBR3 | 1 |
| GO:0022412 | BP | cellular process involved in reproduction in multicellular organism | 1/27 | 326/17910 | 0.391251 | 0.453027 | 0.339165 | CDC25B | 1 |
| GO:2001141 | BP | regulation of RNA biosynthetic process | 6/27 | 3348/17910 | 0.39259 | 0.454226 | 0.340062 | TGFBR3/RRM2/NR3C2/DACH1/NLRC3/CCL5 | 6 |
| GO:0043066 | BP | negative regulation of apoptotic process | 2/27 | 894/17910 | 0.393169 | 0.454342 | 0.340149 | VNN1/CCL5 | 2 |
| GO:0022008 | BP | neurogenesis | 3/27 | 1492/17910 | 0.393298 | 0.454342 | 0.340149 | RAB13/C1QA/GRB10 | 3 |
| GO:0001101 | BP | response to acid chemical | 1/27 | 330/17910 | 0.394981 | 0.455935 | 0.341342 | TGFBR3 | 1 |
| GO:0006869 | BP | lipid transport | 1/27 | 332/17910 | 0.396838 | 0.457373 | 0.342418 | CEACAM1 | 1 |
| GO:0030336 | BP | negative regulation of cell migration | 1/27 | 332/17910 | 0.396838 | 0.457373 | 0.342418 | DACH1 | 1 |
| GO:0045862 | BP | positive regulation of proteolysis | 1/27 | 333/17910 | 0.397765 | 0.458088 | 0.342954 | PCOLCE2 | 1 |
| GO:0030198 | BP | extracellular matrix organization | 1/27 | 334/17910 | 0.39869 | 0.4588 | 0.343487 | DPP4 | 1 |
| GO:0010038 | BP | response to metal ion | 1/27 | 335/17910 | 0.399614 | 0.459187 | 0.343777 | C1QA | 1 |
| GO:0006996 | BP | organelle organization | 6/27 | 3372/17910 | 0.39964 | 0.459187 | 0.343777 | CDC25B/RAB13/F5/GADD45A/PRC1/HEPACAM2 | 6 |
| GO:0043069 | BP | negative regulation of programmed cell death | 2/27 | 916/17910 | 0.405096 | 0.464778 | 0.347962 | VNN1/CCL5 | 2 |
| GO:0030111 | BP | regulation of Wnt signaling pathway | 1/27 | 341/17910 | 0.405127 | 0.464778 | 0.347962 | GRB10 | 1 |
| GO:0018108 | BP | peptidyl-tyrosine phosphorylation | 1/27 | 346/17910 | 0.409685 | 0.469287 | 0.351338 | CCL5 | 1 |
| GO:0150063 | BP | visual system development | 1/27 | 346/17910 | 0.409685 | 0.469287 | 0.351338 | C1QA | 1 |
| GO:2000146 | BP | negative regulation of cell motility | 1/27 | 347/17910 | 0.410592 | 0.469966 | 0.351847 | DACH1 | 1 |
| GO:0018212 | BP | peptidyl-tyrosine modification | 1/27 | 349/17910 | 0.412403 | 0.471678 | 0.353128 | CCL5 | 1 |
| GO:0048880 | BP | sensory system development | 1/27 | 351/17910 | 0.414209 | 0.473381 | 0.354403 | C1QA | 1 |
| GO:0031326 | BP | regulation of cellular biosynthetic process | 7/27 | 4060/17910 | 0.414834 | 0.473734 | 0.354667 | TGFBR3/RRM2/NR3C2/DACH1/NLRC3/CCL5/CEACAM1 | 7 |
| GO:0051604 | BP | protein maturation | 1/27 | 353/17910 | 0.416009 | 0.474713 | 0.3554 | C1QA | 1 |
| GO:0055114 | BP | oxidation-reduction process | 2/27 | 944/17910 | 0.420131 | 0.479052 | 0.358649 | RRM2/HPGD | 2 |
| GO:0042692 | BP | muscle cell differentiation | 1/27 | 359/17910 | 0.421378 | 0.480107 | 0.359439 | PLEKHO1 | 1 |
| GO:0040007 | BP | growth | 2/27 | 948/17910 | 0.422265 | 0.48039 | 0.35965 | TGFBR3/CEACAM1 | 2 |
| GO:0009165 | BP | nucleotide biosynthetic process | 1/27 | 360/17910 | 0.422268 | 0.48039 | 0.35965 | RRM2 | 1 |
| GO:0044271 | BP | cellular nitrogen compound biosynthetic process | 8/27 | 4735/17910 | 0.424116 | 0.482077 | 0.360913 | TGFBR3/RPL10A/RRM2/RPL36/NR3C2/DACH1/NLRC3/CCL5 | 8 |
| GO:0034622 | BP | cellular protein-containing complex assembly | 2/27 | 952/17910 | 0.424396 | 0.482077 | 0.360913 | TGFBR3/NLRC3 | 2 |
| GO:0010959 | BP | regulation of metal ion transport | 1/27 | 365/17910 | 0.426699 | 0.483958 | 0.362322 | CCL5 | 1 |
| GO:1901293 | BP | nucleoside phosphate biosynthetic process | 1/27 | 365/17910 | 0.426699 | 0.483958 | 0.362322 | RRM2 | 1 |
| GO:0010876 | BP | lipid localization | 1/27 | 366/17910 | 0.427581 | 0.484229 | 0.362524 | CEACAM1 | 1 |
| GO:0042493 | BP | response to drug | 2/27 | 958/17910 | 0.427584 | 0.484229 | 0.362524 | RPL10A/HPGD | 2 |
| GO:0032989 | BP | cellular component morphogenesis | 2/27 | 959/17910 | 0.428115 | 0.484463 | 0.3627 | PLEKHO1/GRB10 | 2 |
| GO:0009100 | BP | glycoprotein metabolic process | 1/27 | 369/17910 | 0.43022 | 0.486477 | 0.364207 | ST6GALNAC3 | 1 |
| GO:0045089 | BP | positive regulation of innate immune response | 1/27 | 373/17910 | 0.43372 | 0.490064 | 0.366893 | CCL5 | 1 |
| GO:0048545 | BP | response to steroid hormone | 1/27 | 374/17910 | 0.434592 | 0.490679 | 0.367353 | NR3C2 | 1 |
| GO:0006874 | BP | cellular calcium ion homeostasis | 1/27 | 375/17910 | 0.435463 | 0.490994 | 0.367589 | CCL5 | 1 |
| GO:0009889 | BP | regulation of biosynthetic process | 7/27 | 4135/17910 | 0.435528 | 0.490994 | 0.367589 | TGFBR3/RRM2/NR3C2/DACH1/NLRC3/CCL5/CEACAM1 | 7 |
| GO:0014706 | BP | striated muscle tissue development | 1/27 | 376/17910 | 0.436332 | 0.49153 | 0.367991 | TGFBR3 | 1 |
| GO:0040013 | BP | negative regulation of locomotion | 1/27 | 377/17910 | 0.4372 | 0.492138 | 0.368445 | DACH1 | 1 |
| GO:0032880 | BP | regulation of protein localization | 2/27 | 979/17910 | 0.43868 | 0.493432 | 0.369415 | DPP4/CCL5 | 2 |
| GO:0044703 | BP | multi-organism reproductive process | 2/27 | 980/17910 | 0.439206 | 0.493652 | 0.369579 | CDC25B/HPGD | 2 |
| GO:0032774 | BP | RNA biosynthetic process | 6/27 | 3516/17910 | 0.442004 | 0.496425 | 0.371655 | TGFBR3/RRM2/NR3C2/DACH1/NLRC3/CCL5 | 6 |
| GO:0045765 | BP | regulation of angiogenesis | 1/27 | 383/17910 | 0.442381 | 0.496475 | 0.371693 | CEACAM1 | 1 |
| GO:0009887 | BP | animal organ morphogenesis | 2/27 | 989/17910 | 0.443926 | 0.497837 | 0.372712 | TGFBR3/CEACAM1 | 2 |
| GO:0043062 | BP | extracellular structure organization | 1/27 | 387/17910 | 0.445809 | 0.499573 | 0.374012 | DPP4 | 1 |
| GO:0055074 | BP | calcium ion homeostasis | 1/27 | 388/17910 | 0.446663 | 0.500155 | 0.374448 | CCL5 | 1 |
| GO:0060537 | BP | muscle tissue development | 1/27 | 392/17910 | 0.450066 | 0.503213 | 0.376737 | TGFBR3 | 1 |
| GO:0072503 | BP | cellular divalent inorganic cation homeostasis | 1/27 | 392/17910 | 0.450066 | 0.503213 | 0.376737 | CCL5 | 1 |
| GO:0007409 | BP | axonogenesis | 1/27 | 393/17910 | 0.450914 | 0.503386 | 0.376867 | GRB10 | 1 |
| GO:0044265 | BP | cellular macromolecule catabolic process | 2/27 | 1003/17910 | 0.451231 | 0.503386 | 0.376867 | RPL10A/RPL36 | 2 |
| GO:0060548 | BP | negative regulation of cell death | 2/27 | 1003/17910 | 0.451231 | 0.503386 | 0.376867 | VNN1/CCL5 | 2 |
| GO:0050808 | BP | synapse organization | 1/27 | 394/17910 | 0.45176 | 0.503601 | 0.377028 | C1QA | 1 |
| GO:0051252 | BP | regulation of RNA metabolic process | 6/27 | 3552/17910 | 0.452577 | 0.504137 | 0.377428 | TGFBR3/RRM2/NR3C2/DACH1/NLRC3/CCL5 | 6 |
| GO:0001558 | BP | regulation of cell growth | 1/27 | 396/17910 | 0.453449 | 0.504732 | 0.377874 | CEACAM1 | 1 |
| GO:0006417 | BP | regulation of translation | 1/27 | 397/17910 | 0.454291 | 0.504918 | 0.378014 | CCL5 | 1 |
| GO:2001233 | BP | regulation of apoptotic signaling pathway | 1/27 | 397/17910 | 0.454291 | 0.504918 | 0.378014 | VNN1 | 1 |
| GO:0052547 | BP | regulation of peptidase activity | 1/27 | 398/17910 | 0.455133 | 0.505478 | 0.378433 | PCOLCE2 | 1 |
| GO:0002429 | BP | immune response-activating cell surface receptor signaling pathway | 1/27 | 399/17910 | 0.455973 | 0.506035 | 0.37885 | CEACAM1 | 1 |
| GO:0006508 | BP | proteolysis | 3/27 | 1645/17910 | 0.457017 | 0.506818 | 0.379436 | PCOLCE2/DPP4/C1QA | 3 |
| GO:0048878 | BP | chemical homeostasis | 2/27 | 1015/17910 | 0.457452 | 0.506925 | 0.379516 | IFIT1/CCL5 | 2 |
| GO:0019216 | BP | regulation of lipid metabolic process | 1/27 | 402/17910 | 0.458486 | 0.507492 | 0.37994 | CEACAM1 | 1 |
| GO:0016070 | BP | RNA metabolic process | 8/27 | 4867/17910 | 0.458772 | 0.507492 | 0.37994 | TGFBR3/RPL10A/RRM2/RPL36/NR3C2/DACH1/NLRC3/CCL5 | 8 |
| GO:0007517 | BP | muscle organ development | 1/27 | 403/17910 | 0.459321 | 0.507492 | 0.37994 | TGFBR3 | 1 |
| GO:0051052 | BP | regulation of DNA metabolic process | 1/27 | 403/17910 | 0.459321 | 0.507492 | 0.37994 | DACH1 | 1 |
| GO:0044282 | BP | small molecule catabolic process | 1/27 | 404/17910 | 0.460154 | 0.508038 | 0.380349 | APOBEC3B | 1 |
| GO:0007389 | BP | pattern specification process | 1/27 | 408/17910 | 0.463478 | 0.511329 | 0.382813 | C1QA | 1 |
| GO:0043254 | BP | regulation of protein complex assembly | 1/27 | 409/17910 | 0.464305 | 0.511865 | 0.383214 | NLRC3 | 1 |
| GO:0072507 | BP | divalent inorganic cation homeostasis | 1/27 | 412/17910 | 0.466781 | 0.514215 | 0.384974 | CCL5 | 1 |
| GO:0006816 | BP | calcium ion transport | 1/27 | 415/17910 | 0.469246 | 0.51655 | 0.386722 | CCL5 | 1 |
| GO:0051346 | BP | negative regulation of hydrolase activity | 1/27 | 420/17910 | 0.473329 | 0.520662 | 0.3898 | IFIT1 | 1 |
| GO:0032269 | BP | negative regulation of cellular protein metabolic process | 2/27 | 1049/17910 | 0.474878 | 0.521982 | 0.390788 | GADD45A/CEACAM1 | 2 |
| GO:0051128 | BP | regulation of cellular component organization | 4/27 | 2340/17910 | 0.477278 | 0.523965 | 0.392273 | DPP4/PLEKHO1/NLRC3/CEACAM1 | 4 |
| GO:1901342 | BP | regulation of vasculature development | 1/27 | 425/17910 | 0.477382 | 0.523965 | 0.392273 | CEACAM1 | 1 |
| GO:0048608 | BP | reproductive structure development | 1/27 | 426/17910 | 0.478189 | 0.524465 | 0.392648 | RAB13 | 1 |
| GO:1901990 | BP | regulation of mitotic cell cycle phase transition | 1/27 | 428/17910 | 0.4798 | 0.525572 | 0.393476 | GADD45A | 1 |
| GO:0022604 | BP | regulation of cell morphogenesis | 1/27 | 429/17910 | 0.480603 | 0.525572 | 0.393476 | PLEKHO1 | 1 |
| GO:0051090 | BP | regulation of DNA-binding transcription factor activity | 1/27 | 429/17910 | 0.480603 | 0.525572 | 0.393476 | NLRC3 | 1 |
| GO:0061458 | BP | reproductive system development | 1/27 | 429/17910 | 0.480603 | 0.525572 | 0.393476 | RAB13 | 1 |
| GO:0015711 | BP | organic anion transport | 1/27 | 430/17910 | 0.481405 | 0.526064 | 0.393845 | CEACAM1 | 1 |
| GO:0002768 | BP | immune response-regulating cell surface receptor signaling pathway | 1/27 | 434/17910 | 0.484602 | 0.529171 | 0.396171 | CEACAM1 | 1 |
| GO:0009314 | BP | response to radiation | 1/27 | 435/17910 | 0.485398 | 0.529654 | 0.396533 | GADD45A | 1 |
| GO:0061564 | BP | axon development | 1/27 | 438/17910 | 0.48778 | 0.531865 | 0.398188 | GRB10 | 1 |
| GO:0062012 | BP | regulation of small molecule metabolic process | 1/27 | 439/17910 | 0.488572 | 0.53234 | 0.398544 | CEACAM1 | 1 |
| GO:0006979 | BP | response to oxidative stress | 1/27 | 442/17910 | 0.490939 | 0.534531 | 0.400183 | VNN1 | 1 |
| GO:0007186 | BP | G protein-coupled receptor signaling pathway | 2/27 | 1087/17910 | 0.493984 | 0.537455 | 0.402373 | HPGD/CCL5 | 2 |
| GO:0051656 | BP | establishment of organelle localization | 1/27 | 448/17910 | 0.495643 | 0.538868 | 0.40343 | F5 | 1 |
| GO:0034248 | BP | regulation of cellular amide metabolic process | 1/27 | 452/17910 | 0.498755 | 0.541858 | 0.405669 | CCL5 | 1 |
| GO:0070838 | BP | divalent metal ion transport | 1/27 | 454/17910 | 0.500305 | 0.543146 | 0.406634 | CCL5 | 1 |
| GO:0050769 | BP | positive regulation of neurogenesis | 1/27 | 455/17910 | 0.501077 | 0.543563 | 0.406946 | C1QA | 1 |
| GO:0022603 | BP | regulation of anatomical structure morphogenesis | 2/27 | 1102/17910 | 0.501415 | 0.543563 | 0.406946 | PLEKHO1/CEACAM1 | 2 |
| GO:2000112 | BP | regulation of cellular macromolecule biosynthetic process | 6/27 | 3725/17910 | 0.502979 | 0.544519 | 0.407662 | TGFBR3/RRM2/NR3C2/DACH1/NLRC3/CCL5 | 6 |
| GO:0016311 | BP | dephosphorylation | 1/27 | 458/17910 | 0.503389 | 0.544519 | 0.407662 | CDC25B | 1 |
| GO:0072511 | BP | divalent inorganic cation transport | 1/27 | 458/17910 | 0.503389 | 0.544519 | 0.407662 | CCL5 | 1 |
| GO:1901987 | BP | regulation of cell cycle phase transition | 1/27 | 464/17910 | 0.507982 | 0.548759 | 0.410836 | GADD45A | 1 |
| GO:0051248 | BP | negative regulation of protein metabolic process | 2/27 | 1116/17910 | 0.508293 | 0.548759 | 0.410836 | GADD45A/CEACAM1 | 2 |
| GO:0010605 | BP | negative regulation of macromolecule metabolic process | 5/27 | 3087/17910 | 0.508409 | 0.548759 | 0.410836 | GADD45A/RPL10A/RPL36/DACH1/CEACAM1 | 5 |
| GO:0016049 | BP | cell growth | 1/27 | 466/17910 | 0.509504 | 0.549544 | 0.411423 | CEACAM1 | 1 |
| GO:0006875 | BP | cellular metal ion homeostasis | 1/27 | 467/17910 | 0.510263 | 0.549966 | 0.41174 | CCL5 | 1 |
| GO:1901565 | BP | organonitrogen compound catabolic process | 2/27 | 1123/17910 | 0.51171 | 0.551129 | 0.41261 | APOBEC3B/CEACAM1 | 2 |
| GO:0048871 | BP | multicellular organismal homeostasis | 1/27 | 472/17910 | 0.514042 | 0.553242 | 0.414192 | GRB10 | 1 |
| GO:0071900 | BP | regulation of protein serine/threonine kinase activity | 1/27 | 479/17910 | 0.519285 | 0.558484 | 0.418116 | GADD45A | 1 |
| GO:0060627 | BP | regulation of vesicle-mediated transport | 1/27 | 480/17910 | 0.52003 | 0.558883 | 0.418415 | CEACAM1 | 1 |
| GO:0045595 | BP | regulation of cell differentiation | 3/27 | 1808/17910 | 0.522163 | 0.560772 | 0.419829 | VNN1/C1QA/CEACAM1 | 3 |
| GO:0016055 | BP | Wnt signaling pathway | 1/27 | 498/17910 | 0.533245 | 0.572263 | 0.428432 | GRB10 | 1 |
| GO:0043410 | BP | positive regulation of MAPK cascade | 1/27 | 499/17910 | 0.533968 | 0.572583 | 0.428671 | GADD45A | 1 |
| GO:0048667 | BP | cell morphogenesis involved in neuron differentiation | 1/27 | 500/17910 | 0.534691 | 0.572583 | 0.428671 | GRB10 | 1 |
| GO:0198738 | BP | cell-cell signaling by wnt | 1/27 | 500/17910 | 0.534691 | 0.572583 | 0.428671 | GRB10 | 1 |
| GO:0006281 | BP | DNA repair | 1/27 | 502/17910 | 0.536133 | 0.573716 | 0.42952 | GADD45A | 1 |
| GO:0019219 | BP | regulation of nucleobase-containing compound metabolic process | 6/27 | 3853/17910 | 0.539544 | 0.576953 | 0.431943 | TGFBR3/RRM2/NR3C2/DACH1/NLRC3/CCL5 | 6 |
| GO:0030335 | BP | positive regulation of cell migration | 1/27 | 516/17910 | 0.546109 | 0.58335 | 0.436733 | CCL5 | 1 |
| GO:0010556 | BP | regulation of macromolecule biosynthetic process | 6/27 | 3877/17910 | 0.546306 | 0.58335 | 0.436733 | TGFBR3/RRM2/NR3C2/DACH1/NLRC3/CCL5 | 6 |
| GO:0030003 | BP | cellular cation homeostasis | 1/27 | 518/17910 | 0.547517 | 0.584226 | 0.437388 | CCL5 | 1 |
| GO:0051962 | BP | positive regulation of nervous system development | 1/27 | 519/17910 | 0.548219 | 0.584559 | 0.437637 | C1QA | 1 |
| GO:0002009 | BP | morphogenesis of an epithelium | 1/27 | 527/17910 | 0.553801 | 0.589988 | 0.441702 | CEACAM1 | 1 |
| GO:0010035 | BP | response to inorganic substance | 1/27 | 528/17910 | 0.554494 | 0.589988 | 0.441702 | C1QA | 1 |
| GO:0010720 | BP | positive regulation of cell development | 1/27 | 528/17910 | 0.554494 | 0.589988 | 0.441702 | C1QA | 1 |
| GO:0006873 | BP | cellular ion homeostasis | 1/27 | 531/17910 | 0.556567 | 0.591352 | 0.442724 | CCL5 | 1 |
| GO:0055065 | BP | metal ion homeostasis | 1/27 | 531/17910 | 0.556567 | 0.591352 | 0.442724 | CCL5 | 1 |
| GO:0034762 | BP | regulation of transmembrane transport | 1/27 | 538/17910 | 0.561368 | 0.59603 | 0.446226 | GRB10 | 1 |
| GO:2000147 | BP | positive regulation of cell motility | 1/27 | 540/17910 | 0.56273 | 0.596744 | 0.44676 | CCL5 | 1 |
| GO:0006355 | BP | regulation of transcription, DNA-templated | 5/27 | 3266/17910 | 0.562838 | 0.596744 | 0.44676 | TGFBR3/RRM2/NR3C2/DACH1/NLRC3 | 5 |
| GO:0006820 | BP | anion transport | 1/27 | 542/17910 | 0.564089 | 0.597646 | 0.447436 | CEACAM1 | 1 |
| GO:0060429 | BP | epithelium development | 2/27 | 1251/17910 | 0.571603 | 0.605179 | 0.453075 | RAB13/CEACAM1 | 2 |
| GO:0051272 | BP | positive regulation of cellular component movement | 1/27 | 555/17910 | 0.57282 | 0.606038 | 0.453718 | CCL5 | 1 |
| GO:0007507 | BP | heart development | 1/27 | 556/17910 | 0.573484 | 0.606313 | 0.453924 | TGFBR3 | 1 |
| GO:0030036 | BP | actin cytoskeleton organization | 1/27 | 559/17910 | 0.575472 | 0.607555 | 0.454854 | RAB13 | 1 |
| GO:0048812 | BP | neuron projection morphogenesis | 1/27 | 559/17910 | 0.575472 | 0.607555 | 0.454854 | GRB10 | 1 |
| GO:0009893 | BP | positive regulation of metabolic process | 5/27 | 3313/17910 | 0.576721 | 0.608444 | 0.45552 | CDC25B/PCOLCE2/GADD45A/CCL5/GRB10 | 5 |
| GO:0009892 | BP | negative regulation of metabolic process | 5/27 | 3321/17910 | 0.579064 | 0.610487 | 0.457049 | GADD45A/RPL10A/RPL36/DACH1/CEACAM1 | 5 |
| GO:0048731 | BP | system development | 7/27 | 4670/17910 | 0.580138 | 0.611059 | 0.457478 | TGFBR3/RAB13/HPGD/VNN1/C1QA/GRB10/CEACAM1 | 7 |
| GO:0002757 | BP | immune response-activating signal transduction | 1/27 | 567/17910 | 0.580729 | 0.611059 | 0.457478 | CEACAM1 | 1 |
| GO:0009888 | BP | tissue development | 3/27 | 1964/17910 | 0.580833 | 0.611059 | 0.457478 | TGFBR3/RAB13/CEACAM1 | 3 |
| GO:0040017 | BP | positive regulation of locomotion | 1/27 | 570/17910 | 0.582684 | 0.612171 | 0.45831 | CCL5 | 1 |
| GO:0051241 | BP | negative regulation of multicellular organismal process | 2/27 | 1276/17910 | 0.582708 | 0.612171 | 0.45831 | NLRC3/CEACAM1 | 2 |
| GO:1903506 | BP | regulation of nucleic acid-templated transcription | 5/27 | 3338/17910 | 0.584026 | 0.613125 | 0.459024 | TGFBR3/RRM2/NR3C2/DACH1/NLRC3 | 5 |
| GO:0001525 | BP | angiogenesis | 1/27 | 576/17910 | 0.586569 | 0.615362 | 0.460699 | CEACAM1 | 1 |
| GO:0034654 | BP | nucleobase-containing compound biosynthetic process | 6/27 | 4029/17910 | 0.588292 | 0.616521 | 0.461567 | TGFBR3/RRM2/NR3C2/DACH1/NLRC3/CCL5 | 6 |
| GO:0097190 | BP | apoptotic signaling pathway | 1/27 | 579/17910 | 0.588498 | 0.616521 | 0.461567 | VNN1 | 1 |
| GO:0010469 | BP | regulation of signaling receptor activity | 1/27 | 588/17910 | 0.594233 | 0.621659 | 0.465413 | CCL5 | 1 |
| GO:0055080 | BP | cation homeostasis | 1/27 | 588/17910 | 0.594233 | 0.621659 | 0.465413 | CCL5 | 1 |
| GO:1905114 | BP | cell surface receptor signaling pathway involved in cell-cell signaling | 1/27 | 596/17910 | 0.599266 | 0.626486 | 0.469027 | GRB10 | 1 |
| GO:0098771 | BP | inorganic ion homeostasis | 1/27 | 600/17910 | 0.60176 | 0.628654 | 0.47065 | CCL5 | 1 |
| GO:0048513 | BP | animal organ development | 5/27 | 3403/17910 | 0.602752 | 0.629251 | 0.471097 | TGFBR3/RAB13/HPGD/VNN1/CEACAM1 | 5 |
| GO:0002764 | BP | immune response-regulating signaling pathway | 1/27 | 603/17910 | 0.603621 | 0.629719 | 0.471447 | CEACAM1 | 1 |
| GO:0018130 | BP | heterocycle biosynthetic process | 6/27 | 4089/17910 | 0.604416 | 0.630109 | 0.471739 | TGFBR3/RRM2/NR3C2/DACH1/NLRC3/CCL5 | 6 |
| GO:0019438 | BP | aromatic compound biosynthetic process | 6/27 | 4099/17910 | 0.607076 | 0.632441 | 0.473485 | TGFBR3/RRM2/NR3C2/DACH1/NLRC3/CCL5 | 6 |
| GO:0006351 | BP | transcription, DNA-templated | 5/27 | 3444/17910 | 0.614353 | 0.639577 | 0.478828 | TGFBR3/RRM2/NR3C2/DACH1/NLRC3 | 5 |
| GO:0051640 | BP | organelle localization | 1/27 | 623/17910 | 0.615813 | 0.640652 | 0.479632 | F5 | 1 |
| GO:0048589 | BP | developmental growth | 1/27 | 624/17910 | 0.616413 | 0.64083 | 0.479766 | TGFBR3 | 1 |
| GO:0000904 | BP | cell morphogenesis involved in differentiation | 1/27 | 632/17910 | 0.621181 | 0.645339 | 0.483141 | GRB10 | 1 |
| GO:0043269 | BP | regulation of ion transport | 1/27 | 639/17910 | 0.625307 | 0.649173 | 0.486012 | CCL5 | 1 |
| GO:0090407 | BP | organophosphate biosynthetic process | 1/27 | 640/17910 | 0.625892 | 0.649331 | 0.48613 | RRM2 | 1 |
| GO:0097659 | BP | nucleic acid-templated transcription | 5/27 | 3500/17910 | 0.629924 | 0.65306 | 0.488922 | TGFBR3/RRM2/NR3C2/DACH1/NLRC3 | 5 |
| GO:0009117 | BP | nucleotide metabolic process | 1/27 | 654/17910 | 0.634001 | 0.656832 | 0.491746 | RRM2 | 1 |
| GO:0030029 | BP | actin filament-based process | 1/27 | 661/17910 | 0.637992 | 0.660509 | 0.494499 | RAB13 | 1 |
| GO:0006753 | BP | nucleoside phosphate metabolic process | 1/27 | 663/17910 | 0.639124 | 0.661224 | 0.495034 | RRM2 | 1 |
| GO:0055082 | BP | cellular chemical homeostasis | 1/27 | 666/17910 | 0.640816 | 0.662516 | 0.496002 | CCL5 | 1 |
| GO:0050801 | BP | ion homeostasis | 1/27 | 669/17910 | 0.642501 | 0.663799 | 0.496962 | CCL5 | 1 |
| GO:1901362 | BP | organic cyclic compound biosynthetic process | 6/27 | 4239/17910 | 0.643441 | 0.664312 | 0.497346 | TGFBR3/RRM2/NR3C2/DACH1/NLRC3/CCL5 | 6 |
| GO:0006897 | BP | endocytosis | 1/27 | 675/17910 | 0.645848 | 0.666336 | 0.498862 | CEACAM1 | 1 |
| GO:0051173 | BP | positive regulation of nitrogen compound metabolic process | 4/27 | 2870/17910 | 0.648574 | 0.668688 | 0.500622 | CDC25B/PCOLCE2/GADD45A/CCL5 | 4 |
| GO:0055085 | BP | transmembrane transport | 2/27 | 1462/17910 | 0.659066 | 0.679038 | 0.508371 | SLC2A3/GRB10 | 2 |
| GO:0007399 | BP | nervous system development | 3/27 | 2216/17910 | 0.666384 | 0.686105 | 0.513662 | RAB13/C1QA/GRB10 | 3 |
| GO:0045596 | BP | negative regulation of cell differentiation | 1/27 | 725/17910 | 0.672586 | 0.691899 | 0.517999 | CEACAM1 | 1 |
| GO:0031327 | BP | negative regulation of cellular biosynthetic process | 2/27 | 1499/17910 | 0.672936 | 0.691899 | 0.517999 | DACH1/CEACAM1 | 2 |
| GO:0061024 | BP | membrane organization | 1/27 | 730/17910 | 0.67515 | 0.693698 | 0.519346 | F5 | 1 |
| GO:0000122 | BP | negative regulation of transcription by RNA polymerase II | 1/27 | 735/17910 | 0.677695 | 0.695835 | 0.520946 | DACH1 | 1 |
| GO:0009890 | BP | negative regulation of biosynthetic process | 2/27 | 1522/17910 | 0.68134 | 0.699098 | 0.523389 | DACH1/CEACAM1 | 2 |
| GO:0051172 | BP | negative regulation of nitrogen compound metabolic process | 3/27 | 2271/17910 | 0.68342 | 0.700751 | 0.524626 | GADD45A/DACH1/CEACAM1 | 3 |
| GO:0006811 | BP | ion transport | 2/27 | 1533/17910 | 0.6853 | 0.701657 | 0.525305 | CCL5/CEACAM1 | 2 |
| GO:0010629 | BP | negative regulation of gene expression | 3/27 | 2278/17910 | 0.685545 | 0.701657 | 0.525305 | RPL10A/RPL36/DACH1 | 3 |
| GO:0098657 | BP | import into cell | 1/27 | 751/17910 | 0.685711 | 0.701657 | 0.525305 | CEACAM1 | 1 |
| GO:0031325 | BP | positive regulation of cellular metabolic process | 4/27 | 3007/17910 | 0.687039 | 0.702536 | 0.525963 | CDC25B/PCOLCE2/GADD45A/CCL5 | 4 |
| GO:0050767 | BP | regulation of neurogenesis | 1/27 | 768/17910 | 0.694017 | 0.709186 | 0.530942 | C1QA | 1 |
| GO:0010604 | BP | positive regulation of macromolecule metabolic process | 4/27 | 3041/17910 | 0.696159 | 0.71089 | 0.532217 | CDC25B/PCOLCE2/GADD45A/CCL5 | 4 |
| GO:0006974 | BP | cellular response to DNA damage stimulus | 1/27 | 791/17910 | 0.704918 | 0.719344 | 0.538546 | GADD45A | 1 |
| GO:0030163 | BP | protein catabolic process | 1/27 | 799/17910 | 0.708622 | 0.722606 | 0.540989 | CEACAM1 | 1 |
| GO:0030001 | BP | metal ion transport | 1/27 | 800/17910 | 0.709082 | 0.722606 | 0.540989 | CCL5 | 1 |
| GO:0019725 | BP | cellular homeostasis | 1/27 | 814/17910 | 0.715446 | 0.728595 | 0.545472 | CCL5 | 1 |
| GO:0006357 | BP | regulation of transcription by RNA polymerase II | 3/27 | 2420/17910 | 0.726539 | 0.73939 | 0.553554 | TGFBR3/NR3C2/DACH1 | 3 |
| GO:0031324 | BP | negative regulation of cellular metabolic process | 3/27 | 2432/17910 | 0.72982 | 0.742223 | 0.555675 | GADD45A/DACH1/CEACAM1 | 3 |
| GO:0051960 | BP | regulation of nervous system development | 1/27 | 870/17910 | 0.739587 | 0.751645 | 0.562729 | C1QA | 1 |
| GO:0044087 | BP | regulation of cellular component biogenesis | 1/27 | 891/17910 | 0.748121 | 0.759802 | 0.568836 | NLRC3 | 1 |
| GO:0060284 | BP | regulation of cell development | 1/27 | 893/17910 | 0.748919 | 0.760097 | 0.569057 | C1QA | 1 |
| GO:0007417 | BP | central nervous system development | 1/27 | 935/17910 | 0.765137 | 0.776031 | 0.580986 | C1QA | 1 |
| GO:0051050 | BP | positive regulation of transport | 1/27 | 942/17910 | 0.76774 | 0.778069 | 0.582512 | CCL5 | 1 |
| GO:0006366 | BP | transcription by RNA polymerase II | 3/27 | 2581/17910 | 0.768187 | 0.778069 | 0.582512 | TGFBR3/NR3C2/DACH1 | 3 |
| GO:0051093 | BP | negative regulation of developmental process | 1/27 | 1017/17910 | 0.793942 | 0.803611 | 0.601634 | CEACAM1 | 1 |
| GO:0033554 | BP | cellular response to stress | 2/27 | 1914/17910 | 0.800172 | 0.809369 | 0.605945 | GADD45A/VNN1 | 2 |
| GO:0006812 | BP | cation transport | 1/27 | 1038/17910 | 0.800753 | 0.80941 | 0.605975 | CCL5 | 1 |
| GO:0045892 | BP | negative regulation of transcription, DNA-templated | 1/27 | 1083/17910 | 0.814625 | 0.822876 | 0.616057 | DACH1 | 1 |
| GO:0019637 | BP | organophosphate metabolic process | 1/27 | 1090/17910 | 0.816697 | 0.824412 | 0.617207 | RRM2 | 1 |
| GO:0051276 | BP | chromosome organization | 1/27 | 1097/17910 | 0.818747 | 0.825925 | 0.618339 | PRC1 | 1 |
| GO:0018193 | BP | peptidyl-amino acid modification | 1/27 | 1107/17910 | 0.821638 | 0.828282 | 0.620104 | CCL5 | 1 |
| GO:0010608 | BP | posttranscriptional regulation of gene expression | 1/27 | 1129/17910 | 0.827842 | 0.833974 | 0.624366 | CCL5 | 1 |
| GO:1903507 | BP | negative regulation of nucleic acid-templated transcription | 1/27 | 1137/17910 | 0.830046 | 0.83562 | 0.625598 | DACH1 | 1 |
| GO:1902679 | BP | negative regulation of RNA biosynthetic process | 1/27 | 1139/17910 | 0.830593 | 0.83562 | 0.625598 | DACH1 | 1 |
| GO:0003008 | BP | system process | 2/27 | 2118/17910 | 0.84569 | 0.850236 | 0.636541 | F5/CEACAM1 | 2 |
| GO:0051253 | BP | negative regulation of RNA metabolic process | 1/27 | 1214/17910 | 0.849916 | 0.853911 | 0.639292 | DACH1 | 1 |
| GO:0045934 | BP | negative regulation of nucleobase-containing compound metabolic process | 1/27 | 1342/17910 | 0.878095 | 0.881239 | 0.659751 | DACH1 | 1 |
| GO:2000113 | BP | negative regulation of cellular macromolecule biosynthetic process | 1/27 | 1343/17910 | 0.878294 | 0.881239 | 0.659751 | DACH1 | 1 |
| GO:0010558 | BP | negative regulation of macromolecule biosynthetic process | 1/27 | 1432/17910 | 0.89478 | 0.897178 | 0.671684 | DACH1 | 1 |
| GO:0010557 | BP | positive regulation of macromolecule biosynthetic process | 1/27 | 1698/17910 | 0.932218 | 0.934091 | 0.69932 | CCL5 | 1 |
| GO:0031328 | BP | positive regulation of cellular biosynthetic process | 1/27 | 1809/17910 | 0.943703 | 0.944966 | 0.707461 | CCL5 | 1 |
| GO:0010628 | BP | positive regulation of gene expression | 1/27 | 1827/17910 | 0.945379 | 0.946011 | 0.708244 | CCL5 | 1 |
| GO:0009891 | BP | positive regulation of biosynthetic process | 1/27 | 1842/17910 | 0.946739 | 0.946739 | 0.708788 | CCL5 | 1 |
| GO:0070821 | CC | tertiary granule membrane | 3/27 | 73/18675 | 0.000157 | 0.027733 | 0.019297 | MGAM/SLC2A3/CEACAM1 | 3 |
| GO:0030667 | CC | secretory granule membrane | 4/27 | 293/18675 | 0.000784 | 0.038138 | 0.026536 | MGAM/SLC2A3/VNN1/CEACAM1 | 4 |
| GO:0042788 | CC | polysomal ribosome | 2/27 | 30/18675 | 0.000854 | 0.038138 | 0.026536 | RPL10A/RPL36 | 2 |
| GO:0030141 | CC | secretory granule | 6/27 | 806/18675 | 0.000862 | 0.038138 | 0.026536 | RAB13/F5/MGAM/SLC2A3/VNN1/CEACAM1 | 6 |
| GO:0098588 | CC | bounding membrane of organelle | 9/27 | 1955/18675 | 0.001202 | 0.040465 | 0.028156 | ST6GALNAC3/RAB13/F5/DPP4/MGAM/SLC2A3/VNN1/HEPACAM2/CEACAM1 | 9 |
| GO:0070820 | CC | tertiary granule | 3/27 | 164/18675 | 0.001666 | 0.040465 | 0.028156 | MGAM/SLC2A3/CEACAM1 | 3 |
| GO:0099503 | CC | secretory vesicle | 6/27 | 923/18675 | 0.001736 | 0.040465 | 0.028156 | RAB13/F5/MGAM/SLC2A3/VNN1/CEACAM1 | 6 |
| GO:0022625 | CC | cytosolic large ribosomal subunit | 2/27 | 52/18675 | 0.002553 | 0.040465 | 0.028156 | RPL10A/RPL36 | 2 |
| GO:1903561 | CC | extracellular vesicle | 9/27 | 2185/18675 | 0.00264 | 0.040465 | 0.028156 | TGFBR3/RAB13/F5/RPL10A/DPP4/MGAM/HPGD/SLC2A3/CEACAM1 | 9 |
| GO:0043230 | CC | extracellular organelle | 9/27 | 2187/18675 | 0.002657 | 0.040465 | 0.028156 | TGFBR3/RAB13/F5/RPL10A/DPP4/MGAM/HPGD/SLC2A3/CEACAM1 | 9 |
| GO:0016328 | CC | lateral plasma membrane | 2/27 | 58/18675 | 0.003165 | 0.040465 | 0.028156 | RAB13/CEACAM1 | 2 |
| GO:0044445 | CC | cytosolic part | 3/27 | 207/18675 | 0.003227 | 0.040465 | 0.028156 | RPL10A/RRM2/RPL36 | 3 |
| GO:0005844 | CC | polysome | 2/27 | 60/18675 | 0.003383 | 0.040465 | 0.028156 | RPL10A/RPL36 | 2 |
| GO:0101003 | CC | ficolin-1-rich granule membrane | 2/27 | 61/18675 | 0.003495 | 0.040465 | 0.028156 | MGAM/SLC2A3 | 2 |
| GO:0005576 | CC | extracellular region | 13/27 | 4295/18675 | 0.003613 | 0.040465 | 0.028156 | TGFBR3/PCOLCE2/RAB13/F5/RPL10A/DPP4/MGAM/HPGD/SLC2A3/VNN1/CCL5/C1QA/CEACAM1 | 13 |
| GO:0030659 | CC | cytoplasmic vesicle membrane | 5/27 | 735/18675 | 0.003658 | 0.040465 | 0.028156 | RAB13/MGAM/SLC2A3/VNN1/CEACAM1 | 5 |
| GO:0012506 | CC | vesicle membrane | 5/27 | 754/18675 | 0.004079 | 0.042475 | 0.029554 | RAB13/MGAM/SLC2A3/VNN1/CEACAM1 | 5 |
| GO:0034673 | CC | inhibin-betaglycan-ActRII complex | 1/27 | 3/18675 | 0.004331 | 0.042591 | 0.029635 | TGFBR3 | 1 |
| GO:0046581 | CC | intercellular canaliculus | 1/27 | 5/18675 | 0.007209 | 0.063565 | 0.044229 | DPP4 | 1 |
| GO:0035579 | CC | specific granule membrane | 2/27 | 91/18675 | 0.007615 | 0.063565 | 0.044229 | SLC2A3/CEACAM1 | 2 |
| GO:0005615 | CC | extracellular space | 10/27 | 3051/18675 | 0.007681 | 0.063565 | 0.044229 | TGFBR3/RAB13/F5/RPL10A/DPP4/MGAM/HPGD/SLC2A3/C1QA/CEACAM1 | 10 |
| GO:0022626 | CC | cytosolic ribosome | 2/27 | 95/18675 | 0.008274 | 0.063565 | 0.044229 | RPL10A/RPL36 | 2 |
| GO:0043235 | CC | receptor complex | 3/27 | 292/18675 | 0.008384 | 0.063565 | 0.044229 | TGFBR3/NR3C2/CEACAM1 | 3 |
| GO:0071438 | CC | invadopodium membrane | 1/27 | 6/18675 | 0.008645 | 0.063565 | 0.044229 | DPP4 | 1 |
| GO:0016324 | CC | apical plasma membrane | 3/27 | 300/18675 | 0.009025 | 0.063565 | 0.044229 | DPP4/MGAM/CEACAM1 | 3 |
| GO:0070062 | CC | extracellular exosome | 8/27 | 2162/18675 | 0.009337 | 0.063565 | 0.044229 | TGFBR3/RAB13/RPL10A/DPP4/MGAM/HPGD/SLC2A3/CEACAM1 | 8 |
| GO:0031253 | CC | cell projection membrane | 3/27 | 317/18675 | 0.010482 | 0.066909 | 0.046555 | DPP4/PLEKHO1/CEACAM1 | 3 |
| GO:0015934 | CC | large ribosomal subunit | 2/27 | 108/18675 | 0.010584 | 0.066909 | 0.046555 | RPL10A/RPL36 | 2 |
| GO:0005819 | CC | spindle | 3/27 | 326/18675 | 0.011306 | 0.067321 | 0.046843 | CDC25B/PRC1/HEPACAM2 | 3 |
| GO:0032593 | CC | insulin-responsive compartment | 1/27 | 8/18675 | 0.01151 | 0.067321 | 0.046843 | RAB13 | 1 |
| GO:0031090 | CC | organelle membrane | 9/27 | 2734/18675 | 0.011791 | 0.067321 | 0.046843 | ST6GALNAC3/RAB13/F5/DPP4/MGAM/SLC2A3/VNN1/HEPACAM2/CEACAM1 | 9 |
| GO:0044459 | CC | plasma membrane part | 8/27 | 2283/18675 | 0.012835 | 0.069404 | 0.048292 | TGFBR3/RAB13/DPP4/PLEKHO1/MGAM/HPGD/SLC2A3/CEACAM1 | 8 |
| GO:0070938 | CC | contractile ring | 1/27 | 9/18675 | 0.01294 | 0.069404 | 0.048292 | PRC1 | 1 |
| GO:0044421 | CC | extracellular region part | 10/27 | 3312/18675 | 0.013652 | 0.071072 | 0.049452 | TGFBR3/RAB13/F5/RPL10A/DPP4/MGAM/HPGD/SLC2A3/C1QA/CEACAM1 | 10 |
| GO:0045177 | CC | apical part of cell | 3/27 | 365/18675 | 0.015302 | 0.076553 | 0.053266 | DPP4/MGAM/CEACAM1 | 3 |
| GO:0044433 | CC | cytoplasmic vesicle part | 6/27 | 1450/18675 | 0.01557 | 0.076553 | 0.053266 | RAB13/F5/MGAM/SLC2A3/VNN1/CEACAM1 | 6 |
| GO:0031252 | CC | cell leading edge | 3/27 | 384/18675 | 0.017505 | 0.083741 | 0.058267 | RAB13/DPP4/PLEKHO1 | 3 |
| GO:0000922 | CC | spindle pole | 2/27 | 152/18675 | 0.02022 | 0.092082 | 0.064071 | CDC25B/PRC1 | 2 |
| GO:0031256 | CC | leading edge membrane | 2/27 | 153/18675 | 0.02047 | 0.092082 | 0.064071 | DPP4/PLEKHO1 | 2 |
| GO:0098590 | CC | plasma membrane region | 5/27 | 1130/18675 | 0.021312 | 0.092082 | 0.064071 | DPP4/PLEKHO1/MGAM/HPGD/CEACAM1 | 5 |
| GO:0005911 | CC | cell-cell junction | 3/27 | 414/18675 | 0.02133 | 0.092082 | 0.064071 | RAB13/DPP4/CEACAM1 | 3 |
| GO:0042581 | CC | specific granule | 2/27 | 160/18675 | 0.022254 | 0.093737 | 0.065223 | SLC2A3/CEACAM1 | 2 |
| GO:0071437 | CC | invadopodium | 1/27 | 16/18675 | 0.022892 | 0.093737 | 0.065223 | DPP4 | 1 |
| GO:0098805 | CC | whole membrane | 6/27 | 1586/18675 | 0.023302 | 0.093737 | 0.065223 | RAB13/DPP4/MGAM/SLC2A3/VNN1/CEACAM1 | 6 |
| GO:0042101 | CC | T cell receptor complex | 1/27 | 17/18675 | 0.024306 | 0.094841 | 0.065991 | CEACAM1 | 1 |
| GO:0098791 | CC | Golgi subcompartment | 4/27 | 780/18675 | 0.02467 | 0.094841 | 0.065991 | ST6GALNAC3/RAB13/F5/HEPACAM2 | 4 |
| GO:0030496 | CC | midbody | 2/27 | 171/18675 | 0.025184 | 0.094841 | 0.065991 | PRC1/HEPACAM2 | 2 |
| GO:0044391 | CC | ribosomal subunit | 2/27 | 173/18675 | 0.025733 | 0.094889 | 0.066025 | RPL10A/RPL36 | 2 |
| GO:0101002 | CC | ficolin-1-rich granule | 2/27 | 185/18675 | 0.029128 | 0.104245 | 0.072534 | MGAM/SLC2A3 | 2 |
| GO:0030027 | CC | lamellipodium | 2/27 | 191/18675 | 0.030889 | 0.104245 | 0.072534 | RAB13/DPP4 | 2 |
| GO:0031258 | CC | lamellipodium membrane | 1/27 | 22/18675 | 0.031346 | 0.104245 | 0.072534 | DPP4 | 1 |
| GO:0031982 | CC | vesicle | 10/27 | 3756/18675 | 0.031433 | 0.104245 | 0.072534 | TGFBR3/RAB13/F5/RPL10A/DPP4/MGAM/HPGD/SLC2A3/VNN1/CEACAM1 | 10 |
| GO:0031410 | CC | cytoplasmic vesicle | 7/27 | 2179/18675 | 0.031593 | 0.104245 | 0.072534 | RAB13/F5/DPP4/MGAM/SLC2A3/VNN1/CEACAM1 | 7 |
| GO:0097708 | CC | intracellular vesicle | 7/27 | 2182/18675 | 0.031804 | 0.104245 | 0.072534 | RAB13/F5/DPP4/MGAM/SLC2A3/VNN1/CEACAM1 | 7 |
| GO:0031528 | CC | microvillus membrane | 1/27 | 23/18675 | 0.032748 | 0.105391 | 0.073332 | CEACAM1 | 1 |
| GO:0016323 | CC | basolateral plasma membrane | 2/27 | 201/18675 | 0.033918 | 0.105468 | 0.073386 | HPGD/CEACAM1 | 2 |
| GO:0005912 | CC | adherens junction | 3/27 | 496/18675 | 0.033964 | 0.105468 | 0.073386 | RPL10A/DPP4/CEACAM1 | 3 |
| GO:0070161 | CC | anchoring junction | 3/27 | 514/18675 | 0.037164 | 0.113093 | 0.078691 | RPL10A/DPP4/CEACAM1 | 3 |
| GO:0044431 | CC | Golgi apparatus part | 4/27 | 891/18675 | 0.037698 | 0.113093 | 0.078691 | ST6GALNAC3/RAB13/F5/HEPACAM2 | 4 |
| GO:0009925 | CC | basal plasma membrane | 1/27 | 28/18675 | 0.03973 | 0.117203 | 0.081551 | CEACAM1 | 1 |
| GO:0005840 | CC | ribosome | 2/27 | 252/18675 | 0.051031 | 0.148074 | 0.103031 | RPL10A/RPL36 | 2 |
| GO:0045178 | CC | basal part of cell | 1/27 | 45/18675 | 0.063105 | 0.180155 | 0.125353 | CEACAM1 | 1 |
| GO:0030139 | CC | endocytic vesicle | 2/27 | 289/18675 | 0.065012 | 0.182654 | 0.127092 | RAB13/DPP4 | 2 |
| GO:0009986 | CC | cell surface | 3/27 | 667/18675 | 0.070339 | 0.191944 | 0.133556 | TGFBR3/DPP4/CEACAM1 | 3 |
| GO:0015630 | CC | microtubule cytoskeleton | 4/27 | 1102/18675 | 0.071804 | 0.191944 | 0.133556 | CDC25B/PRC1/NLRC3/HEPACAM2 | 4 |
| GO:0005829 | CC | cytosol | 11/27 | 4909/18675 | 0.072458 | 0.191944 | 0.133556 | CDC25B/RAB13/PRC1/RPL10A/RRM2/RPL36/NR3C2/IFIT1/HPGD/NLRC3/GRB10 | 11 |
| GO:0005815 | CC | microtubule organizing center | 3/27 | 681/18675 | 0.073885 | 0.191944 | 0.133556 | CDC25B/NLRC3/HEPACAM2 | 3 |
| GO:0005876 | CC | spindle microtubule | 1/27 | 53/18675 | 0.073915 | 0.191944 | 0.133556 | PRC1 | 1 |
| GO:0031984 | CC | organelle subcompartment | 5/27 | 1592/18675 | 0.074826 | 0.191944 | 0.133556 | ST6GALNAC3/RAB13/F5/NR3C2/HEPACAM2 | 5 |
| GO:0000139 | CC | Golgi membrane | 3/27 | 691/18675 | 0.076467 | 0.193353 | 0.134536 | ST6GALNAC3/F5/HEPACAM2 | 3 |
| GO:0035577 | CC | azurophil granule membrane | 1/27 | 58/18675 | 0.08061 | 0.200957 | 0.139827 | VNN1 | 1 |
| GO:0005765 | CC | lysosomal membrane | 2/27 | 344/18675 | 0.087851 | 0.213009 | 0.148213 | DPP4/VNN1 | 2 |
| GO:0098852 | CC | lytic vacuole membrane | 2/27 | 344/18675 | 0.087851 | 0.213009 | 0.148213 | DPP4/VNN1 | 2 |
| GO:0031093 | CC | platelet alpha granule lumen | 1/27 | 67/18675 | 0.092543 | 0.218779 | 0.152228 | F5 | 1 |
| GO:0018995 | CC | host | 1/27 | 69/18675 | 0.095175 | 0.218779 | 0.152228 | IFIT1 | 1 |
| GO:0030134 | CC | COPII-coated ER to Golgi transport vesicle | 1/27 | 69/18675 | 0.095175 | 0.218779 | 0.152228 | F5 | 1 |
| GO:0043657 | CC | host cell | 1/27 | 69/18675 | 0.095175 | 0.218779 | 0.152228 | IFIT1 | 1 |
| GO:0033116 | CC | endoplasmic reticulum-Golgi intermediate compartment membrane | 1/27 | 70/18675 | 0.096488 | 0.218953 | 0.152349 | F5 | 1 |
| GO:0030054 | CC | cell junction | 4/27 | 1247/18675 | 0.102216 | 0.220932 | 0.153726 | RAB13/RPL10A/DPP4/CEACAM1 | 4 |
| GO:0044215 | CC | other organism | 1/27 | 75/18675 | 0.103026 | 0.220932 | 0.153726 | IFIT1 | 1 |
| GO:0044216 | CC | other organism cell | 1/27 | 75/18675 | 0.103026 | 0.220932 | 0.153726 | IFIT1 | 1 |
| GO:0044217 | CC | other organism part | 1/27 | 75/18675 | 0.103026 | 0.220932 | 0.153726 | IFIT1 | 1 |
| GO:0055038 | CC | recycling endosome membrane | 1/27 | 76/18675 | 0.104328 | 0.220932 | 0.153726 | RAB13 | 1 |
| GO:0005774 | CC | vacuolar membrane | 2/27 | 382/18675 | 0.104849 | 0.220932 | 0.153726 | DPP4/VNN1 | 2 |
| GO:0005902 | CC | microvillus | 1/27 | 80/18675 | 0.109518 | 0.228054 | 0.158682 | CEACAM1 | 1 |
| GO:0005925 | CC | focal adhesion | 2/27 | 397/18675 | 0.111795 | 0.229348 | 0.159582 | RPL10A/DPP4 | 2 |
| GO:0005924 | CC | cell-substrate adherens junction | 2/27 | 399/18675 | 0.11273 | 0.229348 | 0.159582 | RPL10A/DPP4 | 2 |
| GO:0030055 | CC | cell-substrate junction | 2/27 | 404/18675 | 0.115078 | 0.231464 | 0.161055 | RPL10A/DPP4 | 2 |
| GO:0005581 | CC | collagen trimer | 1/27 | 86/18675 | 0.117249 | 0.23318 | 0.162248 | C1QA | 1 |
| GO:0031091 | CC | platelet alpha granule | 1/27 | 91/18675 | 0.123641 | 0.240489 | 0.167334 | F5 | 1 |
| GO:0032587 | CC | ruffle membrane | 1/27 | 91/18675 | 0.123641 | 0.240489 | 0.167334 | PLEKHO1 | 1 |
| GO:1990204 | CC | oxidoreductase complex | 1/27 | 93/18675 | 0.126186 | 0.242771 | 0.168922 | RRM2 | 1 |
| GO:0012505 | CC | endomembrane system | 9/27 | 4213/18675 | 0.134685 | 0.256336 | 0.178361 | ST6GALNAC3/RAB13/F5/NR3C2/MGAM/SLC2A3/VNN1/HEPACAM2/CEACAM1 | 9 |
| GO:0042995 | CC | cell projection | 5/27 | 1917/18675 | 0.137161 | 0.256647 | 0.178577 | RAB13/DPP4/PLEKHO1/SLC2A3/CEACAM1 | 5 |
| GO:0098797 | CC | plasma membrane protein complex | 2/27 | 451/18675 | 0.137748 | 0.256647 | 0.178577 | TGFBR3/CEACAM1 | 2 |
| GO:0031012 | CC | extracellular matrix | 2/27 | 468/18675 | 0.146187 | 0.269533 | 0.187543 | TGFBR3/C1QA | 2 |
| GO:0005813 | CC | centrosome | 2/27 | 473/18675 | 0.148691 | 0.271322 | 0.188788 | CDC25B/HEPACAM2 | 2 |
| GO:0005794 | CC | Golgi apparatus | 4/27 | 1441/18675 | 0.150935 | 0.272607 | 0.189682 | ST6GALNAC3/RAB13/F5/HEPACAM2 | 4 |
| GO:0005793 | CC | endoplasmic reticulum-Golgi intermediate compartment | 1/27 | 115/18675 | 0.153712 | 0.274819 | 0.191221 | F5 | 1 |
| GO:0005923 | CC | bicellular tight junction | 1/27 | 118/18675 | 0.1574 | 0.278599 | 0.193851 | RAB13 | 1 |
| GO:0070160 | CC | tight junction | 1/27 | 123/18675 | 0.163513 | 0.286552 | 0.199385 | RAB13 | 1 |
| GO:0043204 | CC | perikaryon | 1/27 | 127/18675 | 0.168372 | 0.292176 | 0.203298 | SLC2A3 | 1 |
| GO:0043296 | CC | apical junction complex | 1/27 | 134/18675 | 0.176811 | 0.30384 | 0.211414 | RAB13 | 1 |
| GO:0044430 | CC | cytoskeletal part | 4/27 | 1543/18675 | 0.17974 | 0.305904 | 0.21285 | CDC25B/PRC1/NLRC3/HEPACAM2 | 4 |
| GO:0044437 | CC | vacuolar part | 2/27 | 538/18675 | 0.181995 | 0.306791 | 0.213467 | DPP4/VNN1 | 2 |
| GO:0005798 | CC | Golgi-associated vesicle | 1/27 | 145/18675 | 0.189905 | 0.317106 | 0.220644 | F5 | 1 |
| GO:0098794 | CC | postsynapse | 2/27 | 568/18675 | 0.197754 | 0.323382 | 0.225011 | RPL10A/C1QA | 2 |
| GO:0098802 | CC | plasma membrane receptor complex | 1/27 | 153/18675 | 0.199302 | 0.323382 | 0.225011 | CEACAM1 | 1 |
| GO:0005766 | CC | primary lysosome | 1/27 | 155/18675 | 0.201635 | 0.323382 | 0.225011 | VNN1 | 1 |
| GO:0042582 | CC | azurophil granule | 1/27 | 155/18675 | 0.201635 | 0.323382 | 0.225011 | VNN1 | 1 |
| GO:0031225 | CC | anchored component of membrane | 1/27 | 156/18675 | 0.202799 | 0.323382 | 0.225011 | VNN1 | 1 |
| GO:0055037 | CC | recycling endosome | 1/27 | 164/18675 | 0.212051 | 0.335117 | 0.233177 | RAB13 | 1 |
| GO:0001726 | CC | ruffle | 1/27 | 168/18675 | 0.216639 | 0.339337 | 0.236113 | PLEKHO1 | 1 |
| GO:0005887 | CC | integral component of plasma membrane | 3/27 | 1158/18675 | 0.23302 | 0.361794 | 0.251739 | TGFBR3/SLC2A3/CEACAM1 | 3 |
| GO:0030658 | CC | transport vesicle membrane | 1/27 | 188/18675 | 0.239193 | 0.36815 | 0.256161 | CEACAM1 | 1 |
| GO:0000323 | CC | lytic vacuole | 2/27 | 659/18675 | 0.246495 | 0.372058 | 0.258881 | DPP4/VNN1 | 2 |
| GO:0005764 | CC | lysosome | 2/27 | 659/18675 | 0.246495 | 0.372058 | 0.258881 | DPP4/VNN1 | 2 |
| GO:0005802 | CC | trans-Golgi network | 1/27 | 196/18675 | 0.248039 | 0.372058 | 0.258881 | RAB13 | 1 |
| GO:0009897 | CC | external side of plasma membrane | 1/27 | 199/18675 | 0.25133 | 0.372326 | 0.259067 | TGFBR3 | 1 |
| GO:0098858 | CC | actin-based cell projection | 1/27 | 200/18675 | 0.252424 | 0.372326 | 0.259067 | CEACAM1 | 1 |
| GO:0031226 | CC | intrinsic component of plasma membrane | 3/27 | 1231/18675 | 0.26175 | 0.382891 | 0.266418 | TGFBR3/SLC2A3/CEACAM1 | 3 |
| GO:0120025 | CC | plasma membrane bounded cell projection | 4/27 | 1854/18675 | 0.277542 | 0.402663 | 0.280176 | RAB13/DPP4/PLEKHO1/CEACAM1 | 4 |
| GO:0005773 | CC | vacuole | 2/27 | 737/18675 | 0.288786 | 0.414356 | 0.288312 | DPP4/VNN1 | 2 |
| GO:0044463 | CC | cell projection part | 3/27 | 1308/18675 | 0.292625 | 0.414356 | 0.288312 | DPP4/PLEKHO1/CEACAM1 | 3 |
| GO:0120038 | CC | plasma membrane bounded cell projection part | 3/27 | 1308/18675 | 0.292625 | 0.414356 | 0.288312 | DPP4/PLEKHO1/CEACAM1 | 3 |
| GO:0030135 | CC | coated vesicle | 1/27 | 261/18675 | 0.316329 | 0.444366 | 0.309193 | F5 | 1 |
| GO:0005856 | CC | cytoskeleton | 4/27 | 2041/18675 | 0.340819 | 0.475 | 0.330508 | CDC25B/PRC1/NLRC3/HEPACAM2 | 4 |
| GO:0014069 | CC | postsynaptic density | 1/27 | 290/18675 | 0.344832 | 0.476838 | 0.331787 | RPL10A | 1 |
| GO:0032279 | CC | asymmetric synapse | 1/27 | 294/18675 | 0.348672 | 0.478411 | 0.332882 | RPL10A | 1 |
| GO:0044456 | CC | synapse part | 2/27 | 861/18675 | 0.355601 | 0.47974 | 0.333807 | RPL10A/C1QA | 2 |
| GO:0005788 | CC | endoplasmic reticulum lumen | 1/27 | 306/18675 | 0.360064 | 0.47974 | 0.333807 | F5 | 1 |
| GO:0045121 | CC | membrane raft | 1/27 | 309/18675 | 0.362882 | 0.47974 | 0.333807 | DPP4 | 1 |
| GO:0098857 | CC | membrane microdomain | 1/27 | 310/18675 | 0.363819 | 0.47974 | 0.333807 | DPP4 | 1 |
| GO:0098984 | CC | neuron to neuron synapse | 1/27 | 315/18675 | 0.368482 | 0.47974 | 0.333807 | RPL10A | 1 |
| GO:0099572 | CC | postsynaptic specialization | 1/27 | 316/18675 | 0.369411 | 0.47974 | 0.333807 | RPL10A | 1 |
| GO:0097458 | CC | neuron part | 3/27 | 1500/18675 | 0.370686 | 0.47974 | 0.333807 | RAB13/RPL10A/SLC2A3 | 3 |
| GO:0034774 | CC | secretory granule lumen | 1/27 | 321/18675 | 0.374035 | 0.47974 | 0.333807 | F5 | 1 |
| GO:0098589 | CC | membrane region | 1/27 | 321/18675 | 0.374035 | 0.47974 | 0.333807 | DPP4 | 1 |
| GO:0060205 | CC | cytoplasmic vesicle lumen | 1/27 | 338/18675 | 0.389513 | 0.493592 | 0.343445 | F5 | 1 |
| GO:0031983 | CC | vesicle lumen | 1/27 | 339/18675 | 0.390412 | 0.493592 | 0.343445 | F5 | 1 |
| GO:0016021 | CC | integral component of membrane | 8/27 | 4821/18675 | 0.394967 | 0.495809 | 0.344988 | TGFBR3/ST6GALNAC3/DPP4/MGAM/SLC2A3/VNN1/HEPACAM2/CEACAM1 | 8 |
| GO:0030133 | CC | transport vesicle | 1/27 | 349/18675 | 0.399331 | 0.496193 | 0.345254 | CEACAM1 | 1 |
| GO:0098796 | CC | membrane protein complex | 2/27 | 947/18675 | 0.400879 | 0.496193 | 0.345254 | TGFBR3/CEACAM1 | 2 |
| GO:0005874 | CC | microtubule | 1/27 | 357/18675 | 0.406376 | 0.498193 | 0.346646 | PRC1 | 1 |
| GO:0098552 | CC | side of membrane | 1/27 | 359/18675 | 0.408124 | 0.498193 | 0.346646 | TGFBR3 | 1 |
| GO:0016607 | CC | nuclear speck | 1/27 | 386/18675 | 0.431252 | 0.520087 | 0.36188 | GADD45A | 1 |
| GO:0031224 | CC | intrinsic component of membrane | 8/27 | 4976/18675 | 0.433868 | 0.520087 | 0.36188 | TGFBR3/ST6GALNAC3/DPP4/MGAM/SLC2A3/VNN1/HEPACAM2/CEACAM1 | 8 |
| GO:0032991 | CC | protein-containing complex | 8/27 | 4980/18675 | 0.434875 | 0.520087 | 0.36188 | TGFBR3/RPL10A/RRM2/RPL36/NR3C2/C1QA/GRB10/CEACAM1 | 8 |
| GO:0062023 | CC | collagen-containing extracellular matrix | 1/27 | 399/18675 | 0.442075 | 0.525149 | 0.365402 | C1QA | 1 |
| GO:0045202 | CC | synapse | 2/27 | 1091/18675 | 0.473504 | 0.558735 | 0.388772 | RPL10A/C1QA | 2 |
| GO:0043025 | CC | neuronal cell body | 1/27 | 454/18675 | 0.485708 | 0.56934 | 0.396151 | SLC2A3 | 1 |
| GO:0010008 | CC | endosome membrane | 1/27 | 464/18675 | 0.49328 | 0.574412 | 0.39968 | RAB13 | 1 |
| GO:0044440 | CC | endosomal part | 1/27 | 504/18675 | 0.522509 | 0.604471 | 0.420595 | RAB13 | 1 |
| GO:0044297 | CC | cell body | 1/27 | 522/18675 | 0.535126 | 0.615047 | 0.427954 | SLC2A3 | 1 |
| GO:0044432 | CC | endoplasmic reticulum part | 2/27 | 1289/18675 | 0.564927 | 0.645111 | 0.448873 | F5/NR3C2 | 2 |
| GO:0005654 | CC | nucleoplasm | 5/27 | 3427/18675 | 0.568946 | 0.645535 | 0.449168 | CDC25B/ST6GALNAC3/GADD45A/NR3C2/HPGD | 5 |
| GO:1990904 | CC | ribonucleoprotein complex | 2/27 | 1315/18675 | 0.57612 | 0.649511 | 0.451935 | RPL10A/RPL36 | 2 |
| GO:0099513 | CC | polymeric cytoskeletal fiber | 1/27 | 635/18675 | 0.607299 | 0.680328 | 0.473377 | PRC1 | 1 |
| GO:0048471 | CC | perinuclear region of cytoplasm | 1/27 | 705/18675 | 0.646458 | 0.7128 | 0.495971 | NLRC3 | 1 |
| GO:0031981 | CC | nuclear lumen | 6/27 | 4454/18675 | 0.651642 | 0.7128 | 0.495971 | CDC25B/ST6GALNAC3/GADD45A/RPL36/NR3C2/HPGD | 6 |
| GO:0043232 | CC | intracellular non-membrane-bounded organelle | 6/27 | 4464/18675 | 0.654042 | 0.7128 | 0.495971 | CDC25B/PRC1/RPL10A/RPL36/NLRC3/HEPACAM2 | 6 |
| GO:0043228 | CC | non-membrane-bounded organelle | 6/27 | 4473/18675 | 0.656194 | 0.7128 | 0.495971 | CDC25B/PRC1/RPL10A/RPL36/NLRC3/HEPACAM2 | 6 |
| GO:0036477 | CC | somatodendritic compartment | 1/27 | 724/18675 | 0.65642 | 0.7128 | 0.495971 | SLC2A3 | 1 |
| GO:0016604 | CC | nuclear body | 1/27 | 764/18675 | 0.676518 | 0.730144 | 0.50804 | GADD45A | 1 |
| GO:0099512 | CC | supramolecular fiber | 1/27 | 845/18675 | 0.713805 | 0.755226 | 0.525492 | PRC1 | 1 |
| GO:0099081 | CC | supramolecular polymer | 1/27 | 851/18675 | 0.716396 | 0.755226 | 0.525492 | PRC1 | 1 |
| GO:0005768 | CC | endosome | 1/27 | 852/18675 | 0.716825 | 0.755226 | 0.525492 | RAB13 | 1 |
| GO:0099080 | CC | supramolecular complex | 1/27 | 852/18675 | 0.716825 | 0.755226 | 0.525492 | PRC1 | 1 |
| GO:0044428 | CC | nuclear part | 6/27 | 4801/18675 | 0.729601 | 0.764139 | 0.531693 | CDC25B/ST6GALNAC3/GADD45A/RPL36/NR3C2/HPGD | 6 |
| GO:0005783 | CC | endoplasmic reticulum | 2/27 | 1785/18675 | 0.744467 | 0.775121 | 0.539335 | F5/NR3C2 | 2 |
| GO:0005789 | CC | endoplasmic reticulum membrane | 1/27 | 1005/18675 | 0.775671 | 0.798616 | 0.555683 | NR3C2 | 1 |
| GO:0098827 | CC | endoplasmic reticulum subcompartment | 1/27 | 1009/18675 | 0.777039 | 0.798616 | 0.555683 | NR3C2 | 1 |
| GO:0042175 | CC | nuclear outer membrane-endoplasmic reticulum membrane network | 1/27 | 1026/18675 | 0.782764 | 0.798616 | 0.555683 | NR3C2 | 1 |
| GO:0044451 | CC | nucleoplasm part | 1/27 | 1033/18675 | 0.78508 | 0.798616 | 0.555683 | GADD45A | 1 |
| GO:0043005 | CC | neuron projection | 1/27 | 1091/18675 | 0.803377 | 0.811556 | 0.564687 | RAB13 | 1 |
| GO:1902494 | CC | catalytic complex | 1/27 | 1103/18675 | 0.806971 | 0.811556 | 0.564687 | RRM2 | 1 |
| GO:0005730 | CC | nucleolus | 1/27 | 1369/18675 | 0.872171 | 0.872171 | 0.606863 | RPL36 | 1 |
| GO:0070821 | CC | tertiary granule membrane | 3/27 | 73/18675 | 0.000157 | 0.027733 | 0.019297 | MGAM/SLC2A3/CEACAM1 | 3 |
| GO:0030667 | CC | secretory granule membrane | 4/27 | 293/18675 | 0.000784 | 0.038138 | 0.026536 | MGAM/SLC2A3/VNN1/CEACAM1 | 4 |
| GO:0042788 | CC | polysomal ribosome | 2/27 | 30/18675 | 0.000854 | 0.038138 | 0.026536 | RPL10A/RPL36 | 2 |
| GO:0030141 | CC | secretory granule | 6/27 | 806/18675 | 0.000862 | 0.038138 | 0.026536 | RAB13/F5/MGAM/SLC2A3/VNN1/CEACAM1 | 6 |
| GO:0098588 | CC | bounding membrane of organelle | 9/27 | 1955/18675 | 0.001202 | 0.040465 | 0.028156 | ST6GALNAC3/RAB13/F5/DPP4/MGAM/SLC2A3/VNN1/HEPACAM2/CEACAM1 | 9 |
| GO:0070820 | CC | tertiary granule | 3/27 | 164/18675 | 0.001666 | 0.040465 | 0.028156 | MGAM/SLC2A3/CEACAM1 | 3 |
| GO:0099503 | CC | secretory vesicle | 6/27 | 923/18675 | 0.001736 | 0.040465 | 0.028156 | RAB13/F5/MGAM/SLC2A3/VNN1/CEACAM1 | 6 |
| GO:0022625 | CC | cytosolic large ribosomal subunit | 2/27 | 52/18675 | 0.002553 | 0.040465 | 0.028156 | RPL10A/RPL36 | 2 |
| GO:1903561 | CC | extracellular vesicle | 9/27 | 2185/18675 | 0.00264 | 0.040465 | 0.028156 | TGFBR3/RAB13/F5/RPL10A/DPP4/MGAM/HPGD/SLC2A3/CEACAM1 | 9 |
| GO:0043230 | CC | extracellular organelle | 9/27 | 2187/18675 | 0.002657 | 0.040465 | 0.028156 | TGFBR3/RAB13/F5/RPL10A/DPP4/MGAM/HPGD/SLC2A3/CEACAM1 | 9 |
| GO:0016328 | CC | lateral plasma membrane | 2/27 | 58/18675 | 0.003165 | 0.040465 | 0.028156 | RAB13/CEACAM1 | 2 |
| GO:0044445 | CC | cytosolic part | 3/27 | 207/18675 | 0.003227 | 0.040465 | 0.028156 | RPL10A/RRM2/RPL36 | 3 |
| GO:0005844 | CC | polysome | 2/27 | 60/18675 | 0.003383 | 0.040465 | 0.028156 | RPL10A/RPL36 | 2 |
| GO:0101003 | CC | ficolin-1-rich granule membrane | 2/27 | 61/18675 | 0.003495 | 0.040465 | 0.028156 | MGAM/SLC2A3 | 2 |
| GO:0005576 | CC | extracellular region | 13/27 | 4295/18675 | 0.003613 | 0.040465 | 0.028156 | TGFBR3/PCOLCE2/RAB13/F5/RPL10A/DPP4/MGAM/HPGD/SLC2A3/VNN1/CCL5/C1QA/CEACAM1 | 13 |
| GO:0030659 | CC | cytoplasmic vesicle membrane | 5/27 | 735/18675 | 0.003658 | 0.040465 | 0.028156 | RAB13/MGAM/SLC2A3/VNN1/CEACAM1 | 5 |
| GO:0012506 | CC | vesicle membrane | 5/27 | 754/18675 | 0.004079 | 0.042475 | 0.029554 | RAB13/MGAM/SLC2A3/VNN1/CEACAM1 | 5 |
| GO:0034673 | CC | inhibin-betaglycan-ActRII complex | 1/27 | 3/18675 | 0.004331 | 0.042591 | 0.029635 | TGFBR3 | 1 |
| GO:0046581 | CC | intercellular canaliculus | 1/27 | 5/18675 | 0.007209 | 0.063565 | 0.044229 | DPP4 | 1 |
| GO:0035579 | CC | specific granule membrane | 2/27 | 91/18675 | 0.007615 | 0.063565 | 0.044229 | SLC2A3/CEACAM1 | 2 |
| GO:0005615 | CC | extracellular space | 10/27 | 3051/18675 | 0.007681 | 0.063565 | 0.044229 | TGFBR3/RAB13/F5/RPL10A/DPP4/MGAM/HPGD/SLC2A3/C1QA/CEACAM1 | 10 |
| GO:0022626 | CC | cytosolic ribosome | 2/27 | 95/18675 | 0.008274 | 0.063565 | 0.044229 | RPL10A/RPL36 | 2 |
| GO:0043235 | CC | receptor complex | 3/27 | 292/18675 | 0.008384 | 0.063565 | 0.044229 | TGFBR3/NR3C2/CEACAM1 | 3 |
| GO:0071438 | CC | invadopodium membrane | 1/27 | 6/18675 | 0.008645 | 0.063565 | 0.044229 | DPP4 | 1 |
| GO:0016324 | CC | apical plasma membrane | 3/27 | 300/18675 | 0.009025 | 0.063565 | 0.044229 | DPP4/MGAM/CEACAM1 | 3 |
| GO:0070062 | CC | extracellular exosome | 8/27 | 2162/18675 | 0.009337 | 0.063565 | 0.044229 | TGFBR3/RAB13/RPL10A/DPP4/MGAM/HPGD/SLC2A3/CEACAM1 | 8 |
| GO:0031253 | CC | cell projection membrane | 3/27 | 317/18675 | 0.010482 | 0.066909 | 0.046555 | DPP4/PLEKHO1/CEACAM1 | 3 |
| GO:0015934 | CC | large ribosomal subunit | 2/27 | 108/18675 | 0.010584 | 0.066909 | 0.046555 | RPL10A/RPL36 | 2 |
| GO:0005819 | CC | spindle | 3/27 | 326/18675 | 0.011306 | 0.067321 | 0.046843 | CDC25B/PRC1/HEPACAM2 | 3 |
| GO:0032593 | CC | insulin-responsive compartment | 1/27 | 8/18675 | 0.01151 | 0.067321 | 0.046843 | RAB13 | 1 |
| GO:0031090 | CC | organelle membrane | 9/27 | 2734/18675 | 0.011791 | 0.067321 | 0.046843 | ST6GALNAC3/RAB13/F5/DPP4/MGAM/SLC2A3/VNN1/HEPACAM2/CEACAM1 | 9 |
| GO:0044459 | CC | plasma membrane part | 8/27 | 2283/18675 | 0.012835 | 0.069404 | 0.048292 | TGFBR3/RAB13/DPP4/PLEKHO1/MGAM/HPGD/SLC2A3/CEACAM1 | 8 |
| GO:0070938 | CC | contractile ring | 1/27 | 9/18675 | 0.01294 | 0.069404 | 0.048292 | PRC1 | 1 |
| GO:0044421 | CC | extracellular region part | 10/27 | 3312/18675 | 0.013652 | 0.071072 | 0.049452 | TGFBR3/RAB13/F5/RPL10A/DPP4/MGAM/HPGD/SLC2A3/C1QA/CEACAM1 | 10 |
| GO:0045177 | CC | apical part of cell | 3/27 | 365/18675 | 0.015302 | 0.076553 | 0.053266 | DPP4/MGAM/CEACAM1 | 3 |
| GO:0044433 | CC | cytoplasmic vesicle part | 6/27 | 1450/18675 | 0.01557 | 0.076553 | 0.053266 | RAB13/F5/MGAM/SLC2A3/VNN1/CEACAM1 | 6 |
| GO:0031252 | CC | cell leading edge | 3/27 | 384/18675 | 0.017505 | 0.083741 | 0.058267 | RAB13/DPP4/PLEKHO1 | 3 |
| GO:0000922 | CC | spindle pole | 2/27 | 152/18675 | 0.02022 | 0.092082 | 0.064071 | CDC25B/PRC1 | 2 |
| GO:0031256 | CC | leading edge membrane | 2/27 | 153/18675 | 0.02047 | 0.092082 | 0.064071 | DPP4/PLEKHO1 | 2 |
| GO:0098590 | CC | plasma membrane region | 5/27 | 1130/18675 | 0.021312 | 0.092082 | 0.064071 | DPP4/PLEKHO1/MGAM/HPGD/CEACAM1 | 5 |
| GO:0005911 | CC | cell-cell junction | 3/27 | 414/18675 | 0.02133 | 0.092082 | 0.064071 | RAB13/DPP4/CEACAM1 | 3 |
| GO:0042581 | CC | specific granule | 2/27 | 160/18675 | 0.022254 | 0.093737 | 0.065223 | SLC2A3/CEACAM1 | 2 |
| GO:0071437 | CC | invadopodium | 1/27 | 16/18675 | 0.022892 | 0.093737 | 0.065223 | DPP4 | 1 |
| GO:0098805 | CC | whole membrane | 6/27 | 1586/18675 | 0.023302 | 0.093737 | 0.065223 | RAB13/DPP4/MGAM/SLC2A3/VNN1/CEACAM1 | 6 |
| GO:0042101 | CC | T cell receptor complex | 1/27 | 17/18675 | 0.024306 | 0.094841 | 0.065991 | CEACAM1 | 1 |
| GO:0098791 | CC | Golgi subcompartment | 4/27 | 780/18675 | 0.02467 | 0.094841 | 0.065991 | ST6GALNAC3/RAB13/F5/HEPACAM2 | 4 |
| GO:0030496 | CC | midbody | 2/27 | 171/18675 | 0.025184 | 0.094841 | 0.065991 | PRC1/HEPACAM2 | 2 |
| GO:0044391 | CC | ribosomal subunit | 2/27 | 173/18675 | 0.025733 | 0.094889 | 0.066025 | RPL10A/RPL36 | 2 |
| GO:0101002 | CC | ficolin-1-rich granule | 2/27 | 185/18675 | 0.029128 | 0.104245 | 0.072534 | MGAM/SLC2A3 | 2 |
| GO:0030027 | CC | lamellipodium | 2/27 | 191/18675 | 0.030889 | 0.104245 | 0.072534 | RAB13/DPP4 | 2 |
| GO:0031258 | CC | lamellipodium membrane | 1/27 | 22/18675 | 0.031346 | 0.104245 | 0.072534 | DPP4 | 1 |
| GO:0031982 | CC | vesicle | 10/27 | 3756/18675 | 0.031433 | 0.104245 | 0.072534 | TGFBR3/RAB13/F5/RPL10A/DPP4/MGAM/HPGD/SLC2A3/VNN1/CEACAM1 | 10 |
| GO:0031410 | CC | cytoplasmic vesicle | 7/27 | 2179/18675 | 0.031593 | 0.104245 | 0.072534 | RAB13/F5/DPP4/MGAM/SLC2A3/VNN1/CEACAM1 | 7 |
| GO:0097708 | CC | intracellular vesicle | 7/27 | 2182/18675 | 0.031804 | 0.104245 | 0.072534 | RAB13/F5/DPP4/MGAM/SLC2A3/VNN1/CEACAM1 | 7 |
| GO:0031528 | CC | microvillus membrane | 1/27 | 23/18675 | 0.032748 | 0.105391 | 0.073332 | CEACAM1 | 1 |
| GO:0016323 | CC | basolateral plasma membrane | 2/27 | 201/18675 | 0.033918 | 0.105468 | 0.073386 | HPGD/CEACAM1 | 2 |
| GO:0005912 | CC | adherens junction | 3/27 | 496/18675 | 0.033964 | 0.105468 | 0.073386 | RPL10A/DPP4/CEACAM1 | 3 |
| GO:0070161 | CC | anchoring junction | 3/27 | 514/18675 | 0.037164 | 0.113093 | 0.078691 | RPL10A/DPP4/CEACAM1 | 3 |
| GO:0044431 | CC | Golgi apparatus part | 4/27 | 891/18675 | 0.037698 | 0.113093 | 0.078691 | ST6GALNAC3/RAB13/F5/HEPACAM2 | 4 |
| GO:0009925 | CC | basal plasma membrane | 1/27 | 28/18675 | 0.03973 | 0.117203 | 0.081551 | CEACAM1 | 1 |
| GO:0005840 | CC | ribosome | 2/27 | 252/18675 | 0.051031 | 0.148074 | 0.103031 | RPL10A/RPL36 | 2 |
| GO:0045178 | CC | basal part of cell | 1/27 | 45/18675 | 0.063105 | 0.180155 | 0.125353 | CEACAM1 | 1 |
| GO:0030139 | CC | endocytic vesicle | 2/27 | 289/18675 | 0.065012 | 0.182654 | 0.127092 | RAB13/DPP4 | 2 |
| GO:0009986 | CC | cell surface | 3/27 | 667/18675 | 0.070339 | 0.191944 | 0.133556 | TGFBR3/DPP4/CEACAM1 | 3 |
| GO:0015630 | CC | microtubule cytoskeleton | 4/27 | 1102/18675 | 0.071804 | 0.191944 | 0.133556 | CDC25B/PRC1/NLRC3/HEPACAM2 | 4 |
| GO:0005829 | CC | cytosol | 11/27 | 4909/18675 | 0.072458 | 0.191944 | 0.133556 | CDC25B/RAB13/PRC1/RPL10A/RRM2/RPL36/NR3C2/IFIT1/HPGD/NLRC3/GRB10 | 11 |
| GO:0005815 | CC | microtubule organizing center | 3/27 | 681/18675 | 0.073885 | 0.191944 | 0.133556 | CDC25B/NLRC3/HEPACAM2 | 3 |
| GO:0005876 | CC | spindle microtubule | 1/27 | 53/18675 | 0.073915 | 0.191944 | 0.133556 | PRC1 | 1 |
| GO:0031984 | CC | organelle subcompartment | 5/27 | 1592/18675 | 0.074826 | 0.191944 | 0.133556 | ST6GALNAC3/RAB13/F5/NR3C2/HEPACAM2 | 5 |
| GO:0000139 | CC | Golgi membrane | 3/27 | 691/18675 | 0.076467 | 0.193353 | 0.134536 | ST6GALNAC3/F5/HEPACAM2 | 3 |
| GO:0035577 | CC | azurophil granule membrane | 1/27 | 58/18675 | 0.08061 | 0.200957 | 0.139827 | VNN1 | 1 |
| GO:0005765 | CC | lysosomal membrane | 2/27 | 344/18675 | 0.087851 | 0.213009 | 0.148213 | DPP4/VNN1 | 2 |
| GO:0098852 | CC | lytic vacuole membrane | 2/27 | 344/18675 | 0.087851 | 0.213009 | 0.148213 | DPP4/VNN1 | 2 |
| GO:0031093 | CC | platelet alpha granule lumen | 1/27 | 67/18675 | 0.092543 | 0.218779 | 0.152228 | F5 | 1 |
| GO:0018995 | CC | host | 1/27 | 69/18675 | 0.095175 | 0.218779 | 0.152228 | IFIT1 | 1 |
| GO:0030134 | CC | COPII-coated ER to Golgi transport vesicle | 1/27 | 69/18675 | 0.095175 | 0.218779 | 0.152228 | F5 | 1 |
| GO:0043657 | CC | host cell | 1/27 | 69/18675 | 0.095175 | 0.218779 | 0.152228 | IFIT1 | 1 |
| GO:0033116 | CC | endoplasmic reticulum-Golgi intermediate compartment membrane | 1/27 | 70/18675 | 0.096488 | 0.218953 | 0.152349 | F5 | 1 |
| GO:0030054 | CC | cell junction | 4/27 | 1247/18675 | 0.102216 | 0.220932 | 0.153726 | RAB13/RPL10A/DPP4/CEACAM1 | 4 |
| GO:0044215 | CC | other organism | 1/27 | 75/18675 | 0.103026 | 0.220932 | 0.153726 | IFIT1 | 1 |
| GO:0044216 | CC | other organism cell | 1/27 | 75/18675 | 0.103026 | 0.220932 | 0.153726 | IFIT1 | 1 |
| GO:0044217 | CC | other organism part | 1/27 | 75/18675 | 0.103026 | 0.220932 | 0.153726 | IFIT1 | 1 |
| GO:0055038 | CC | recycling endosome membrane | 1/27 | 76/18675 | 0.104328 | 0.220932 | 0.153726 | RAB13 | 1 |
| GO:0005774 | CC | vacuolar membrane | 2/27 | 382/18675 | 0.104849 | 0.220932 | 0.153726 | DPP4/VNN1 | 2 |
| GO:0005902 | CC | microvillus | 1/27 | 80/18675 | 0.109518 | 0.228054 | 0.158682 | CEACAM1 | 1 |
| GO:0005925 | CC | focal adhesion | 2/27 | 397/18675 | 0.111795 | 0.229348 | 0.159582 | RPL10A/DPP4 | 2 |
| GO:0005924 | CC | cell-substrate adherens junction | 2/27 | 399/18675 | 0.11273 | 0.229348 | 0.159582 | RPL10A/DPP4 | 2 |
| GO:0030055 | CC | cell-substrate junction | 2/27 | 404/18675 | 0.115078 | 0.231464 | 0.161055 | RPL10A/DPP4 | 2 |
| GO:0005581 | CC | collagen trimer | 1/27 | 86/18675 | 0.117249 | 0.23318 | 0.162248 | C1QA | 1 |
| GO:0031091 | CC | platelet alpha granule | 1/27 | 91/18675 | 0.123641 | 0.240489 | 0.167334 | F5 | 1 |
| GO:0032587 | CC | ruffle membrane | 1/27 | 91/18675 | 0.123641 | 0.240489 | 0.167334 | PLEKHO1 | 1 |
| GO:1990204 | CC | oxidoreductase complex | 1/27 | 93/18675 | 0.126186 | 0.242771 | 0.168922 | RRM2 | 1 |
| GO:0012505 | CC | endomembrane system | 9/27 | 4213/18675 | 0.134685 | 0.256336 | 0.178361 | ST6GALNAC3/RAB13/F5/NR3C2/MGAM/SLC2A3/VNN1/HEPACAM2/CEACAM1 | 9 |
| GO:0042995 | CC | cell projection | 5/27 | 1917/18675 | 0.137161 | 0.256647 | 0.178577 | RAB13/DPP4/PLEKHO1/SLC2A3/CEACAM1 | 5 |
| GO:0098797 | CC | plasma membrane protein complex | 2/27 | 451/18675 | 0.137748 | 0.256647 | 0.178577 | TGFBR3/CEACAM1 | 2 |
| GO:0031012 | CC | extracellular matrix | 2/27 | 468/18675 | 0.146187 | 0.269533 | 0.187543 | TGFBR3/C1QA | 2 |
| GO:0005813 | CC | centrosome | 2/27 | 473/18675 | 0.148691 | 0.271322 | 0.188788 | CDC25B/HEPACAM2 | 2 |
| GO:0005794 | CC | Golgi apparatus | 4/27 | 1441/18675 | 0.150935 | 0.272607 | 0.189682 | ST6GALNAC3/RAB13/F5/HEPACAM2 | 4 |
| GO:0005793 | CC | endoplasmic reticulum-Golgi intermediate compartment | 1/27 | 115/18675 | 0.153712 | 0.274819 | 0.191221 | F5 | 1 |
| GO:0005923 | CC | bicellular tight junction | 1/27 | 118/18675 | 0.1574 | 0.278599 | 0.193851 | RAB13 | 1 |
| GO:0070160 | CC | tight junction | 1/27 | 123/18675 | 0.163513 | 0.286552 | 0.199385 | RAB13 | 1 |
| GO:0043204 | CC | perikaryon | 1/27 | 127/18675 | 0.168372 | 0.292176 | 0.203298 | SLC2A3 | 1 |
| GO:0043296 | CC | apical junction complex | 1/27 | 134/18675 | 0.176811 | 0.30384 | 0.211414 | RAB13 | 1 |
| GO:0044430 | CC | cytoskeletal part | 4/27 | 1543/18675 | 0.17974 | 0.305904 | 0.21285 | CDC25B/PRC1/NLRC3/HEPACAM2 | 4 |
| GO:0044437 | CC | vacuolar part | 2/27 | 538/18675 | 0.181995 | 0.306791 | 0.213467 | DPP4/VNN1 | 2 |
| GO:0005798 | CC | Golgi-associated vesicle | 1/27 | 145/18675 | 0.189905 | 0.317106 | 0.220644 | F5 | 1 |
| GO:0098794 | CC | postsynapse | 2/27 | 568/18675 | 0.197754 | 0.323382 | 0.225011 | RPL10A/C1QA | 2 |
| GO:0098802 | CC | plasma membrane receptor complex | 1/27 | 153/18675 | 0.199302 | 0.323382 | 0.225011 | CEACAM1 | 1 |
| GO:0005766 | CC | primary lysosome | 1/27 | 155/18675 | 0.201635 | 0.323382 | 0.225011 | VNN1 | 1 |
| GO:0042582 | CC | azurophil granule | 1/27 | 155/18675 | 0.201635 | 0.323382 | 0.225011 | VNN1 | 1 |
| GO:0031225 | CC | anchored component of membrane | 1/27 | 156/18675 | 0.202799 | 0.323382 | 0.225011 | VNN1 | 1 |
| GO:0055037 | CC | recycling endosome | 1/27 | 164/18675 | 0.212051 | 0.335117 | 0.233177 | RAB13 | 1 |
| GO:0001726 | CC | ruffle | 1/27 | 168/18675 | 0.216639 | 0.339337 | 0.236113 | PLEKHO1 | 1 |
| GO:0005887 | CC | integral component of plasma membrane | 3/27 | 1158/18675 | 0.23302 | 0.361794 | 0.251739 | TGFBR3/SLC2A3/CEACAM1 | 3 |
| GO:0030658 | CC | transport vesicle membrane | 1/27 | 188/18675 | 0.239193 | 0.36815 | 0.256161 | CEACAM1 | 1 |
| GO:0000323 | CC | lytic vacuole | 2/27 | 659/18675 | 0.246495 | 0.372058 | 0.258881 | DPP4/VNN1 | 2 |
| GO:0005764 | CC | lysosome | 2/27 | 659/18675 | 0.246495 | 0.372058 | 0.258881 | DPP4/VNN1 | 2 |
| GO:0005802 | CC | trans-Golgi network | 1/27 | 196/18675 | 0.248039 | 0.372058 | 0.258881 | RAB13 | 1 |
| GO:0009897 | CC | external side of plasma membrane | 1/27 | 199/18675 | 0.25133 | 0.372326 | 0.259067 | TGFBR3 | 1 |
| GO:0098858 | CC | actin-based cell projection | 1/27 | 200/18675 | 0.252424 | 0.372326 | 0.259067 | CEACAM1 | 1 |
| GO:0031226 | CC | intrinsic component of plasma membrane | 3/27 | 1231/18675 | 0.26175 | 0.382891 | 0.266418 | TGFBR3/SLC2A3/CEACAM1 | 3 |
| GO:0120025 | CC | plasma membrane bounded cell projection | 4/27 | 1854/18675 | 0.277542 | 0.402663 | 0.280176 | RAB13/DPP4/PLEKHO1/CEACAM1 | 4 |
| GO:0005773 | CC | vacuole | 2/27 | 737/18675 | 0.288786 | 0.414356 | 0.288312 | DPP4/VNN1 | 2 |
| GO:0044463 | CC | cell projection part | 3/27 | 1308/18675 | 0.292625 | 0.414356 | 0.288312 | DPP4/PLEKHO1/CEACAM1 | 3 |
| GO:0120038 | CC | plasma membrane bounded cell projection part | 3/27 | 1308/18675 | 0.292625 | 0.414356 | 0.288312 | DPP4/PLEKHO1/CEACAM1 | 3 |
| GO:0030135 | CC | coated vesicle | 1/27 | 261/18675 | 0.316329 | 0.444366 | 0.309193 | F5 | 1 |
| GO:0005856 | CC | cytoskeleton | 4/27 | 2041/18675 | 0.340819 | 0.475 | 0.330508 | CDC25B/PRC1/NLRC3/HEPACAM2 | 4 |
| GO:0014069 | CC | postsynaptic density | 1/27 | 290/18675 | 0.344832 | 0.476838 | 0.331787 | RPL10A | 1 |
| GO:0032279 | CC | asymmetric synapse | 1/27 | 294/18675 | 0.348672 | 0.478411 | 0.332882 | RPL10A | 1 |
| GO:0044456 | CC | synapse part | 2/27 | 861/18675 | 0.355601 | 0.47974 | 0.333807 | RPL10A/C1QA | 2 |
| GO:0005788 | CC | endoplasmic reticulum lumen | 1/27 | 306/18675 | 0.360064 | 0.47974 | 0.333807 | F5 | 1 |
| GO:0045121 | CC | membrane raft | 1/27 | 309/18675 | 0.362882 | 0.47974 | 0.333807 | DPP4 | 1 |
| GO:0098857 | CC | membrane microdomain | 1/27 | 310/18675 | 0.363819 | 0.47974 | 0.333807 | DPP4 | 1 |
| GO:0098984 | CC | neuron to neuron synapse | 1/27 | 315/18675 | 0.368482 | 0.47974 | 0.333807 | RPL10A | 1 |
| GO:0099572 | CC | postsynaptic specialization | 1/27 | 316/18675 | 0.369411 | 0.47974 | 0.333807 | RPL10A | 1 |
| GO:0097458 | CC | neuron part | 3/27 | 1500/18675 | 0.370686 | 0.47974 | 0.333807 | RAB13/RPL10A/SLC2A3 | 3 |
| GO:0034774 | CC | secretory granule lumen | 1/27 | 321/18675 | 0.374035 | 0.47974 | 0.333807 | F5 | 1 |
| GO:0098589 | CC | membrane region | 1/27 | 321/18675 | 0.374035 | 0.47974 | 0.333807 | DPP4 | 1 |
| GO:0060205 | CC | cytoplasmic vesicle lumen | 1/27 | 338/18675 | 0.389513 | 0.493592 | 0.343445 | F5 | 1 |
| GO:0031983 | CC | vesicle lumen | 1/27 | 339/18675 | 0.390412 | 0.493592 | 0.343445 | F5 | 1 |
| GO:0016021 | CC | integral component of membrane | 8/27 | 4821/18675 | 0.394967 | 0.495809 | 0.344988 | TGFBR3/ST6GALNAC3/DPP4/MGAM/SLC2A3/VNN1/HEPACAM2/CEACAM1 | 8 |
| GO:0030133 | CC | transport vesicle | 1/27 | 349/18675 | 0.399331 | 0.496193 | 0.345254 | CEACAM1 | 1 |
| GO:0098796 | CC | membrane protein complex | 2/27 | 947/18675 | 0.400879 | 0.496193 | 0.345254 | TGFBR3/CEACAM1 | 2 |
| GO:0005874 | CC | microtubule | 1/27 | 357/18675 | 0.406376 | 0.498193 | 0.346646 | PRC1 | 1 |
| GO:0098552 | CC | side of membrane | 1/27 | 359/18675 | 0.408124 | 0.498193 | 0.346646 | TGFBR3 | 1 |
| GO:0016607 | CC | nuclear speck | 1/27 | 386/18675 | 0.431252 | 0.520087 | 0.36188 | GADD45A | 1 |
| GO:0031224 | CC | intrinsic component of membrane | 8/27 | 4976/18675 | 0.433868 | 0.520087 | 0.36188 | TGFBR3/ST6GALNAC3/DPP4/MGAM/SLC2A3/VNN1/HEPACAM2/CEACAM1 | 8 |
| GO:0032991 | CC | protein-containing complex | 8/27 | 4980/18675 | 0.434875 | 0.520087 | 0.36188 | TGFBR3/RPL10A/RRM2/RPL36/NR3C2/C1QA/GRB10/CEACAM1 | 8 |
| GO:0062023 | CC | collagen-containing extracellular matrix | 1/27 | 399/18675 | 0.442075 | 0.525149 | 0.365402 | C1QA | 1 |
| GO:0045202 | CC | synapse | 2/27 | 1091/18675 | 0.473504 | 0.558735 | 0.388772 | RPL10A/C1QA | 2 |
| GO:0043025 | CC | neuronal cell body | 1/27 | 454/18675 | 0.485708 | 0.56934 | 0.396151 | SLC2A3 | 1 |
| GO:0010008 | CC | endosome membrane | 1/27 | 464/18675 | 0.49328 | 0.574412 | 0.39968 | RAB13 | 1 |
| GO:0044440 | CC | endosomal part | 1/27 | 504/18675 | 0.522509 | 0.604471 | 0.420595 | RAB13 | 1 |
| GO:0044297 | CC | cell body | 1/27 | 522/18675 | 0.535126 | 0.615047 | 0.427954 | SLC2A3 | 1 |
| GO:0044432 | CC | endoplasmic reticulum part | 2/27 | 1289/18675 | 0.564927 | 0.645111 | 0.448873 | F5/NR3C2 | 2 |
| GO:0005654 | CC | nucleoplasm | 5/27 | 3427/18675 | 0.568946 | 0.645535 | 0.449168 | CDC25B/ST6GALNAC3/GADD45A/NR3C2/HPGD | 5 |
| GO:1990904 | CC | ribonucleoprotein complex | 2/27 | 1315/18675 | 0.57612 | 0.649511 | 0.451935 | RPL10A/RPL36 | 2 |
| GO:0099513 | CC | polymeric cytoskeletal fiber | 1/27 | 635/18675 | 0.607299 | 0.680328 | 0.473377 | PRC1 | 1 |
| GO:0048471 | CC | perinuclear region of cytoplasm | 1/27 | 705/18675 | 0.646458 | 0.7128 | 0.495971 | NLRC3 | 1 |
| GO:0031981 | CC | nuclear lumen | 6/27 | 4454/18675 | 0.651642 | 0.7128 | 0.495971 | CDC25B/ST6GALNAC3/GADD45A/RPL36/NR3C2/HPGD | 6 |
| GO:0043232 | CC | intracellular non-membrane-bounded organelle | 6/27 | 4464/18675 | 0.654042 | 0.7128 | 0.495971 | CDC25B/PRC1/RPL10A/RPL36/NLRC3/HEPACAM2 | 6 |
| GO:0043228 | CC | non-membrane-bounded organelle | 6/27 | 4473/18675 | 0.656194 | 0.7128 | 0.495971 | CDC25B/PRC1/RPL10A/RPL36/NLRC3/HEPACAM2 | 6 |
| GO:0036477 | CC | somatodendritic compartment | 1/27 | 724/18675 | 0.65642 | 0.7128 | 0.495971 | SLC2A3 | 1 |
| GO:0016604 | CC | nuclear body | 1/27 | 764/18675 | 0.676518 | 0.730144 | 0.50804 | GADD45A | 1 |
| GO:0099512 | CC | supramolecular fiber | 1/27 | 845/18675 | 0.713805 | 0.755226 | 0.525492 | PRC1 | 1 |
| GO:0099081 | CC | supramolecular polymer | 1/27 | 851/18675 | 0.716396 | 0.755226 | 0.525492 | PRC1 | 1 |
| GO:0005768 | CC | endosome | 1/27 | 852/18675 | 0.716825 | 0.755226 | 0.525492 | RAB13 | 1 |
| GO:0099080 | CC | supramolecular complex | 1/27 | 852/18675 | 0.716825 | 0.755226 | 0.525492 | PRC1 | 1 |
| GO:0044428 | CC | nuclear part | 6/27 | 4801/18675 | 0.729601 | 0.764139 | 0.531693 | CDC25B/ST6GALNAC3/GADD45A/RPL36/NR3C2/HPGD | 6 |
| GO:0005783 | CC | endoplasmic reticulum | 2/27 | 1785/18675 | 0.744467 | 0.775121 | 0.539335 | F5/NR3C2 | 2 |
| GO:0005789 | CC | endoplasmic reticulum membrane | 1/27 | 1005/18675 | 0.775671 | 0.798616 | 0.555683 | NR3C2 | 1 |
| GO:0098827 | CC | endoplasmic reticulum subcompartment | 1/27 | 1009/18675 | 0.777039 | 0.798616 | 0.555683 | NR3C2 | 1 |
| GO:0042175 | CC | nuclear outer membrane-endoplasmic reticulum membrane network | 1/27 | 1026/18675 | 0.782764 | 0.798616 | 0.555683 | NR3C2 | 1 |
| GO:0044451 | CC | nucleoplasm part | 1/27 | 1033/18675 | 0.78508 | 0.798616 | 0.555683 | GADD45A | 1 |
| GO:0043005 | CC | neuron projection | 1/27 | 1091/18675 | 0.803377 | 0.811556 | 0.564687 | RAB13 | 1 |
| GO:1902494 | CC | catalytic complex | 1/27 | 1103/18675 | 0.806971 | 0.811556 | 0.564687 | RRM2 | 1 |
| GO:0005730 | CC | nucleolus | 1/27 | 1369/18675 | 0.872171 | 0.872171 | 0.606863 | RPL36 | 1 |
| GO:0042803 | MF | protein homodimerization activity | 6/27 | 809/16967 | 0.001443 | 0.129821 | 0.096115 | GADD45A/RRM2/DPP4/HPGD/CCL5/CEACAM1 | 6 |
| GO:0004558 | MF | alpha-1,4-glucosidase activity | 1/27 | 3/16967 | 0.004767 | 0.129821 | 0.096115 | MGAM | 1 |
| GO:0004957 | MF | prostaglandin E receptor activity | 1/27 | 3/16967 | 0.004767 | 0.129821 | 0.096115 | HPGD | 1 |
| GO:0017159 | MF | pantetheine hydrolase activity | 1/27 | 3/16967 | 0.004767 | 0.129821 | 0.096115 | VNN1 | 1 |
| GO:0030298 | MF | receptor signaling protein tyrosine kinase activator activity | 1/27 | 3/16967 | 0.004767 | 0.129821 | 0.096115 | CCL5 | 1 |
| GO:0032450 | MF | maltose alpha-glucosidase activity | 1/27 | 3/16967 | 0.004767 | 0.129821 | 0.096115 | MGAM | 1 |
| GO:0047844 | MF | deoxycytidine deaminase activity | 1/27 | 3/16967 | 0.004767 | 0.129821 | 0.096115 | APOBEC3B | 1 |
| GO:0031726 | MF | CCR1 chemokine receptor binding | 1/27 | 5/16967 | 0.007932 | 0.129821 | 0.096115 | CCL5 | 1 |
| GO:0005024 | MF | transforming growth factor beta-activated receptor activity | 1/27 | 6/16967 | 0.009511 | 0.129821 | 0.096115 | TGFBR3 | 1 |
| GO:0008199 | MF | ferric iron binding | 1/27 | 6/16967 | 0.009511 | 0.129821 | 0.096115 | RRM2 | 1 |
| GO:0016160 | MF | amylase activity | 1/27 | 6/16967 | 0.009511 | 0.129821 | 0.096115 | MGAM | 1 |
| GO:0090599 | MF | alpha-glucosidase activity | 1/27 | 6/16967 | 0.009511 | 0.129821 | 0.096115 | MGAM | 1 |
| GO:0016810 | MF | hydrolase activity, acting on carbon-nitrogen (but not peptide) bonds | 2/27 | 97/16967 | 0.010346 | 0.129821 | 0.096115 | APOBEC3B/VNN1 | 2 |
| GO:0004955 | MF | prostaglandin receptor activity | 1/27 | 7/16967 | 0.011088 | 0.129821 | 0.096115 | HPGD | 1 |
| GO:0005114 | MF | type II transforming growth factor beta receptor binding | 1/27 | 7/16967 | 0.011088 | 0.129821 | 0.096115 | TGFBR3 | 1 |
| GO:0031730 | MF | CCR5 chemokine receptor binding | 1/27 | 7/16967 | 0.011088 | 0.129821 | 0.096115 | CCL5 | 1 |
| GO:0004954 | MF | prostanoid receptor activity | 1/27 | 8/16967 | 0.012663 | 0.129821 | 0.096115 | HPGD | 1 |
| GO:0008239 | MF | dipeptidyl-peptidase activity | 1/27 | 8/16967 | 0.012663 | 0.129821 | 0.096115 | DPP4 | 1 |
| GO:0016725 | MF | oxidoreductase activity, acting on CH or CH2 groups | 1/27 | 8/16967 | 0.012663 | 0.129821 | 0.096115 | RRM2 | 1 |
| GO:0046983 | MF | protein dimerization activity | 6/27 | 1285/16967 | 0.013884 | 0.129821 | 0.096115 | GADD45A/RRM2/DPP4/HPGD/CCL5/CEACAM1 | 6 |
| GO:0036312 | MF | phosphatidylinositol 3-kinase regulatory subunit binding | 1/27 | 10/16967 | 0.015804 | 0.129821 | 0.096115 | NLRC3 | 1 |
| GO:0005536 | MF | glucose binding | 1/27 | 11/16967 | 0.017371 | 0.129821 | 0.096115 | SLC2A3 | 1 |
| GO:0015926 | MF | glucosidase activity | 1/27 | 11/16967 | 0.017371 | 0.129821 | 0.096115 | MGAM | 1 |
| GO:0016004 | MF | phospholipase activator activity | 1/27 | 11/16967 | 0.017371 | 0.129821 | 0.096115 | CCL5 | 1 |
| GO:0031005 | MF | filamin binding | 1/27 | 11/16967 | 0.017371 | 0.129821 | 0.096115 | CEACAM1 | 1 |
| GO:0048185 | MF | activin binding | 1/27 | 11/16967 | 0.017371 | 0.129821 | 0.096115 | TGFBR3 | 1 |
| GO:0019900 | MF | kinase binding | 4/27 | 647/16967 | 0.018303 | 0.129821 | 0.096115 | CDC25B/GADD45A/PRC1/CEACAM1 | 4 |
| GO:0004953 | MF | icosanoid receptor activity | 1/27 | 12/16967 | 0.018936 | 0.129821 | 0.096115 | HPGD | 1 |
| GO:0015125 | MF | bile acid transmembrane transporter activity | 1/27 | 12/16967 | 0.018936 | 0.129821 | 0.096115 | CEACAM1 | 1 |
| GO:0070403 | MF | NAD+ binding | 1/27 | 12/16967 | 0.018936 | 0.129821 | 0.096115 | HPGD | 1 |
| GO:0005355 | MF | glucose transmembrane transporter activity | 1/27 | 13/16967 | 0.020498 | 0.129821 | 0.096115 | SLC2A3 | 1 |
| GO:0015149 | MF | hexose transmembrane transporter activity | 1/27 | 13/16967 | 0.020498 | 0.129821 | 0.096115 | SLC2A3 | 1 |
| GO:0060229 | MF | lipase activator activity | 1/27 | 13/16967 | 0.020498 | 0.129821 | 0.096115 | CCL5 | 1 |
| GO:0008201 | MF | heparin binding | 2/27 | 148/16967 | 0.023001 | 0.137101 | 0.101505 | TGFBR3/PCOLCE2 | 2 |
| GO:0015145 | MF | monosaccharide transmembrane transporter activity | 1/27 | 15/16967 | 0.023615 | 0.137101 | 0.101505 | SLC2A3 | 1 |
| GO:0048019 | MF | receptor antagonist activity | 1/27 | 15/16967 | 0.023615 | 0.137101 | 0.101505 | CCL5 | 1 |
| GO:0004675 | MF | transmembrane receptor protein serine/threonine kinase activity | 1/27 | 16/16967 | 0.025171 | 0.137566 | 0.101849 | TGFBR3 | 1 |
| GO:0003735 | MF | structural constituent of ribosome | 2/27 | 157/16967 | 0.025668 | 0.137566 | 0.101849 | RPL10A/RPL36 | 2 |
| GO:0051119 | MF | sugar transmembrane transporter activity | 1/27 | 17/16967 | 0.026723 | 0.137566 | 0.101849 | SLC2A3 | 1 |
| GO:0004435 | MF | phosphatidylinositol phospholipase C activity | 1/27 | 19/16967 | 0.029822 | 0.137566 | 0.101849 | CCL5 | 1 |
| GO:0019239 | MF | deaminase activity | 1/27 | 19/16967 | 0.029822 | 0.137566 | 0.101849 | APOBEC3B | 1 |
| GO:0030296 | MF | protein tyrosine kinase activator activity | 1/27 | 19/16967 | 0.029822 | 0.137566 | 0.101849 | CCL5 | 1 |
| GO:0005158 | MF | insulin receptor binding | 1/27 | 20/16967 | 0.031367 | 0.137566 | 0.101849 | GRB10 | 1 |
| GO:0005160 | MF | transforming growth factor beta receptor binding | 1/27 | 20/16967 | 0.031367 | 0.137566 | 0.101849 | TGFBR3 | 1 |
| GO:0008373 | MF | sialyltransferase activity | 1/27 | 20/16967 | 0.031367 | 0.137566 | 0.101849 | ST6GALNAC3 | 1 |
| GO:0000979 | MF | RNA polymerase II core promoter sequence-specific DNA binding | 1/27 | 21/16967 | 0.03291 | 0.137566 | 0.101849 | GADD45A | 1 |
| GO:0016814 | MF | hydrolase activity, acting on carbon-nitrogen (but not peptide) bonds, in cyclic amidines | 1/27 | 21/16967 | 0.03291 | 0.137566 | 0.101849 | APOBEC3B | 1 |
| GO:0017134 | MF | fibroblast growth factor binding | 1/27 | 21/16967 | 0.03291 | 0.137566 | 0.101849 | TGFBR3 | 1 |
| GO:0030547 | MF | receptor inhibitor activity | 1/27 | 21/16967 | 0.03291 | 0.137566 | 0.101849 | CCL5 | 1 |
| GO:0050431 | MF | transforming growth factor beta binding | 1/27 | 21/16967 | 0.03291 | 0.137566 | 0.101849 | TGFBR3 | 1 |
| GO:0004629 | MF | phospholipase C activity | 1/27 | 24/16967 | 0.037526 | 0.153783 | 0.113856 | CCL5 | 1 |
| GO:0005539 | MF | glycosaminoglycan binding | 2/27 | 199/16967 | 0.039648 | 0.157103 | 0.116314 | TGFBR3/PCOLCE2 | 2 |
| GO:0008028 | MF | monocarboxylic acid transmembrane transporter activity | 1/27 | 26/16967 | 0.040591 | 0.157103 | 0.116314 | CEACAM1 | 1 |
| GO:0048020 | MF | CCR chemokine receptor binding | 1/27 | 26/16967 | 0.040591 | 0.157103 | 0.116314 | CCL5 | 1 |
| GO:0015144 | MF | carbohydrate transmembrane transporter activity | 1/27 | 28/16967 | 0.043647 | 0.158295 | 0.117196 | SLC2A3 | 1 |
| GO:0043548 | MF | phosphatidylinositol 3-kinase binding | 1/27 | 28/16967 | 0.043647 | 0.158295 | 0.117196 | NLRC3 | 1 |
| GO:0005126 | MF | cytokine receptor binding | 2/27 | 210/16967 | 0.043697 | 0.158295 | 0.117196 | TGFBR3/CCL5 | 2 |
| GO:0042802 | MF | identical protein binding | 6/27 | 1670/16967 | 0.043929 | 0.158295 | 0.117196 | GADD45A/RRM2/DPP4/HPGD/CCL5/CEACAM1 | 6 |
| GO:0042056 | MF | chemoattractant activity | 1/27 | 29/16967 | 0.045171 | 0.160014 | 0.118469 | CCL5 | 1 |
| GO:0008009 | MF | chemokine activity | 1/27 | 30/16967 | 0.046693 | 0.162649 | 0.12042 | CCL5 | 1 |
| GO:1901681 | MF | sulfur compound binding | 2/27 | 221/16967 | 0.047895 | 0.164099 | 0.121494 | TGFBR3/PCOLCE2 | 2 |
| GO:0001046 | MF | core promoter sequence-specific DNA binding | 1/27 | 32/16967 | 0.04973 | 0.16764 | 0.124115 | GADD45A | 1 |
| GO:0016504 | MF | peptidase activator activity | 1/27 | 36/16967 | 0.055777 | 0.182146 | 0.134855 | PCOLCE2 | 1 |
| GO:1901618 | MF | organic hydroxy compound transmembrane transporter activity | 1/27 | 36/16967 | 0.055777 | 0.182146 | 0.134855 | CEACAM1 | 1 |
| GO:0019901 | MF | protein kinase binding | 3/27 | 568/16967 | 0.060227 | 0.189901 | 0.140596 | CDC25B/PRC1/CEACAM1 | 3 |
| GO:0030246 | MF | carbohydrate binding | 2/27 | 252/16967 | 0.060475 | 0.189901 | 0.140596 | MGAM/SLC2A3 | 2 |
| GO:0015026 | MF | coreceptor activity | 1/27 | 40/16967 | 0.061786 | 0.189901 | 0.140596 | TGFBR3 | 1 |
| GO:0019894 | MF | kinesin binding | 1/27 | 40/16967 | 0.061786 | 0.189901 | 0.140596 | PRC1 | 1 |
| GO:0004177 | MF | aminopeptidase activity | 1/27 | 41/16967 | 0.063282 | 0.191681 | 0.141915 | DPP4 | 1 |
| GO:0001047 | MF | core promoter binding | 1/27 | 42/16967 | 0.064777 | 0.193404 | 0.14319 | GADD45A | 1 |
| GO:0042379 | MF | chemokine receptor binding | 1/27 | 47/16967 | 0.072213 | 0.212572 | 0.157381 | CCL5 | 1 |
| GO:0051287 | MF | NAD binding | 1/27 | 50/16967 | 0.076648 | 0.222492 | 0.164726 | HPGD | 1 |
| GO:0005070 | MF | SH3/SH2 adaptor activity | 1/27 | 52/16967 | 0.079593 | 0.227252 | 0.16825 | GRB10 | 1 |
| GO:0005507 | MF | copper ion binding | 1/27 | 53/16967 | 0.081062 | 0.227252 | 0.16825 | F5 | 1 |
| GO:0003707 | MF | steroid hormone receptor activity | 1/27 | 54/16967 | 0.082529 | 0.227252 | 0.16825 | NR3C2 | 1 |
| GO:0046914 | MF | transition metal ion binding | 4/27 | 1051/16967 | 0.082637 | 0.227252 | 0.16825 | F5/RRM2/NR3C2/APOBEC3B | 4 |
| GO:0042578 | MF | phosphoric ester hydrolase activity | 2/27 | 305/16967 | 0.084253 | 0.228686 | 0.169312 | CDC25B/CCL5 | 2 |
| GO:0043621 | MF | protein self-association | 1/27 | 56/16967 | 0.085456 | 0.228979 | 0.169529 | CCL5 | 1 |
| GO:0016811 | MF | hydrolase activity, acting on carbon-nitrogen (but not peptide) bonds, in linear amides | 1/27 | 58/16967 | 0.088374 | 0.233801 | 0.173099 | VNN1 | 1 |
| GO:0005518 | MF | collagen binding | 1/27 | 61/16967 | 0.092735 | 0.24227 | 0.179369 | PCOLCE2 | 1 |
| GO:0048029 | MF | monosaccharide binding | 1/27 | 62/16967 | 0.094184 | 0.243017 | 0.179922 | SLC2A3 | 1 |
| GO:0019199 | MF | transmembrane receptor protein kinase activity | 1/27 | 65/16967 | 0.098517 | 0.250009 | 0.185098 | TGFBR3 | 1 |
| GO:0016787 | MF | hydrolase activity | 6/27 | 2052/16967 | 0.099286 | 0.250009 | 0.185098 | CDC25B/DPP4/MGAM/APOBEC3B/VNN1/CCL5 | 6 |
| GO:0030295 | MF | protein kinase activator activity | 1/27 | 68/16967 | 0.102831 | 0.255853 | 0.189425 | CCL5 | 1 |
| GO:0001540 | MF | amyloid-beta binding | 1/27 | 71/16967 | 0.107124 | 0.261598 | 0.193679 | C1QA | 1 |
| GO:0046332 | MF | SMAD binding | 1/27 | 72/16967 | 0.108551 | 0.261598 | 0.193679 | TGFBR3 | 1 |
| GO:0001618 | MF | virus receptor activity | 1/27 | 74/16967 | 0.111398 | 0.261598 | 0.193679 | DPP4 | 1 |
| GO:0019209 | MF | kinase activator activity | 1/27 | 74/16967 | 0.111398 | 0.261598 | 0.193679 | CCL5 | 1 |
| GO:0104005 | MF | hijacked molecular function | 1/27 | 74/16967 | 0.111398 | 0.261598 | 0.193679 | DPP4 | 1 |
| GO:0008081 | MF | phosphoric diester hydrolase activity | 1/27 | 78/16967 | 0.117066 | 0.268866 | 0.19906 | CCL5 | 1 |
| GO:0035591 | MF | signaling adaptor activity | 1/27 | 78/16967 | 0.117066 | 0.268866 | 0.19906 | GRB10 | 1 |
| GO:0030165 | MF | PDZ domain binding | 1/27 | 79/16967 | 0.118478 | 0.269151 | 0.19927 | TGFBR3 | 1 |
| GO:1990782 | MF | protein tyrosine kinase binding | 1/27 | 80/16967 | 0.119887 | 0.269424 | 0.199473 | CEACAM1 | 1 |
| GO:0004553 | MF | hydrolase activity, hydrolyzing O-glycosyl compounds | 1/27 | 82/16967 | 0.122699 | 0.270181 | 0.200033 | MGAM | 1 |
| GO:0004620 | MF | phospholipase activity | 1/27 | 83/16967 | 0.124102 | 0.270181 | 0.200033 | CCL5 | 1 |
| GO:0005496 | MF | steroid binding | 1/27 | 83/16967 | 0.124102 | 0.270181 | 0.200033 | NR3C2 | 1 |
| GO:0004725 | MF | protein tyrosine phosphatase activity | 1/27 | 92/16967 | 0.136631 | 0.29439 | 0.217957 | CDC25B | 1 |
| GO:0008047 | MF | enzyme activator activity | 2/27 | 419/16967 | 0.142778 | 0.299545 | 0.221773 | PCOLCE2/CCL5 | 2 |
| GO:0008238 | MF | exopeptidase activity | 1/27 | 97/16967 | 0.143517 | 0.299545 | 0.221773 | DPP4 | 1 |
| GO:0046943 | MF | carboxylic acid transmembrane transporter activity | 1/27 | 98/16967 | 0.144887 | 0.299545 | 0.221773 | CEACAM1 | 1 |
| GO:0005342 | MF | organic acid transmembrane transporter activity | 1/27 | 99/16967 | 0.146256 | 0.299545 | 0.221773 | CEACAM1 | 1 |
| GO:0016298 | MF | lipase activity | 1/27 | 100/16967 | 0.147623 | 0.299545 | 0.221773 | CCL5 | 1 |
| GO:0019955 | MF | cytokine binding | 1/27 | 100/16967 | 0.147623 | 0.299545 | 0.221773 | TGFBR3 | 1 |
| GO:0005319 | MF | lipid transporter activity | 1/27 | 103/16967 | 0.15171 | 0.304678 | 0.225574 | CEACAM1 | 1 |
| GO:0016798 | MF | hydrolase activity, acting on glycosyl bonds | 1/27 | 104/16967 | 0.153068 | 0.304678 | 0.225574 | MGAM | 1 |
| GO:0016616 | MF | oxidoreductase activity, acting on the CH-OH group of donors, NAD or NADP as acceptor | 1/27 | 108/16967 | 0.15848 | 0.312186 | 0.231132 | HPGD | 1 |
| GO:0047485 | MF | protein N-terminus binding | 1/27 | 109/16967 | 0.159827 | 0.312186 | 0.231132 | GADD45A | 1 |
| GO:0043565 | MF | sequence-specific DNA binding | 3/27 | 908/16967 | 0.173448 | 0.328127 | 0.242935 | GADD45A/NR3C2/DACH1 | 3 |
| GO:0016614 | MF | oxidoreductase activity, acting on CH-OH group of donors | 1/27 | 120/16967 | 0.174516 | 0.328127 | 0.242935 | HPGD | 1 |
| GO:0005102 | MF | signaling receptor binding | 4/27 | 1386/16967 | 0.174654 | 0.328127 | 0.242935 | TGFBR3/DPP4/CCL5/GRB10 | 4 |
| GO:0002020 | MF | protease binding | 1/27 | 121/16967 | 0.175839 | 0.328127 | 0.242935 | DPP4 | 1 |
| GO:0019838 | MF | growth factor binding | 1/27 | 121/16967 | 0.175839 | 0.328127 | 0.242935 | TGFBR3 | 1 |
| GO:0044877 | MF | protein-containing complex binding | 3/27 | 938/16967 | 0.185448 | 0.342996 | 0.253943 | TGFBR3/PCOLCE2/GRB10 | 3 |
| GO:0019899 | MF | enzyme binding | 5/27 | 1941/16967 | 0.189637 | 0.345131 | 0.255524 | CDC25B/GADD45A/PRC1/DPP4/CEACAM1 | 5 |
| GO:0019903 | MF | protein phosphatase binding | 1/27 | 132/16967 | 0.190257 | 0.345131 | 0.255524 | CEACAM1 | 1 |
| GO:0004252 | MF | serine-type endopeptidase activity | 1/27 | 133/16967 | 0.191556 | 0.345131 | 0.255524 | DPP4 | 1 |
| GO:0019887 | MF | protein kinase regulator activity | 1/27 | 136/16967 | 0.19544 | 0.349119 | 0.258476 | CCL5 | 1 |
| GO:0004672 | MF | protein kinase activity | 2/27 | 517/16967 | 0.1983 | 0.350041 | 0.259159 | TGFBR3/CCL5 | 2 |
| GO:0008514 | MF | organic anion transmembrane transporter activity | 1/27 | 139/16967 | 0.199306 | 0.350041 | 0.259159 | CEACAM1 | 1 |
| GO:0005506 | MF | iron ion binding | 1/27 | 144/16967 | 0.205709 | 0.358277 | 0.265257 | RRM2 | 1 |
| GO:0003824 | MF | catalytic activity | 10/27 | 4828/16967 | 0.215472 | 0.370524 | 0.274324 | CDC25B/TGFBR3/ST6GALNAC3/RRM2/DPP4/MGAM/HPGD/APOBEC3B/VNN1/CCL5 | 10 |
| GO:0005125 | MF | cytokine activity | 1/27 | 154/16967 | 0.218369 | 0.370524 | 0.274324 | CCL5 | 1 |
| GO:0019207 | MF | kinase regulator activity | 1/27 | 154/16967 | 0.218369 | 0.370524 | 0.274324 | CCL5 | 1 |
| GO:0030674 | MF | protein binding, bridging | 1/27 | 156/16967 | 0.220878 | 0.370524 | 0.274324 | GRB10 | 1 |
| GO:0004721 | MF | phosphoprotein phosphatase activity | 1/27 | 157/16967 | 0.222129 | 0.370524 | 0.274324 | CDC25B | 1 |
| GO:0008236 | MF | serine-type peptidase activity | 1/27 | 158/16967 | 0.223378 | 0.370524 | 0.274324 | DPP4 | 1 |
| GO:0017171 | MF | serine hydrolase activity | 1/27 | 163/16967 | 0.229596 | 0.37784 | 0.27974 | DPP4 | 1 |
| GO:0008017 | MF | microtubule binding | 1/27 | 166/16967 | 0.233304 | 0.380942 | 0.282037 | PRC1 | 1 |
| GO:0005516 | MF | calmodulin binding | 1/27 | 168/16967 | 0.235767 | 0.381978 | 0.282804 | CEACAM1 | 1 |
| GO:0000977 | MF | RNA polymerase II regulatory region sequence-specific DNA binding | 2/27 | 594/16967 | 0.243679 | 0.390669 | 0.289239 | GADD45A/DACH1 | 2 |
| GO:0001012 | MF | RNA polymerase II regulatory region DNA binding | 2/27 | 596/16967 | 0.244869 | 0.390669 | 0.289239 | GADD45A/DACH1 | 2 |
| GO:0019902 | MF | phosphatase binding | 1/27 | 177/16967 | 0.246753 | 0.390692 | 0.289256 | CEACAM1 | 1 |
| GO:0016773 | MF | phosphotransferase activity, alcohol group as acceptor | 2/27 | 611/16967 | 0.253802 | 0.398831 | 0.295282 | TGFBR3/CCL5 | 2 |
| GO:0016788 | MF | hydrolase activity, acting on ester bonds | 2/27 | 621/16967 | 0.259766 | 0.405157 | 0.299965 | CDC25B/CCL5 | 2 |
| GO:0061134 | MF | peptidase regulator activity | 1/27 | 192/16967 | 0.264727 | 0.409836 | 0.30343 | PCOLCE2 | 1 |
| GO:0000976 | MF | transcription regulatory region sequence-specific DNA binding | 2/27 | 644/16967 | 0.273498 | 0.420302 | 0.311178 | GADD45A/DACH1 | 2 |
| GO:0005198 | MF | structural molecule activity | 2/27 | 653/16967 | 0.278874 | 0.424012 | 0.313924 | RPL10A/RPL36 | 2 |
| GO:0001664 | MF | G protein-coupled receptor binding | 1/27 | 205/16967 | 0.279969 | 0.424012 | 0.313924 | CCL5 | 1 |
| GO:0016491 | MF | oxidoreductase activity | 2/27 | 662/16967 | 0.284251 | 0.426129 | 0.315492 | RRM2/HPGD | 2 |
| GO:0016301 | MF | kinase activity | 2/27 | 664/16967 | 0.285445 | 0.426129 | 0.315492 | TGFBR3/CCL5 | 2 |
| GO:1990837 | MF | sequence-specific double-stranded DNA binding | 2/27 | 674/16967 | 0.291417 | 0.427688 | 0.316647 | GADD45A/DACH1 | 2 |
| GO:0042277 | MF | peptide binding | 1/27 | 215/16967 | 0.291487 | 0.427688 | 0.316647 | C1QA | 1 |
| GO:0060090 | MF | molecular adaptor activity | 1/27 | 216/16967 | 0.292629 | 0.427688 | 0.316647 | GRB10 | 1 |
| GO:0001227 | MF | DNA-binding transcription repressor activity, RNA polymerase II-specific | 1/27 | 224/16967 | 0.301701 | 0.434865 | 0.32196 | DACH1 | 1 |
| GO:0016791 | MF | phosphatase activity | 1/27 | 224/16967 | 0.301701 | 0.434865 | 0.32196 | CDC25B | 1 |
| GO:0008509 | MF | anion transmembrane transporter activity | 1/27 | 240/16967 | 0.31951 | 0.45738 | 0.33863 | CEACAM1 | 1 |
| GO:0044212 | MF | transcription regulatory region DNA binding | 2/27 | 739/16967 | 0.330102 | 0.460318 | 0.340805 | GADD45A/DACH1 | 2 |
| GO:0001067 | MF | regulatory region nucleic acid binding | 2/27 | 740/16967 | 0.330695 | 0.460318 | 0.340805 | GADD45A/DACH1 | 2 |
| GO:0015631 | MF | tubulin binding | 1/27 | 252/16967 | 0.332579 | 0.460318 | 0.340805 | PRC1 | 1 |
| GO:0003690 | MF | double-stranded DNA binding | 2/27 | 744/16967 | 0.333063 | 0.460318 | 0.340805 | GADD45A/DACH1 | 2 |
| GO:0140096 | MF | catalytic activity, acting on a protein | 4/27 | 1837/16967 | 0.334282 | 0.460318 | 0.340805 | CDC25B/TGFBR3/DPP4/CCL5 | 4 |
| GO:0016757 | MF | transferase activity, transferring glycosyl groups | 1/27 | 257/16967 | 0.337952 | 0.460318 | 0.340805 | ST6GALNAC3 | 1 |
| GO:0050662 | MF | coenzyme binding | 1/27 | 257/16967 | 0.337952 | 0.460318 | 0.340805 | HPGD | 1 |
| GO:0003723 | MF | RNA binding | 4/27 | 1850/16967 | 0.339182 | 0.460318 | 0.340805 | RPL10A/RPL36/IFIT1/APOBEC3B | 4 |
| GO:0033218 | MF | amide binding | 1/27 | 266/16967 | 0.34752 | 0.468592 | 0.34693 | C1QA | 1 |
| GO:0016772 | MF | transferase activity, transferring phosphorus-containing groups | 2/27 | 784/16967 | 0.35663 | 0.477793 | 0.353742 | TGFBR3/CCL5 | 2 |
| GO:0038023 | MF | signaling receptor activity | 3/27 | 1344/16967 | 0.362263 | 0.480314 | 0.355609 | TGFBR3/NR3C2/HPGD | 3 |
| GO:0008092 | MF | cytoskeletal protein binding | 2/27 | 797/16967 | 0.364238 | 0.480314 | 0.355609 | PRC1/CEACAM1 | 2 |
| GO:0008270 | MF | zinc ion binding | 2/27 | 799/16967 | 0.365406 | 0.480314 | 0.355609 | NR3C2/APOBEC3B | 2 |
| GO:0060089 | MF | molecular transducer activity | 3/27 | 1381/16967 | 0.378824 | 0.494838 | 0.366362 | TGFBR3/NR3C2/HPGD | 3 |
| GO:0022857 | MF | transmembrane transporter activity | 2/27 | 838/16967 | 0.388035 | 0.501029 | 0.370946 | SLC2A3/CEACAM1 | 2 |
| GO:0030234 | MF | enzyme regulator activity | 2/27 | 840/16967 | 0.389188 | 0.501029 | 0.370946 | PCOLCE2/CCL5 | 2 |
| GO:0043168 | MF | anion binding | 5/27 | 2573/16967 | 0.390755 | 0.501029 | 0.370946 | TGFBR3/PCOLCE2/RAB13/HPGD/NLRC3 | 5 |
| GO:0005525 | MF | GTP binding | 1/27 | 313/16967 | 0.395362 | 0.502756 | 0.372224 | RAB13 | 1 |
| GO:0032550 | MF | purine ribonucleoside binding | 1/27 | 318/16967 | 0.400248 | 0.502756 | 0.372224 | RAB13 | 1 |
| GO:0001883 | MF | purine nucleoside binding | 1/27 | 321/16967 | 0.403161 | 0.502756 | 0.372224 | RAB13 | 1 |
| GO:0004175 | MF | endopeptidase activity | 1/27 | 322/16967 | 0.404129 | 0.502756 | 0.372224 | DPP4 | 1 |
| GO:0032549 | MF | ribonucleoside binding | 1/27 | 322/16967 | 0.404129 | 0.502756 | 0.372224 | RAB13 | 1 |
| GO:0001882 | MF | nucleoside binding | 1/27 | 329/16967 | 0.410864 | 0.504502 | 0.373517 | RAB13 | 1 |
| GO:0019001 | MF | guanyl nucleotide binding | 1/27 | 331/16967 | 0.412774 | 0.504502 | 0.373517 | RAB13 | 1 |
| GO:0032561 | MF | guanyl ribonucleotide binding | 1/27 | 331/16967 | 0.412774 | 0.504502 | 0.373517 | RAB13 | 1 |
| GO:0005215 | MF | transporter activity | 2/27 | 908/16967 | 0.427837 | 0.519469 | 0.384598 | SLC2A3/CEACAM1 | 2 |
| GO:0097367 | MF | carbohydrate derivative binding | 4/27 | 2090/16967 | 0.429991 | 0.519469 | 0.384598 | TGFBR3/PCOLCE2/RAB13/NLRC3 | 4 |
| GO:0003779 | MF | actin binding | 1/27 | 361/16967 | 0.440727 | 0.529379 | 0.391935 | CEACAM1 | 1 |
| GO:0003676 | MF | nucleic acid binding | 7/27 | 3955/16967 | 0.446516 | 0.533267 | 0.394814 | GADD45A/RPL10A/RPL36/NR3C2/IFIT1/APOBEC3B/DACH1 | 7 |
| GO:0004674 | MF | protein serine/threonine kinase activity | 1/27 | 371/16967 | 0.449757 | 0.534086 | 0.39542 | TGFBR3 | 1 |
| GO:0048018 | MF | receptor ligand activity | 1/27 | 397/16967 | 0.472581 | 0.55802 | 0.41314 | CCL5 | 1 |
| GO:0030545 | MF | receptor regulator activity | 1/27 | 425/16967 | 0.496141 | 0.582548 | 0.4313 | CCL5 | 1 |
| GO:0000978 | MF | RNA polymerase II proximal promoter sequence-specific DNA binding | 1/27 | 432/16967 | 0.501871 | 0.585984 | 0.433843 | DACH1 | 1 |
| GO:0048037 | MF | cofactor binding | 1/27 | 438/16967 | 0.506732 | 0.588372 | 0.435612 | HPGD | 1 |
| GO:0000987 | MF | proximal promoter sequence-specific DNA binding | 1/27 | 452/16967 | 0.517898 | 0.598015 | 0.442751 | DACH1 | 1 |
| GO:0036094 | MF | small molecule binding | 4/27 | 2340/16967 | 0.522075 | 0.599526 | 0.443869 | RAB13/HPGD/SLC2A3/NLRC3 | 4 |
| GO:0004888 | MF | transmembrane signaling receptor activity | 2/27 | 1133/16967 | 0.546478 | 0.624119 | 0.462078 | TGFBR3/HPGD | 2 |
| GO:0046982 | MF | protein heterodimerization activity | 1/27 | 497/16967 | 0.552164 | 0.627186 | 0.464348 | GADD45A | 1 |
| GO:0070011 | MF | peptidase activity, acting on L-amino acid peptides | 1/27 | 511/16967 | 0.562337 | 0.635289 | 0.470347 | DPP4 | 1 |
| GO:0008233 | MF | peptidase activity | 1/27 | 536/16967 | 0.579953 | 0.651668 | 0.482474 | DPP4 | 1 |
| GO:0016740 | MF | transferase activity | 3/27 | 1947/16967 | 0.613194 | 0.685334 | 0.507399 | TGFBR3/ST6GALNAC3/CCL5 | 3 |
| GO:0015318 | MF | inorganic molecular entity transmembrane transporter activity | 1/27 | 619/16967 | 0.633674 | 0.700545 | 0.518661 | CEACAM1 | 1 |
| GO:0000166 | MF | nucleotide binding | 3/27 | 2012/16967 | 0.636508 | 0.700545 | 0.518661 | RAB13/HPGD/NLRC3 | 3 |
| GO:1901265 | MF | nucleoside phosphate binding | 3/27 | 2013/16967 | 0.636859 | 0.700545 | 0.518661 | RAB13/HPGD/NLRC3 | 3 |
| GO:0008289 | MF | lipid binding | 1/27 | 643/16967 | 0.647932 | 0.708993 | 0.524916 | NR3C2 | 1 |
| GO:0015075 | MF | ion transmembrane transporter activity | 1/27 | 672/16967 | 0.664448 | 0.723279 | 0.535492 | CEACAM1 | 1 |
| GO:0019904 | MF | protein domain specific binding | 1/27 | 680/16967 | 0.668871 | 0.724321 | 0.536264 | TGFBR3 | 1 |
| GO:0003677 | MF | DNA binding | 3/27 | 2196/16967 | 0.697526 | 0.75032 | 0.555512 | GADD45A/NR3C2/DACH1 | 3 |
| GO:0098772 | MF | molecular function regulator | 2/27 | 1492/16967 | 0.700059 | 0.75032 | 0.555512 | PCOLCE2/CCL5 | 2 |
| GO:0004930 | MF | G protein-coupled receptor activity | 1/27 | 763/16967 | 0.71157 | 0.758766 | 0.561766 | HPGD | 1 |
| GO:0000981 | MF | DNA-binding transcription factor activity, RNA polymerase II-specific | 2/27 | 1586/16967 | 0.73281 | 0.777448 | 0.575597 | NR3C2/DACH1 | 2 |
| GO:0003700 | MF | DNA-binding transcription factor activity | 2/27 | 1663/16967 | 0.757452 | 0.799532 | 0.591948 | NR3C2/DACH1 | 2 |
| GO:0035639 | MF | purine ribonucleoside triphosphate binding | 2/27 | 1732/16967 | 0.77793 | 0.817022 | 0.604897 | RAB13/NLRC3 | 2 |
| GO:0032555 | MF | purine ribonucleotide binding | 2/27 | 1790/16967 | 0.79402 | 0.825399 | 0.611099 | RAB13/NLRC3 | 2 |
| GO:0017076 | MF | purine nucleotide binding | 2/27 | 1798/16967 | 0.796161 | 0.825399 | 0.611099 | RAB13/NLRC3 | 2 |
| GO:0032553 | MF | ribonucleotide binding | 2/27 | 1804/16967 | 0.797754 | 0.825399 | 0.611099 | RAB13/NLRC3 | 2 |
| GO:0140110 | MF | transcription regulator activity | 2/27 | 1977/16967 | 0.839392 | 0.864201 | 0.639827 | NR3C2/DACH1 | 2 |
| GO:0005524 | MF | ATP binding | 1/27 | 1451/16967 | 0.910695 | 0.928017 | 0.687074 | NLRC3 | 1 |
| GO:0046872 | MF | metal ion binding | 4/27 | 4068/16967 | 0.917036 | 0.928017 | 0.687074 | F5/RRM2/NR3C2/APOBEC3B | 4 |
| GO:0032559 | MF | adenyl ribonucleotide binding | 1/27 | 1502/16967 | 0.918297 | 0.928017 | 0.687074 | NLRC3 | 1 |
| GO:0030554 | MF | adenyl nucleotide binding | 1/27 | 1509/16967 | 0.919291 | 0.928017 | 0.687074 | NLRC3 | 1 |
| GO:0043169 | MF | cation binding | 4/27 | 4133/16967 | 0.923577 | 0.928017 | 0.687074 | F5/RRM2/NR3C2/APOBEC3B | 4 |
| GO:0008144 | MF | drug binding | 1/27 | 1658/16967 | 0.937877 | 0.937877 | 0.694374 | NLRC3 | 1 |

# ****Supplementary Table 5. KEGG function enrichment analysis****

| **ID** | **Description** | **GeneRatio** | **BgRatio** | **pvalue** | **p.adjust** | **qvalue** | **geneID** | **Count** |
| --- | --- | --- | --- | --- | --- | --- | --- | --- |
| hsa05020 | Prion diseases | 2/18 | 35/7914 | 0.00278092893919211 | 0.164074807412334 | 0.149291974630313 | CCL5/C1QA | 2 |
| hsa04115 | p53 signaling pathway | 2/18 | 72/7914 | 0.0113668607823767 | 0.266946466780447 | 0.242895090201656 | GADD45A/RRM2 | 2 |
| hsa04610 | Complement and coagulation cascades | 2/18 | 79/7914 | 0.0135735491583278 | 0.266946466780447 | 0.242895090201656 | F5/C1QA | 2 |
| hsa05142 | Chagas disease (American trypanosomiasis) | 2/18 | 102/7914 | 0.0220044702268 | 0.3245659358453 | 0.295323153043895 | CCL5/C1QA | 2 |
| hsa04110 | Cell cycle | 2/18 | 124/7914 | 0.0316329818901451 | 0.33048023355547 | 0.300704583609794 | CDC25B/GADD45A | 2 |
| hsa00604 | Glycosphingolipid biosynthesis - ganglio series | 1/18 | 15/7914 | 0.033608159344624 | 0.33048023355547 | 0.300704583609794 | ST6GALNAC3 | 1 |
| hsa00770 | Pantothenate and CoA biosynthesis | 1/18 | 19/7914 | 0.0423884941457685 | 0.342221231578731 | 0.311387739705893 | VNN1 | 1 |
| hsa03010 | Ribosome | 2/18 | 153/7914 | 0.046402878858133 | 0.342221231578731 | 0.311387739705893 | RPL10A/RPL36 | 2 |
| hsa05202 | Transcriptional misregulation in cancer | 2/18 | 186/7914 | 0.0657093846647619 | 0.345207999553406 | 0.314105405481244 | GADD45A/HPGD | 2 |
| hsa00052 | Galactose metabolism | 1/18 | 31/7914 | 0.0682796425926243 | 0.345207999553406 | 0.314105405481244 | MGAM | 1 |
| hsa00500 | Starch and sucrose metabolism | 1/18 | 36/7914 | 0.0788712761642959 | 0.345207999553406 | 0.314105405481244 | MGAM | 1 |
| hsa04960 | Aldosterone-regulated sodium reabsorption | 1/18 | 37/7914 | 0.0809759114815136 | 0.345207999553406 | 0.314105405481244 | NR3C2 | 1 |
| hsa05216 | Thyroid cancer | 1/18 | 37/7914 | 0.0809759114815136 | 0.345207999553406 | 0.314105405481244 | GADD45A | 1 |
| hsa04973 | Carbohydrate digestion and absorption | 1/18 | 47/7914 | 0.101773961149657 | 0.345207999553406 | 0.314105405481244 | MGAM | 1 |
| hsa00480 | Glutathione metabolism | 1/18 | 56/7914 | 0.120111401697373 | 0.345207999553406 | 0.314105405481244 | RRM2 | 1 |
| hsa00240 | Pyrimidine metabolism | 1/18 | 57/7914 | 0.122126926610767 | 0.345207999553406 | 0.314105405481244 | RRM2 | 1 |
| hsa05213 | Endometrial cancer | 1/18 | 58/7914 | 0.124138090581875 | 0.345207999553406 | 0.314105405481244 | GADD45A | 1 |
| hsa04623 | Cytosolic DNA-sensing pathway | 1/18 | 63/7914 | 0.134128806571634 | 0.345207999553406 | 0.314105405481244 | CCL5 | 1 |
| hsa05217 | Basal cell carcinoma | 1/18 | 63/7914 | 0.134128806571634 | 0.345207999553406 | 0.314105405481244 | GADD45A | 1 |
| hsa05223 | Non-small cell lung cancer | 1/18 | 66/7914 | 0.140071470685117 | 0.345207999553406 | 0.314105405481244 | GADD45A | 1 |
| hsa04010 | MAPK signaling pathway | 2/18 | 295/7914 | 0.143406508517231 | 0.345207999553406 | 0.314105405481244 | CDC25B/GADD45A | 2 |
| hsa05120 | Epithelial cell signaling in Helicobacter pylori infection | 1/18 | 70/7914 | 0.147935120778973 | 0.345207999553406 | 0.314105405481244 | CCL5 | 1 |
| hsa05218 | Melanoma | 1/18 | 72/7914 | 0.15184143028419 | 0.345207999553406 | 0.314105405481244 | GADD45A | 1 |
| hsa05212 | Pancreatic cancer | 1/18 | 75/7914 | 0.157669194953476 | 0.345207999553406 | 0.314105405481244 | GADD45A | 1 |
| hsa05214 | Glioma | 1/18 | 75/7914 | 0.157669194953476 | 0.345207999553406 | 0.314105405481244 | GADD45A | 1 |
| hsa05133 | Pertussis | 1/18 | 76/7914 | 0.159603364425455 | 0.345207999553406 | 0.314105405481244 | C1QA | 1 |
| hsa05220 | Chronic myeloid leukemia | 1/18 | 76/7914 | 0.159603364425455 | 0.345207999553406 | 0.314105405481244 | GADD45A | 1 |
| hsa00983 | Drug metabolism - other enzymes | 1/18 | 79/7914 | 0.165380736722923 | 0.345207999553406 | 0.314105405481244 | RRM2 | 1 |
| hsa05210 | Colorectal cancer | 1/18 | 86/7914 | 0.178715738606287 | 0.345207999553406 | 0.314105405481244 | GADD45A | 1 |
| hsa05222 | Small cell lung cancer | 1/18 | 92/7914 | 0.189985332909944 | 0.345207999553406 | 0.314105405481244 | GADD45A | 1 |
| hsa05323 | Rheumatoid arthritis | 1/18 | 93/7914 | 0.191849340070212 | 0.345207999553406 | 0.314105405481244 | CCL5 | 1 |
| hsa04974 | Protein digestion and absorption | 1/18 | 95/7914 | 0.195565207666528 | 0.345207999553406 | 0.314105405481244 | DPP4 | 1 |
| hsa05150 | Staphylococcus aureus infection | 1/18 | 96/7914 | 0.1974170846664 | 0.345207999553406 | 0.314105405481244 | C1QA | 1 |
| hsa04914 | Progesterone-mediated oocyte maturation | 1/18 | 98/7914 | 0.201108766360036 | 0.345207999553406 | 0.314105405481244 | CDC25B | 1 |
| hsa04061 | Viral protein interaction with cytokine and cytokine receptor | 1/18 | 100/7914 | 0.204784406514732 | 0.345207999553406 | 0.314105405481244 | CCL5 | 1 |
| hsa04620 | Toll-like receptor signaling pathway | 1/18 | 104/7914 | 0.212087824443859 | 0.347588378949657 | 0.316271317153122 | CCL5 | 1 |
| hsa04668 | TNF signaling pathway | 1/18 | 112/7914 | 0.226505032390387 | 0.361183700298184 | 0.328641725516635 | CCL5 | 1 |
| hsa00230 | Purine metabolism | 1/18 | 130/7914 | 0.258038053592974 | 0.386056325417352 | 0.351273373707136 | RRM2 | 1 |
| hsa04068 | FoxO signaling pathway | 1/18 | 131/7914 | 0.259753792934615 | 0.386056325417352 | 0.351273373707136 | GADD45A | 1 |
| hsa05322 | Systemic lupus erythematosus | 1/18 | 133/7914 | 0.263174036523828 | 0.386056325417352 | 0.351273373707136 | C1QA | 1 |
| hsa04210 | Apoptosis | 1/18 | 136/7914 | 0.268276429527312 | 0.386056325417352 | 0.351273373707136 | GADD45A | 1 |
| hsa05224 | Breast cancer | 1/18 | 147/7914 | 0.286701153160816 | 0.389904855076141 | 0.354775158053224 | GADD45A | 1 |
| hsa05226 | Gastric cancer | 1/18 | 149/7914 | 0.290003670569263 | 0.389904855076141 | 0.354775158053224 | GADD45A | 1 |
| hsa04150 | mTOR signaling pathway | 1/18 | 153/7914 | 0.296565430927546 | 0.389904855076141 | 0.354775158053224 | GRB10 | 1 |
| hsa05160 | Hepatitis C | 1/18 | 155/7914 | 0.299824792602395 | 0.389904855076141 | 0.354775158053224 | IFIT1 | 1 |
| hsa04218 | Cellular senescence | 1/18 | 160/7914 | 0.307910907638484 | 0.389904855076141 | 0.354775158053224 | GADD45A | 1 |
| hsa05225 | Hepatocellular carcinoma | 1/18 | 168/7914 | 0.320665503589449 | 0.389904855076141 | 0.354775158053224 | GADD45A | 1 |
| hsa04530 | Tight junction | 1/18 | 169/7914 | 0.322244127516042 | 0.389904855076141 | 0.354775158053224 | RAB13 | 1 |
| hsa05164 | Influenza A | 1/18 | 170/7914 | 0.323819286419168 | 0.389904855076141 | 0.354775158053224 | CCL5 | 1 |
| hsa04621 | NOD-like receptor signaling pathway | 1/18 | 181/7914 | 0.340919380434522 | 0.402284868912735 | 0.36603975583496 | CCL5 | 1 |
| hsa04062 | Chemokine signaling pathway | 1/18 | 189/7914 | 0.353098390277768 | 0.408486373066438 | 0.371682516081861 | CCL5 | 1 |
| hsa05169 | Epstein-Barr virus infection | 1/18 | 201/7914 | 0.370969059430417 | 0.42090720204605 | 0.382984251638689 | GADD45A | 1 |
| hsa05170 | Human immunodeficiency virus 1 infection | 1/18 | 212/7914 | 0.386940004023004 | 0.430744532780326 | 0.391935257302348 | APOBEC3B | 1 |
| hsa05163 | Human cytomegalovirus infection | 1/18 | 225/7914 | 0.405320991505249 | 0.44285071294092 | 0.402950693309312 | CCL5 | 1 |
| hsa04060 | Cytokine-cytokine receptor interaction | 1/18 | 294/7914 | 0.494482465528292 | 0.530444826657622 | 0.482652741472591 | CCL5 | 1 |
| hsa05206 | MicroRNAs in cancer | 1/18 | 310/7914 | 0.513272022006533 | 0.540768737471169 | 0.492046487261902 | CDC25B | 1 |
| hsa01100 | Metabolic pathways | 3/18 | 1439/7914 | 0.66137333028205 | 0.684579412046333 | 0.622900089462319 | ST6GALNAC3/RRM2/MGAM | 3 |
| hsa05168 | Herpes simplex virus 1 infection | 1/18 | 491/7914 | 0.684684553066415 | 0.696489459153767 | 0.633737063636791 | CCL5 | 1 |
| hsa05200 | Pathways in cancer | 1/18 | 530/7914 | 0.713240385761641 | 0.713240385761641 | 0.648978763137265 | GADD45A | 1 |

# ****Supplementary Table 6. Model and gene ID****

| No | Model | Gene ID | Train (AUC) | GSE67401 (AUC) |
| --- | --- | --- | --- | --- |
| 1 | Lasso+Stepglm[both] | NR3C2, PLEKHO1, CEACAM1, CDC25B, HEPACAM2, VNN1, SLC2A3, IFIT1, ST6GALNAC3, RPL36 | 1 | 0.5 |
| 2 | Lasso+Stepglm[forward] | NR3C2, RAB13, TGFBR3, PLEKHO1, CEACAM1, GADD45A, DACH1, NLRC3, CDC25B, GRB10, PRC1, RRM2, CCL5, DPP4, F5, HEPACAM2, KIAA0101, PCOLCE2, VNN1, APOBEC3B, MGAM, SLC2A3, IFIT1, C1QA, ST6GALNAC3, RPL36, HPGD, RPL10A | 1 | 0.5 |
| 3 | Lasso+Stepglm[backward] | NR3C2, PLEKHO1, CEACAM1, CDC25B, HEPACAM2, VNN1, SLC2A3, IFIT1, ST6GALNAC3, RPL36 | 1 | 0.5 |
| 4 | SVM | NR3C2, RAB13, TGFBR3, PLEKHO1, CEACAM1, GADD45A, DACH1, NLRC3, CDC25B, GRB10, PRC1, RRM2, CCL5, DPP4, F5, HEPACAM2, KIAA0101, PCOLCE2, VNN1, APOBEC3B, MGAM, SLC2A3, IFIT1, C1QA, ST6GALNAC3, RPL36, HPGD, RPL10A | 1 | 0.5 |
| 5 | glmBoost+SVM | NR3C2, PLEKHO1, CEACAM1, GADD45A, CDC25B, DPP4, HEPACAM2, KIAA0101, VNN1, APOBEC3B, SLC2A3, C1QA, RPL36, HPGD, RPL10A | 0.955662862159789 | 0.5 |
| 6 | Ridge | NR3C2, RAB13, TGFBR3, PLEKHO1, CEACAM1, GADD45A, DACH1, NLRC3, CDC25B, GRB10, PRC1, RRM2, CCL5, DPP4, F5, HEPACAM2, KIAA0101, PCOLCE2, VNN1, APOBEC3B, MGAM, SLC2A3, IFIT1, C1QA, ST6GALNAC3, RPL36, HPGD, RPL10A | 0.983318700614574 | 0.645413142485361 |
| 7 | Lasso+SVM | NR3C2, PLEKHO1, CEACAM1, GADD45A, CDC25B, DPP4, HEPACAM2, KIAA0101, VNN1, APOBEC3B, SLC2A3, IFIT1, C1QA, ST6GALNAC3, RPL36, HPGD, RPL10A | 0.941176470588235 | 0.5 |
| 8 | glmBoost+Ridge | NR3C2, PLEKHO1, CEACAM1, GADD45A, CDC25B, DPP4, HEPACAM2, KIAA0101, VNN1, APOBEC3B, SLC2A3, C1QA, RPL36, HPGD, RPL10A | 0.974539069359087 | 0.64053350683149 |
| 9 | Enet[alpha=0.1] | NR3C2, TGFBR3, PLEKHO1, CEACAM1, GADD45A, NLRC3, CDC25B, GRB10, RRM2, CCL5, DPP4, F5, HEPACAM2, KIAA0101, PCOLCE2, VNN1, APOBEC3B, MGAM, SLC2A3, IFIT1, C1QA, ST6GALNAC3, RPL36, HPGD, RPL10A | 0.985074626865672 | 0.643786597267404 |
| 10 | glmBoost+Enet[alpha=0.1] | NR3C2, PLEKHO1, CEACAM1, GADD45A, CDC25B, DPP4, HEPACAM2, KIAA0101, VNN1, APOBEC3B, SLC2A3, C1QA, RPL36, HPGD, RPL10A | 0.974539069359087 | 0.64508783344177 |
| 11 | Enet[alpha=0.2] | NR3C2, TGFBR3, PLEKHO1, CEACAM1, GADD45A, NLRC3, CDC25B, GRB10, RRM2, CCL5, DPP4, F5, HEPACAM2, KIAA0101, PCOLCE2, VNN1, APOBEC3B, MGAM, SLC2A3, IFIT1, C1QA, ST6GALNAC3, RPL36, HPGD, RPL10A | 0.984196663740123 | 0.642160052049447 |
| 12 | Enet[alpha=0.3] | NR3C2, TGFBR3, PLEkHO1, CEACAM1, GADD45A, CDC25B, RRM2, CCL5, DPP4, F5, HEPACAM2, KIAA0101, VNN1, APOBEC3B, SLC2A3, IFIT1, C1QA, ST6GALNAC3, RPL36, HPGD, RPL10A | 0.978050921861282 | 0.637931034482759 |
| 13 | glmBoost+Enet[alpha=0.3] | NR3C2, PLEKHO1, CEACAM1, GADD45A, CDC25B, DPP4, HEPACAM2, KIAA0101, VNN1, APOBEC3B, SLC2A3, C1QA, RPL36, HPGD, RPL10A | 0.97278314310799 | 0.63825634352635 |
| 14 | glmBoost+Enet[alpha=0.2] | NR3C2, PLEKHO1, CEACAM1, GADD45A, CDC25B, DPP4, HEPACAM2, KIAA0101, VNN1, APOBEC3B, SLC2A3, C1QA, RPL36, HPGD, RPL10A | 0.972783143107989 | 0.64053350683149 |
| 15 | Enet[alpha=0.4] | NR3C2, TGFBR3, PLEKHO1, CEACAM1, GADD45A, CDC25B, DPP4, HEPACAM2, KIAA0101, VNN1, APOBEC3B, SLC2A3, IFIT1, C1QA, ST6GALNAC3, RPL36, HPGD, RPL10A | 0.967515364354697 | 0.630448926480156 |
| 16 | glmBoost+Enet[alpha=0.4] | NR3C2, PLEKHO1, CEACAM1, GADD45A, CDC25B, DPP4, HEPACAM2, KIAA0101, VNN1, APOBEC3B, SLC2A3, C1QA, RPL36, HPGD, RPL10A | 0.974539069359087 | 0.64053350683149 |
| 17 | Lasso+glmBoost | NR3C2, PLEKHO1, CEACAM1, GADD45A, CDC25B, DPP4, HEPACAM2, KIAA0101, VNN1, APOBEC3B, SLC2A3, C1QA, RPL36, HPGD, RPL10A | 0.966637401229148 | 0.620689655172414 |
| 18 | Enet[alpha=0.5] | NR3C2, TGFBR3, PLEKHO1, CEACAM1, GADD45A, CDC25B, DPP4, F5, HEPACAM2, KIAA0101, VNN1, APOBEC3B, SLC2A3, IFIT1, C1QA, ST6GALNAC3, RPL36, HPGD, RPL10A | 0.975417032484636 | 0.63825634352635 |
| 19 | glmBoost | NR3C2, PLEkHO1, CEACAM1, GADD45A, CDC25B, DPP4, HEPACAM2, KIAA0101, VNN1, APOBEC3B, SLC2A3, C1QA, RPL36, HPGD, RPL10A | 0.966637401229148 | 0.620689655172414 |
| 20 | glmBoost+Enet[alpha=0.5] | NR3C2, PLEKHO1, CEACAM1, GADD45A, CDC25B, DPP4, HEPACAM2, KIAA0101, VNN1, APOBEC3B, SLC2A3, C1QA, RPL36, HPGD, RPL10A | 0.977172958735733 | 0.646063760572544 |
| 21 | Enet[alpha=0.6] | NR3C2, TGFBR3, PLEKHO1, CEACAM1, GADD45A, CDC25B, DPP4, HEPACAM2, KIAA0101, VNN1, APOBEC3B, SLC2A3, IFIT1, C1QA, ST6GALNAC3, RPL36, HPGD, RPL10A | 0.971905179982441 | 0.63370201691607 |
| 22 | glmBoost+Enet[alpha=0.6] | NR3C2, PLEKHO1, CEACAM1, GADD45A, CDC25B, DPP4, HEPACAM2, KIAA0101, VNN1, APOBEC3B, SLC2A3, C1QA, RPL36, HPGD, RPL10A | 0.977172958735733 | 0.644437215354587 |
| 23 | glmBoost+Enet[alpha=0.7] | NR3C2, PLEkHO1, CEACAM1, GADD45A, CDC25B, DPP4, HEPACAM2, KIAA0101, VNN1, APOBEC3B, SLC2A3, C1QA, RPL36, HPGD, RPL10A | 0.976294995610184 | 0.642485361093038 |
| 24 | glmBoost+Enet[alpha=0.8] | NR3C2, PLEKHO1, CEACAM1, GADD45A, CDC25B, DPP4, HEPACAM2, KIAA0101, VNN1, APOBEC3B, SLC2A3, C1QA, RPL36, HPGD, RPL10A | 0.977172958735733 | 0.641184124918673 |
| 25 | Enet[alpha=0.8] | NR3C2, PLEKHO1, GADD45A, CDC25B, DPP4, KIAA0101, APOBEC3B, SLC2A3 | 0.938542581211589 | 0.592387768379961 |
| 26 | Enet[alpha=0.9] | NR3C2, PLEkHO1, GADD45A, CDC25B, DPP4, HEPACAM2, KIAA0101, VNN1, APOBEC3B, SLC2A3 | 0.949078138718174 | 0.597592713077424 |
| 27 | glmBoost+Enet[alpha=0.9] | NR3C2, PLEkHO1, CEACAM1, GADD45A, CDC25B, DPP4, HEPACAM2, KIAA0101, VNN1, APOBEC3B, SLC2A3, C1QA, RPL36, HPGD, RPL10A | 0.972783143107989 | 0.63370201691607 |
| 28 | Lasso | NR3C2, PLEKHO1, CEACAM1, GADD45A, CDC25B, DPP4, HEPACAM2, KIAA0101, VNN1, APOBEC3B, SLC2A3, RPL10A | 0.954345917471466 | 0.603448275862069 |
| 29 | Lasso+plsRglm | NR3C2, PLEkHO1, CEACAM1, GADD45A, CDC25B, DPP4, HEPACAM2, KIAA0101, VNN1, APOBEC3B, SLC2A3, IFIT1, C1QA, ST6GALNAC3, RPL36, HPGD, RPL10A | 0.979806848112379 | 0.618087182823682 |
| 30 | glmBoost+plsRglm | NR3C2, PLEkHO1, CEACAM1, GADD45A, CDC25B, DPP4, HEPACAM2, KIAA0101, VNN1, APOBEC3B, SLC2A3, C1QA, RPL36, HPGD, RPL10A | 0.973661106233538 | 0.624593363695511 |
| 31 | glmBoost+Stepglm[forward] | NR3C2, PLEkHO1, CEACAM1, GADD45A, CDC25B, DPP4, HEPACAM2, KIAA0101, VNN1, APOBEC3B, SLC2A3, C1QA, RPL36, HPGD, RPL10A | 0.990342405618964 | 0.5 |
| 32 | RF+SVM | CDC25B, APOBEC3B, PLEKHO1, GADD45A, C1QA, RPL10A, IFIT1, DPP4, NR3C2, KIAA0101, GRB10 | 0.941176470588235 | 0.5 |
| 33 | Stepglm[forward] | NR3C2, RAB13, TGFBR3, PLEkHO1, CEACAM1, GADD45A, DACH1, NLRC3, CDC25B, GRB10, PRC1, RRM2, CCL5, DPP4, F5, HEPACAM2, KIAA0101, PCOLCE2, VNN1, APOBEC3B, MGAM, SLC2A3, IFIT1, C1QA, ST6GALNAC3, RPL36, HPGD, RPL10A | 1 | 0.490566037735849 |
| 34 | plsRglm | NR3C2, RAB13, TGFBR3, PLEkHO1, CEACAM1, GADD45A, DACH1, NLRC3, CDC25B, GRB10, PRC1, RRM2, CCL5, DPP4, F5, HEPACAM2, KIAA0101, PCOLCE2, VNN1, APOBEC3B, MGAM, SLC2A3, IFIT1, C1QA, ST6GALNAC3, RPL36, HPGD, RPL10A | 0.980684811237928 | 0.632400780741705 |
| 35 | RF+Ridge | CDC25B, APOBEC3B, PLEkHO1, GADD45A, C1QA, RPL10A, IFIT1, DPP4, NR3C2, KIAA0101, GRB10 | 0.943810359964881 | 0.550422901756669 |
| 36 | RF+Enet[alpha=0.1] | CDC25B, APOBEC3B, PLEkHO1, GADD45A, C1QA, RPL10A, IFIT1, DPP4, NR3C2, KIAA0101, GRB10 | 0.94468832309043 | 0.548796356538712 |
| 37 | RF+plsRglm | CDC25B, APOBEC3B, PLEkHO1, GADD45A, NR3C2, KIAA0101 | 0.923617208077261 | 0.586532205595316 |
| 38 | RF+Stepglm[forward] | CDC25B, APOBEC3B, PLEkHO1, GADD45A, C1QA, RPL10A, IFIT1, DPP4, NR3C2, KIAA0101, GRB10 | 0.955223880597015 | 0.539037085230969 |
| 39 | RF+Enet[alpha=0.2] | CDC25B, APOBEC3B, PLEkHO1, GADD45A, C1QA, RPL10A, IFIT1, DPP4, NR3C2, KIAA0101, GRB10 | 0.945566286215979 | 0.545868575146389 |
| 40 | RF+Enet[alpha=0.3] | CDC25B, APOBEC3B, PLEkHO1, GADD45A, C1QA, RPL10A, IFIT1, DPP4, NR3C2, KIAA0101, GRB10 | 0.947322212467076 | 0.546519193233572 |
| 41 | RF+Enet[alpha=0.6] | CDC25B, APOBEC3B, PLEkHO1, GADD45A, C1QA, RPL10A, IFIT1, DPP4, NR3C2, KIAA0101 | 0.950834064969271 | 0.543266102797658 |
| 42 | RF+Lasso | CDC25B, APOBEC3B, PLEkHO1, GADD45A, C1QA, RPL10A, IFIT1, DPP4, NR3C2, KIAA0101 | 0.950834064969271 | 0.544242029928432 |
| 43 | RF+Enet[alpha=0.7] | CDC25B, APOBEC3B, PLEkHO1, GADD45A, C1QA, RPL10A, IFIT1, DPP4, NR3C2, KIAA0101, GRB10 | 0.95171202809482 | 0.545217957059206 |
| 44 | RF+Enet[alpha=0.5] | CDC25B, APOBEC3B, PLEKHO1, GADD45A, C1QA, RPL10A, IFIT1, DPP4, NR3C2, KIAA0101, GRB10 | 0.949956101843723 | 0.543916720884841 |
| 45 | RF+glmBoost | CDC25B, APOBEC3B, PLEKHO1, GADD45A, C1QA, RPL10A, IFIT1, DPP4, NR3C2, KIAA0101 | 0.946444249341528 | 0.55757970071568 |
| 46 | RF+Enet[alpha=0.9] | CDC25B, APOBEC3B, PLEKHO1, GADD45A, C1QA, RPL10A, IFIT1, DPP4, NR3C2, KIAA0101 | 0.950834064969271 | 0.543916720884841 |
| 47 | RF+Enet[alpha=0.4] | CDC25B, APOBEC3B, PLEKHO1, GADD45A, C1QA, RPL10A, IFIT1, DPP4, NR3C2, KIAA0101, GRB10 | 0.948200175592625 | 0.544242029928432 |
| 48 | RF+Enet[alpha=0.8] | CDC25B, APOBEC3B, PLEKHO1, GADD45A, C1QA, RPL10A, IFIT1, DPP4, NR3C2, KIAA0101 | 0.95171202809482 | 0.544567338972023 |
| 49 | RF+Stepglm[both] | CDC25B, APOBEC3B, PLEKHO1, RPL10A, IFIT1, DPP4, NR3C2 | 0.954345917471466 | 0.53220559531555 |
| 50 | RF+Stepglm[backward] | CDC25B, APOBEC3B, PLEKHO1, RPL10A, IFIT1, DPP4, NR3C2 | 0.954345917471466 | 0.53220559531555 |
| 51 | Stepglm[both]+Ridge | NR3C2, PLEKHO1, CDC25B, PRC1, RRM2, HEPACAM2, VNN1, APOBEC3B, SLC2A3, IFIT1 | 0.9657594381036 | 0.623292127521145 |
| 52 | Stepglm[backward]+Ridge | NR3C2, PLEKHO1, CDC25B, PRC1, RRM2, HEPACAM2, VNN1, APOBEC3B, SLC2A3, IFIT1 | 0.955223880597015 | 0.622966818477554 |
| 53 | Stepglm[both]+plsRglm | NR3C2, PLEKHO1, CDC25B, HEPACAM2, APOBEC3B, SLC2A3 | 0.942932396839333 | 0.622316200390371 |
| 54 | Stepglm[backward]+plsRglm | NR3C2, PLEKHO1, CDC25B, HEPACAM2, APOBEC3B, SLC2A3 | 0.942932396839333 | 0.622316200390371 |
| 55 | Stepglm[both]+Enet[alpha=0.9] | NR3C2, PLEKHO1, CDC25B, PRC1, RRM2, HEPACAM2, VNN1, APOBEC3B, SLC2A3, IFIT1 | 0.966637401229148 | 0.625894599869876 |
| 56 | Stepglm[backward]+Enet[alpha=0.9] | NR3C2, PLEKHO1, CDC25B, PRC1, RRM2, HEPACAM2, VNN1, APOBEC3B, SLC2A3, IFIT1 | 0.963125548726953 | 0.624593363695511 |
| 57 | Stepglm[both]+Enet[alpha=0.1] | NR3C2, PLEKHO1, CDC25B, PRC1, RRM2, HEPACAM2, VNN1, APOBEC3B, SLC2A3, IFIT1 | 0.967515364354697 | 0.624918672739102 |
| 58 | Stepglm[backward]+Enet[alpha=0.1] | NR3C2, PLEKHO1, CDC25B, PRC1, RRM2, HEPACAM2, VNN1, APOBEC3B, SLC2A3, IFIT1 | 0.963125548726953 | 0.624593363695511 |
| 59 | Stepglm[both]+Enet[alpha=0.8] | NR3C2, PLEKHO1, CDC25B, PRC1, HEPACAM2, VNN1, APOBEC3B, SLC2A3, IFIT1 | 0.95873573309921 | 0.623617436564737 |
| 60 | Stepglm[backward]+Enet[alpha=0.8] | NR3C2, PLEKHO1, CDC25B, PRC1, RRM2, HEPACAM2, VNN1, APOBEC3B, SLC2A3, IFIT1 | 0.970149253731343 | 0.626870527000651 |
| 61 | Stepglm[both]+Enet[alpha=0.2] | NR3C2, PLEKHO1, CDC25B, PRC1, RRM2, HEPACAM2, VNN1, APOBEC3B, SLC2A3, IFIT1 | 0.967515364354697 | 0.624593363695511 |
| 62 | Stepglm[backward]+Enet[alpha=0.2] | NR3C2, PLEKHO1, CDC25B, PRC1, RRM2, HEPACAM2, VNN1, APOBEC3B, SLC2A3, IFIT1 | 0.971027216856892 | 0.626545217957059 |
| 63 | Stepglm[both]+Lasso | NR3C2, PLEKHO1, CDC25B, HEPACAM2, VNN1, APOBEC3B, SLC2A3, IFIT1 | 0.952589991220369 | 0.623292127521145 |
| 64 | Stepglm[backward]+Lasso | NR3C2, PLEKHO1, CDC25B, HEPACAM2, VNN1, APOBEC3B, SLC2A3, IFIT1 | 0.955223880597015 | 0.621990891346779 |
| 65 | Stepglm[both]+Enet[alpha=0.6] | NR3C2, PLEKHO1, CDC25B, PRC1, RRM2, HEPACAM2, VNN1, APOBEC3B, SLC2A3, IFIT1 | 0.970149253731343 | 0.626545217957059 |
| 66 | Stepglm[backward]+Enet[alpha=0.6] | NR3C2, PLEKHO1, CDC25B, PRC1, RRM2, HEPACAM2, VNN1, APOBEC3B, SLC2A3, IFIT1 | 0.971027216856892 | 0.627521145087833 |
| 67 | glmBoost+GBM | CDC25B, APOBEC3B, PLEKHO1, DPP4, KIAA0101, HPGD, NR3C2, SLC2A3, RPL36, HEPACAM2, VNN1, CEACAM1, RPL10A, GADD45A, C1QA | 1 | 0.595966167859467 |
| 68 | Stepglm[both]+Enet[alpha=0.7] | NR3C2, PLEKHO1, CDC25B, PRC1, HEPACAM2, VNN1, APOBEC3B, SLC2A3, IFIT1 | 0.957857769973661 | 0.624593363695511 |
| 69 | Stepglm[backward]+Enet[alpha=0.7] | NR3C2, PLEKHO1, CDC25B, PRC1, RRM2, HEPACAM2, VNN1, APOBEC3B, SLC2A3, IFIT1 | 0.971027216856892 | 0.626219908913468 |
| 70 | glmBoost+Lasso | NR3C2, PLEKHO1, CEACAM1, GADD45A, CDC25B, DPP4, HEPACAM2, KIAA0101, VNN1, APOBEC3B, SLC2A3, C1QA, RPL36, HPGD, RPL10A | 0.976294995610184 | 0.646714378659727 |
| 71 | Stepglm[both] | NR3C2, PLEKHO1, CDC25B, PRC1, RRM2, HEPACAM2, VNN1, APOBEC3B, SLC2A3, IFIT1 | 1 | 0.5 |
| 72 | Stepglm[backward] | NR3C2, PLEKHO1, CDC25B, PRC1, RRM2, HEPACAM2, VNN1, APOBEC3B, SLC2A3, IFIT1 | 1 | 0.5 |
| 73 | glmBoost+Stepglm[both] | NR3C2, PLEKHO1, CEACAM1, CDC25B, HEPACAM2, VNN1, SLC2A3, RPL36 | 0.978050921861282 | 0.671437865972674 |
| 74 | glmBoost+Stepglm[backward] | NR3C2, PLEKHO1, CEACAM1, CDC25B, HEPACAM2, VNN1, SLC2A3, RPL36 | 0.978050921861282 | 0.671437865972674 |
| 75 | Stepglm[both]+Enet[alpha=0.4] | NR3C2, PLEKHO1, CDC25B, PRC1, RRM2, HEPACAM2, VNN1, APOBEC3B, SLC2A3, IFIT1 | 0.971905179982441 | 0.628171763175016 |
| 76 | Stepglm[backward]+Enet[alpha=0.4] | NR3C2, PLEKHO1, CDC25B, PRC1, RRM2, HEPACAM2, VNN1, APOBEC3B, SLC2A3, IFIT1 | 0.95873573309921 | 0.623942745608328 |
| 77 | Stepglm[both]+Enet[alpha=0.3] | NR3C2, PLEKHO1, CDC25B, PRC1, RRM2, HEPACAM2, VNN1, APOBEC3B, SLC2A3, IFIT1 | 0.967515364354697 | 0.625243981782694 |
| 78 | Stepglm[backward]+Enet[alpha=0.3] | NR3C2, PLEKHO1, CDC25B, PRC1, RRM2, HEPACAM2, VNN1, APOBEC3B, SLC2A3, IFIT1 | 0.966637401229148 | 0.625569290826285 |
| 79 | Stepglm[both]+glmBoost | NR3C2, PLEKHO1, CDC25B, HEPACAM2, VNN1, APOBEC3B, SLC2A3, IFIT1 | 0.942932396839333 | 0.626870527000651 |
| 80 | Stepglm[backward]+glmBoost | NR3C2, PLEKHO1, CDC25B, HEPACAM2, VNN1, APOBEC3B, SLC2A3, IFIT1 | 0.942932396839333 | 0.626870527000651 |
| 81 | Stepglm[both]+Enet[alpha=0.5] | NR3C2, PLEKHO1, CDC25B, PRC1, RRM2, HEPACAM2, VNN1, APOBEC3B, SLC2A3, IFIT1 | 0.961369622475856 | 0.624268054651919 |
| 82 | Stepglm[backward]+Enet[alpha=0.5] | NR3C2, PLEKHO1, CDC25B, PRC1, RRM2, HEPACAM2, VNN1, APOBEC3B, SLC2A3, IFIT1 | 0.9657594381036 | 0.624918672739102 |
| 83 | glmBoost+RF | CDC25B, APOBEC3B, PLEKHO1, C1QA, RPL10A, GADD45A | 1 | 0.611581001951854 |
| 84 | RF | CDC25B, APOBEC3B, PLEKHO1, GADD45A, C1QA, RPL10A, NR3C2, IFIT1, DPP4, GRB10 | 1 | 0.612556929082628 |
| 85 | Lasso+GBM | CDC25B, APOBEC3B, PLEKHO1, DPP4, KIAA0101, HEPACAM2, RPL36, HPGD, SLC2A3, NR3C2, CEACAM1, VNN1, ST6GALNAC3, RPL10A, GADD45A, C1QA, IFIT1 | 0.999122036874451 | 0.584580351333767 |
| 86 | RF+GBM | CDC25B, APOBEC3B, PLEKHO1, DPP4, KIAA0101, GRB10, NR3C2, RPL10A, GADD45A, IFIT1, C1QA | 0.992098331870061 | 0.567664281067014 |
| 87 | GBM | NR3C2, APOBEC3B, PLEKHO1, DPP4, KIAA0101, HPGD, GRB10, SLC2A3, HEPACAM2, RPL36, CEACAM1, TGFBR3, MGAM, VNN1, ST6GALNAC3, RPL10A, RAB13, GADD45A, RRM2, PRC1, C1QA, IFIT1, PCOLCE2, F5, NLRC3, DACH1 | 1 | 0.584255042290176 |
| 88 | Stepglm[both]+SVM | NR3C2, PLEKHO1, CDC25B, PRC1, RRM2, HEPACAM2, VNN1, APOBEC3B, SLC2A3, IFIT1 | 0.970588235294118 | 0.490566037735849 |
| 89 | Stepglm[backward]+SVM | NR3C2, PLEKHO1, CDC25B, PRC1, RRM2, HEPACAM2, VNN1, APOBEC3B, SLC2A3, IFIT1 | 0.970588235294118 | 0.490566037735849 |
| 90 | Lasso+RF | CDC25B, APOBEC3B, PLEKHO1, GADD45A, C1QA, RPL10A, NR3C2 | 1 | 0.599544567338972 |
| 91 | Stepglm[both]+GBM | CDC25B, APOBEC3B, PLEKHO1, HEPACAM2, SLC2A3, NR3C2, VNN1, PRC1, IFIT1, RRM2 | 0.989464442493415 | 0.532856213402733 |
| 92 | Stepglm[backward]+GBM | CDC25B, APOBEC3B, PLEKHO1, HEPACAM2, SLC2A3, NR3C2, VNN1, PRC1, IFIT1, RRM2 | 0.991220368744513 | 0.53448275862069 |
| 93 | LDA | NR3C2, RAB13, TGFBR3, PLEKHO1, CEACAM1, GADD45A, DACH1, NLRC3, CDC25B, GRB10, PRC1, RRM2, CCL5, DPP4, F5, HEPACAM2, KIAA0101, PCOLCE2, VNN1, APOBEC3B, MGAM, SLC2A3, IFIT1, C1QA, ST6GALNAC3, RPL36, HPGD, RPL10A | 0.978928884986831 | 0.666558230318803 |
| 94 | glmBoost+LDA | NR3C2, PLEKHO1, CEACAM1, GADD45A, CDC25B, DPP4, HEPACAM2, KIAA0101, VNN1, APOBEC3B, SLC2A3, C1QA, RPL36, HPGD, RPL10A | 0.97278314310799 | 0.656798959011061 |
| 95 | RF+LDA | CDC25B, APOBEC3B, PLEKHO1, GADD45A, C1QA, RPL10A, IFIT1, DPP4, NR3C2, KIAA0101, GRB10 | 0.939420544337138 | 0.55757970071568 |
| 96 | Stepglm[both]+LDA | NR3C2, PLEKHO1, CDC25B, PRC1, RRM2, HEPACAM2, VNN1, APOBEC3B, SLC2A3, IFIT1 | 0.942932396839333 | 0.639557579700716 |
| 97 | Stepglm[backward]+LDA | NR3C2, PLEKHO1, CDC25B, PRC1, RRM2, HEPACAM2, VNN1, APOBEC3B, SLC2A3, IFIT1 | 0.942932396839333 | 0.639557579700716 |
| 98 | Lasso+LDA | NR3C2, PLEKHO1, CEACAM1, GADD45A, CDC25B, DPP4, HEPACAM2, KIAA0101, VNN1, APOBEC3B, SLC2A3, IFIT1, C1QA, ST6GALNAC3, RPL36, HPGD, RPL10A | 0.973661106233538 | 0.658750813272609 |
| 99 | XGBoost | NR3C2, RAB13, TGFBR3, PLEKHO1, CEACAM1, GADD45A, DACH1, NLRC3, CDC25B, GRB10, PRC1, RRM2, CCL5, DPP4, F5, HEPACAM2, KIAA0101, PCOLCE2, VNN1, APOBEC3B, MGAM, SLC2A3, IFIT1, C1QA, ST6GALNAC3, RPL36, HPGD, RPL10A | 1 | 0.607026675341575 |
| 100 | Lasso+XGBoost | NR3C2, PLEKHO1, CEACAM1, GADD45A, CDC25B, DPP4, HEPACAM2, KIAA0101, VNN1, APOBEC3B, SLC2A3, IFIT1, C1QA, ST6GALNAC3, RPL36, HPGD, RPL10A | 1 | 0.565712426805465 |
| 101 | glmBoost+XGBoost | NR3C2, PLEKHO1, CEACAM1, GADD45A, CDC25B, DPP4, HEPACAM2, KIAA0101, VNN1, APOBEC3B, SLC2A3, C1QA, RPL36, HPGD, RPL10A | 0.964003511852502 | 0.516428106701366 |
| 102 | RF+XGBoost | CDC25B, APOBEC3B, PLEKHO1, GADD45A, C1QA, RPL10A, IFIT1, DPP4, NR3C2, KIAA0101, GRB10 | 0.998244073748903 | 0.468282368249837 |
| 103 | Stepglm[both]+XGBoost | NR3C2, PLEKHO1, CDC25B, PRC1, RRM2, HEPACAM2, VNN1, APOBEC3B, SLC2A3, IFIT1 | 0.966637401229148 | 0.466493168510085 |
| 104 | Stepglm[backward]+XGBoost | NR3C2, PLEKHO1, CDC25B, PRC1, RRM2, HEPACAM2, VNN1, APOBEC3B, SLC2A3, IFIT1 | 0.881913959613696 | 0.47462589459987 |
| 105 | NaiveBayes | NR3C2, RAB13, TGFBR3, PLEKHO1, CEACAM1, GADD45A, DACH1, NLRC3, CDC25B, GRB10, PRC1, RRM2, CCL5, DPP4, F5, HEPACAM2, KIAA0101, PCOLCE2, VNN1, APOBEC3B, MGAM, SLC2A3, IFIT1, C1QA, ST6GALNAC3, RPL36, HPGD, RPL10A | 0.977172958735733 | 0.60377358490566 |
| 106 | Lasso+NaiveBayes | NR3C2, PLEKHO1, CEACAM1, GADD45A, CDC25B, DPP4, HEPACAM2, KIAA0101, VNN1, APOBEC3B, SLC2A3, IFIT1, C1QA, ST6GALNAC3, RPL36, HPGD, RPL10A | 0.971027216856892 | 0.60832791151594 |
| 107 | glmBoost+NaiveBayes | NR3C2, PLEKHO1, CEACAM1, GADD45A, CDC25B, DPP4, HEPACAM2, KIAA0101, VNN1, APOBEC3B, SLC2A3, C1QA, RPL36, HPGD, RPL10A | 0.971027216856892 | 0.601496421600521 |
| 108 | RF+NaiveBayes | CDC25B, APOBEC3B, PLEKHO1, GADD45A, C1QA, RPL10A, IFIT1, DPP4, NR3C2, KIAA0101, GRB10 | 0.957857769973661 | 0.486011711125569 |
| 109 | Stepglm[both]+NaiveBayes | NR3C2, PLEKHO1, CDC25B, PRC1, RRM2, HEPACAM2, VNN1, APOBEC3B, SLC2A3, IFIT1 | 0.962247585601405 | 0.585881587508133 |
| 110 | Stepglm[backward]+NaiveBayes | NR3C2, PLEKho1, CDC25B, PRC1, RRM2, HEPACAM2, VNN1, APOBEC3B, SLC2A3, IFIT1 | 0.962247585601405 | 0.585881587508133 |
| 111 | Enet[alpha=0.7] | NR3C2, TGFBR3, PLEKHO1, CEACAM1, GADD45A, CDC25B, DPP4, HEPACAM2, KIAA0101, VNN1, APOBEC3B, SLC2A3, IFIT1, C1QA, ST6GALNAC3, RPL36, HPGD, RPL10A | 0.97278314310799 | 0.633051398828887 |
| 112 | Stepglm[both]+RF | CDC25B, APOBEC3B, PLEKHO1, NR3C2 | 1 | 0.544242029928432 |
| 113 | Stepglm[backward]+RF | CDC25B, APOBEC3B, PLEKHO1, NR3C2 | 1 | 0.540338321405335 |
